# Supplementary material for: 1,3-Difunctionalization of [1.1.1]propellane through iron-hydride catalyzed hydropyridylation
Source: Nat Commun. 2024 Jul 17;15:5993. doi: 10.1038/s41467-024-50356-3 (PMC11252317; doi:10.1038/s41467-024-50356-3)
Supplement: Supplementary file 1 — Supplementary Information [file 41467_2024_50356_MOESM1_ESM.pdf]

Supplementary Information

1,3-Difunctionalization of [1.1.1]Propellane through Iron-Hydride Catalyzed Hydropyridylation

*Changha Kim[a,b], Yuhyun Kim[a,b] and Sungwoo Hong[b,a]**

Email : hongorg@kaist.ac.kr

[a]Department of Chemistry, Korea Advanced Institute of Science and Technology (KAIST), Daejeon, 34141, Korea

[b]Center for Catalytic Hydrocarbon Functionalizations, Institute for Basic Science (IBS), Daejeon, 34141, Korea

**I. General Methods and Materials**	S2
**II. Experimental Procedure**	S2
**III. Optimization Table**	S5
**IV. Control Experiments**	S10
**V. Mechansitc investigation**	S11
**VI. Determination of major diastereomer of 1,3-difunctionalization of [1.1.1]propellane**	S15
**VII. Proposed Mechanism**	S16
**VIII. Compound Characterizations**	S17

Appendix I

**Spectral Copies of ^{1}H-, ^{13}C-, ^{11}B- and ^{19}F-NMR Data Obtained in this Study**	S57

I. General Methods and Materials

Analytical thin layer chromatography (TLC) was performed on precoated silica gel 60 F^{254} plates and silica gel 60 RP-18 F^{254}s, and visualization on TLC was achieved by UV light (254 and 365 nm). Flash column chromatography was performed on silica gel (400-630 mesh) or a Combi*Flash*® R_f^+ system with Redi*Sep*® R_f silica columns (230-400 mesh) using a proper eluent. ^{1}H NMR was recorded on Brucker Avance 400 MHz, Brucker Avance 500 MHz or Agilent Technologies DD2 600 MHz. Chemical shifts were quoted in parts per million (ppm) referenced to the appropriate solvent peak or 0.0 ppm for tetramethylsilane. The following abbreviations were used to describe peak splitting patterns when appropriate: br = broad, s = singlet, d = doublet, t = triplet, q = quartet, m = multiplet, dd = doublet of doublet, td = triplet of doublet, ddd = doublet of doublet of doublet. Coupling constants, *J*, were reported in hertz unit (Hz). ^{13}C NMR was recorded on Brucker Avance 100 MHz, Brucker Avance 125 MHz and was fully decoupled by broad band proton decoupling. Chemical shifts were reported in ppm referenced to the centerline of a pentet at 77.2 ppm of $CDCl_3$. ^{19}F NMR was recorded on Brucker Avance 376 MHz. ^{11}B NMR was recorded on Brucker Avance 128 MHz. High resolution mass spectroscopy was conducted on a Bruker Daltonik micrOTOF-QII and obtained by using ESI from Korea Basic Science Institute (Ochang). Commercial grade reagents and solvents were used without further purification except as indicated below.

II. Experimental Procedure

General procedure 1 for C4/ Markovnikov selective hydropyridylation of alkene (GP1)

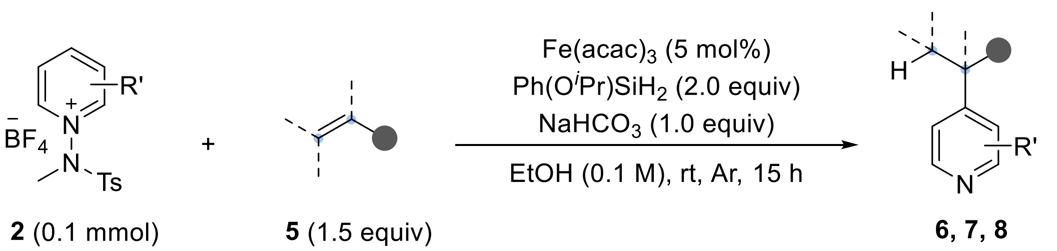

Supplementary Figure 1. General procedure 1 for C4/ Markovnikov selective hydropyridylation of alkene

To a 12 mL test tube equipped with a magnetic bar were added N-amidopyridinium salt **2** (1.0 equiv., 0.10 mmol), olefin **5** (1.5 equiv., 0.15 mmol), sodium bicarbonate (1.0 equiv., 0.10 mmol), and iron catalyst $Fe(acac)_3$ (5 mol%). The tube was then sealed with a PTFE septum, evacuated, and back-filled with argon. Anhydrous ethanol (0.1 M, 1.0 mL) were added using a syringe, followed by $Ph(O^iPr)SiH_2$ (2.0 equiv., 0.20 mmol) via micro syringe. The reaction mixture was stirred at room temperature for 15

h. After reaction completion, the reaction mixture was diluted with ethyl acetate, washed with water and extracted ethyl acetate three times. After removal of solvent, the residue was purified by flash column chromatography on silica gel (eluent: ethyl acetate/*n*-hexane = 1:3) to give the desired product **6-8**.

General procedure 2 for 1,3-difunctionalization of [1.1.1]propellane (GP2)

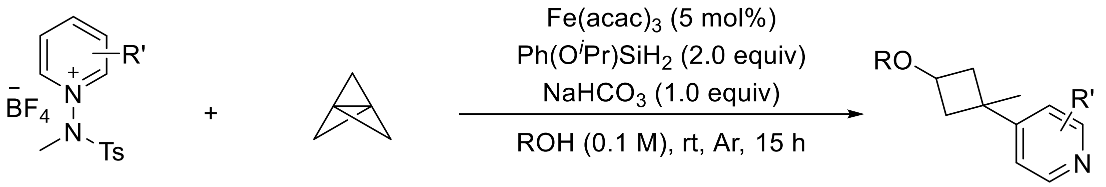

2 (0.1 mmol) **1** (1.5 equiv) **3**

Supplementary Figure 2. General procedure 2 for 1,3-difunctionalization of [1.1.1]propellane

To a 12 mL test tube equipped with a magnetic bar were added N-amidopyridinium salt **2** (1.0 equiv., 0.10 mmol), sodium bicarbonate (1.0 equiv., 0.10 mmol), and iron catalyst Fe(acac)$_3$ (5 mol%). The tube was then sealed with a PTFE septum, evacuated, and back-filled with argon. Alcohol solvent (0.1 M, 1.0 mL) and Ph(O^{i}Pr)SiH$_2$ (2.0 equiv., 0.20 mmol) were added, followed by [1.1.1]propellane **1** (1.5 equiv., 0.15 mmol) via syringe. The reaction mixture was stirred at room temperature for 15 h. After reaction completion, the reaction mixture was diluted with ethyl acetate, washed with water and extracted ethyl acetate three times. After removal of solvent, the residue was purified by flash column chromatography on silica gel (eluent: ethyl acetate/*n*-hexane = 1:3) to give the desired product **3**.

General procedure 3 for synthesis alcohol inserted methylenecyclobutane (GP3)

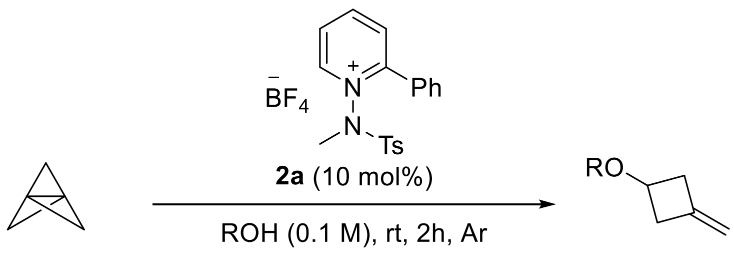

1 (0.1 mmol) **4**

Supplementary Figure 3. General procedure 3 for synthesis alcohol inserted methylenecyclobutane

To a 12 mL test tube equipped with a magnetic bar were added N-amidopyridinium salt **2** (10 mol%, 0.01 mmol). The tube was then sealed with a PTFE septum, evacuated, and back-filled with argon.

Alcohol solvent (0.1 M, 1.0 mL) were added, followed by [1.1.1]propellane **1** (1.0 equiv., 0.10 mmol) via syringe. The reaction mixture was stirred at room temperature for 2 h. After reaction completion, the reaction mixture was evaporated under vaccum. The residue was purified by flash column chromatography on silica gel (eluent: dichloromethane/*n*-hexane = 1:6) to give the desired product **4**. In the case of measuring the NMR yield. After reaction completion, add the interneral standard 1,1,2,2-tetrabromoethane to the reaction mixture. Subsequently, a 50 μl portion of the reaction mixture was dissolved in 1.0 ml of $CDCl_3$ for NMR measurement.

General procedure 4 for Preparation of *N*-protected amidopyridinium salts (GP4)

Preparation of N-protected amidopyridinium salts were based on literature methods.[S1]

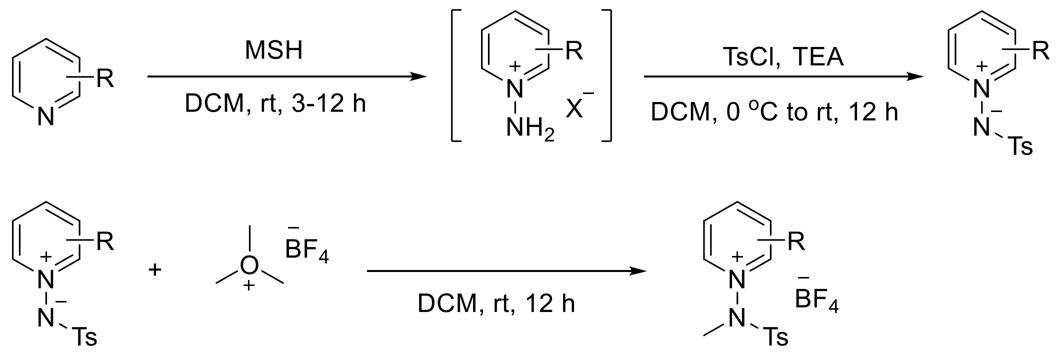

Supplementary Figure 4. General procedure 4 for Preparation of *N*-protected amidopyridinium salts

(1) Synthesis of *N*-amidopyridinium ylide from pyridines

Amination of pyridine was conducted using the previously developed method with hydroxylamine-*O*-sulfonic acid[S2] or *O*-mesitylsulfonylhydroxylamine (MSH)[S3]. A solution of ethyl-*O*-(mesitylsulfonyl)acetohydroxamate (MSA) (1.426 g, 5 mmol) in dioxane (5.0 mL) was added perchloric acid (70%, ~1.0 mL) at 0 °C until the mixture was solidified. The contents of the reaction were added to 50 mL of ice water and filtered. The filtrate was diluted with DCM and washed with water. The combined organic layers were dried over sodium sulfate, filtered, and used directly for the next step.[S4] To the crude mixture of 1-amidopyridinium (1.0 equiv.) in dichloromethane (DCM, 0.2 M) were added triethylamine (2.3 equiv.) and *p*-toluenesulfonyl chloride (TsCl, 1.0 equiv.) at 0 °C. The reaction mixture was stirred at room temperature for 12 h. The resulting mixture was diluted with DCM and washed with sat. $NaHCO_3$ solution and brine. The combined organic layers were dried over sodium sulfate, filtered, and concentrated in a vacuum. The resulting mixture was recrystallized with DCM/Et_2O to obtain *N*-protected 1-amidopyridinium ylides.

(2) Synthesis of *N*-protected amidopyridinium salts

To a solution of *N*-protected 1-amidopyridinium ylide (1.0 equiv.) in dichloromethane (0.3 M) were added trimethyloxonium tetrafluoroborate (meerwein's reagent, 1.1 equiv.) at room temperature. The mixture was stirred at room temperature for 12 h. The resulting mixture was concentrated under reduced pressure and recrystallized with DCM/Et_2O to obatain *N*-protected 1-amidopyridinium salts.

General procedure 5 for Preparation of [1.1.1]proepllane (GP5)

Preparation of [1.1.1]propellane were based on literature methods.[S4]

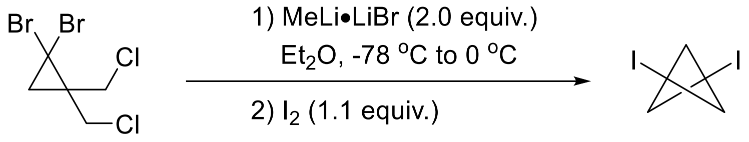

Supplementary Figure 5. General procedure 5 for Preparation of [1.1.1]proepllane

A solution of 1,1-dibromo-2,2-bis(chloromethyl)cyclopropane (11.2 g, 32.5 mmol, 1.0 equiv.) in diethylether (40 mL) under argon was cooled to -78 °C and treated with MeLi•LiBr by slow dropwise (50 mL, 75 mmol, 2.0 equiv., 1.5 M in diethylether) maintaining the temperature below -78 °C. The reaction was warmed to 0 °C and stirred at the same temperature 3 h. Iodine crystals (10.4 g, 41 mmol, 1.1 equiv.) were slowly added maintaining the temperature below 0 °C. After the addition was completed the reaction was slowly allowed to reach r.t. and stirred overnight. The mixture was diluted with Ethyl acetate and $Na_2S_2O_3$. The layer were separated and the organic layer was washed with brine, dired over with Na_2SO_4, and filtered. The filtrate was concentrated to a minimum amount of solvent and stored at -20 °C for recrystallization. Off white solid of 1,3-diiodobicyclo[1.1.1]pentane were formed are collected by filtration (6.5g, 21 mmol, 66%).

To a solution of NaCN (539 mg, 11 mmol, 2.2 equiv.) in DMSO (25 mL) at r.t. under an argon atmosphere in a flame dried 2-neck flask was added diiodobicyclo[1.1.1]pentane (1.6 g, 5 mmol, 1.0 equiv.) and the mixture was stirred for 1h. The flask was connected to a trap with pre-cooled flask to -78 °C and benezene (5 mL) was slowly added. Distillation of volatile material from 70 to 0 mbar was maintained for 10 min, while the reaction flask was kept at room temperature. After completion of the distillation approximate concentration of the solution (~1.0 M) was calculated using quantitative NMR with dichlromethane as internal standard. The solution was stored at -20 °C and its concentration did not change over a period of 1 month. If polymerization occurs rapidly, it is recommended to add hydroquinone (0.1 mg) as an additive to the distillation trap flask.

III. Optimization Table

Supplementary Table 1. Optimization of reaction conditions for Markovnikov hydropyridylation.[a]

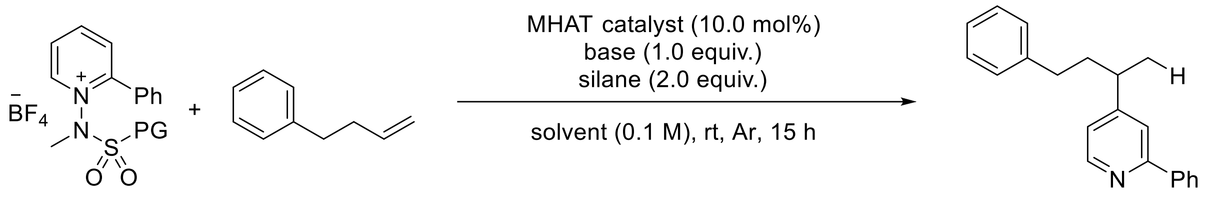

2 **5j** **6j**

Entry	Salt	MHAT catalyst	Silane	Base	Solvent	Yield (%)[b]
1	**2a**	$Fe(acac)_3$	$PhSiH_3$	NaOAc	EtOH	43
2	**2a**	$Fe(acac)_3$	$PhMeSiH_2$	NaOAc	EtOH	8
3	**2a**	$Fe(acac)_3$	PMHS	NaOAc	EtOH	8
4	**2a**	$Fe(acac)_3$	$Ph(O^iPr)SiH_2$	NaOAc	EtOH	53
5	**2b**	$Fe(acac)_3$	$Ph(O^iPr)SiH_2$	NaOAc	EtOH	38
6	**2c**	$Fe(acac)_3$	$Ph(O^iPr)SiH_2$	NaOAc	EtOH	37
7	**2d**	$Fe(acac)_3$	$Ph(O^iPr)SiH_2$	NaOAc	EtOH	52
8	**2e**	$Fe(acac)_3$	$Ph(O^iPr)SiH_2$	NaOAc	EtOH	39
9	**2a**	$Fe(acac)_3$	$Ph(O^iPr)SiH_2$	CsF	EtOH	4
10	**2a**	$Fe(acac)_3$	$Ph(O^iPr)SiH_2$	TEA	EtOH	20
11	**2a**	$Fe(acac)_3$	$Ph(O^iPr)SiH_2$	DBU	EtOH	9
12	**2a**	$Fe(acac)_3$	$Ph(O^iPr)SiH_2$	Na_2HPO_4	EtOH	46
**13**	**2a**	**$Fe(acac)_3$**	**$Ph(O^iPr)SiH_2$**	**$NaHCO_3$**	**EtOH**	**63**
14	**2a**	$Fe(acac)_3$	$Ph(O^iPr)SiH_2$	$CsHCO_3$	EtOH	41

[a]Reaction condition: **2** (0.1 mmol), **5j** (0.1 mmol), base (0.1 mmol), silane (0.2 mmol) and MHAT catalyst (10.0 mol%) in solvent (1.0 mL) under Ar at rt for 15 h. [b]Yields were determined by ^1H NMR with 1,1,2,2-tetrabromoethane.

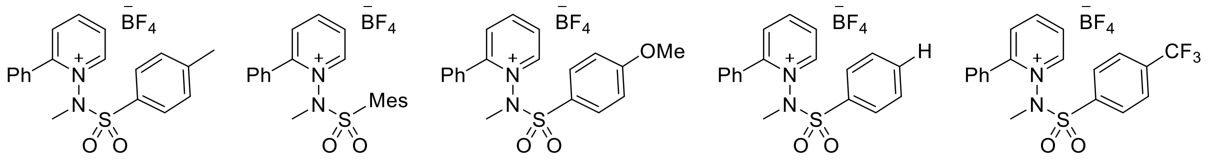

2a **2b** **2c** **2d** **2e**

Supplementary Table 2. Optimization of reaction conditions for Markovnikov hydropyridylation.[a]

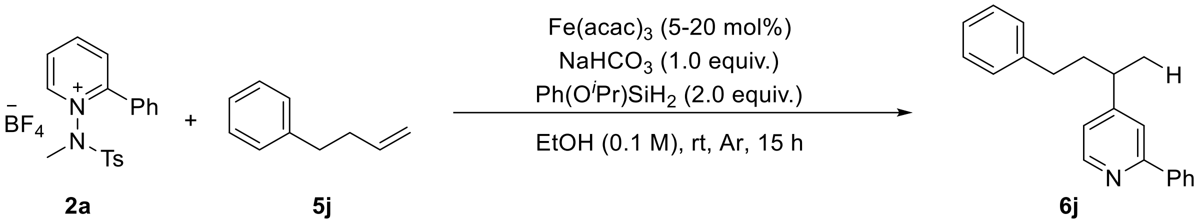

Entry	Fe(acac)3 (mol%)	Salt (equiv.)	Alkene (equiv.)	Yield (%)[b]
1	5	1.0	1.0	63
2	10	1.0	1.0	64
3	20	1.0	1.0	64
4	5	1.2	1.0	63
5	5	1.5	1.0	64
**6**	**5**	**1.0**	**1.5**	**71**
7	5	1.0	2.0	68

[a]Reaction condition: **2a** (0.1 mmol), **5j** (0.1 mmol), $NaHCO_3$ (0.1 mmol), $Ph(O^iPr)SiH_2$ (0.2 mmol) and $Fe(acac)_3$ (5.0 - 20.0 mol%) in solvent (1.0 mL) under Ar at rt for 15 h. [b]Yields were determined by ^1H NMR with 1,1,2,2-tetrabromoethane.

Supplementary Table 3. Optimization of reaction conditions for Markovnikov hydropyridylation.[a]

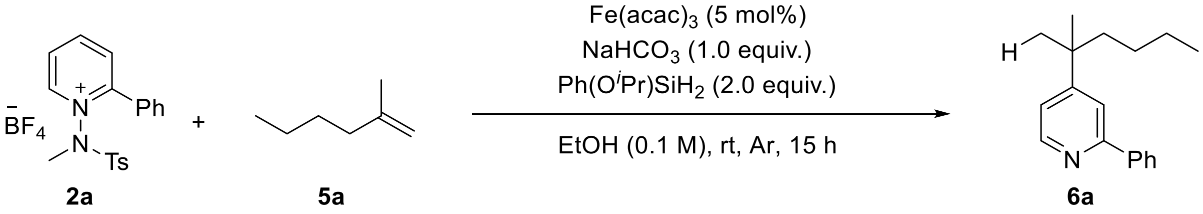

Entry	variation	Yield (%)[b]
1	No variation	77 (82)[c]
2	$Fe(dpm)_3$/ $Fe(dibm)_3$ instead of $Fe(acac)_3$	38 / 56
3	$Fe(acac)_2$/ $Fe(OTf)_2$ instead of $Fe(acac)_3$	55 / 6
4	$Co(acac)_2$/ Co(salen)/ $Mn(acac)_3$ instead of $Fe(acac)_3$	9 / trace / trace
5	Using 10 mol% of $Fe(acac)_3$	80
6	MeOH/ *i*PrOH instead of EtOH	70/ 35
7	$PhSiH_3$/ PhMeSiH2/ Ph3SiH/ (EtO)2MeSiH instead of $Ph(iOPr)SiH_2$	58/ 6/ trace/ 13
8	O_2/ Air condition instead of N_2 condition	trace/ 39

9	Without Fe catalyst	n.d.
10	Without silane	n.d.
11	Without base	43

[a]Reaction condition: **2a** (0.1 mmol), **5a** (0.15 mmol), $NaHCO_3$ (0.1 mmol), Ph(O^{i}Pr)SiH_2 (0.2 mmol) and $Fe(acac)_3$ (5.0 mol%) in EtOH (1.0 mL) under Ar at rt for 15 h. [b]Yields were determined by ^{1}H NMR with 1,1,2,2-tetrabromoethane. [c]Isolated yield.

Supplementary Table 4. Optimization of reaction conditions for 1,3-difunctionalization of [1.1.1]propellane.[a]

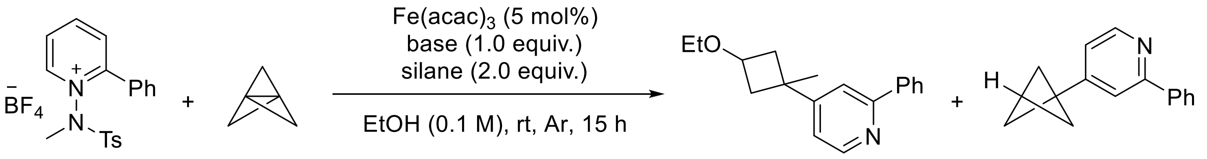

2a 1 3a 3aa

Entry	Fe catalyst	Catalyst loading	Silane	Base	Yield 3a (%)[b]	Yield 3aa (%)[b]
1	$Fe(acac)_3$	5	Ph(O^{i}Pr)SiH_2	$NaHCO_3$	73	trace
2	$Fe(dpm)_3$	5	Ph(O^{i}Pr)SiH_2	$NaHCO_3$	57	trace
3	Fe(TPP)Cl	5	Ph(O^{i}Pr)SiH_2	$NaHCO_3$	18	trace
4	$Fe(acac)_3$	10	Ph(O^{i}Pr)SiH_2	$NaHCO_3$	70	4
5	$Fe(acac)_3$	5	Ph(O^{i}Pr)SiH_2	NaF	67	trace
6	$Fe(acac)_3$	5	Ph(O^{i}Pr)SiH_2	NaOAc	8	32
7	$Fe(acac)_3$	5	Ph(O^{i}Pr)SiH_2	Na_2CO_3	63	trace
8	$Fe(acac)_3$	5	Ph(O^{i}Pr)SiH_2	-	46	trace
9	$Fe(acac)_3$	5	$PhSiH_3$	$NaHCO_3$	23	trace
10	$Fe(acac)_3$	5	$PhMeSiH_2$	$NaHCO_3$	4	trace
11	$Fe(acac)_3$	5	Ph_3SiH	$NaHCO_3$	Trace	trace
12	$Fe(acac)_3$	5	$(EtO)_2MeSiH$	$NaHCO_3$	12	trace

[a]Reaction condition: **2a** (0.1 mmol), **1** (0.15 mmol), base (0.1 mmol), Ph(O^{i}Pr)SiH_2 (0.2 mmol) and $Fe(acac)_3$ (5.0 mol%) in EtOH (1.0 mL) under N_2 at rt for 15 h. [b]Yields were determined by ^{1}H NMR with 1,1,2,2-tetrabromoethane.

Supplementary Figure 6. Representative substrate using 10 mol% of Fe catalyst

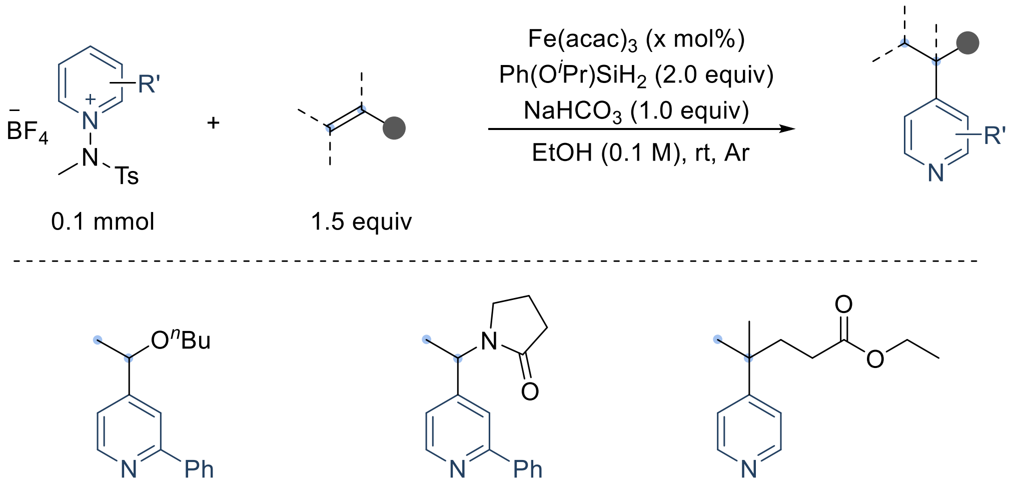

Fe 5 mol% : 46%
Fe 10 mol% : 72%

Fe 5 mol% : 42%
Fe 10 mol% : 58%

Fe 5 mol% : 59%
Fe 10 mol% : 75%

IV. Control Experiments

- Using EtOH as additive

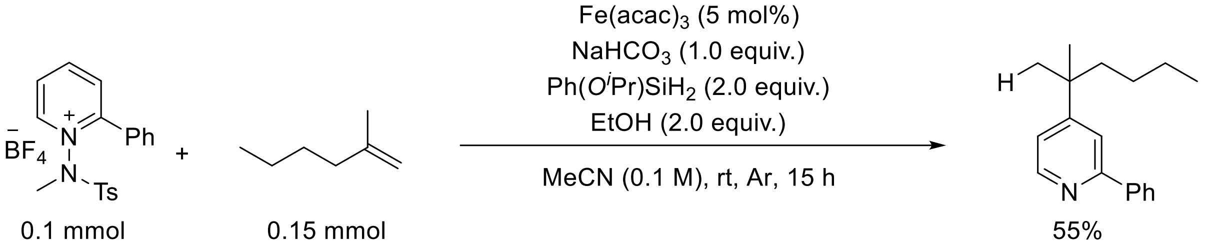

- Reaction with N–O salt

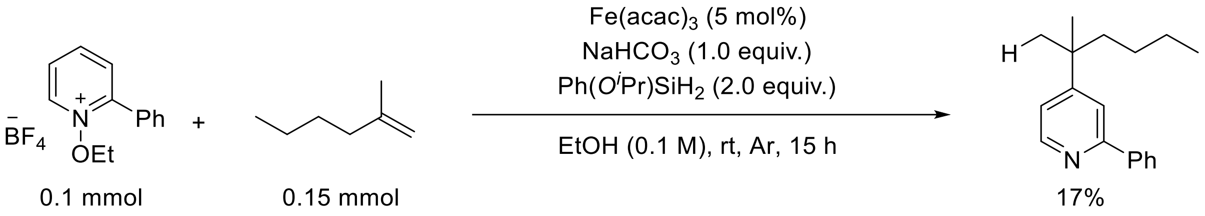

- Hydropyridylation reaction with C4-blocked *N*-amidopyridinium salt

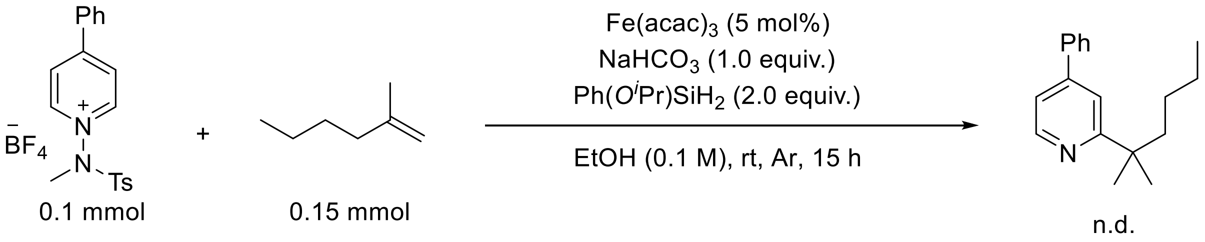

- 1,3-difunctionalization reaction with C4-blocked *N*-amidopyridinium salt

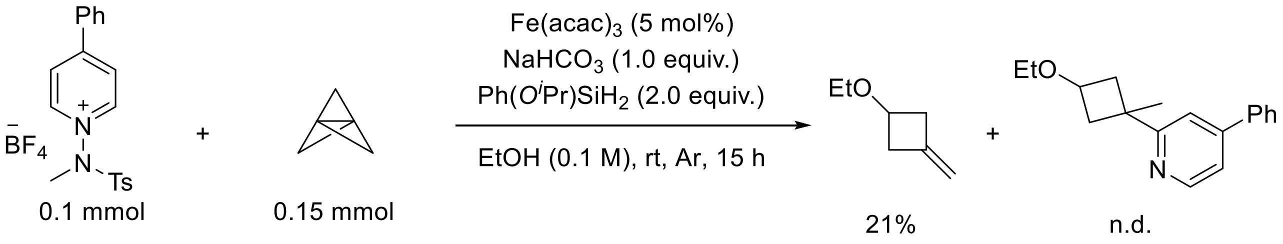

Supplementary Figure 7. Control experiments conducted in this study.

V. Mechanistic investigation

(a) Radical trapping experiment

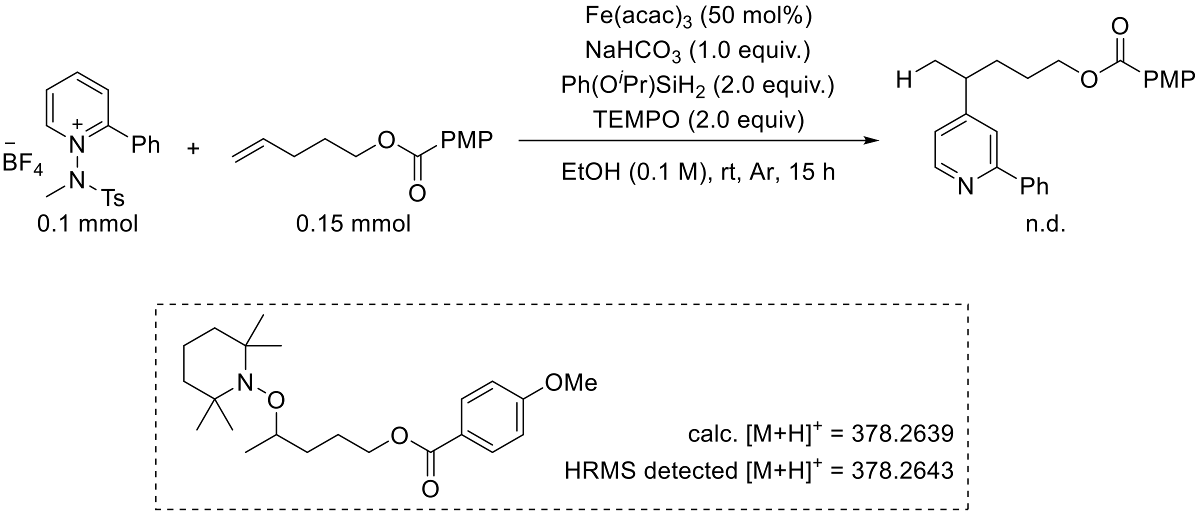

Supplementary Figure 8. Radical trapping experiment with TEMPO

(b) [1.1.1]propellane ring opening with additive

- with *N*-amidopyridinium salt 2a

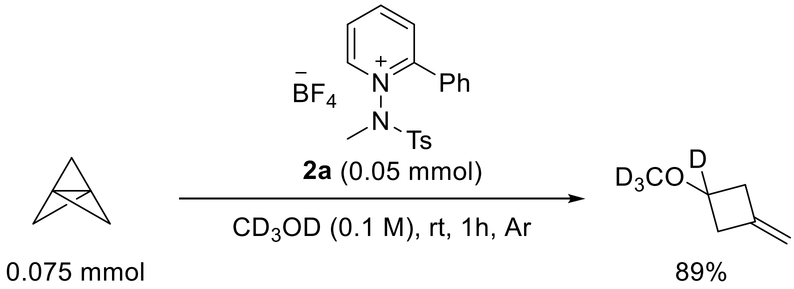

- with $Fe(acac)_3$

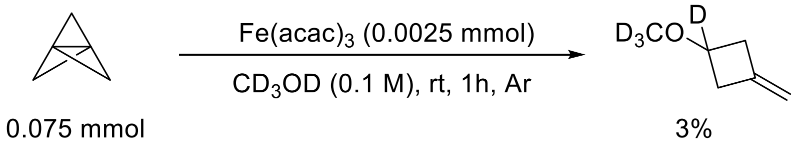

- with Fe(acac)3 + Ph(O^{i}Pr)SiH_2

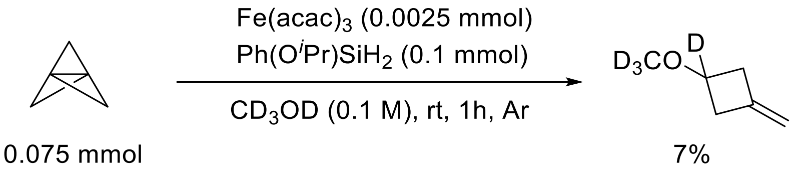

- with Fe(acac)$_2$

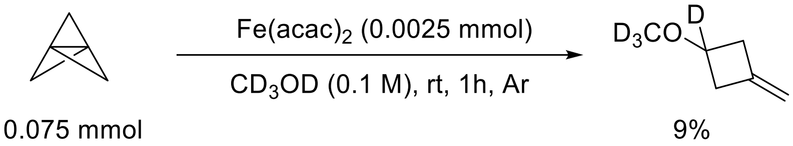

- with FeCl$_2$

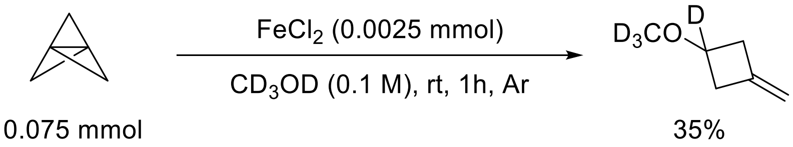

- with Ph(O^{i}Pr)SiH$_2$

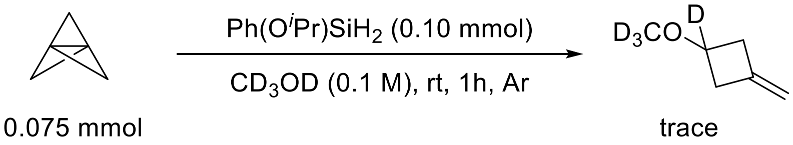

Supplementary Figure 9. [1.1.1]propellane ring opening with various additive.

(d) Control experiment of [1.1.1]propellane ring opening

- without *N*-amidopyridinium salt 2a

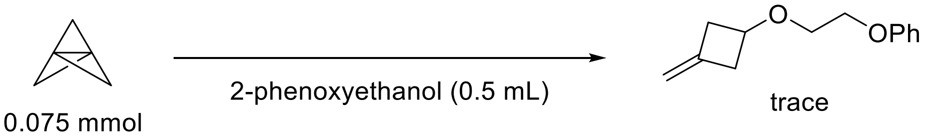

- with $NaBF_4$ (source of BF_4 anion)

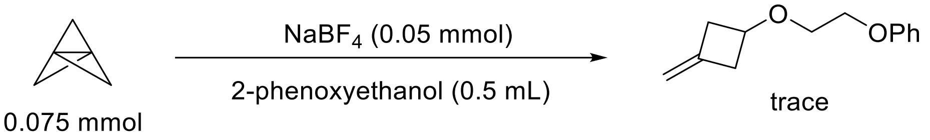

Supplementary Figure 10. Control experiment of [1.1.1]propellane ring opening

(e) Deuterium labeling experiment

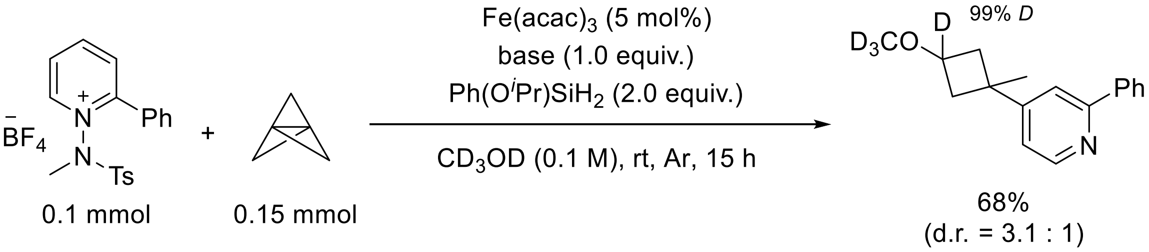

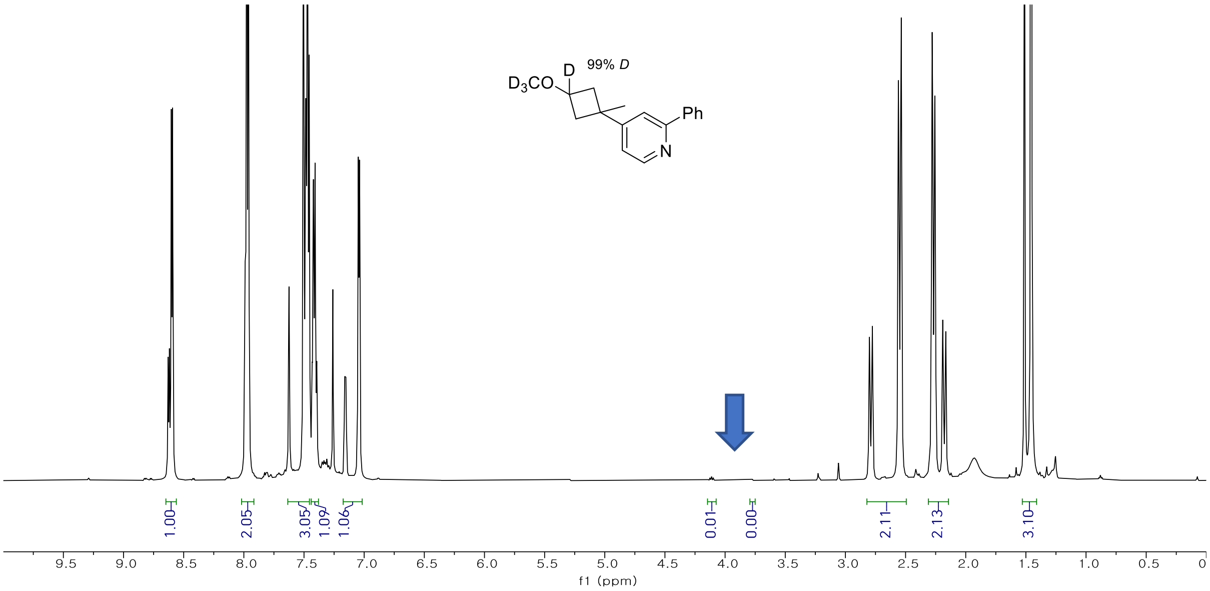

Supplementary Figure 11. ^1H NMR spectrum of the deuterium labeling experiment (Solvent : $CDCl_3$).

(f) NMR experiment with N-amidopyridinium salt & [1.1.1]propellane

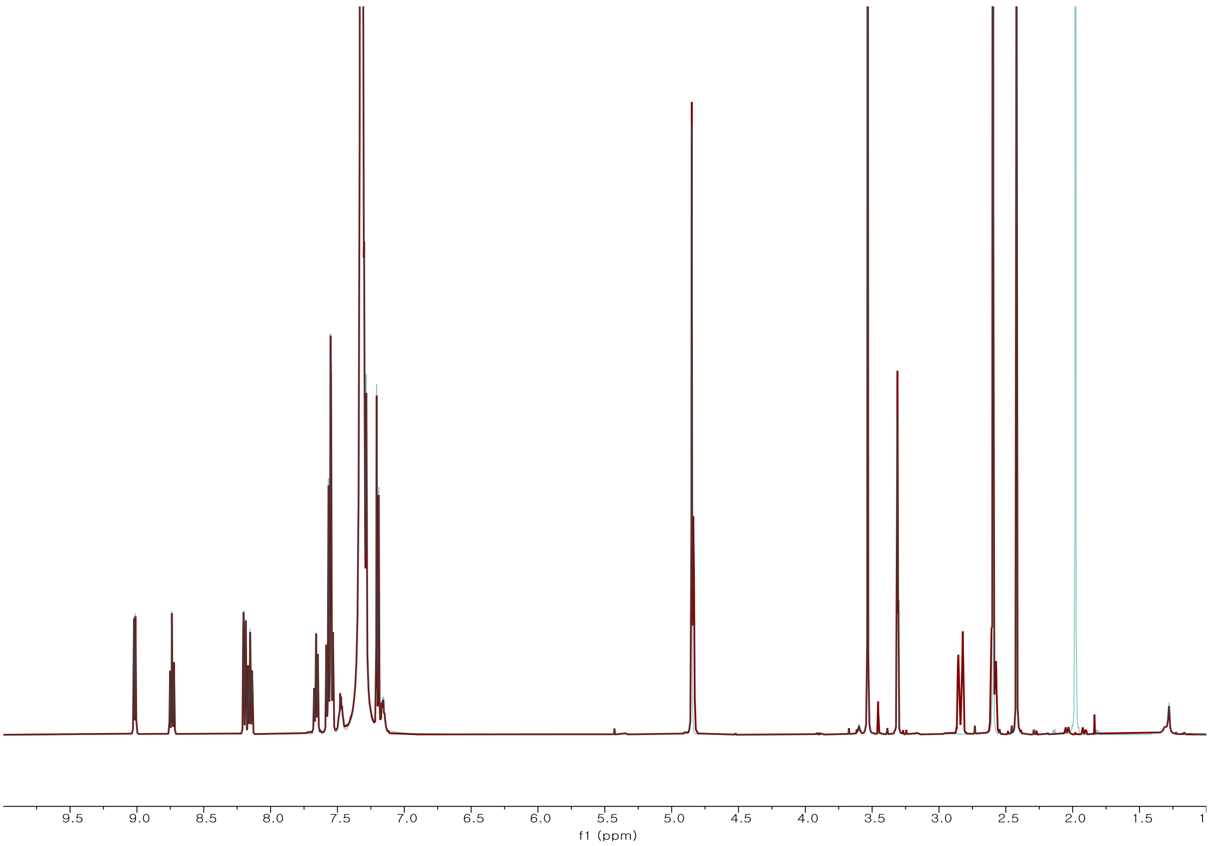

Supplementary Figure 12. ^{1}H NMR spectrum of [1.1.1]propellane and N-amidopyridinium salt **2a** mixture in MeOD solvent (red line : after 2 h, green line : after 0 h)

After 2 hours, when [1.1.1]propellane was completely consumed, there was no change in the NMR shift of the *N*-amidopyridinium salt **2a** from the mixture with [1.1.1]propellane (at 0 h). It may indicate the absence of direct interaction between the *N*-amidopyridinium salt and [1.1.1]propellane.

VI. Determination of major diastereomer of 1,3-difunctionalization of [1.1.1]propellane

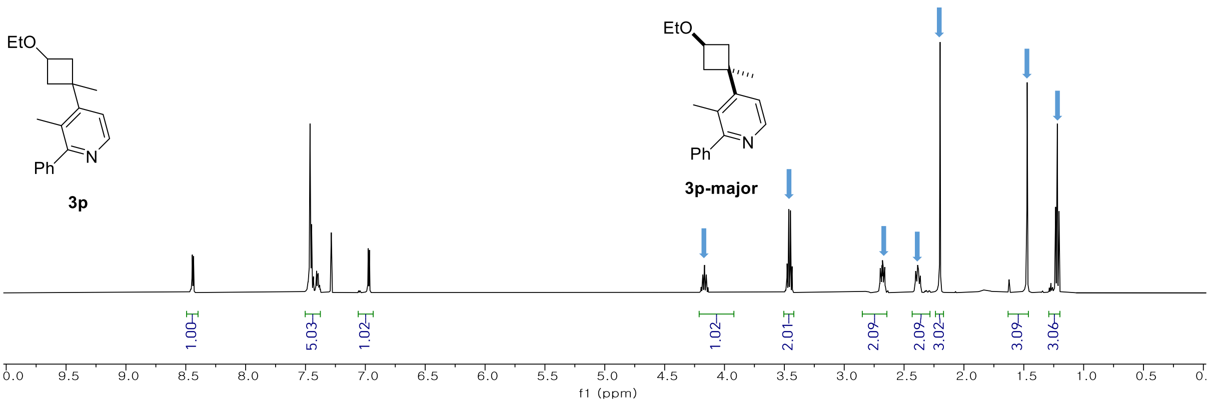

Supplementary Figure 13. ^1H NMR spectrum of product **3p** (Solvent : $CDCl_3$).

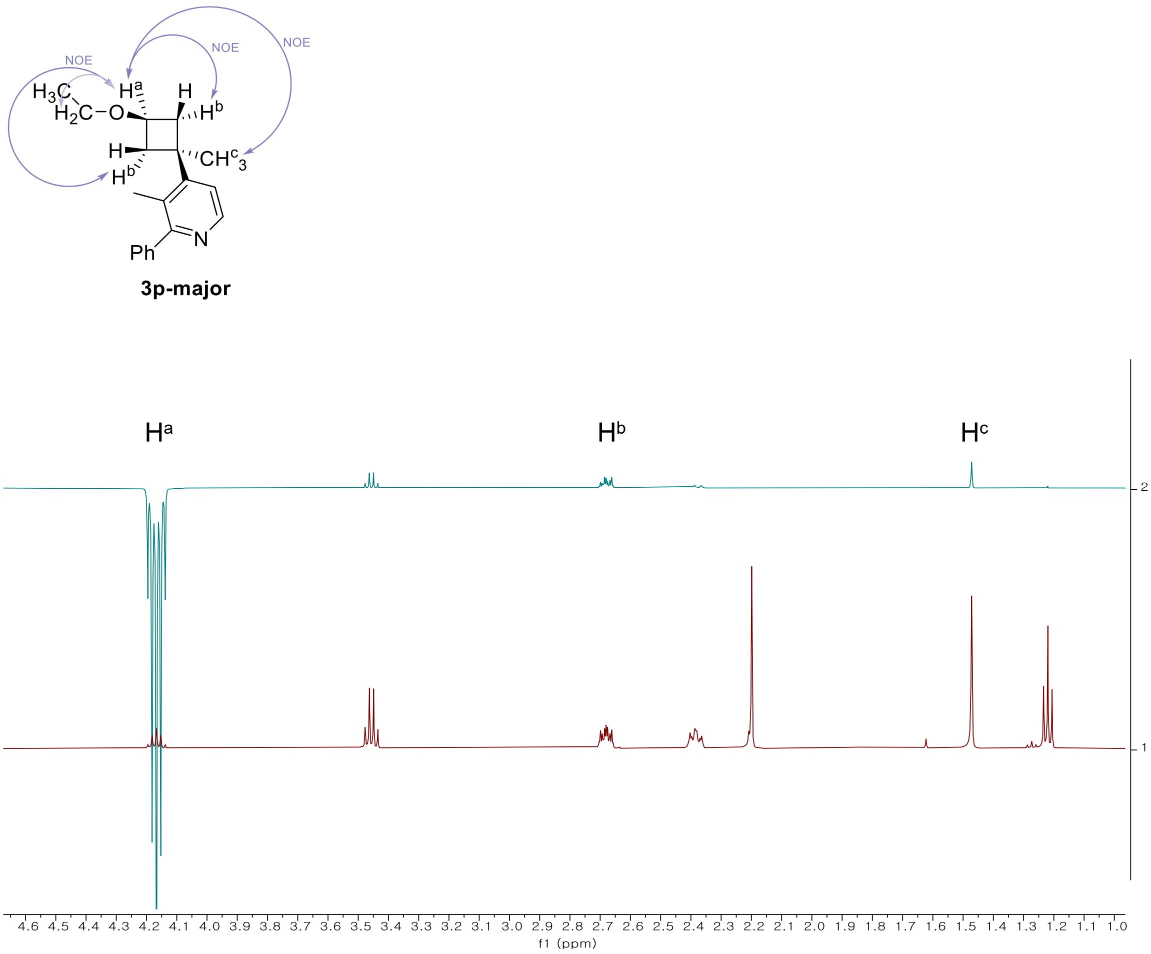

Supplementary Figure 14. NOE spectrum of product **3p** (Solvent : $CDCl_3$).

VII. Proposed mechanism

a) Part A : [1.1.1]propellane ring opening and nucleophilic addition

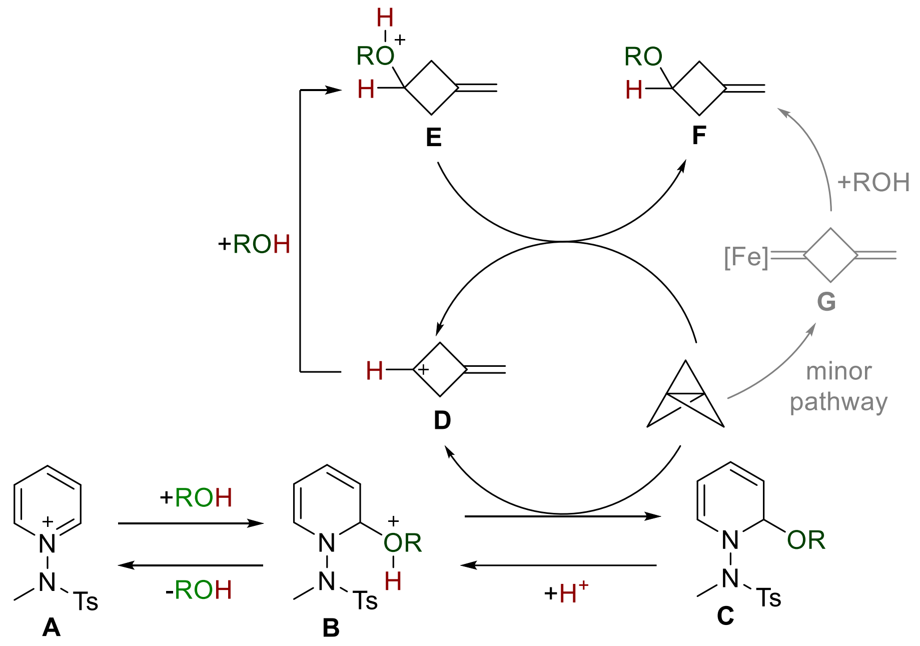

b) Part B : C4-selective iron-catalyzed hydropyridylation

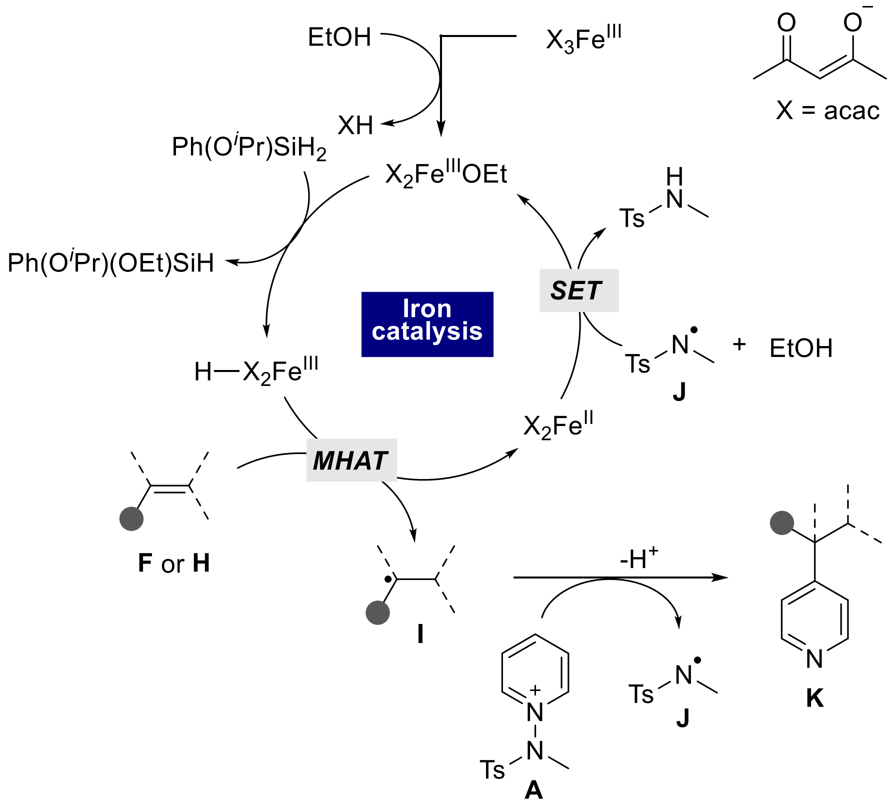

Supplementary Figure 15. Proposed mechanism of part A and part B.

VIII. Compound Characterizations

Characterization of hydropyridylat products obtained in this study

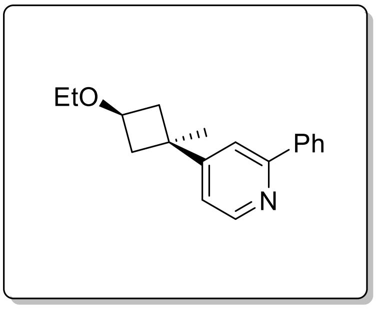

4-((1s,3s)-3-ethoxy-1-methylcyclobutyl)-2-phenylpyridine (3a). Mixture product. Yield 73% (19.5 mg) for 0.1 mmol scale. Yield 71% (189.1 mg) for 1.0 mmol scale. Colourless oil. ^{1}H NMR (500 MHz, $CDCl_3$) δ 8.59 (dd, *J* = 5.2, 0.8 Hz, 1H), 8.02 – 7.82 (m, 2H), 7.50 (dd, *J* = 1.8, 0.8 Hz, 1H), 7.50 – 7.45 (m, 2H), 7.44 – 7.38 (m, 1H), 7.04 (dd, *J* = 5.1, 1.7 Hz, 1H), 4.20 (p, *J* = 7.2 Hz, 1H), 3.42 (q, *J* = 7.0 Hz, 2H), 2.56 (ddt, *J* = 11.5, 6.9, 2.1 Hz, 2H), 2.35 – 2.25 (m, 2H), 1.46 (s, 3H), 1.19 (t, *J* = 7.0 Hz, 3H).; ^{13}C NMR (125 MHz, $CDCl_3$) δ 161.3, 159.7, 157.8, 157.8, 149.9, 149.8, 139.9, 139.9, 129.0, 128.9, 128.9, 128.8, 127.2, 127.1, 119.8, 119.0, 118.3, 117.4, 68.9, 68.5, 63.5, 63.5, 42.5, 42.0, 37.0, 35.0, 32.7, 30.2, 15.5, 15.5.; HRMS (ESI) m/z calcd. for $[C_{18}H_{22}NO]^+$: 268.1696, found: 268.1701

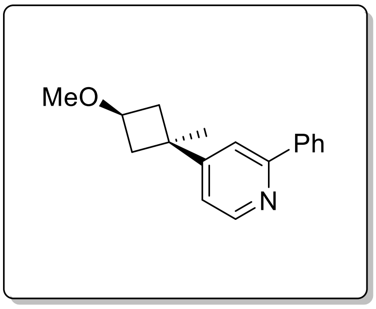

4-((1s,3s)-3-methoxy-1-methylcyclobutyl)-2-phenylpyridine (3b). Mixture product. Yield 69% (17.4 mg). Colourless oil. ^{1}H NMR (400 MHz, $CDCl_3$) δ 8.60 (dd, *J* = 5.1, 0.8 Hz, 1H), 8.02 – 7.91 (m, 2H), 7.52 – 7.49 (m, 1H), 7.50 – 7.44 (m, 2H), 7.44 – 7.37 (m, 1H), 7.04 (dd, *J* = 5.1, 1.7 Hz, 1H), 4.11 (p, *J* = 7.1 Hz, 1H), 3.26 (s, 3H), 2.56 (ddd, *J* = 9.5, 6.9, 2.7 Hz, 2H), 2.28 (ddd, *J* = 9.7, 7.3, 2.8 Hz, 2H), 1.46 (s, 3H).; ^{13}C NMR (100 MHz, $CDCl_3$) δ 161.3, 159.7, 157.8, 157.8, 149.9, 149.8, 139.9, 139.9, 129.0, 129.0, 128.9, 128.8, 127.1, 119.7, 119.0, 118.2, 117.4, 70.5, 70.3, 55.6, 55.5, 42.0, 41.5, 37.0, 34.8, 32.7, 30.2.; HRMS (ESI) m/z calcd. for $[C_{15}H_{18}NO]^+$: 254.1539, found: 254.1546

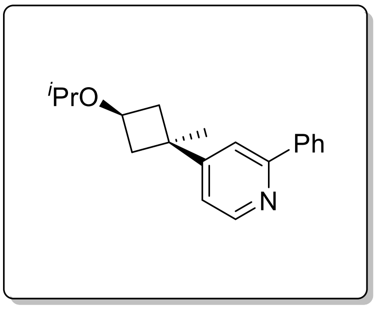

4-((1s,3s)-3-isopropoxy-1-methylcyclobutyl)-2-phenylpyridine (3c). Mixture product. Using DCM : ^{i}PrOH = 1 : 1 cosolvent. Yield 61% (17.5 mg). Colourless oil. ^{1}H NMR (500 MHz, $CDCl_3$) δ 8.59 (d, *J* = 5.1 Hz, 1H), 8.02 – 7.87 (m, 2H), 7.50 (d, *J* = 1.7 Hz, 1H), 7.47 (dd, *J* = 8.2, 6.8 Hz, 2H), 7.44 – 7.36 (m, 1H), 7.04 (dd, *J* = 5.2, 1.7 Hz, 1H), 4.26 (p, *J* = 7.2 Hz, 1H), 3.62 (hept, *J* = 5.9 Hz, 1H), 2.55 (ddd, *J* = 9.4, 6.9, 2.9 Hz, 2H), 2.30 (td, *J* = 8.1, 2.8 Hz, 2H), 1.46 (s, 3H), 1.15 (d, *J* = 6.1 Hz, 6H).; ^{13}C NMR (100 MHz, $CDCl_3$) δ 161.4, 159.6, 157.8, 157.8, 149.9, 149.8, 139.9, 139.9, 129.0, 128.9, 128.9, 128.8, 127.2, 127.2, 119.9, 119.0, 118.4, 117.4, 70.1, 70.0, 67.1, 66.5, 43.6, 42.9, 36.9, 35.2, 32.7, 30.1, 22.8.; HRMS (ESI) m/z calcd. for $[C_{19}H_{24}NO]^+$: 282.1852, found: 282.1859

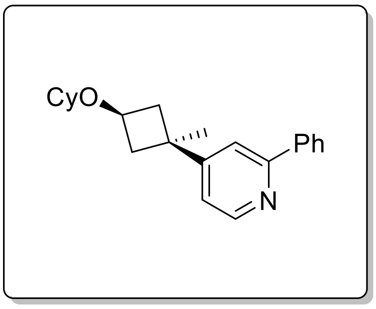

4-((1s,3s)-3-(cyclohexyloxy)-1-methylcyclobutyl)-2-phenylpyridine (3d). Mixture product. Using DCM : CyOH = 1 : 1 cosolvent. Yield 53% (17.1 mg). Colourless oil. ^{1}H NMR (500 MHz, $CDCl_3$) δ 8.59 (d, *J* = 5.1 Hz, 1H), 7.96 (dd, *J* = 8.5, 1.4 Hz, 2H), 7.49 (d, *J* = 1.7 Hz, 1H), 7.47 (dd, *J* = 8.2, 6.8 Hz, 2H), 7.44 – 7.38 (m, 1H), 7.03 (dd, *J* = 5.1, 1.7 Hz, 1H), 4.30 (p, *J* = 7.2 Hz, 1H), 3.27 (dp, *J* = 9.1, 4.1 Hz, 1H), 2.55 (ddd, *J* = 9.4, 6.9, 2.8 Hz, 2H), 2.37 – 2.26 (m, 2H), 1.87 (td, *J* = 10.5, 5.6 Hz, 2H), 1.73 (dt, *J* = 9.6, 4.8 Hz, 2H), 1.57 – 1.50 (m, 1H), 1.45 (s, 3H), 1.32 – 1.11 (m, 5H).; ^{13}C NMR (100 MHz, $CDCl_3$) δ 161.5, 159.6, 157.8, 157.7, 149.9, 149.8, 139.9, 139.9, 129.0, 128.9, 128.9, 128.8, 127.2, 127.1, 119.9, 119.0, 118.4, 117.4, 76.5, 76.4, 67.1, 66.4, 43.7, 43.0, 36.9, 35.2, 33.1, 33.0, 32.6, 30.1, 25.9, 24.5, 24.4.; HRMS (ESI) m/z calcd. for $[C_{22}H_{28}NO]^+$: 322.2165, found: 322.2172

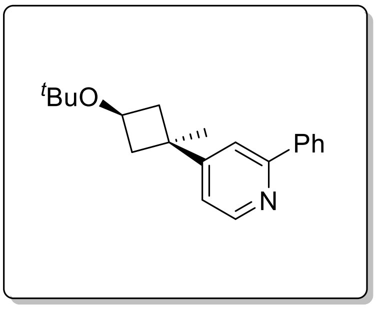

4-((1s,3s)-3-(tert-butoxy)-1-methylcyclobutyl)-2-phenylpyridine (3e). Mixture product. Using DCM : ^{t}BuOH = 1 : 1 cosolvent. Yield 47% (13.8 mg). Colourless oil. ^{1}H NMR (500 MHz, $CDCl_3$) δ 8.64 (d, *J* = 5.2 Hz, 1H), 8.58 (d, *J* = 5.1 Hz, 3H), 8.02 – 7.90 (m, 9H), 7.67 (d, *J* = 1.9 Hz, 1H), 7.52 – 7.45 (m, 13H), 7.44 – 7.37 (m, 5H), 7.20 (dd, *J* = 5.2, 1.8 Hz, 1H), 7.03 (dd, *J* = 5.1, 1.7 Hz, 3H), 4.36 (p, *J* = 7.3 Hz, 4H), 3.93 (p, *J* = 7.5 Hz, 1H), 2.81 (ddd, *J* = 9.8, 7.3, 2.8 Hz, 2H), 2.53 (ddd, *J* = 9.4, 6.9, 2.8 Hz, 7H), 2.32 (td, *J* = 8.2, 2.8 Hz, 7H), 2.25 – 2.17 (m, 2H), 1.46 (d, *J* = 1.9 Hz, 14H), 1.20 (s, 32H), 1.15 (s, 9H).; ^{13}C NMR (125 MHz, $CDCl_3$) δ 161.5, 159.6, 157.9, 157.7, 149.9, 149.8, 140.0, 139.9, 129.0, 128.9, 128.9, 128.8, 127.2, 127.1, 120.0, 119.1, 118.5, 117.5, 73.9, 73.8, 62.4, 61.6, 45.6, 44.7, 36.7, 35.4, 32.8, 30.1, 28.5.; HRMS (ESI) m/z calcd. for $[C_{20}H_{26}NO]^+$: 296.2009, found: 296.2015

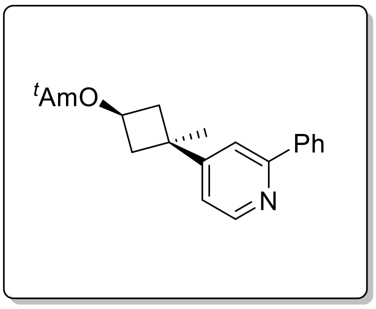

4-((1s,3s)-1-methyl-3-(tert-pentyloxy)cyclobutyl)-2-phenylpyridine (3f). Mixture product. Using DCM : ^{t}AmOH = 1 : 1 cosolvent. Yield 43% (14.6 mg). Colourless oil. ^{1}H NMR (500 MHz, $CDCl_3$) δ 8.58 (dd, *J* = 5.1, 0.8 Hz, 1H), 7.96 (dd, *J* = 8.3, 1.3 Hz, 2H), 7.51 – 7.44 (m, 3H), 7.41 (qt, *J* = 6.3, 1.3 Hz, 1H), 7.02 (dd, *J* = 5.1, 1.7 Hz, 1H), 4.32 (p, *J* = 7.3 Hz, 1H), 2.51 (ddd, *J* = 9.4, 6.9, 2.8 Hz, 2H), 2.31 (td, *J* = 8.2, 2.8 Hz, 2H), 1.53 – 1.39 (m, 5H), 1.13 (s, 6H), 0.91 – 0.83 (m, 3H).; ^{13}C NMR (100 MHz, $CDCl_3$) δ 161.6, 159.6, 157.8, 157.7, 149.9, 149.7, 140.0, 139.9, 129.0, 128.9, 128.9, 128.8, 127.2, 127.1, 120.0, 119.1, 118.5, 117.5, 76.0, 76.0, 62.0, 61.2, 45.6, 44.7, 36.7, 35.4, 33.6, 33.5, 32.8, 30.1, 26.0, 8.5.; HRMS (ESI) m/z calcd. for $[C_{21}H_{28}NO]^+$: 310.2165, found: 310.2173

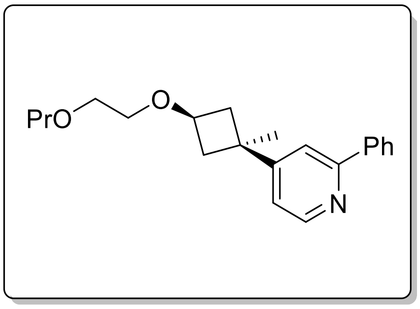

4-((1s,3s)-1-methyl-3-(2-propoxyethoxy)cyclobutyl)-2-phenylpyridine (3g). Mixture product. Using DCM : 2-propoxyethanol = 1 : 1 cosolvent. Using $Fe(acac)_3$ 10 mol%. Yield 48% (15.6 mg). Colourless oil. ^{1}H NMR (500 MHz, $CDCl_3$) δ 8.59 (dd, *J* = 5.2, 0.8 Hz, 1H), 8.00 – 7.89 (m, 2H), 7.50 – 7.49 (m, 1H), 7.49 – 7.45 (m, 2H), 7.44 – 7.37 (m, 1H), 7.04 (dd, *J* = 5.1, 1.7 Hz, 1H), 4.25 (p, *J* = 7.1 Hz, 1H), 3.61 – 3.48 (m, 4H), 3.41 (t, *J* = 6.9 Hz, 2H), 2.55 (ddt, *J* = 9.1, 7.0, 2.4 Hz, 2H), 2.33 (ddd, *J* = 11.9, 6.3, 2.8 Hz, 2H), 1.60 (h, *J* = 7.1 Hz, 2H), 1.45 (s, 3H), 0.90 (t, *J* = 7.4 Hz, 3H).; ^{13}C NMR (100 MHz, $CDCl_3$) δ 161.3, 159.7, 157.8, 157.8, 149.9, 149.8, 139.9, 139.9, 129.0, 128.9, 128.9, 128.8, 127.2, 127.1, 119.8, 119.0, 118.2, 117.4, 73.3, 73.3, 70.1, 69.6, 69.2, 67.6, 67.5, 42.4, 41.8, 36.9, 34.9, 32.6, 30.2, 22.9, 22.9, 10.6, 10.6.; HRMS (ESI) m/z calcd. for $[C_{21}H_{28}NO_2]^+$: 326.2115, found: 326.2121

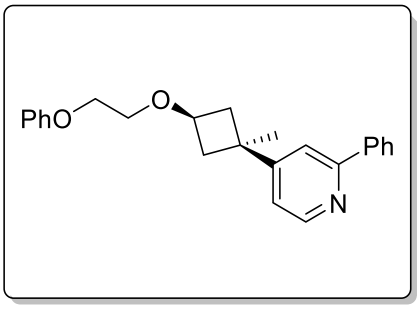

4-((1s,3s)-3-(cyclohexyloxy)-1-methylcyclobutyl)-2-phenylpyridine (3h). Mixture product. Using DCM : 2-phenoxyethanol = 1 : 1 cosolvent. Using $Fe(acac)_3$ 10 mol%. Using 3.0 equivalent of [1.1.1]propellane. Yield 50% (17.8 mg). Colourless oil. ^{1}H NMR (500 MHz, CD_2Cl_2) δ 8.57 (d, *J* = 5.1 Hz, 1H), 8.04 – 7.93 (m, 2H), 7.56 (d, *J* = 1.8 Hz, 1H), 7.47 (dd, *J* = 8.1, 6.4 Hz, 2H), 7.44 – 7.38 (m, 1H), 7.32 – 7.23 (m, 2H), 7.06 (dd, *J* = 5.1, 1.8 Hz, 1H), 6.99 – 6.84 (m, 3H), 4.30 (p, *J* = 7.1 Hz, 1H), 4.14 – 4.04 (m, 2H), 3.78 – 3.63 (m, 2H), 2.60 (ddd, *J* = 9.4, 6.9, 2.8 Hz, 2H), 2.39 – 2.24 (m, 2H), 1.47 (s, 3H).; ^{13}C NMR (100 MHz, CD_2Cl_2) δ 161.6, 160.0, 159.2, 159.2, 157.7, 157.6, 150.1, 150.0, 140.1, 129.8, 129.8, 129.2, 129.1, 129.0, 129.0, 121.2, 120.1, 119.3, 118.3, 117.4, 114.8, 70.0, 69.6, 67.7, 67.7, 67.0, 67.0, 42.5, 42.1, 37.3, 35.2, 32.6, 30.2.; HRMS (ESI) m/z calcd. for $[C_{24}H_{26}NO_2]^+$: 360.1958, found: 360.1963

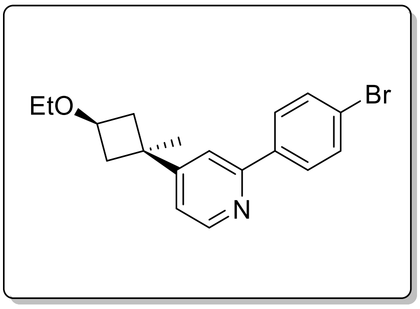

2-(4-bromophenyl)-4-((1s,3s)-3-ethoxy-1-methylcyclobutyl)pyridine (3i). Mixture product. Yield 66% (22.8 mg). Colourless oil. ^{1}H NMR (500 MHz, $CDCl_3$) δ 8.57 (d, *J* = 5.1 Hz, 1H), 7.85 (d, *J* = 8.6 Hz, 2H), 7.62 – 7.45 (m, 3H), 7.05 (dd, *J* = 5.1, 1.7 Hz, 1H), 4.19 (p, *J* = 7.2 Hz, 1H), 3.42 (q, *J* = 7.0 Hz, 2H), 2.62 – 2.49 (m, 2H), 2.33 – 2.24 (m, 2H), 1.45 (s, 3H), 1.19 (t, *J* = 7.0 Hz, 3H).; ^{13}C NMR (100 MHz, $CDCl_3$) δ 161.5, 159.9, 156.6, 156.5, 149.9, 149.9, 138.7, 138.7, 132.0, 131.9, 128.7, 128.7, 123.5, 123.4, 120.1, 119.3, 117.9, 117.1, 68.9, 68.5, 63.6, 63.5, 42.5, 42.0, 37.0, 35.0, 32.7, 30.2, 15.5, 15.5.; HRMS (ESI) m/z calcd. for $[C_{18}H_{21}BrNO]^+$: 346.0801, found: 346.0808

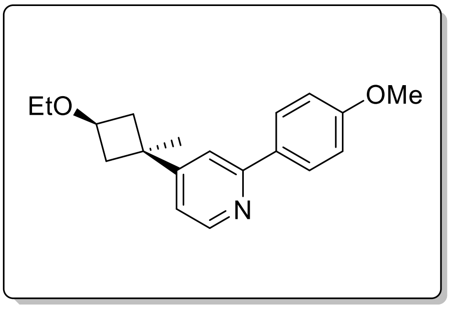

4-((1s,3s)-3-ethoxy-1-methylcyclobutyl)-2-(4-methoxyphenyl)pyridine (3j). Mixture product. Yield 62% (18.5 mg). Colourless oil. ^{1}H NMR (400 MHz, $CDCl_3$) δ 8.55 (dd, *J* = 5.1, 0.7 Hz, 1H), 7.94 (dd, *J* = 8.7, 6.3 Hz, 2H), 7.44 (dd, *J* = 1.8, 0.8 Hz, 1H), 7.04 – 6.93 (m, 3H), 4.19 (p, *J* = 7.2 Hz, 1H), 3.86 (s, 3H), 3.42 (q, *J* = 7.1 Hz, 2H), 2.54 (ddd, *J* = 9.4, 6.9, 2.7 Hz, 2H), 2.35 – 2.24 (m, 2H), 1.45 (s, 3H), 1.19 (t, *J* = 7.0 Hz, 3H).; ^{13}C NMR (100 MHz, $CDCl_3$) δ 161.2, 160.5, 160.5, 159.5, 157.4, 157.4, 149.7, 149.7, 132.5, 132.5, 128.4, 128.4, 119.2, 118.4, 117.4, 116.6, 114.2, 114.2, 68.9, 68.5, 63.5, 63.5, 55.5, 42.5, 42.0, 37.0, 35.0, 32.7, 30.2, 15.5, 15.5.; HRMS (ESI) m/z calcd. for $[C_{19}H_{24}NO_2]^+$: 298.1802, found: 298.1808

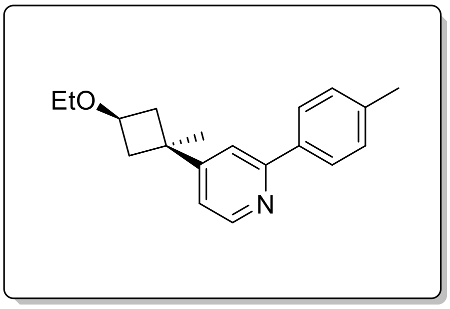

4-((1s,3s)-3-ethoxy-1-methylcyclobutyl)-2-(p-tolyl)pyridine (3k). Mixture product. Using $Fe(acac)_3$ 10 mol%. Yield 71% (19.9 mg). Colourless oil. ^{1}H NMR (400 MHz, CD_2Cl_2) δ 8.54 (d, *J* = 5.0 Hz, 1H), 7.95 – 7.85 (m, 3H), 7.52 (d, *J* = 1.8 Hz, 1H), 7.29 (dd, *J* = 8.3, 3.0 Hz, 3H), 7.03 (dd, *J* = 5.1, 1.7 Hz, 1H), 4.18 (p, *J* = 7.2 Hz, 1H), 3.40 (q, *J* = 7.0 Hz, 2H), 2.54 (ddt, *J* = 9.0, 6.9, 2.4 Hz, 2H), 2.41 (s, 5H), 2.25 (ddd, *J* = 9.6, 7.4, 2.8 Hz, 2H), 1.45 (s, 3H), 1.15 (t, *J* = 7.0 Hz, 3H).; ^{13}C NMR (100 MHz, CD_2Cl_2) δ 161.7, 160.0, 157.6, 157.6, 149.9, 149.9, 139.3, 139.3, 137.3, 129.7, 129.7, 127.1, 127.1, 119.9, 119.0, 117.9, 117.0, 69.1, 68.8, 63.6, 63.6, 42.7, 42.3, 37.3, 35.3, 32.7, 30.2, 21.4, 15.6.; HRMS (ESI) m/z calcd. for $[C_{19}H_{24}NO]^+$: 282.1852, found: 282.1859

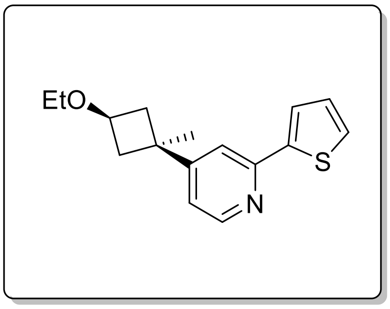

4-((1s,3s)-3-(cyclohexyloxy)-1-methylcyclobutyl)-2-phenylpyridine (3l). Mixture product. Using

Fe(acac)$_3$ 10 mol%. Yield 54% (14.8 mg). Colourless oil. ^{1}H NMR (500 MHz, CDCl$_3$) δ 8.47 (d, J = 5.2 Hz, 1H), 7.58 (d, J = 3.7 Hz, 1H), 7.43 (d, J = 1.7 Hz, 1H), 7.38 (d, J = 5.2 Hz, 1H), 7.11 (dd, J = 5.2, 3.7 Hz, 1H), 6.95 (dd, J = 5.2, 1.7 Hz, 1H), 4.19 (p, J = 7.1 Hz, 1H), 3.42 (q, J = 7.0 Hz, 2H), 2.54 (ddd, J = 9.4, 6.9, 2.8 Hz, 2H), 2.27 (ddd, J = 9.7, 7.5, 2.9 Hz, 2H), 1.44 (s, 3H), 1.19 (t, J = 7.0 Hz, 3H).; ^{13}C NMR (100 MHz, CDCl$_3$) δ 161.3, 159.7, 152.8, 152.7, 149.7, 149.7, 145.2, 128.1, 128.1, 127.6, 127.5, 124.5, 124.5, 119.8, 118.9, 116.3, 115.4, 68.9, 68.5, 63.6, 63.5, 42.5, 41.9, 36.9, 35.0, 32.6, 30.1, 15.5, 15.5.; HRMS (ESI) m/z calcd. for [$C_{16}H_{20}NOS$]$^+$: 274.1260, found: 274.1265

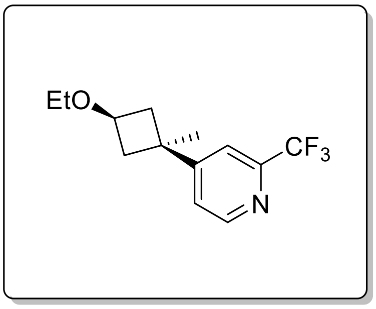

4-((1s,3s)-3-ethoxy-1-methylcyclobutyl)-2-(trifluoromethyl)pyridine (3m). Mixture product. Using Fe(acac)$_3$ 10 mol%. Yield 46% (11.8 mg). Colourless oil. ^{1}H NMR (500 MHz, CDCl$_3$) δ 8.62 (d, J = 5.1 Hz, 1H), 7.46 (d, J = 1.7 Hz, 1H), 7.28 (dd, J = 5.1, 1.7 Hz, 1H), 4.19 (p, J = 7.1 Hz, 1H), 3.41 (q, J = 7.1 Hz, 2H), 2.56 (ddt, J = 11.5, 6.9, 2.1 Hz, 2H), 2.29 – 2.19 (m, 2H), 1.44 (s, 3H), 1.18 (t, J = 7.1 Hz, 3H).; ^{13}C NMR (125 MHz, CDCl$_3$) δ 162.5, 161.2, 150.2, 150.2, 148.6 (q, J = 33.5, 32.8 Hz), 148.5 (q, J = 33.9 Hz), 123.9, 123.1, 121.8 (q, J = 274.0 Hz), 121.8 (q, J = 274.1 Hz), 118.0 (q, J = 2.9 Hz), 117.3 (q, J = 2.8 Hz), 68.7, 68.3, 63.6, 63.6, 42.4, 42.0, 37.3, 35.2, 32.4, 30.0, 15.5, 15.4.; HRMS (ESI) m/z calcd. for [$C_{13}H_{17}F_3NO$]$^+$: 260.1257, found: 260.1262

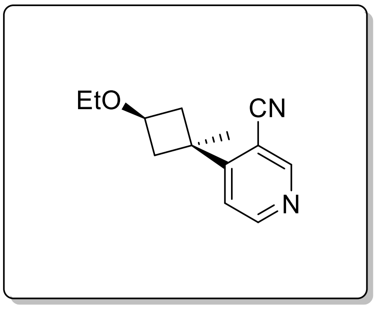

4-((1s,3s)-3-ethoxy-1-methylcyclobutyl)nicotinonitrile (3n). Mixture product. Using Fe(acac)$_3$ 10 mol%. Yield 53% (11.4 mg). Colourless oil. ^{1}H NMR (400 MHz, CDCl$_3$) δ 8.78 (s, 1H), 8.67 (d, J = 5.5 Hz, 1H), 7.17 (d, J = 5.2 Hz, 1H), 4.17 (p, J = 7.1 Hz, 1H), 3.42 (q, J = 7.0 Hz, 2H), 2.77 (ddd, J = 11.7, 6.0, 2.5 Hz, 2H), 2.33 (ddd, J = 12.2, 6.3, 2.7 Hz, 2H), 1.52 (s, 3H), 1.18 (t, J = 7.0 Hz, 3H).; ^{13}C NMR (100 MHz, CDCl$_3$) δ 164.2, 163.0, 154.4, 154.0, 153.0, 152.9, 121.0, 120.9, 116.7, 116.7, 107.4, 69.0, 68.5, 63.7, 63.6, 42.1, 41.4, 39.0, 35.5, 30.9, 28.9, 15.5, 15.4.; HRMS (ESI) m/z calcd. for [$C_{13}H_{17}N_2O$]$^+$: 217.1335, found: 217.1342

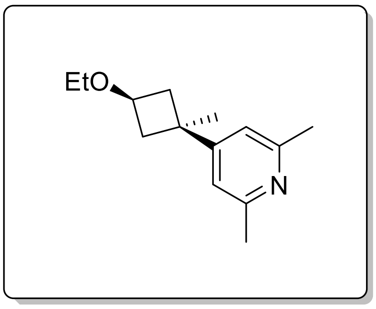

4-((1s,3s)-3-ethoxy-1-methylcyclobutyl)-2,6-dimethylpyridine (3o). Mixture product. Using $Fe(acac)_3$ 10 mol%. Yield 48% (10.5 mg). Colourless oil. ^{1}H NMR (400 MHz, $CDCl_3$) δ 6.75 (s, 2H), 4.14 (p, *J* = 7.2 Hz, 1H), 3.47 – 3.30 (m, 2H), 2.50 (s, 6H), 2.49 – 2.43 (m, 2H), 2.20 (ddt, *J* = 9.7, 7.5, 2.6 Hz, 2H), 1.37 (s, 3H), 1.18 (t, *J* = 7.0 Hz, 3H).; ^{13}C NMR (100 MHz, $CDCl_3$) δ 161.3, 159.5, 157.7, 157.7, 117.9, 117.0, 69.0, 68.5, 63.5, 63.4, 42.5, 41.9, 36.6, 34.7, 32.6, 30.1, 24.7, 24.6, 15.5, 15.5.; HRMS (ESI) m/z calcd. for $[C_{14}H_{22}NO]^+$: 220.1696, found: 220.1701

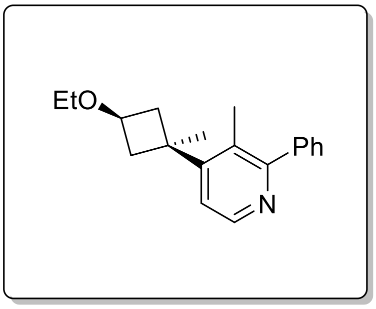

4-((1s,3s)-3-ethoxy-1-methylcyclobutyl)-3-methyl-2-phenylpyridine (3p). Mixture product. Using NaF as base. Yield 51% (14.4 mg). Colourless oil. ^{1}H NMR (400 MHz, $CDCl_3$) δ 8.42 (d, *J* = 5.1 Hz, 1H), 7.45 – 7.34 (m, 6H), 6.95 (d, *J* = 5.1 Hz, 1H), 4.14 (p, *J* = 7.3 Hz, 1H), 3.43 (q, *J* = 7.0 Hz, 2H), 2.65 (ddt, *J* = 9.2, 6.8, 2.5 Hz, 2H), 2.43 – 2.30 (m, 2H), 2.17 (s, 3H), 1.45 (s, 3H), 1.20 (t, *J* = 7.0 Hz, 3H).; ^{13}C NMR (100 MHz, $CDCl_3$) δ 160.2, 159.0, 146.8, 141.4, 129.2, 128.2, 127.8, 120.0, 68.7, 63.5, 43.2, 36.1, 28.3, 17.0, 15.5.; HRMS (ESI) m/z calcd. for $[C_{19}H_{24}NO]^+$: 282.1852, found: 282.1858

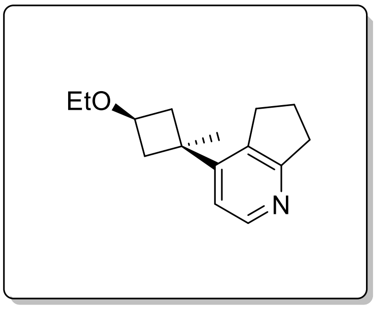

4-((1s,3s)-3-ethoxy-1-methylcyclobutyl)-6,7-dihydro-5H-cyclopenta[b]pyridine (3q). Mixture product. Yield 42% (9.7 mg). Colourless oil. ^{1}H NMR (400 MHz, $CDCl_3$) δ 8.25 (d, *J* = 5.3 Hz, 1H), 6.75 (d, *J* = 5.2 Hz, 1H), 4.13 (p, *J* = 7.2 Hz, 1H), 3.40 (q, *J* = 7.0 Hz, 2H), 2.97 (t, *J* = 7.7 Hz, 2H), 2.84 (t, *J* = 7.4 Hz, 2H), 2.52 (ddt, *J* = 9.2, 6.9, 2.5 Hz, 2H), 2.24 (td, *J* = 8.1, 2.9 Hz, 2H), 2.08 (p, *J* = 7.7 Hz, 2H), 1.35 (s, 3H), 1.17 (t, *J* = 7.0 Hz, 3H).; ^{13}C NMR (100 MHz, $CDCl_3$) δ 166.3, 166.0, 155.9,

154.5, 147.8, 134.3, 133.3, 118.0, 117.9, 69.3, 68.9, 63.4, 41.9, 41.0, 38.3, 35.1, 34.0, 34.0, 30.6, 30.0, 29.9, 28.1, 23.4, 23.1, 15.5, 15.5.; HRMS (ESI) m/z calcd. for $[C_{15}H_{22}NO]^+$: 232.1696, found: 232.1701

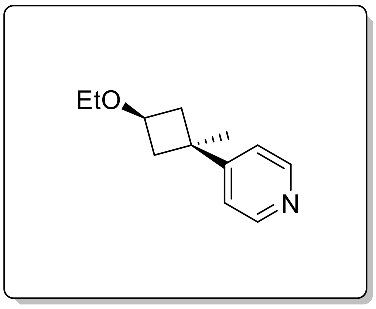

4-((1s,3s)-3-ethoxy-1-methylcyclobutyl)pyridine (3r). Mixture product. Using $Fe(acac)_3$ 10 mol%. Using 3.0 equivalent of [1.1.1]propellane. Yield 46% (8.7 mg). Colourless oil. ^{1}H NMR (400 MHz, $CDCl_3$) δ 8.55 (s, 2H), 7.12 (d, *J* = 5.4 Hz, 2H), 4.17 (p, *J* = 7.1 Hz, 1H), 3.41 (q, *J* = 7.0 Hz, 2H), 2.51 (ddt, *J* = 9.0, 6.9, 2.4 Hz, 2H), 2.24 (ddd, *J* = 9.7, 7.4, 2.8 Hz, 2H), 1.41 (s, 3H), 1.18 (t, *J* = 6.9 Hz, 3H).; ^{13}C NMR (100 MHz, $CDCl_3$) δ 161.0, 159.3, 149.6, 149.5, 120.7, 68.9, 68.4, 63.6, 63.5, 42.5, 42.0, 36.8, 34.9, 32.5, 30.0, 15.5, 15.5.; HRMS (ESI) m/z calcd. for $[C_{12}H_{18}NO]^+$: 192.1383, found: 192.1388

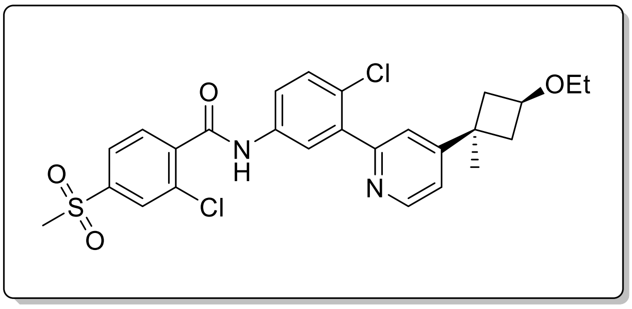

2-chloro-N-(4-chloro-3-(4-((1s,3s)-3-ethoxy-1-methylcyclobutyl)pyridin-2-yl)phenyl)-4-(methylsulfonyl)benzamide (3s). Mixture product. Using $Fe(acac)_3$ 10 mol%. Yield 61% (32.5 mg). White solid. ^{1}H NMR (500 MHz, CD_2Cl_2) δ 10.07 (s, 1H), 8.25 (d, *J* = 5.3 Hz, 1H), 8.05 (dt, *J* = 8.8, 2.1 Hz, 1H), 7.86 (d, *J* = 1.7 Hz, 1H), 7.74 – 7.62 (m, 2H), 7.53 (d, *J* = 8.0 Hz, 1H), 7.48 (d, *J* = 8.8 Hz, 1H), 7.44 (d, *J* = 1.8 Hz, 1H), 7.04 (dd, *J* = 5.3, 1.8 Hz, 1H), 4.15 (p, *J* = 7.1 Hz, 1H), 3.37 (q, *J* = 7.0 Hz, 2H), 2.96 (s, 3H), 2.51 (ddt, *J* = 9.0, 6.9, 2.0 Hz, 2H), 2.21 – 2.07 (m, 2H), 1.40 (s, 3H), 1.13 (t, *J* = 7.0 Hz, 3H).; ^{13}C NMR (100 MHz, CD_2Cl_2) δ 164.4, 162.4, 160.8, 155.8, 148.6, 148.5, 143.1, 141.1, 138.9, 138.7, 137.8, 137.8, 132.4, 131.2, 131.1, 130.1, 129.2, 127.4, 127.4, 126.2, 123.7, 123.0, 123.0, 122.9, 122.0, 121.9, 120.7, 120.1, 68.9, 68.6, 63.7, 63.6, 44.7, 42.6, 42.3, 37.5, 35.3, 32.3, 29.9, 15.6, 15.5.; HRMS (ESI) m/z calcd. for $[C_{26}H_{27}Cl_2N_2O_4S]^+$: 533.1063, found: 533.1068

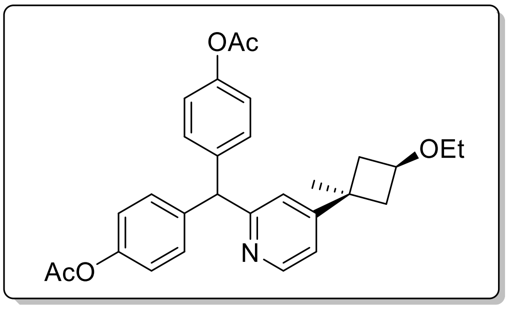

((4-((1s,3s)-3-ethoxy-1-methylcyclobutyl)pyridin-2-yl)methylene)bis(4,1-phenylene) diacetate (3t). Mixture product. Using Fe(acac)$_3$ 10 mol%. Yield 53% (25.1 mg). White solid. ^{1}H NMR (400 MHz, CDCl$_3$) δ 8.48 (d, J = 5.1 Hz, 1H), 7.18 (d, J = 8.8 Hz, 4H), 7.01 (d, J = 8.5 Hz, 4H), 6.95 (dd, J = 5.2, 1.7 Hz, 1H), 6.92 (s, 1H), 5.60 (s, 1H), 4.13 (p, J = 7.2 Hz, 1H), 3.39 (q, J = 7.1 Hz, 2H), 2.46 (ddd, J = 9.5, 6.8, 2.9 Hz, 2H), 2.28 (s, 6H), 2.16 (ddd, J = 12.2, 6.4, 2.7 Hz, 2H), 1.35 (s, 3H), 1.17 (t, J = 7.0 Hz, 3H).; ^{13}C NMR (100 MHz, CDCl$_3$) δ 169.6, 162.5, 162.5, 161.5, 159.8, 149.7, 149.6, 149.4, 149.4, 140.3, 140.2, 130.4, 130.4, 121.5, 121.5, 121.3, 120.4, 119.3, 118.6, 68.9, 68.4, 63.5, 58.2, 58.2, 42.5, 42.0, 36.8, 34.9, 32.5, 30.1, 21.3, 15.5, 15.4.; HRMS (ESI) m/z calcd. for $[C_{29}H_{32}NO_5]^+$: 474.2275, found: 474.2280

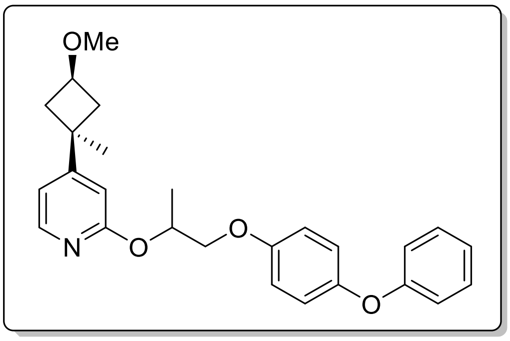

4-((1s,3s)-3-methoxy-1-methylcyclobutyl)-2-((1-(4-phenoxyphenoxy)propan-2-yl)oxy)pyridine (3u). Mixture product. Using Fe(acac)$_3$ 10 mol%. Using 3.0 equivalent of [1.1.1]propellane. Using NaF as base. Yield 59% (24.7 mg). Colourless oil. ^{1}H NMR (500 MHz, CDCl$_3$) δ 8.06 (d, J = 5.4 Hz, 1H), 7.31 – 7.27 (m, 2H), 7.07 – 7.00 (m, 1H), 6.98 – 6.90 (m, 6H), 6.69 (dd, J = 5.4, 1.6 Hz, 1H), 6.55 (d, J = 1.6 Hz, 1H), 5.64 – 5.51 (m, 1H), 4.22 – 4.15 (m, 1H), 4.13 – 4.02 (m, 2H), 3.23 (s, 3H), 2.62 – 2.38 (m, 2H), 2.20 (ddt, J = 9.6, 7.5, 2.2 Hz, 2H), 1.49 (t, J = 6.3 Hz, 3Hz), 1.39 (s, 3H).; ^{13}C NMR (100 MHz, CDCl$_3$) δ 163.7, 163.6, 163.6, 162.0, 158.6, 158.6, 155.4, 155.4, 150.4, 150.4, 146.8, 146.8, 129.7, 122.6, 122.5, 120.9, 117.7, 115.9, 115.1, 114.3, 108.5, 107.8, 71.2, 70.5, 70.2, 69.4, 69.4, 55.6, 55.4, 41.9 (d, J = 1.5 Hz), 41.4 (d, J = 1.4 Hz), 36.6, 34.6, 32.3, 29.9, 17.2.; HRMS (ESI) m/z calcd. for $[C_{26}H_{30}NO_4]^+$: 420.2169, found: 420.2175

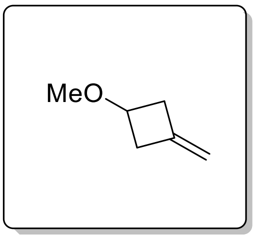

1-methoxy-3-methylenecyclobutane (4a). Yield 83%. ^{1}H NMR (500 MHz, $CDCl_3$, crude) δ 4.82 (p, *J* = 2.3 Hz, 2H), 3.90 (p, *J* = 6.5 Hz, 1H), 3.22 (s, 3H), 2.65 – 2.59 (m, 2H). Other peaks overlap with the solvent and are difficult to confirm.

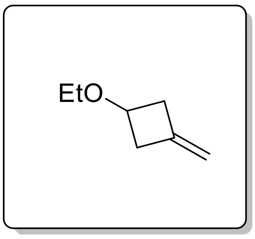

1-ethoxy-3-methylenecyclobutane (4b). Yield 62%. ^{1}H NMR (500 MHz, $CDCl_3$, crude) δ 4.81 (tt, *J* = 2.9, 1.9 Hz, 2H), 3.97 (p, *J* = 6.7 Hz, 1H), 3.39 (q, *J* = 7.1 Hz, 2H), 2.91 – 2.77 (m, 2H), 2.69 – 2.61 (m, 2H). Other peaks overlap with the solvent and are difficult to confirm.

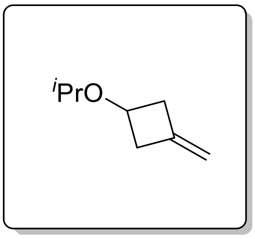

1-isopropoxy-3-methylenecyclobutane (4c). Using 1.0 equivalent of *N*-amidopyridinium salt. Using DCM : ^{i}PrOH = 1 : 1 cosolvent. Yield 66%. ^{1}H NMR (500 MHz, $CDCl_3$, crude) δ 4.81 (tt, *J* = 2.9, 1.9 Hz, 2H), 2.90 – 2.82 (m, 2H), 2.70 – 2.62 (m, 2H). Other peaks overlap with the solvent and are difficult to confirm.

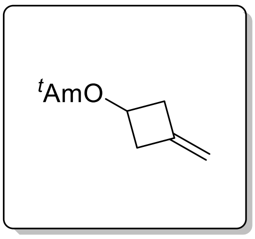

1-methylene-3-(tert-pentyloxy)cyclobutane (4d). Yield 40%. Using 1.0 equivalent of *N*-amidopyridinium salt. Using DCM : ^{t}AmOH = 1 : 1 cosolvent. ^{1}H NMR (500 MHz, $CDCl_3$, crude) δ 4.81 – 4.72 (m, 2H), 4.09 (p, *J* = 7.0 Hz, 1H), 2.86 – 2.77 (m, 2H), 2.72 – 2.64 (m, 2H). Other peaks

overlap with the solvent and are difficult to confirm.

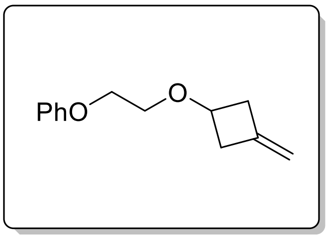

(2-(3-methylenecyclobutoxy)ethoxy)benzene (4e). Yield 54% (10.9 mg). Colourless oil. ^{1}H NMR (500 MHz, CD_2Cl_2) δ 7.28 (t, *J* = 7.8 Hz, 2H), 6.95 (t, *J* = 7.4 Hz, 1H), 6.91 (d, *J* = 8.2 Hz, 2H), 4.86 (t, *J* = 2.6 Hz, 2H), 4.15 – 4.05 (m, 3H), 3.71 (t, *J* = 4.8 Hz, 2H), 2.99 – 2.85 (m, 2H), 2.71 (ddt, *J* = 14.0, 6.6, 3.1 Hz, 2H).; ^{13}C NMR (125 MHz, CD_2Cl_2) δ 159.2, 141.7, 129.8, 121.2, 114.8, 107.1, 70.1, 67.7, 67.2, 40.6.

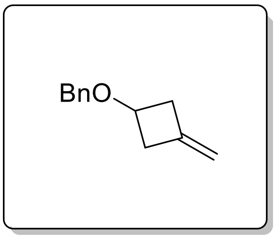

((3-methylenecyclobutoxy)methyl)benzene (4f). Yield 75% (13.0 mg). Colourless oil. ^{1}H NMR (600 MHz, $CDCl_3$) δ 7.36 – 7.33 (m, 4H), 7.29 (dt, *J* = 8.7, 4.4 Hz, 1H), 4.94 – 4.74 (m, 2H), 4.44 (s, 2H), 4.11 (p, *J* = 6.6 Hz, 1H), 2.94 – 2.82 (m, 2H), 2.80 – 2.67 (m, 2H).; ^{13}C NMR (150 MHz, $CDCl_3$) δ 141.3, 138.2, 128.6, 128.0, 127.8, 107.1, 70.6, 69.0, 40.4. The NMR data are consistent with the literature.[S5]

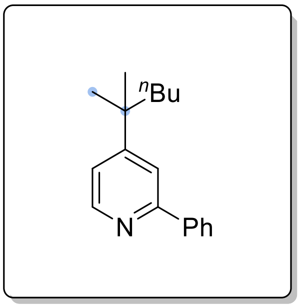

4-(2-methylhexan-2-yl)-2-phenylpyridine (6a). Yield 82% (20.8 mg) for 0.1 mmol scale. Yield 77% (194.5 mg) for 1.0 mmol scale. Colourless oil. ^{1}H NMR (400 MHz, $CDCl_3$) δ 8.60 (dd, *J* = 5.3, 0.8 Hz, 1H), 8.02 – 7.94 (m, 2H), 7.66 (dd, *J* = 1.8, 0.8 Hz, 1H), 7.52 – 7.45 (m, 2H), 7.44 – 7.38 (m, 1H), 7.18 (dd, *J* = 5.3, 1.8 Hz, 1H), 1.69 – 1.54 (m, 2H), 1.34 (s, 6H), 1.31 – 1.17 (m, 2H), 1.11 – 1.01 (m, 2H), 0.84 (t, *J* = 7.3 Hz, 3H).; ^{13}C NMR (100 MHz, $CDCl_3$) δ 159.8, 157.6, 149.6, 140.2, 128.8, 128.8, 127.2, 120.1, 118.5, 43.8, 38.1, 28.4, 27.0, 23.4, 14.1.; HRMS (ESI) m/z calcd. for $[C_{18}H_{24}N]^+$: 254.1903, found: 254.1909.

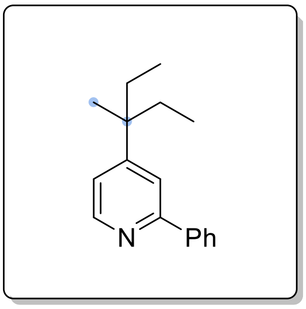

4-(3-methylpentan-3-yl)-2-phenylpyridine (6b). Yield 73% (17.4 mg) Colourless oil. ^{1}H NMR (400 MHz, $CDCl_3$) δ 8.60 (dd, *J* = 5.3, 0.7 Hz, 1H), 8.02 – 7.94 (m, 2H), 7.64 – 7.59 (m, 1H), 7.52 – 7.44 (m, 2H), 7.44 – 7.37 (m, 1H), 7.15 (dd, *J* = 5.3, 1.8 Hz, 1H), 1.86 – 1.73 (m, 2H), 1.67 – 1.56 (m, 2H), 1.30 (s, 3H), 0.71 (t, *J* = 7.5 Hz, 6H).; ^{13}C NMR (100 MHz, $CDCl_3$) δ 158.1, 157.5, 149.5, 140.1, 128.9, 128.8, 127.2, 120.9, 119.2, 41.8, 34.8, 22.3, 8.7.; HRMS (ESI) m/z calcd. for $[C_{17}H_{22}N]^+$: 240.1747, found: 240.1753.

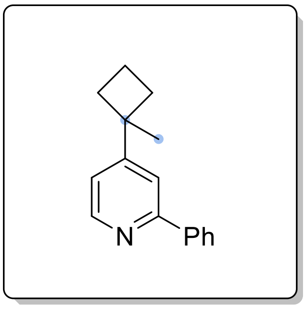

4-(1-methylcyclobutyl)-2-phenylpyridine (6c). Yield 74% (16.5 mg). Colourless oil. ^{1}H NMR (400 MHz, $CDCl_3$) δ 8.59 (d, *J* = 5.0 Hz, 1H), 8.01 – 7.94 (m, 2H), 7.52 – 7.44 (m, 3H), 7.44 – 7.38 (m, 1H), 7.04 (dd, *J* = 5.1, 1.7 Hz, 1H), 2.47 – 2.37 (m, 2H), 2.23 – 2.05 (m, 3H), 1.93 – 1.81 (m, 1H), 1.50 (s, 3H).; ^{13}C NMR (100 MHz, $CDCl_3$) δ 162.0, 157.6, 149.6, 140.0, 128.9, 128.8, 127.1, 119.0, 117.4, 42.8, 33.9, 29.8, 15.8.; HRMS (ESI) m/z calcd. for $[C_{16}H_{18}N]^+$: 224.1434, found: 224.1439.

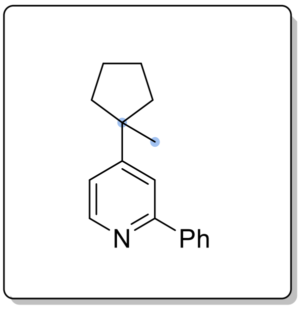

4-(1-methylcyclopentyl)-2-phenylpyridine (6d). Yield 75% (17.7 mg). Colourless oil. ^{1}H NMR (400 MHz, $CDCl_3$) δ 8.58 (d, *J* = 5.3 Hz, 1H), 8.01 – 7.93 (m, 2H), 7.66 (d, *J* = 1.7 Hz, 1H), 7.47 (dd, *J* = 8.3, 6.7 Hz, 2H), 7.44 – 7.37 (m, 1H), 7.19 (dd, *J* = 5.2, 1.8 Hz, 1H), 2.00 – 1.90 (m, 2H), 1.89 – 1.72

(m, 6H), 1.31 (s, 3H).; ^{13}C NMR (100 MHz, $CDCl_3$) δ 161.1, 157.6, 149.6, 140.1, 128.8, 128.8, 127.2, 120.2, 118.7, 47.3, 39.4, 28.9, 23.9.; HRMS (ESI) m/z calcd. for $[C_{17}H_{20}N]^+$: 238.1590, found: 238.1597

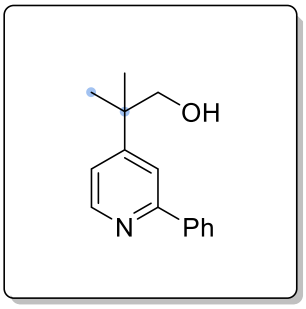

2-methyl-2-(2-phenylpyridin-4-yl)propan-1-ol (6e). Yield 66% (15.1 mg). Colourless oil. ^{1}H NMR (400 MHz, $CDCl_3$) δ 8.49 (d, *J* = 5.3 Hz, 1H), 7.96 – 7.88 (m, 2H), 7.67 (d, *J* = 1.8 Hz, 1H), 7.51 – 7.45 (m, 2H), 7.44 – 7.39 (m, 1H), 7.17 (dd, *J* = 5.4, 1.8 Hz, 1H), 3.63 (s, 2H), 2.41 (s, 1H), 1.34 (s, 6H).; ^{13}C NMR (100 MHz, $CDCl_3$) δ 157.5, 157.4, 149.4, 139.5, 129.1, 128.9, 127.3, 120.4, 119.1, 72.4, 40.5, 24.8.; HRMS (ESI) m/z calcd. for $[C_{15}H_{18}NO]^+$: 228.1383, found: 228.1389

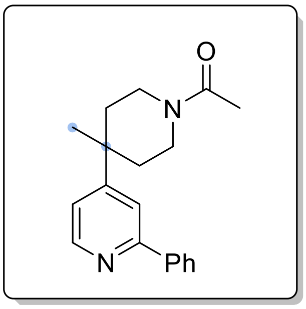

1-(4-methyl-4-(2-phenylpyridin-4-yl)piperidin-1-yl)ethan-1-one (6f). Yield 73% (21.4 mg). Colourless oil. ^{1}H NMR (400 MHz, $CDCl_3$) δ 8.64 (d, *J* = 5.2 Hz, 1H), 7.98 – 7.91 (m, 2H), 7.64 (d, *J* = 1.9 Hz, 1H), 7.48 (dd, *J* = 8.3, 6.7 Hz, 2H), 7.44 – 7.39 (m, 1H), 7.18 (dd, *J* = 5.4, 1.8 Hz, 1H), 3.75 – 3.65 (m, 1H), 3.64 – 3.49 (m, 2H), 3.48 – 3.35 (m, 1H), 2.19 – 2.08 (m, 5H), 1.84 – 1.70 (m, 2H), 1.33 (s, 3H).; ^{13}C NMR (100 MHz, $CDCl_3$) δ 169.0, 158.3, 157.7, 150.2, 139.8, 129.1, 128.9, 127.1, 119.6, 118.1, 43.3, 38.2, 37.1, 37.1, 36.0, 28.5, 21.6.; HRMS (ESI) m/z calcd. for $[C_{19}H_{23}N_2O]^+$: 295.1805, found: 295.1811

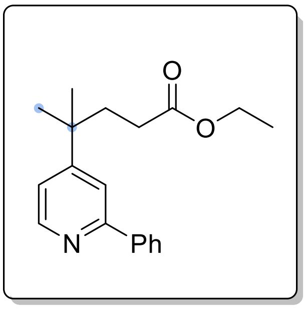

ethyl 4-methyl-4-(2-phenylpyridin-4-yl)pentanoate (6g). Yield 75% (22.2 mg). Colourless oil. ^{1}H NMR (400 MHz, $CDCl_3$) δ 8.54 (dd, *J* = 5.3, 0.8 Hz, 1H), 7.93 – 7.86 (m, 2H), 7.61 – 7.55 (m, 1H), 7.45 – 7.37 (m, 2H), 7.37 – 7.31 (m, 1H), 7.11 (dd, *J* = 5.3, 1.8 Hz, 1H), 3.97 (q, *J* = 7.1 Hz, 2H), 2.06 – 2.00 (m, 2H), 1.99 – 1.93 (m, 2H), 1.30 (s, 6H), 1.12 (t, *J* = 7.1 Hz, 3H).; ^{13}C NMR (100 MHz, $CDCl_3$) δ 173.6, 158.2, 157.8, 149.8, 139.9, 129.0, 128.8, 127.2, 119.9, 118.4, 60.6, 38.3, 37.7, 30.2, 28.2, 14.3.; HRMS (ESI) m/z calcd. for $[C_{19}H_{24}NO_2]^+$: 298.1802, found: 298.1807

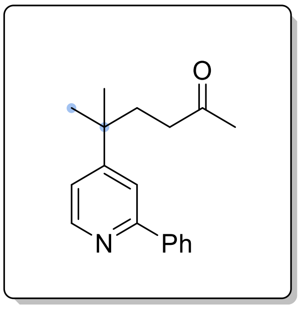

5-methyl-5-(2-phenylpyridin-4-yl)hexan-2-one (6h). Yield 71% (18.8 mg). Colourless oil. ^{1}H NMR (400 MHz, $CDCl_3$) δ 8.61 (dd, *J* = 5.3, 0.8 Hz, 1H), 8.00 – 7.93 (m, 2H), 7.67 – 7.62 (m, 1H), 7.52 – 7.44 (m, 2H), 7.44 – 7.38 (m, 1H), 7.17 (dd, *J* = 5.3, 1.8 Hz, 1H), 2.23 – 2.17 (m, 2H), 2.05 (s, 3H), 2.01 – 1.94 (m, 2H), 1.35 (s, 6H).; ^{13}C NMR (100 MHz, $CDCl_3$) δ 208.5, 158.4, 157.8, 149.8, 139.8, 129.0, 128.9, 127.2, 119.9, 118.3, 39.4, 37.5, 36.8, 30.1, 28.3.; HRMS (ESI) m/z calcd. for $[C_{18}H_{22}NO]^+$: 268.1696, found: 268.1700

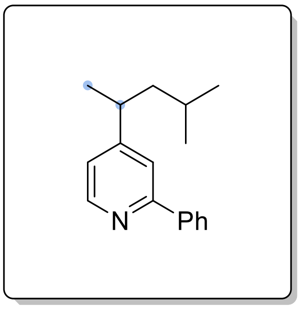

4-(4-methylpentan-2-yl)-2-phenylpyridine (6i). Yield 68% (16.2 mg). Colourless oil. ^{1}H NMR (500 MHz, $CDCl_3$) δ 8.59 (d, *J* = 5.1 Hz, 1H), 7.99 (dd, *J* = 7.3, 1.7 Hz, 2H), 7.54 (d, *J* = 1.6 Hz, 1H), 7.48 (t, *J* = 7.5 Hz, 2H), 7.41 (t, *J* = 7.3 Hz, 1H), 7.08 (dd, *J* = 5.1, 1.6 Hz, 1H), 2.85 (h, *J* = 6.9 Hz, 1H), 1.61 – 1.53 (m, 1H), 1.52 – 1.39 (m, 2H), 1.27 (d, *J* = 6.9 Hz, 3H), 0.91 (d, *J* = 6.2 Hz, 3H), 0.88 (d, *J* = 6.2 Hz, 3H).; ^{13}C NMR (100 MHz, $CDCl_3$) δ 158.1, 157.6, 149.6, 139.7, 129.0, 128.8, 127.2, 121.2, 119.8, 47.1, 37.6, 25.7, 23.0, 22.5, 22.1.; HRMS (ESI) m/z calcd. for $[C_{17}H_{22}N]^+$: 240.1747, found: 240.1753

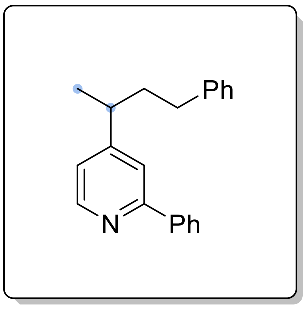

2-phenyl-4-(4-phenylbutan-2-yl)pyridine (6j). Yield 71% (20.3 mg). Colourless oil. ^{1}H NMR (500 MHz, CD_2Cl_2) δ 8.58 (d, *J* = 5.0 Hz, 1H), 8.06 – 7.96 (m, 2H), 7.60 (s, 1H), 7.48 (t, *J* = 7.5 Hz, 2H), 7.45 – 7.39 (m, 1H), 7.27 (t, *J* = 7.6 Hz, 2H), 7.20 – 7.13 (m, 3H), 7.11 (dd, *J* = 5.0, 1.6 Hz, 1H), 2.80 (h, *J* = 7.0 Hz, 1H), 2.63 – 2.46 (m, 2H), 1.97 (dp, *J* = 9.2, 6.9 Hz, 2H), 1.33 (d, *J* = 6.9 Hz, 3H).; ^{13}C NMR (100 MHz, CD_2Cl_2) δ 157.6, 157.4, 150.1, 142.6, 140.1, 129.1, 129.0, 128.7, 128.7, 127.2, 126.2, 121.5, 119.8, 39.7, 39.6, 34.1, 21.9.; HRMS (ESI) m/z calcd. for $[C_{21}H_{22}N]^+$: 288.1747, found: 288.1753

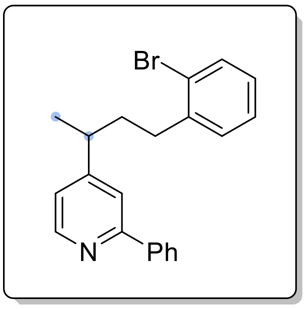

4-(4-(2-bromophenyl)butan-2-yl)-2-phenylpyridine (6k). Yield 67% (24.6 mg). Colourless oil. ^{1}H NMR (600 MHz, $CDCl_3$) δ 8.62 (d, *J* = 5.0 Hz, 1H), 8.00 (d, *J* = 7.6 Hz, 2H), 7.60 (s, 1H), 7.53 – 7.46 (m, 3H), 7.42 (t, *J* = 7.5 Hz, 1H), 7.21 (t, *J* = 7.5 Hz, 1H), 7.16 – 7.11 (m, 2H), 7.04 (td, *J* = 7.7, 1.8 Hz, 1H), 2.85 (h, *J* = 7.2 Hz, 1H), 2.76 – 2.68 (m, 1H), 2.68 – 2.60 (m, 1H), 2.02 – 1.90 (m, 2H), 1.36 (d, *J* = 6.8 Hz, 3H).; ^{13}C NMR (100 MHz, $CDCl_3$) δ 157.8, 156.9, 149.8, 141.4, 139.7, 133.0, 130.4, 129.0, 128.8, 127.8, 127.6, 127.1, 124.4, 121.2, 119.8, 39.8, 37.7, 34.5, 21.9.; HRMS (ESI) m/z calcd. for $[C_{21}H_{21}BrN]^+$: 366.0852, found: 366.0857

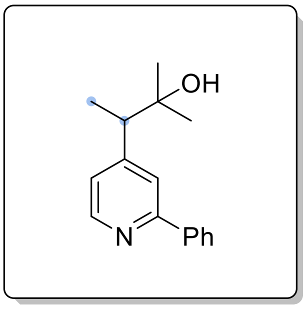

2-methyl-3-(2-phenylpyridin-4-yl)butan-2-ol (6l). Yield 48% (11.5 mg). Colourless oil. ^{1}H NMR (600 MHz, $CDCl_3$) δ 8.58 (d, *J* = 5.1 Hz, 1H), 7.98 – 7.95 (m, 2H), 7.63 – 7.57 (m, 1H), 7.49 – 7.44 (m, 2H), 7.42 – 7.37 (m, 1H), 7.13 (dd, *J* = 5.1, 1.6 Hz, 1H), 2.82 (q, *J* = 7.1 Hz, 1H), 1.38 (d, *J* = 7.1 Hz, 3H), 1.23 (s, 3H), 1.19 (s, 3H).; ^{13}C NMR (100 MHz, $CDCl_3$) δ 157.4, 153.6, 149.4, 139.7, 129.0, 128.8,

127.2, 123.0, 121.6, 72.5, 50.3, 28.5, 27.6, 15.5.; HRMS (ESI) m/z calcd. for $[C_{16}H_{20}NO]^+$: 242.1539, found: 242.1546

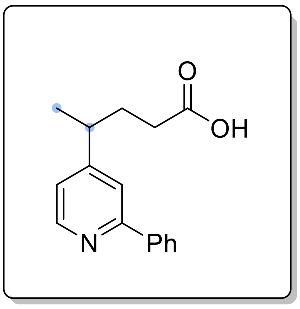

4-(2-phenylpyridin-4-yl)pentanoic acid (6m). Yield 66% (16.9 mg). Colourless oil. ^{1}H NMR (400 MHz, $CDCl_3$) δ 8.62 (d, *J* = 5.1 Hz, 1H), 7.94 – 7.88 (m, 2H), 7.52 (d, *J* = 1.6 Hz, 1H), 7.49 – 7.38 (m, 3H), 7.09 (dd, *J* = 5.1, 1.7 Hz, 1H), 2.83 (h, *J* = 7.0 Hz, 1H), 2.31 – 2.24 (m, 2H), 2.02 – 1.93 (m, 2H), 1.32 (d, *J* = 6.9 Hz, 3H).; ^{13}C NMR (100 MHz, $CDCl_3$) δ 178.0, 157.9, 156.6, 149.6, 139.3, 129.1, 128.9, 127.3, 121.2, 120.2, 39.1, 32.4, 32.2, 21.5.; HRMS (ESI) m/z calcd. for $[C_{16}H_{18}NO_2]^+$: 256.1332, found: 256.1337

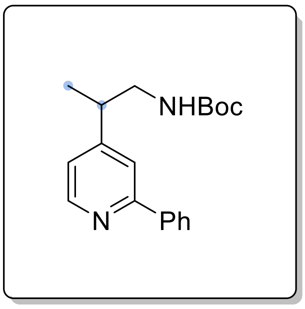

tert-butyl (2-(2-phenylpyridin-4-yl)propyl)carbamate (6n). Yield 65% (20.3 mg). Colourless oil. ^{1}H NMR (500 MHz, $CDCl_3$) δ 8.61 (d, *J* = 5.1 Hz, 1H), 7.98 (d, *J* = 7.6 Hz, 2H), 7.56 (s, 1H), 7.47 (t, *J* = 7.3 Hz, 2H), 7.45 – 7.38 (m, 1H), 7.08 (dd, *J* = 5.1, 1.6 Hz, 1H), 4.57 (t, *J* = 6.6 Hz, 1H), 3.42 (dt, *J* = 13.5, 6.4 Hz, 1H), 3.26 (ddd, *J* = 13.8, 8.2, 5.8 Hz, 1H), 3.02 (h, *J* = 6.7 Hz, 1H), 1.40 (s, 9H), 1.31 (d, *J* = 7.0 Hz, 3H).; ^{13}C NMR (125 MHz, $CDCl_3$) δ 157.8, 156.0, 154.3, 149.9, 139.4, 129.1, 128.9, 127.2, 121.2, 120.0, 79.6, 46.8, 40.0, 28.5, 18.5.; HRMS (ESI) m/z calcd. for $[C_{19}H_{25}N_2O_2]^+$: 313.1911, found: 313.1916

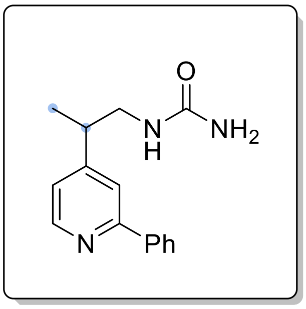

1-(2-(2-phenylpyridin-4-yl)propyl)urea (6o). Yield 63% (16.1 mg). Colourless oil. ^{1}H NMR (400 MHz, CD_2Cl_2) δ 8.46 (d, *J* = 5.0 Hz, 1H), 7.99 – 7.91 (m, 2H), 7.58 – 7.53 (m, 1H), 7.51 – 7.42 (m, 3H), 7.04 (dd, *J* = 5.2, 1.6 Hz, 1H), 5.09 (s, 1H), 4.55 (s, 2H), 3.50 – 3.39 (m, 1H), 3.22 – 3.11 (m, 1H), 3.03 – 2.90 (m, 1H), 1.25 (d, *J* = 7.0 Hz, 3H).; ^{13}C NMR (125 MHz, CD_2Cl_2) δ 158.9, 157.6, 155.3, 149.7, 139.5, 129.4, 129.1, 127.3, 121.7, 120.3, 46.6, 40.5, 18.8.; HRMS (ESI) m/z calcd. for $[C_{15}H_{18}N_3O]^+$: 256.1444, found: 228.1450

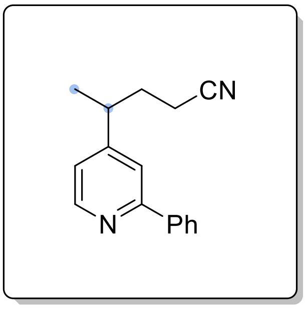

4-(2-phenylpyridin-4-yl)pentanenitrile (6p). Yield 60% (14.1 mg). Colourless oil. ^{1}H NMR (400 MHz, $CDCl_3$) δ 8.64 (dd, *J* = 5.1, 0.7 Hz, 1H), 8.02 – 7.95 (m, 2H), 7.59 – 7.54 (m, 1H), 7.52 – 7.46 (m, 2H), 7.46 – 7.40 (m, 1H), 7.09 (dd, *J* = 5.1, 1.7 Hz, 1H), 2.97 (dp, *J* = 9.1, 6.8 Hz, 1H), 2.32 (ddd, *J* = 16.9, 7.2, 6.2 Hz, 1H), 2.18 (dt, *J* = 16.9, 7.7 Hz, 1H), 2.09 – 1.89 (m, 2H), 1.37 (d, *J* = 6.9 Hz, 3H).; ^{13}C NMR (100 MHz, $CDCl_3$) δ 158.1, 154.8, 150.1, 139.1, 129.3, 128.9, 127.2, 120.8, 119.6, 119.2, 38.7, 32.9, 21.3, 15.6.; HRMS (ESI) m/z calcd. for $[C_{16}H_{17}N_2]^+$: 237.1386, found: 237.1393

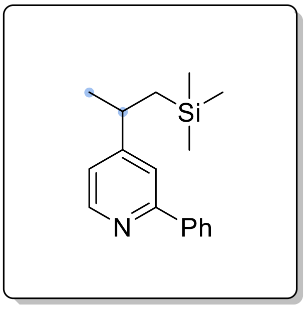

2-phenyl-4-(1-(trimethylsilyl)propan-2-yl)pyridine (6q). Yield 71% (19.2 mg). Colourless oil. ^{1}H NMR (400 MHz, $CDCl_3$) δ 8.58 (dd, *J* = 5.1, 0.8 Hz, 1H), 8.02 – 7.95 (m, 2H), 7.60 – 7.55 (m, 1H), 7.51 – 7.44 (m, 2H), 7.44 – 7.38 (m, 1H), 7.10 (dd, *J* = 5.1, 1.7 Hz, 1H), 2.93 (h, *J* = 7.0 Hz, 1H), 1.32 (d, *J* = 7.0 Hz, 3H), 1.08 – 0.88 (m, 2H), -0.04 (s, 9H).; ^{13}C NMR (100 MHz, $CDCl_3$) δ 159.9, 157.6, 149.6, 139.7, 129.0, 128.8, 127.1, 120.9, 119.3, 36.3, 26.3, 25.5, -0.7.; HRMS (ESI) m/z calcd. for $[C_{17}H_{24}NSi]^+$: 270.1673, found: 270.1679

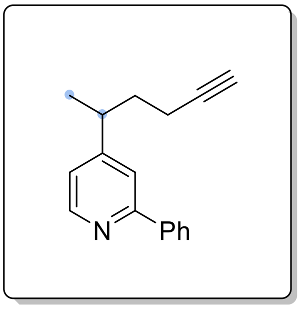

4-(hex-5-yn-2-yl)-2-phenylpyridine (6r). Yield 48% (11.3 mg). Colourless oil. ^{1}H NMR (400 MHz, $CDCl_3$) δ 8.60 (dd, *J* = 5.1, 0.8 Hz, 1H), 8.02 – 7.94 (m, 2H), 7.56 (t, *J* = 1.1 Hz, 1H), 7.50 – 7.45 (m, 2H), 7.44 – 7.39 (m, 1H), 7.09 (dd, *J* = 5.1, 1.7 Hz, 1H), 2.98 (h, *J* = 7.1 Hz, 1H), 2.18 (dtd, *J* = 16.6, 6.9, 2.6 Hz, 1H), 2.07 (dtd, *J* = 17.0, 7.4, 2.6 Hz, 1H), 1.99 (t, *J* = 2.6 Hz, 1H), 1.90 – 1.82 (m, 2H), 1.33 (d, *J* = 7.0 Hz, 3H).; ^{13}C NMR (100 MHz, $CDCl_3$) δ 157.8, 156.3, 149.9, 139.7, 129.1, 128.9, 127.1, 121.1, 119.8, 83.8, 69.1, 38.4, 36.2, 21.3, 16.7.; HRMS (ESI) m/z calcd. for $[C_{17}H_{18}N]^+$: 236.1434, found: 236.1440

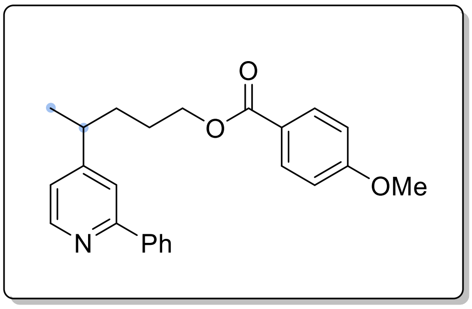

4-(2-phenylpyridin-4-yl)pentyl 4-methoxybenzoate (6s). Yield 70% (26.3 mg). Colourless oil. ^{1}H NMR (500 MHz, CD_2Cl_2) δ 8.57 (d, *J* = 5.0 Hz, 1H), 8.04 – 7.99 (m, 2H), 7.95 (d, *J* = 8.9 Hz, 2H), 7.60 (d, *J* = 1.7 Hz, 1H), 7.47 (dd, *J* = 8.2, 6.6 Hz, 2H), 7.44 – 7.38 (m, 1H), 7.11 (dd, *J* = 5.1, 1.6 Hz, 1H), 6.90 (d, *J* = 8.8 Hz, 2H), 4.25 (t, *J* = 6.3 Hz, 2H), 3.84 (s, 3H), 2.84 (h, *J* = 6.9 Hz, 1H), 1.84 – 1.77 (m, 2H), 1.77 – 1.71 (m, 1H), 1.70 – 1.60 (m, 1H), 1.33 (d, *J* = 6.9 Hz, 3H).; ^{13}C NMR (100 MHz, CD_2Cl_2) δ 166.5, 163.8, 157.6, 157.3, 150.0, 140.0, 131.8, 129.2, 129.0, 127.3, 123.2, 121.5, 119.7, 114.0, 64.8, 55.8, 39.8, 34.3, 27.2, 21.8.; HRMS (ESI) m/z calcd. for $[C_{24}H_{26}NO_3]^+$: 376.1907, found: 376.1915

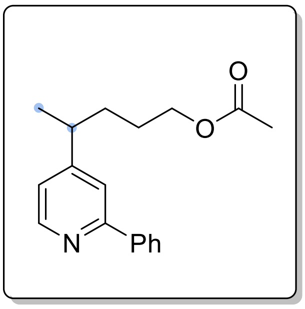

4-(2-phenylpyridin-4-yl)pentyl acetate (6t). Yield 65% (18.4 mg). Colourless oil. ^{1}H NMR (400 MHz, $CDCl_3$) δ 8.59 (dd, *J* = 5.0, 0.8 Hz, 1H), 8.01 – 7.94 (m, 2H), 7.55 – 7.52 (m, 1H), 7.51 – 7.44 (m, 2H),

7.44 – 7.38 (m, 1H), 7.06 (dd, *J* = 5.0, 1.4 Hz, 1H), 4.04 (t, *J* = 6.5 Hz, 2H), 2.77 (h, *J* = 7.0 Hz, 1H), 2.03 (s, 3H), 1.76 – 1.47 (m, 4H), 1.31 (d, *J* = 7.0 Hz, 3H).; ^{13}C NMR (100 MHz, $CDCl_3$) δ 171.3, 157.8, 156.9, 149.9, 139.7, 129.0, 128.8, 127.1, 121.1, 119.7, 64.4, 39.5, 33.9, 26.8, 21.7, 21.1.; HRMS (ESI) m/z calcd. for $[C_{18}H_{22}NO_2]^+$: 284.1645, found: 284.1652

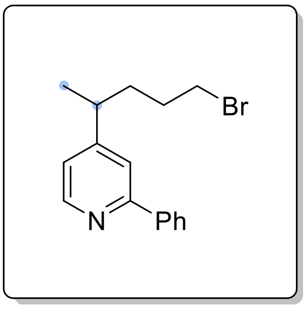

4-(5-bromopentan-2-yl)-2-phenylpyridine (6u). Yield 73% (22.3 mg). Colourless oil. ^{1}H NMR (600 MHz, $CDCl_3$) δ 8.60 (d, *J* = 5.0 Hz, 1H), 8.01 – 7.96 (m, 2H), 7.54 (s, 1H), 7.48 (t, *J* = 7.6 Hz, 2H), 7.41 (t, *J* = 7.4 Hz, 1H), 7.07 (dd, *J* = 5.1, 2.0 Hz, 1H), 3.37 (t, *J* = 6.2, 4.1 Hz, 2H), 2.78 (h, *J* = 7.1 Hz, 1H), 1.89 – 1.71 (m, 4H), 1.32 (d, *J* = 6.8 Hz, 3H).; ^{13}C NMR (125 MHz, $CDCl_3$) δ 157.8, 156.7, 149.9, 139.6, 129.1, 128.9, 127.1, 121.0, 119.6, 39.3, 36.0, 33.7, 30.8, 21.8.; HRMS (ESI) m/z calcd. for $[C_{16}H_{19}BrN]^+$: 304.0695, found: 304.0702

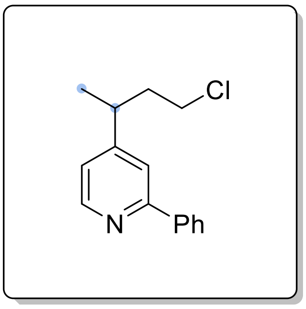

4-(4-chlorobutan-2-yl)-2-phenylpyridine (6v). Yield 67% (16.4 mg). Colourless oil. ^{1}H NMR (400 MHz, $CDCl_3$) δ 8.62 (d, *J* = 5.1 Hz, 1H), 8.00 – 7.95 (m, 2H), 7.57 – 7.55 (m, 1H), 7.51 – 7.45 (m, 2H), 7.44 – 7.39 (m, 1H), 7.09 (dd, *J* = 5.1, 1.6 Hz, 1H), 3.51 (dt, *J* = 11.0, 6.1 Hz, 1H), 3.36 (dt, *J* = 11.0, 7.0 Hz, 1H), 3.08 (h, *J* = 7.1 Hz, 1H), 2.08 (td, *J* = 7.2, 6.1 Hz, 2H), 1.34 (d, *J* = 7.0 Hz, 3H).; ^{13}C NMR (100 MHz, $CDCl_3$) δ 158.0, 155.5, 150.1, 139.6, 129.1, 128.9, 127.1, 121.0, 119.7, 42.9, 40.1, 36.7, 21.1.; HRMS (ESI) m/z calcd. for $[C_{15}H_{17}ClN]^+$: 246.1044, found: 246.1051

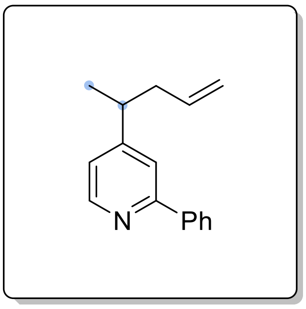

4-(pent-4-en-2-yl)-2-phenylpyridine (6w). Yield 53% (11.8 mg). Colourless oil. ^{1}H NMR (400 MHz, $CDCl_3$) δ 8.59 (d, *J* = 5.2 Hz, 1H), 8.02 – 7.94 (m, 2H), 7.55 (s, 1H), 7.51 – 7.44 (m, 2H), 7.44 – 7.38 (m, 1H), 7.09 – 7.06 (m, 1H), 5.79 – 5.64 (m, 1H), 5.09 – 4.95 (m, 2H), 2.86 (h, *J* = 7.0 Hz, 1H), 2.49 – 2.29 (m, 2H), 1.31 (d, *J* = 7.0 Hz, 3H).; ^{13}C NMR (100 MHz, $CDCl_3$) δ 157.7, 156.8, 149.7, 139.8, 136.2, 129.0, 128.8, 127.1, 121.2, 119.8, 116.9, 42.0, 39.6, 20.9.; HRMS (ESI) m/z calcd. for $[C_{16}H_{18}N]^+$: 224.1434, found: 224.1439

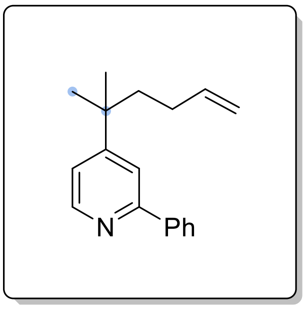

4-(2-methylhex-5-en-2-yl)-2-phenylpyridine (6x). 48 h reaction. Yield 67% (16.8 mg). Colourless oil. ^{1}H NMR (400 MHz, $CDCl_3$) δ 8.61 (d, *J* = 5.4 Hz, 1H), 7.98 (dd, *J* = 7.4, 1.9 Hz, 2H), 7.66 (d, *J* = 1.8 Hz, 1H), 7.48 (dd, *J* = 8.3, 6.6 Hz, 2H), 7.45 – 7.37 (m, 1H), 7.19 (dd, *J* = 5.3, 1.8 Hz, 1H), 5.75 (ddt, *J* = 16.8, 10.3, 6.3 Hz, 1H), 5.01 – 4.87 (m, 2H), 1.90 – 1.81 (m, 2H), 1.81 – 1.72 (m, 2H), 1.37 (s, 6H).; ^{13}C NMR (100 MHz, $CDCl_3$) δ 159.3, 157.7, 149.7, 140.1, 138.8, 128.9, 128.9, 127.2, 120.0, 118.4, 114.5, 43.1, 38.1, 29.2, 28.4.; HRMS (ESI) m/z calcd. for $[C_{18}H_{22}N]^+$: 252.1747, found: 252.1751

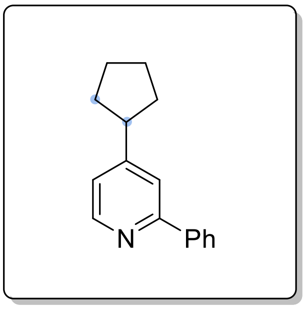

4-cyclopentyl-2-phenylpyridine (6y). Yield 75% (16.6 mg). Colourless oil. ^{1}H NMR (400 MHz, $CDCl_3$) δ 8.57 (dd, *J* = 5.1, 0.8 Hz, 1H), 8.06 – 7.87 (m, 2H), 7.61 – 7.54 (m, 1H), 7.47 (ddt, *J* = 8.2, 6.4, 1.7 Hz, 2H), 7.44 – 7.37 (m, 1H), 7.11 (dd, *J* = 5.1, 1.8 Hz, 1H), 3.05 (tt, *J* = 9.3, 7.5 Hz, 1H), 2.20 – 2.06 (m, 2H), 1.89 – 1.80 (m, 2H), 1.78 – 1.70 (m, 2H), 1.70 – 1.58 (m, 2H).; ^{13}C NMR (100 MHz, $CDCl_3$) δ 157.6, 156.5, 149.6, 139.9, 128.9, 128.8, 127.1, 121.3, 119.8, 45.5, 34.1, 25.7.; HRMS (ESI)

m/z calcd. for $[C_{16}H_{18}N]^+$: 224.1434, found: 224.1438

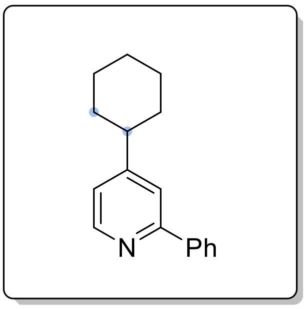

4-cyclohexyl-2-phenylpyridine (6z). Yield 69% (16.3 mg). Colourless oil. ^{1}H NMR (500 MHz, $CDCl_3$) δ 8.57 (d, *J* = 5.1 Hz, 1H), 8.01 – 7.91 (m, 2H), 7.60 – 7.54 (m, 1H), 7.47 (dd, *J* = 8.2, 6.7 Hz, 2H), 7.44 – 7.37 (m, 1H), 7.08 (dd, *J* = 5.1, 1.7 Hz, 1H), 2.57 (tt, *J* = 11.6, 3.4 Hz, 1H), 1.99 – 1.84 (m, 4H), 1.78 (ddt, *J* = 12.9, 3.2, 1.6 Hz, 1H), 1.45 (ddtd, *J* = 25.5, 15.9, 12.6, 3.0 Hz, 4H), 1.34 – 1.21 (m, 1H).; ^{13}C NMR (100 MHz, $CDCl_3$) δ 157.7, 157.5, 149.7, 139.9, 128.9, 128.8, 127.1, 121.0, 119.6, 44.3, 33.7, 26.7, 26.1.; HRMS (ESI) m/z calcd. for $[C_{17}H_{20}N]^+$: 238.1590, found: 238.1597

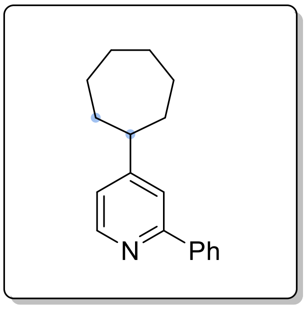

4-cycloheptyl-2-phenylpyridine (6aa). Yield 74% (18.6 mg). Colourless oil. ^{1}H NMR (500 MHz, $CDCl_3$) δ 8.56 (d, *J* = 5.1 Hz, 1H), 8.01 – 7.95 (m, 2H), 7.55 (d, *J* = 1.9 Hz, 1H), 7.49 – 7.44 (m, 2H), 7.44 – 7.37 (m, 1H), 7.07 (dd, *J* = 5.1, 1.6 Hz, 1H), 2.73 (tt, *J* = 10.5, 3.6 Hz, 1H), 1.98 – 1.91 (m, 2H), 1.88 – 1.79 (m, 2H), 1.76 – 1.54 (m, 8H).; ^{13}C NMR (100 MHz, $CDCl_3$) δ 159.5, 157.5, 149.6, 139.7, 128.9, 128.8, 127.1, 120.9, 119.5, 46.7, 36.1, 28.0, 27.3.; HRMS (ESI) m/z calcd. for $[C_{18}H_{22}N]^+$: 252.1747, found: 252.1752

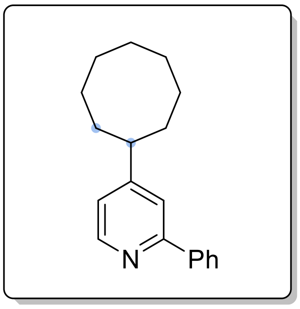

4-cyclooctyl-2-phenylpyridine (6ab). Yield 68% (17.9 mg). Colourless oil. ^{1}H NMR (400 MHz,

$CDCl_3$) δ 8.56 (d, *J* = 5.1 Hz, 1H), 8.03 – 7.94 (m, 2H), 7.54 (d, *J* = 1.6 Hz, 1H), 7.50 – 7.44 (m, 2H), 7.43 – 7.37 (m, 1H), 7.06 (dd, *J* = 5.1, 1.6 Hz, 1H), 2.82 (tt, *J* = 9.7, 3.6 Hz, 1H), 1.96 – 1.74 (m, 6H), 1.73 – 1.54 (m, 8H).; ^{13}C NMR (100 MHz, $CDCl_3$) 159.8, 157.6, 149.7, 139.9, 128.9, 128.8, 127.1, 121.2, 119.7, 44.5, 33.9, 26.9, 26.4, 26.0.; HRMS (ESI) m/z calcd. for $[C_{19}H_{24}N]^+$: 266.1903, found: 266.1910

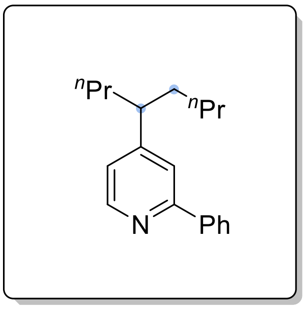

4-(octan-4-yl)-2-phenylpyridine (6ac). Using $Fe(acac)_3$ 10 mol%. Yield 48% (12.8 mg). Colourless oil. ^1H NMR (500 MHz, $CDCl_3$) δ 8.58 (d, *J* = 5.0 Hz, 1H), 8.02 – 7.96 (m, 2H), 7.52 – 7.44 (m, 3H), 7.44 – 7.38 (m, 1H), 7.03 (dd, *J* = 5.0, 1.6 Hz, 1H), 2.57 (tt, *J* = 9.2, 5.3 Hz, 1H), 1.73 – 1.52 (m, 4H), 1.37 – 1.05 (m, 6H), 0.85 (dt, *J* = 12.2, 7.3 Hz, 6H).; ^{13}C NMR (100 MHz, $CDCl_3$) δ 157.5, 156.5, 149.6, 139.8, 128.9, 128.8, 127.2, 121.9, 120.5, 45.8, 38.7, 36.1, 29.8, 22.9, 20.8, 14.2, 14.1.; HRMS (ESI) m/z calcd. for $[C_{19}H_{26}N]^+$: 268.2060, found: 268.2066

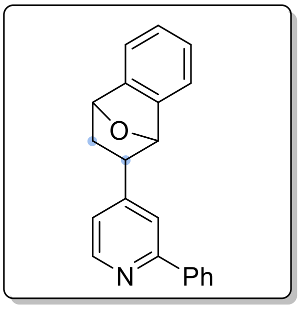

2-phenyl-4-(1,2,3,4-tetrahydro-1,4-epoxynaphthalen-2-yl)pyridine (6ad). Yield 53% (15.9 mg). Colourless oil. ^1H NMR (400 MHz, $CDCl_3$) δ 8.63 (d, *J* = 5.1 Hz, 1H), 8.05 – 7.97 (m, 2H), 7.79 – 7.74 (m, 1H), 7.52 – 7.45 (m, 2H), 7.45 – 7.39 (m, 1H), 7.34 – 7.27 (m, 3H), 7.24 – 7.20 (m, 2H), 5.60 (t, *J* = 2.6 Hz, 1H), 5.34 (s, 1H), 2.92 (dd, *J* = 7.1, 5.7 Hz, 1H), 2.14 – 2.08 (m, 2H).; ^{13}C NMR (100 MHz, $CDCl_3$) δ 157.7, 155.3, 149.7, 146.0, 145.4, 139.4, 129.1, 128.9, 127.2, 127.0, 121.6, 120.1, 119.4, 119.1, 84.8, 79.3, 45.8, 38.5.; HRMS (ESI) m/z calcd. for $[C_{21}H_{17}NONa]^+$: 322.1202, found: 322.1207

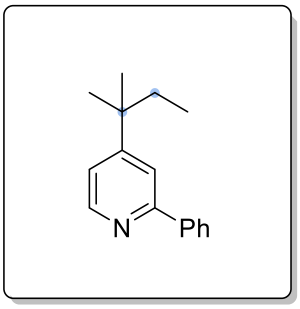

4-(tert-pentyl)-2-phenylpyridine (6ae). Yield 75% (16.9 mg). Colourless oil. ^{1}H NMR (400 MHz, $CDCl_3$) δ 8.60 (dd, *J* = 5.3, 0.8 Hz, 1H), 8.02 – 7.90 (m, 2H), 7.65 (dd, *J* = 1.8, 0.8 Hz, 1H), 7.51 – 7.45 (m, 2H), 7.44 – 7.37 (m, 1H), 7.19 (dd, *J* = 5.3, 1.8 Hz, 1H), 1.70 (q, *J* = 7.5 Hz, 2H), 1.33 (s, 6H), 0.73 (t, *J* = 7.5 Hz, 3H).; ^{13}C NMR (100 MHz, $CDCl_3$) δ 159.5, 157.6, 149.6, 140.2, 128.9, 128.8, 127.2, 120.2, 118.6, 38.3, 36.4, 27.9, 9.2.; HRMS (ESI) m/z calcd. for $[C_{16}H_{20}N]^+$: 226.1590, found: 226.1597

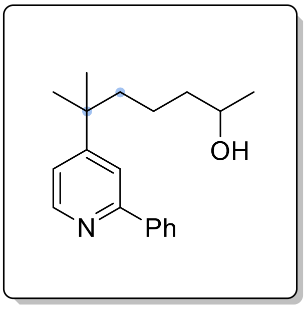

6-methyl-6-(2-phenylpyridin-4-yl)heptan-2-ol (6af). Yield 53% (14.9 mg). Colourless oil. ^{1}H NMR (400 MHz, $CDCl_3$) δ 8.59 (d, *J* = 5.3 Hz, 1H), 8.00 – 7.92 (m, 2H), 7.65 (d, *J* = 1.8 Hz, 1H), 7.51 – 7.44 (m, 2H), 7.43 – 7.38 (m, 1H), 7.18 (dd, *J* = 5.3, 1.9 Hz, 1H), 3.77 – 3.65 (m, 1H), 1.75 – 1.58 (m, 2H), 1.42 – 1.30 (m, 8H), 1.28 – 1.15 (m, 2H), 1.11 (d, *J* = 6.3 Hz, 3H).; ^{13}C NMR (100 MHz, $CDCl_3$) δ 159.7, 157.6, 149.5, 140.0, 128.9, 128.8, 127.2, 120.0, 118.5, 68.0, 44.0, 39.9, 38.1, 28.4, 28.3, 23.7, 21.1.; HRMS (ESI) m/z calcd. for $[C_{19}H_{26}NO]^+$: 284.2009, found: 284.2015

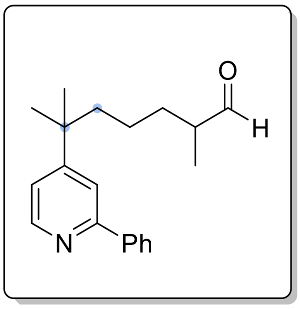

2,6-dimethyl-6-(2-phenylpyridin-4-yl)heptanal (6ag). 24 h reaction. Yield 50% (14.8 mg). Colourless oil. ^{1}H NMR (400 MHz, $CDCl_3$) δ 9.55 (d, *J* = 2.0 Hz, 1H), 8.60 (dd, *J* = 5.3, 0.7 Hz, 1H), 8.02 – 7.88 (m, 2H), 7.64 (dd, *J* = 1.9, 0.8 Hz, 1H), 7.54 – 7.45 (m, 2H), 7.45 – 7.38 (m, 1H), 7.17 (dd, *J* = 5.3, 1.8 Hz, 1H), 2.27 (hd, *J* = 6.9, 2.0 Hz, 1H), 1.66 (t, *J* = 8.5 Hz, 3H), 1.34 (s, 6H), 1.31 – 1.22 (m, 1H), 1.19 – 1.09 (m, 2H), 1.02 (d, *J* = 7.0 Hz, 3H).; ^{13}C NMR (100 MHz, $CDCl_3$) δ 205.1, 159.3, 157.7, 149.7, 140.1, 128.9, 128.8, 127.2, 119.9, 118.3, 46.3, 44.0, 38.1, 31.1, 28.3, 22.2, 13.5.; HRMS

(ESI) m/z calcd. for $[C_{20}H_{26}NO]^+$: 296.2009, found: 296.2016

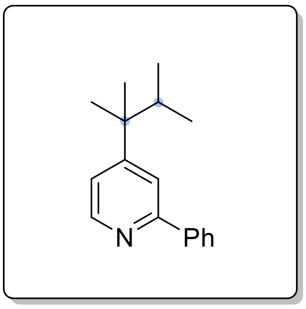

4-(2,3-dimethylbutan-2-yl)-2-phenylpyridine (6ah). Yield 65% (15.7 mg). Colourless oil. ^{1}H NMR (400 MHz, $CDCl_3$) δ 8.59 (dd, *J* = 5.3, 0.8 Hz, 1H), 7.99 – 7.94 (m, 2H), 7.66 (dd, *J* = 1.9, 0.8 Hz, 1H), 7.52 – 7.45 (m, 2H), 7.45 – 7.37 (m, 1H), 7.19 (dd, *J* = 5.3, 1.9 Hz, 1H), 1.97 (hept, *J* = 6.8 Hz, 1H), 1.29 (s, 6H), 0.81 (d, *J* = 6.8 Hz, 6H).; ^{13}C NMR (100 MHz, $CDCl_3$) δ 160.4, 157.4, 149.4, 140.2, 128.9, 128.8, 127.2, 120.4, 118.8, 41.0, 37.9, 24.3, 18.0.; HRMS (ESI) m/z calcd. for $[C_{17}H_{22}N]^+$: 240.1747, found: 240.1753

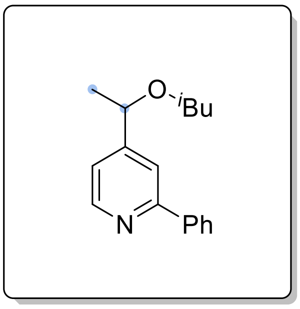

4-(1-isobutoxyethyl)-2-phenylpyridine (6ai). Using $Fe(acac)_3$ 10 mol%. Yield 73% (18.6 mg). Colourless oil. ^{1}H NMR (500 MHz, CD_2Cl_2) δ 8.61 (dd, *J* = 5.0, 0.9 Hz, 1H), 8.07 – 8.00 (m, 2H), 7.74 – 7.70 (m, 1H), 7.52 – 7.45 (m, 2H), 7.45 – 7.38 (m, 1H), 7.20 (dd, *J* = 5.0, 1.6 Hz, 1H), 4.43 (q, *J* = 6.5 Hz, 1H), 3.20 – 3.06 (m, 2H), 1.87 (dp, *J* = 13.3, 6.7 Hz, 1H), 1.44 (d, *J* = 6.5 Hz, 3H), 0.92 (dd, *J* = 9.0, 6.7 Hz, 6H).; ^{13}C NMR (100 MHz, CD_2Cl_2) δ 157.6, 154.9, 150.0, 139.8, 129.3, 129.0, 127.2, 120.2, 118.1, 77.4, 76.4, 29.1, 23.8, 19.6, 19.5.; HRMS (ESI) m/z calcd. for $[C_{17}H_{22}NO]^+$: 256.1696, found: 256.1702

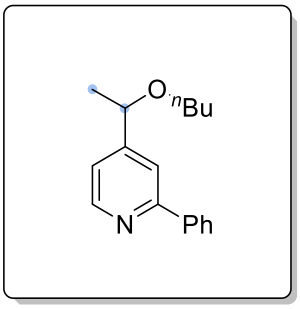

4-(1-butoxyethyl)-2-phenylpyridine (6aj). Using $Fe(acac)_3$ 10 mol%. Yield 72% (18.2 mg). Colourless oil. ^{1}H NMR (500 MHz, $CDCl_3$) δ 8.65 (d, *J* = 5.0 Hz, 1H), 8.03 – 7.95 (m, 2H), 7.68 (s, 1H), 7.48 (dd, *J* = 8.4, 6.7 Hz, 2H), 7.42 (t, *J* = 7.3 Hz, 1H), 7.18 (dd, *J* = 5.0, 1.5 Hz, 1H), 4.43 (q, *J* = 6.6 Hz, 1H), 3.37 (tt, *J* = 6.1, 2.9 Hz, 2H), 1.58 (ddq, *J* = 13.4, 10.6, 6.6 Hz, 2H), 1.46 (d, *J* = 6.6 Hz, 3H), 1.39 (hept, *J* = 7.3 Hz, 2H), 0.91 (t, *J* = 7.4 Hz, 3H).; ^{13}C NMR (100 MHz, $CDCl_3$) δ 157.9, 154.4, 150.0, 139.6, 129.1, 128.9, 127.1, 119.8, 118.0, 77.1, 69.2, 32.1, 23.9, 19.5, 14.1.; HRMS (ESI) m/z calcd. for $[C_{17}H_{22}NO]^+$: 256.1696, found: 256.1701

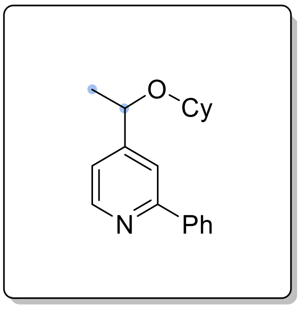

4-(1-(cyclohexyloxy)ethyl)-2-phenylpyridine (6ak). Using $Fe(acac)_3$ 10 mol%. Yield 63% (17.6 mg). Colourless oil. ^{1}H NMR (500 MHz, $CDCl_3$) δ 8.64 (d, *J* = 4.9 Hz, 1H), 8.00 (d, *J* = 7.5 Hz, 2H), 7.71 (s, 1H), 7.48 (t, *J* = 7.5 Hz, 2H), 7.42 (t, *J* = 7.3 Hz, 1H), 7.20 (d, *J* = 5.0 Hz, 1H), 4.63 (q, *J* = 6.6 Hz, 1H), 3.21 (tt, *J* = 9.2, 3.8 Hz, 1H), 1.97 (d, *J* = 12.4 Hz, 1H), 1.84 – 1.70 (m, 3H), 1.54 – 1.48 (m, 1H), 1.44 (d, *J* = 6.5 Hz, 3H), 1.39 – 1.28 (m, 2H), 1.27 – 1.11 (m, 3H).; ^{13}C NMR (100 MHz, $CDCl_3$) δ 157.8, 155.2, 149.9, 139.7, 129.1, 128.9, 127.1, 119.9, 118.1, 75.9, 73.6, 33.5, 32.0, 25.9, 24.6, 24.4, 24.3.; HRMS (ESI) m/z calcd. for $[C_{19}H_{24}NO]^+$: 282.1852, found: 282.1859

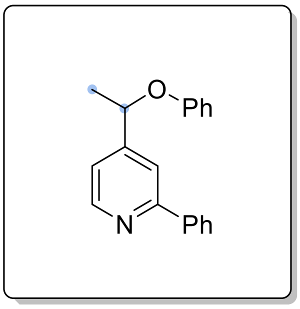

4-(1-phenoxyethyl)-2-phenylpyridine (6al). Using $Fe(acac)_3$ 10 mol%. Yield 69% (19.1 mg). Colourless oil. ^{1}H NMR (500 MHz, $CDCl_3$) δ 8.65 (d, *J* = 5.0 Hz, 1H), 8.02 – 7.94 (m, 2H), 7.73 (s, 1H), 7.47 (dd, *J* = 8.3, 6.7 Hz, 2H), 7.42 (t, *J* = 7.3 Hz, 1H), 7.26 – 7.19 (m, 3H), 6.92 (t, *J* = 7.4 Hz, 1H), 6.87 (d, *J* = 8.1 Hz, 2H), 5.34 (q, *J* = 6.5 Hz, 1H), 1.68 (d, *J* = 6.5 Hz, 3H).; ^{13}C NMR (100 MHz, $CDCl_3$) δ 158.1, 157.6, 153.2, 150.2, 139.4, 129.7, 129.2, 128.9, 127.1, 121.4, 119.2, 117.5, 115.9, 75.0, 24.1.; HRMS (ESI) m/z calcd. for $[C_{19}H_{18}NO]^+$: 276.1383, found: 276.1389

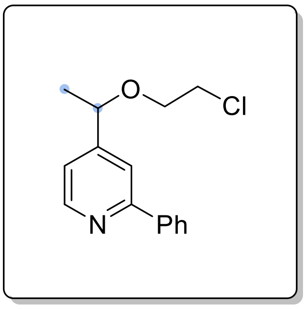

4-(1-(2-chloroethoxy)ethyl)-2-phenylpyridine (6am). Using Fe(acac)$_3$ 10 mol%. Yield 75% (19.7 mg). Colourless oil. ^{1}H NMR (400 MHz, CDCl$_3$) δ 8.66 (d, *J* = 5.0 Hz, 1H), 8.01 (dd, *J* = 8.3, 1.5 Hz, 2H), 7.74 – 7.68 (m, 1H), 7.48 (dd, *J* = 8.3, 6.5 Hz, 2H), 7.45 – 7.39 (m, 1H), 7.20 (dd, *J* = 5.1, 1.6 Hz, 1H), 4.53 (q, *J* = 6.5 Hz, 1H), 3.69 – 3.61 (m, 4H), 1.51 (d, *J* = 6.5 Hz, 3H).; ^{13}C NMR (100 MHz, CDCl$_3$) δ 158.1, 153.3, 150.1, 139.4, 129.2, 128.9, 127.1, 119.7, 118.0, 77.7, 69.4, 43.2, 23.8.; HRMS (ESI) m/z calcd. for $[C_{15}H_{17}ClNO]^+$: 262.0993, found: 262.0999

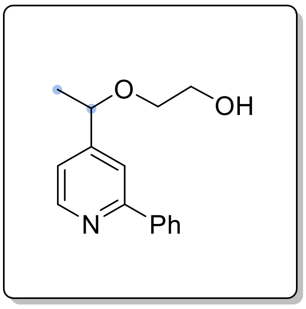

2-(1-(2-phenylpyridin-4-yl)ethoxy)ethan-1-ol (6an). Using Fe(acac)$_3$ 10 mol%. Yield 74% (17.9 mg). Colourless oil. ^{1}H NMR (500 MHz, CDCl$_3$) δ 8.65 (d, *J* = 4.9 Hz, 1H), 7.99 (d, *J* = 7.5 Hz, 2H), 7.67 (s, 1H), 7.48 (t, *J* = 7.5 Hz, 2H), 7.42 (t, *J* = 7.3 Hz, 1H), 7.18 (d, *J* = 4.9 Hz, 1H), 4.51 (q, *J* = 6.6 Hz, 1H), 3.76 (q, *J* = 4.8 Hz, 2H), 3.50 (t, *J* = 4.5 Hz, 2H), 2.20 (t, *J* = 6.0 Hz, 1H), 1.49 (d, *J* = 6.5 Hz, 3H).; ^{13}C NMR (100 MHz, CDCl$_3$) δ 158.1, 153.5, 150.1, 139.4, 129.2, 128.9, 127.1, 119.7, 118.0, 77.6, 70.4, 62.1, 23.8.; HRMS (ESI) m/z calcd. for $[C_{15}H_{18}NO_2]^+$: 244.1332, found: 244.1339

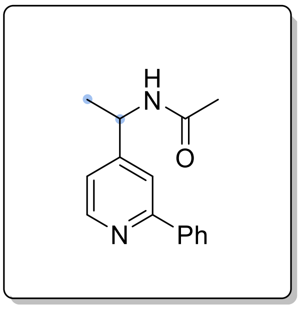

N-(1-(2-phenylpyridin-4-yl)ethyl)acetamide (6ao). Using Fe(acac)$_3$ 10 mol%. 48 h reaction. Yield 52% (12.6 mg). White solid. ^{1}H NMR (500 MHz, CDCl$_3$) δ 8.62 (d, *J* = 5.1 Hz, 1H), 8.00 – 7.91 (m, 2H), 7.65 – 7.59 (m, 1H), 7.46 (t, *J* = 7.4 Hz, 2H), 7.44 – 7.38 (m, 1H), 7.15 (dd, *J* = 5.2, 1.7 Hz, 1H), 5.96 – 5.88 (m, 1H), 5.15 (p, *J* = 7.2 Hz, 1H), 2.02 (s, 3H), 1.50 (d, *J* = 7.0 Hz, 3H).; ^{13}C NMR (100 MHz, CDCl$_3$) δ 169.5, 158.2, 153.1, 150.1, 139.5, 129.2, 128.9, 127.1, 119.6, 118.4, 48.3, 23.5, 21.6.; HRMS

(ESI) m/z calcd. for $[C_{15}H_{17}N_2O]^+$: 241.1335, found: 241.1341

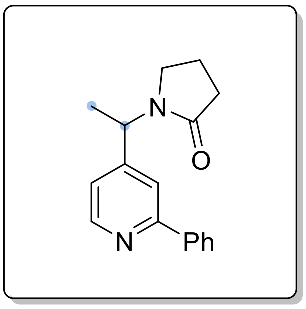

1-(1-(2-phenylpyridin-4-yl)ethyl)pyrrolidin-2-one (6ap). Using $Fe(acac)_3$ 10 mol%. 48 h reaction. Yield 58% (15.5 mg). White solid. ^1H NMR (500 MHz, $CDCl_3$) δ 8.65 (d, J = 5.1 Hz, 1H), 7.98 – 7.93 (m, 2H), 7.63 – 7.59 (m, 1H), 7.51 – 7.45 (m, 2H), 7.44 – 7.39 (m, 1H), 7.14 (dd, J = 5.1, 1.7 Hz, 1H), 5.53 (q, J = 7.1 Hz, 1H), 3.37 (ddd, J = 9.6, 8.2, 6.3 Hz, 1H), 3.05 (ddd, J = 9.6, 8.5, 5.0 Hz, 1H), 2.52 – 2.35 (m, 2H), 2.09 – 1.92 (m, 2H), 1.58 (d, J = 7.1 Hz, 3H).; ^{13}C NMR (100 MHz, $CDCl_3$) δ 175.0, 158.2, 150.2, 150.2, 139.4, 129.2, 128.9, 127.1, 120.4, 119.1, 48.5, 42.5, 31.3, 18.1, 16.0.; HRMS (ESI) m/z calcd. for $[C_{17}H_{19}N_2O]^+$: 267.1492, found: 267.1498

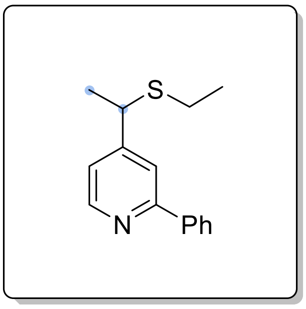

4-(1-(ethylthio)ethyl)-2-phenylpyridine (6aq). Using $Fe(acac)_3$ 10 mol%. 48 h reaction. Yield 59% (14.3 mg). Colourless oil. ^1H NMR (500 MHz, $CDCl_3$) δ 8.63 (d, J = 5.1 Hz, 1H), 8.00 (dd, J = 7.3, 1.8 Hz, 2H), 7.70 (d, J = 1.6 Hz, 1H), 7.48 (t, J = 7.5 Hz, 2H), 7.42 (dd, J = 8.2, 6.1 Hz, 1H), 7.22 (dd, J = 5.1, 1.7 Hz, 1H), 3.99 (q, J = 7.1 Hz, 1H), 2.42 – 2.31 (m, 2H), 1.60 (d, J = 7.0 Hz, 3H), 1.19 (t, J = 7.4 Hz, 3H).; ^{13}C NMR (100 MHz, $CDCl_3$) δ 158.0, 154.2, 150.0, 139.5, 129.2, 128.9, 127.1, 121.0, 119.4, 43.2, 25.4, 22.1, 14.5.; HRMS (ESI) m/z calcd. for $[C_{15}H_{18}NS]^+$: 244.1154, found: 244.1161

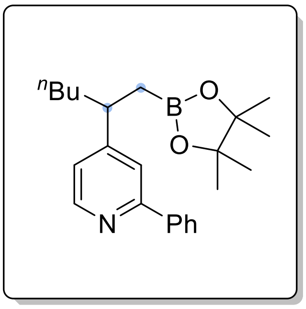

2-phenyl-4-(1-(4,4,5,5-tetramethyl-1,3,2-dioxaborolan-2-yl)hexan-2-yl)pyridine (6ar). Using $Fe(acac)_3$ 10 mol%. Yield 48% (17.5 mg). Colourless oil. ^{1}H NMR (500 MHz, $CDCl_3$) δ 8.55 (d, J = 5.0 Hz, 1H), 8.00 – 7.94 (m, 2H), 7.57 (d, J = 1.6 Hz, 1H), 7.47 (t, J = 7.6 Hz, 2H), 7.42 – 7.37 (m, 1H), 7.08 (dd, J = 5.2, 1.6 Hz, 1H), 2.88 (ddd, J = 15.1, 8.6, 6.5 Hz, 1H), 1.69 – 1.57 (m, 2H), 1.34 – 1.13 (m, 6H), 1.11 (s, 6H), 1.09 (s, 6H), 0.84 (t, J = 7.2 Hz, 3H).; ^{13}C NMR (100 MHz, $CDCl_3$) δ 157.6, 157.4, 149.6, 139.9, 128.8, 128.8, 127.1, 121.6, 120.2, 83.3, 41.4, 38.6, 29.8, 24.8, 24.8, 22.8, 14.1.; ^{11}B NMR (128 MHz, $CDCl_3$) δ 34.3.; HRMS (ESI) m/z calcd. for $[C_{23}H_{33}BNO_2]^+$: 366.2599, found: 366.2604

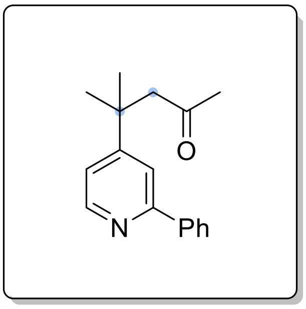

4-methyl-4-(2-phenylpyridin-4-yl)pentan-2-one (6as). Using $Fe(acac)_3$ 10 mol%. Yield 60% (15.1 mg). Colourless oil. ^{1}H NMR (400 MHz, $CDCl_3$) δ 8.61 (dd, J = 5.3, 0.8 Hz, 1H), 8.01 – 7.91 (m, 2H), 7.67 (dd, J = 1.9, 0.8 Hz, 1H), 7.53 – 7.45 (m, 2H), 7.45 – 7.37 (m, 1H), 7.20 (dd, J = 5.2, 1.9 Hz, 1H), 2.84 (s, 2H), 1.97 (s, 3H), 1.47 (s, 6H).; ^{13}C NMR (100 MHz, $CDCl_3$) δ 206.7, 158.4, 157.9, 149.8, 140.0, 129.0, 128.8, 127.2, 119.5, 118.1, 55.8, 37.3, 31.9, 28.5.; HRMS (ESI) m/z calcd. for $[C_{17}H_{20}NO]^+$: 254.1539, found: 254.1546

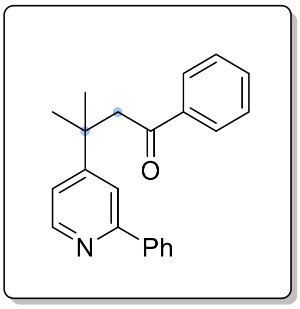

3-methyl-1-phenyl-3-(2-phenylpyridin-4-yl)butan-1-one (6at). Using $Fe(acac)_3$ 10 mol%. 48 h reaction. Yield 47% (14.9 mg). Colourless oil. ^{1}H NMR (500 MHz, $CDCl_3$) δ 8.58 (d, J = 5.3 Hz, 1H), 7.96 – 7.88 (m, 2H), 7.88 – 7.82 (m, 2H), 7.68 (d, J = 1.9 Hz, 1H), 7.52 (t, J = 7.4 Hz, 1H), 7.45 (dd, J = 8.4, 6.7 Hz, 2H), 7.43 – 7.38 (m, 3H), 7.22 (dd, J = 5.2, 1.9 Hz, 1H), 3.40 (s, 2H), 1.55 (s, 6H).; ^{13}C NMR (125 MHz, $CDCl_3$) δ 198.1, 158.8, 157.8, 149.7, 140.1, 137.8, 133.2, 128.9, 128.8, 128.7, 128.1, 127.2, 119.5, 118.2, 50.2, 37.6, 28.9.; HRMS (ESI) m/z calcd. for $[C_{22}H_{22}NO]^+$: 316.1696, found: 316.1702

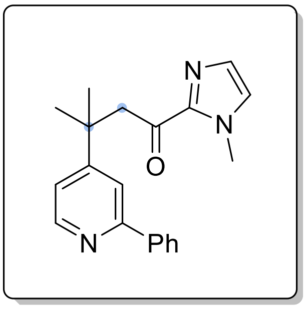

3-methyl-1-(1-methyl-1H-imidazol-2-yl)-3-(2-phenylpyridin-4-yl)butan-1-one (6au). Using $Fe(acac)_3$ 10 mol%. 48 h reaction. Yield 50% (16.1 mg). White solid. ^1H NMR (500 MHz, CD_2Cl_2) δ 8.53 (dd, J = 5.2, 0.8 Hz, 1H), 7.98 (dd, J = 8.4, 1.3 Hz, 2H), 7.78 (dd, J = 1.9, 0.6 Hz, 1H), 7.49 – 7.43 (m, 2H), 7.43 – 7.37 (m, 1H), 7.26 (dd, J = 5.2, 1.9 Hz, 1H), 7.06 (d, J = 0.9 Hz, 1H), 6.98 (d, J = 0.9 Hz, 1H), 3.83 (s, 3H), 3.62 (s, 2H), 1.51 (s, 6H).; ^{13}C NMR (125 MHz, CD_2Cl_2) δ 191.2, 159.2, 157.4, 149.8, 144.1, 140.3, 129.0, 129.0, 128.9, 127.6, 127.3, 120.1, 118.4, 50.1, 38.0, 36.4, 29.2.; HRMS (ESI) m/z calcd. for $[C_{20}H_{22}N_3O]^+$: 320.1757, found: 320.1764

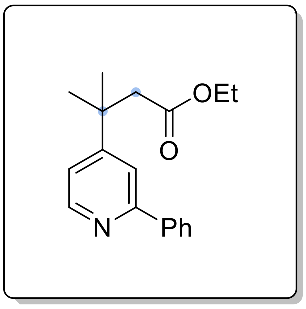

ethyl 3-methyl-3-(2-phenylpyridin-4-yl)butanoate (6av). Using $Fe(acac)_3$ 10 mol%. 48 h reaction. Yield 44% (12.3 mg). Colourless oil. ^1H NMR (500 MHz, $CDCl_3$) δ 8.61 (d, J = 5.2 Hz, 1H), 8.01 – 7.93 (m, 2H), 7.69 (d, J = 1.8 Hz, 1H), 7.53 – 7.43 (m, 2H), 7.43 – 7.37 (m, 1H), 7.22 (dd, J = 5.3, 1.9 Hz, 1H), 3.99 (q, J = 7.1 Hz, 2H), 2.68 (s, 2H), 1.50 (s, 6H), 1.08 (t, J = 7.1 Hz, 3H).; ^{13}C NMR (125 MHz, $CDCl_3$) δ 171.1, 158.1, 157.8, 149.8, 140.0, 129.0, 128.8, 127.2, 119.6, 118.2, 60.3, 47.7, 37.4, 28.6, 14.2.; HRMS (ESI) m/z calcd. for $[C_{18}H_{22}NO_2]^+$: 284.1645, found: 284.1652

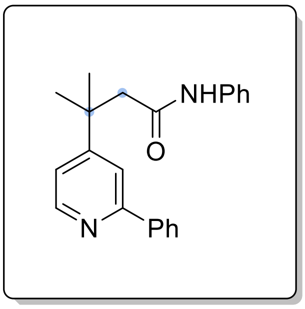

3-methyl-N-phenyl-3-(2-phenylpyridin-4-yl)butanamide (6aw). Using $Fe(acac)_3$ 10 mol%. 48 h reaction. Yield 55% (18.1 mg). Colourless oil. ^1H NMR (500 MHz, CD_2Cl_2) δ 8.61 (d, J = 5.3 Hz, 1H), 7.98 (dd, J = 7.2, 1.9 Hz, 2H), 7.77 (d, J = 2.0 Hz, 1H), 7.56 – 7.37 (m, 3H), 7.32 – 7.19 (m, 5H), 7.05 (t, J = 7.3 Hz, 1H), 6.99 (s, 1H), 2.66 (s, 2H), 1.54 (s, 6H).; ^{13}C NMR (100 MHz, CD_2Cl_2) δ 168.8,

158.6, 158.0, 150.2, 140.1, 138.1, 129.3, 129.2, 129.0, 127.4, 124.6, 120.2, 120.0, 118.3, 51.4, 38.1, 28.4.; HRMS (ESI) m/z calcd. for $[C_{22}H_{23}N_2O]^+$: 331.1805, found: 331.1811

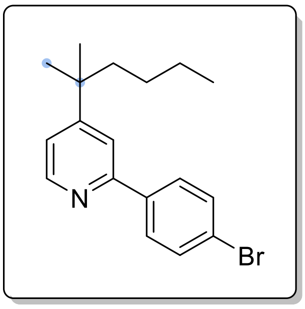

2-(4-bromophenyl)-4-(2-methylhexan-2-yl)pyridine (7a). Yield 61% (20.4 mg). Colourless oil. ^{1}H NMR (600 MHz, $CDCl_3$) δ 8.58 (d, J = 5.3 Hz, 1H), 7.89 – 7.83 (m, 2H), 7.64 – 7.57 (m, 3H), 7.20 (dd, J = 5.3, 1.8 Hz, 1H), 1.68 – 1.61 (m, 2H), 1.33 (s, 6H), 1.24 (h, J = 7.4 Hz, 2H), 1.09 – 1.01 (m, 2H), 0.83 (t, J = 7.3 Hz, 3H).; ^{13}C NMR (100 MHz, $CDCl_3$) δ 160.1, 156.3, 149.7, 139.0, 131.9, 128.8, 123.3, 120.4, 118.2, 43.8, 38.1, 28.4, 27.0, 23.4, 14.1.; HRMS (ESI) m/z calcd. for $[C_{18}H_{23}BrN]^+$: 332.1008, found: 332.1013

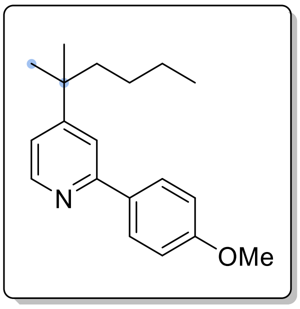

2-(4-methoxyphenyl)-4-(2-methylhexan-2-yl)pyridine (7b). Yield 71% (20.1 mg). Colourless oil. ^{1}H NMR (500 MHz, $CDCl_3$) δ 8.55 (dd, J = 5.3, 0.8 Hz, 1H), 7.97 – 7.90 (m, 2H), 7.59 (dd, J = 1.8, 0.9 Hz, 1H), 7.13 (dd, J = 5.3, 1.8 Hz, 1H), 7.03 – 6.97 (m, 2H), 3.87 (s, 3H), 1.66 – 1.60 (m, 2H), 1.33 (s, 6H), 1.24 (h, J = 7.2 Hz, 2H), 1.11 – 1.01 (m, 2H), 0.83 (t, J = 7.2 Hz, 3H).; ^{13}C NMR (125 MHz, $CDCl_3$) δ 160.4, 159.6, 157.2, 149.5, 132.8, 128.4, 119.4, 117.7, 114.2, 55.5, 43.8, 38.0, 28.4, 27.0, 23.4, 14.1.; HRMS (ESI) m/z calcd. for $[C_{19}H_{26}NO]^+$: 284.2009, found: 284.2013

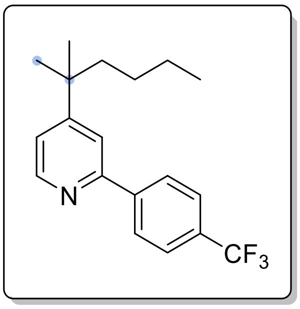

4-(2-methylhexan-2-yl)-2-(4-(trifluoromethyl)phenyl)pyridine (7c). 48 h reaction. Yield 74% (23.7 mg). Colourless oil. ^{1}H NMR (400 MHz, $CDCl_3$) δ 8.62 (d, *J* = 5.3 Hz, 1H), 8.10 (d, *J* = 8.1 Hz, 2H), 7.73 (d, *J* = 8.3 Hz, 2H), 7.68 (d, *J* = 1.8 Hz, 1H), 7.24 (dd, *J* = 5.3, 1.8 Hz, 1H), 1.70 – 1.61 (m, 2H), 1.35 (s, 6H), 1.30 – 1.18 (m, 2H), 1.12 – 1.00 (m, 2H), 0.84 (t, *J* = 7.3 Hz, 3H).; ^{13}C NMR (125 MHz, $CDCl_3$) δ 160.2, 156.0, 149.9, 143.5, 130.7 (q, *J* = 32.6 Hz), 127.5, 125.8 (q, *J* = 3.7 Hz), 124.4 (q, *J* = 272.1 Hz), 120.9, 118.7, 43.8, 38.2, 28.4, 27.0, 23.4, 14.1.; ^{19}F NMR (376 MHz, $CDCl_3$) δ -62.5.; HRMS (ESI) m/z calcd. for $[C_{19}H_{23}F_3N]^+$: 322.1777, found: 322.1783

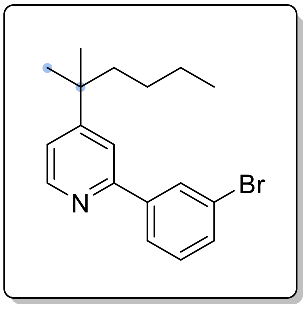

2-(3-bromophenyl)-4-(2-methylhexan-2-yl)pyridine (7d). Yield 75% (24.9 mg). Colourless oil. ^{1}H NMR (500 MHz, $CDCl_3$) δ 8.59 (dd, *J* = 5.2, 0.7 Hz, 1H), 8.14 (t, *J* = 1.9 Hz, 1H), 7.90 (dt, *J* = 7.8, 1.3 Hz, 1H), 7.61 (dd, *J* = 1.9, 0.8 Hz, 1H), 7.53 (ddd, *J* = 7.9, 1.9, 0.8 Hz, 1H), 7.34 (t, *J* = 7.8 Hz, 1H), 7.21 (dd, *J* = 5.2, 1.8 Hz, 1H), 1.68 – 1.61 (m, 2H), 1.34 (s, 6H), 1.28 – 1.19 (m, 2H), 1.10 – 1.00 (m, 2H), 0.83 (t, *J* = 7.3 Hz, 3H).; ^{13}C NMR (125 MHz, $CDCl_3$) δ 160.1, 156.0, 149.7, 142.2, 131.8, 130.3, 130.2, 125.7, 123.1, 120.6, 118.4, 43.8, 38.1, 28.4, 27.0, 23.4, 14.1.; HRMS (ESI) m/z calcd. for $[C_{18}H_{23}BrN]^+$: 332.1008, found: 332.1013

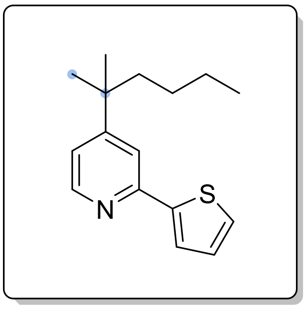

4-(2-methylhexan-2-yl)-2-(thiophen-2-yl)pyridine (7e). Yield 62% (16.1 mg). Colourless oil. ^{1}H NMR (600 MHz, $CDCl_3$) δ 8.47 (dd, *J* = 5.3, 0.8 Hz, 1H), 7.62 – 7.57 (m, 2H), 7.38 (dd, *J* = 5.1, 1.1 Hz, 1H), 7.14 – 7.08 (m, 2H), 1.65 – 1.60 (m, 2H), 1.32 (s, 6H), 1.23 (h, *J* = 7.3 Hz, 2H), 1.09 – 1.01 (m, 2H), 0.83 (t, *J* = 7.3 Hz, 3H).; ^{13}C NMR (100 MHz, $CDCl_3$) δ 159.9, 152.5, 149.4, 145.4, 128.1, 127.4, 124.4, 120.1, 116.5, 43.7, 38.0, 28.3, 27.0, 23.4, 14.1.; HRMS (ESI) m/z calcd. for $[C_{16}H_{22}NS]^+$: 260.1467, found: 260.1474

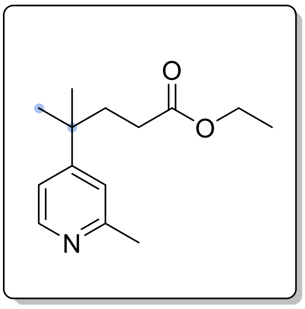

ethyl 4-methyl-4-(2-methylpyridin-4-yl)pentanoate (7f). Using Fe(acac)$_3$ 10 mol%. Yield 81% (19.1 mg). Colourless oil. ^{1}H NMR (500 MHz, CDCl$_3$) δ 8.39 (d, *J* = 5.3 Hz, 1H), 7.07 (d, *J* = 1.9 Hz, 1H), 7.05 – 7.00 (m, 1H), 4.04 (q, *J* = 7.1 Hz, 2H), 2.53 (s, 3H), 2.05 – 1.92 (m, 4H), 1.28 (s, 6H), 1.19 (t, *J* = 7.1 Hz, 3H).; ^{13}C NMR (125 MHz, CDCl$_3$) δ 173.7, 158.3, 158.0, 149.0, 120.9, 118.5, 60.5, 38.2, 37.4, 30.1, 28.0, 24.6, 14.3.; HRMS (ESI) m/z calcd. for $[C_{14}H_{22}NO_2]^+$: 236.1645, found: 236.1653

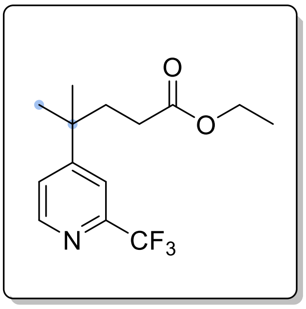

ethyl 4-methyl-4-(2-(trifluoromethyl)pyridin-4-yl)pentanoate (7g). Using Fe(acac)$_3$ 10 mol%. Yield 62% (17.8 mg). Colourless oil. ^{1}H NMR (500 MHz, CDCl$_3$) δ 8.65 (d, *J* = 5.2 Hz, 1H), 7.61 (dd, *J* = 1.9, 0.7 Hz, 1H), 7.43 (dd, *J* = 5.2, 1.9 Hz, 1H), 4.05 (q, *J* = 7.1 Hz, 2H), 2.10 – 1.95 (m, 4H), 1.35 (s, 6H), 1.21 (t, *J* = 7.1 Hz, 3H).; ^{13}C NMR (100 MHz, CDCl$_3$) δ 173.2, 159.8, 150.2, 148.6 (q, *J* = 34.0 Hz), 124.0, 121.8 (q, *J* = 274.4 Hz), 118.0 (q, *J* = 2.9 Hz), 60.7, 38.2, 38.0, 30.0, 27.9, 14.2.; ^{19}F NMR (376 MHz, CDCl$_3$) δ -67.84.; HRMS (ESI) m/z calcd. for $[C_{14}H_{19}F_3NO_2]^+$: 290.1362, found: 290.1367

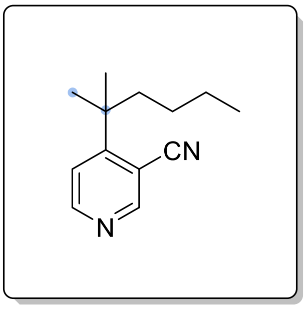

4-(2-methylhexan-2-yl)nicotinonitrile (7h). Yield 62% (12.5 mg). Colourless oil. ^{1}H NMR (400 MHz, CDCl$_3$) δ 8.82 (s, 1H), 8.67 (s, 1H), 7.34 (d, *J* = 5.4 Hz, 1H), 1.98 – 1.89 (m, 2H), 1.46 (s, 6H), 1.32 – 1.21 (m, 2H), 1.03 – 0.91 (m, 2H), 0.84 (t, *J* = 7.3 Hz, 3H).; ^{13}C NMR (125 MHz, CDCl$_3$) δ 161.8, 155.3, 152.9, 122.2, 118.3, 108.7, 41.2, 39.3, 27.8, 27.1, 23.2, 14.0.; HRMS (ESI) m/z calcd. for $[C_{13}H_{19}N_2]^+$: 203.1543, found: 203.1547

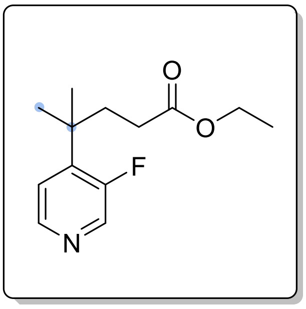

ethyl 4-(3-fluoropyridin-4-yl)-4-methylpentanoate (7i). Using Fe(acac)$_3$ 10 mol%. Yield 53% (12.8 mg). Colourless oil. ^{1}H NMR (500 MHz, CDCl$_3$) δ 8.34 (d, *J* = 4.1 Hz, 1H), 8.31 (dd, *J* = 5.1, 1.0 Hz, 1H), 7.15 (dd, *J* = 7.2, 5.1 Hz, 1H), 4.04 (q, *J* = 7.1 Hz, 2H), 2.14 – 2.08 (m, 2H), 2.08 – 2.02 (m, 2H), 1.37 (d, *J* = 1.1 Hz, 6H), 1.20 (t, *J* = 7.1 Hz, 3H).; ^{13}C NMR (125 MHz, CDCl$_3$) δ 173.4, 158.8 (d, *J* = 257.0 Hz), 146.0 (d, *J* = 4.9 Hz), 143.2 (d, *J* = 9.2 Hz), 139.0 (d, *J* = 27.9 Hz), 122.7 (d, *J* = 2.9 Hz), 60.6, 37.4 (d, *J* = 2.8 Hz), 36.0 (d, *J* = 3.9 Hz), 30.4, 27.3 (d, *J* = 2.8 Hz), 14.3.; ^{19}F NMR (471 MHz, CDCl$_3$) δ -124.3.; HRMS (ESI) m/z calcd. for $[C_{13}H_{19}FNO_2]^+$: 240.1394, found: 240.1401

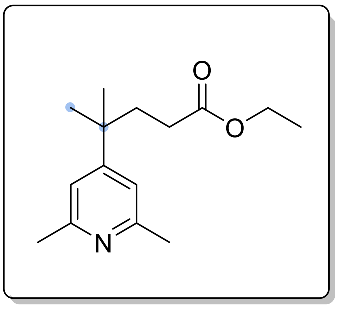

ethyl 4-(2,6-dimethylpyridin-4-yl)-4-methylpentanoate (7j). Using Fe(acac)$_3$ 10 mol%. Yield 82% (20.4 mg). Colourless oil. ^{1}H NMR (500 MHz, CDCl$_3$) δ 6.87 (s, 2H), 4.05 (q, *J* = 7.1 Hz, 2H), 2.49 (s, 6H), 2.05 – 2.00 (m, 2H), 1.95 – 1.90 (m, 2H), 1.26 (s, 6H), 1.20 (t, *J* = 7.1 Hz, 3H).; ^{13}C NMR (125 MHz, CDCl$_3$) δ 173.8, 158.0, 157.7, 117.8, 60.5, 38.3, 37.2, 30.2, 28.1, 24.7, 14.3.; HRMS (ESI) m/z calcd. for $[C_{15}H_{24}NO_2]^+$: 250.1802, found: 250.1806

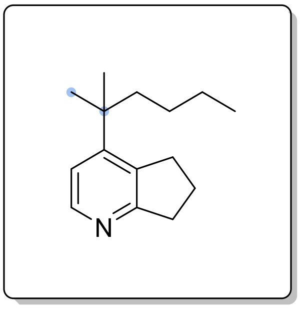

4-(2-methylhexan-2-yl)-6,7-dihydro-5H-cyclopenta[b]pyridine (7k). Yield 66% (14.4 mg). Colourless oil. ^{1}H NMR (400 MHz, CDCl$_3$) ^{1}H NMR (400 MHz, CDCl$_3$) δ 8.23 (d, *J* = 5.3 Hz, 1H), 6.96 (d, *J* = 5.3 Hz,1H), 3.07 (t, *J* = 7.3 Hz, 2H), 2.98 (t, *J* = 7.7 Hz, 2H), 2.06 (p, *J* = 7.5 Hz, 2H), 1.70

– 1.61 (m, 2H), 1.31 (s, 6H), 1.22 (h, *J* = 7.4 Hz, 2H), 1.03 – 0.91 (m, 2H), 0.82 (t, *J* = 7.4 Hz, 3H).; ^{13}C NMR (100 MHz, $CDCl_3$) δ 165.9, 154.9, 147.2, 134.8, 119.5, 41.4, 39.0, 34.0, 32.9, 28.2, 27.2, 23.4, 23.3, 14.1.; HRMS (ESI) m/z calcd. for $[C_{15}H_{24}N]^+$: 218.1903, found: 218.1908

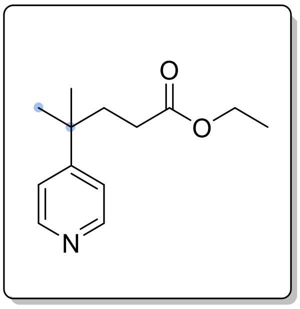

ethyl 4-methyl-4-(pyridin-4-yl)pentanoate (7l). Using Fe(acac)$_3$ 10 mol%. Yield 74% (16.3 mg). Colourless oil. ^{1}H NMR (500 MHz, $CDCl_3$) δ 8.52 (s, 2H), 7.22 (s, 2H), 4.05 (q, *J* = 7.1 Hz, 2H), 2.07 – 2.00 (m, 2H), 2.00 – 1.94 (m, 2H), 1.30 (s, 6H), 1.20 (t, *J* = 7.1 Hz, 3H).; ^{13}C NMR (125 MHz, $CDCl_3$) δ 173.6, 157.5, 149.9, 121.4, 60.5, 38.3, 37.5, 30.1, 28.0, 14.3.; HRMS (ESI) m/z calcd. for $[C_{13}H_{20}NO_2]^+$: 222.1489, found: 222.1495

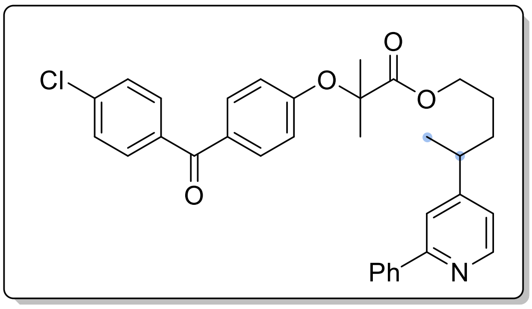

4-(2-phenylpyridin-4-yl)pentyl 2-(4-(4-chlorobenzoyl)phenoxy)-2-methylpropanoate (8a). Using Fe(acac)$_3$ 10 mol%. Yield 72% (39.1 mg). White solid. ^{1}H NMR (500 MHz, $CDCl_3$) ^{1}H NMR (500 MHz, Chloroform-*d*) δ 8.55 (dd, *J* = 5.0, 0.8 Hz, 1H), 8.03 – 7.89 (m, 2H), 7.76 – 7.69 (m, 2H), 7.69 – 7.65 (m, 2H), 7.51 – 7.35 (m, 6H), 6.97 (dd, *J* = 5.1, 1.6 Hz, 1H), 6.84 (d, *J* = 8.8 Hz, 2H), 4.20 – 4.05 (m, 2H), 2.67 (h, *J* = 6.7 Hz, 1H), 1.66 (s, 3H), 1.66 (s, 3H), 1.60 – 1.44 (m, 4H), 1.21 (d, *J* = 6.9 Hz, 3H).; ^{13}C NMR (100 MHz, $CDCl_3$) δ 194.2, 173.9, 159.8, 157.8, 156.5, 149.9, 139.6, 138.6, 136.4, 132.2, 131.3, 130.5, 129.0, 128.8, 128.7, 127.1, 121.0, 119.5, 117.2, 79.5, 65.6, 39.4, 33.7, 26.6, 25.7, 25.4, 21.7.; HRMS (ESI) m/z calcd. for $[C_{33}H_{33}ClNO_4]^+$: 542.2093, found: 542.2098

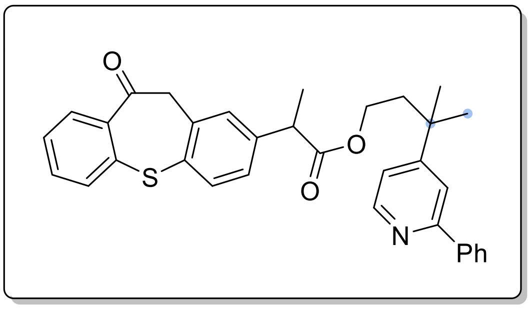

3-methyl-3-(2-phenylpyridin-4-yl)butyl 2-(10-oxo-10,11-dihydrodibenzo[b,f]thiepin-2-yl)propanoate (8b). Yield 68% (35.4 mg). White solid. ^{1}H NMR (400 MHz, $CDCl_3$) δ 8.59 (d, *J* = 5.2 Hz, 1H), 8.19 (dd, *J* = 8.0, 1.6 Hz, 1H), 8.03 – 7.88 (m, 2H), 7.62 (d, *J* = 1.8 Hz, 1H), 7.61 – 7.52 (m, 2H), 7.48 (dd, *J* = 8.2, 6.5 Hz, 2H), 7.44 – 7.38 (m, 2H), 7.35 – 7.27 (m, 2H), 7.14 (dd, *J* = 5.3, 1.8 Hz, 1H), 7.04 (dd, *J* = 8.0, 2.0 Hz, 1H), 4.33 (t, *J* = 12.1 Hz, 2H), 4.05 – 3.84 (m, 2H), 3.50 (q, *J* = 7.1 Hz, 1H), 1.98 (t, *J* = 7.2 Hz, 2H), 1.37 (d, *J* = 7.2 Hz, 3H), 1.33 (s, 6H).; ^{13}C NMR (100 MHz, $CDCl_3$) δ 191.4, 173.9, 158.3, 157.9, 149.9, 142.7, 140.3, 139.9, 138.0, 136.3, 133.3, 132.6, 131.6, 131.6, 131.0, 129.0, 128.9, 128.7, 127.2, 127.0, 126.4, 119.7, 118.2, 62.2, 51.1, 45.2, 41.6, 36.9, 28.6, 28.4, 18.5.; HRMS (ESI) m/z calcd. for $[C_{33}H_{32}NO_3S]^+$: 522.2097, found: 522.2102

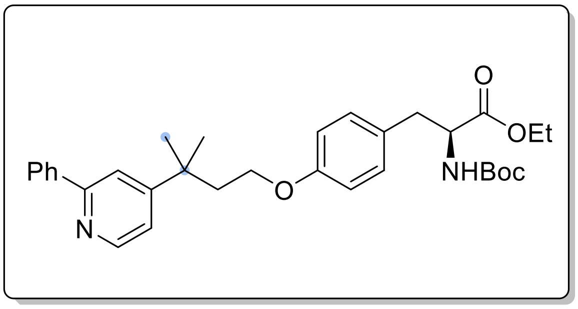

ethyl (S)-2-((tert-butoxycarbonyl)amino)-3-(4-(3-methyl-3-(2-phenylpyridin-4-yl)butoxy)phenyl)propanoate (8c). Yield 66% (35.1 mg). White solid. ^{1}H NMR (500 MHz, $CDCl_3$) δ 8.62 (d, *J* = 5.3 Hz, 1H), 7.96 (d, *J* = 7.5 Hz, 2H), 7.73 – 7.60 (m, 1H), 7.47 (t, *J* = 7.5 Hz, 2H), 7.41 (t, *J* = 7.3 Hz, 1H), 7.23 (dd, *J* = 5.3, 1.8 Hz, 1H), 6.97 (d, *J* = 8.1 Hz, 2H), 6.66 (d, *J* = 8.1 Hz, 2H), 4.94 (d, *J* = 8.3 Hz, 1H), 4.55 – 4.22 (m, 1H), 4.13 (q, *J* = 7.1 Hz, 2H), 3.80 (t, *J* = 7.0 Hz, 2H), 2.98 (qd, *J* = 14.6, 14.1, 5.7 Hz, 2H), 2.20 (t, *J* = 7.0 Hz, 2H), 1.45 (s, 6H), 1.40 (s, 10H), 1.21 (t, *J* = 7.1 Hz, 3H).; ^{13}C NMR (125 MHz, $CDCl_3$) δ 172.0, 158.6, 157.9, 157.8, 155.2, 149.9, 139.9, 130.4, 129.0, 128.8, 128.2, 127.2, 119.8, 118.2, 114.5, 79.9, 64.8, 61.4, 54.6, 42.4, 37.5, 37.1, 28.8, 28.4, 14.3.; HRMS (ESI) m/z calcd. for $[C_{32}H_{41}N_2O_5]^+$: 533.3010, found: 533.3015

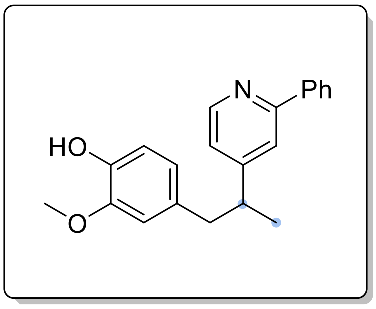

2-methoxy-4-(2-(2-phenylpyridin-4-yl)propyl)phenol (8d). Yield 58% (18.4 mg). Colourless oil. ^{1}H NMR (600 MHz, $CDCl_3$) δ 8.57 (dd, *J* = 5.1, 0.8 Hz, 1H), 7.92 (dd, *J* = 8.3, 1.3 Hz, 2H), 7.51 – 7.44 (m, 3H), 7.44 – 7.36 (m, 1H), 7.03 (dd, *J* = 5.1, 1.7 Hz, 1H), 6.80 (d, *J* = 8.0 Hz, 1H), 6.61 (dd, *J* = 8.0, 1.9 Hz, 1H), 6.45 (d, *J* = 1.9 Hz, 1H), 5.58 (s, 1H), 3.74 (s, 3H), 3.02 (h, *J* = 7.1 Hz, 1H), 2.86 (dd, *J* = 13.6, 7.1 Hz, 1H), 2.80 (dd, *J* = 13.6, 7.5 Hz, 1H), 1.32 (d, *J* = 6.9 Hz, 3H).; ^{13}C NMR (100 MHz, $CDCl_3$) δ 157.6, 156.6, 149.7, 146.3, 144.2, 139.8, 131.7, 129.0, 128.8, 127.1, 122.0, 121.3, 120.0, 114.3, 111.8, 55.9, 44.1, 42.0, 20.5.; HRMS (ESI) m/z calcd. for $[C_{21}H_{22}NO_2]^+$: 320.1645, found: 320.1651

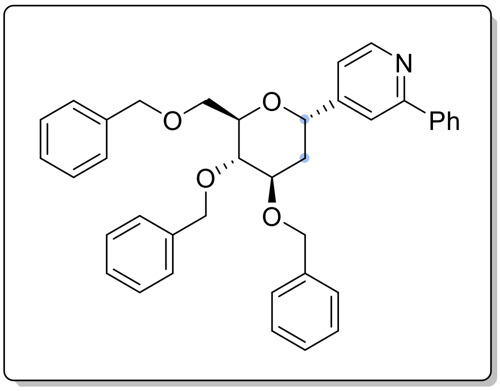

4-((2S,4R,5S,6R)-4,5-bis(benzyloxy)-6-((benzyloxy)methyl)tetrahydro-2H-pyran-2-yl)-2-phenyl-pyridine (8e). Using $Fe(acac)_3$ 10 mol%. Yield 52% (29.9 mg). White solid. ^{1}H NMR (400 MHz, $CDCl_3$) δ 8.60 (d, *J* = 5.1 Hz, 1H), 7.93 (dd, *J* = 7.9, 1.8 Hz, 2H), 7.72 (d, *J* = 1.5 Hz, 1H), 7.48 – 7.40 (m, 4H), 7.39 – 7.27 (m, 12H), 7.20 (dd, *J* = 7.5, 2.0 Hz, 2H), 7.07 (dt, *J* = 5.1, 1.2 Hz, 1H), 5.10 (t, *J* = 4.7 Hz, 1H), 4.79 (d, *J* = 11.2 Hz, 1H), 4.73 – 4.61 (m, 3H), 4.61 – 4.48 (m, 2H), 3.85 – 3.71 (m, 3H), 3.70 – 3.56 (m, 2H), 2.50 (dt, *J* = 14.1, 4.1 Hz, 1H), 2.11 (ddd, *J* = 14.0, 9.1, 5.1 Hz, 1H).; ^{13}C NMR (100 MHz, $CDCl_3$) δ 158.1, 150.1, 150.0, 139.4, 138.3, 138.2, 138.1, 129.0, 128.7, 128.6, 128.4, 128.4, 127.9, 127.9, 127.9, 127.9, 127.7, 127.7, 127.1, 119.6, 118.2, 77.0, 76.1, 74.1, 73.9, 73.5, 72.0, 70.9, 69.0, 32.4.; HRMS (ESI) m/z calcd. for $[C_{38}H_{38}NO_4]^+$: 572.2795, found: 572.2804

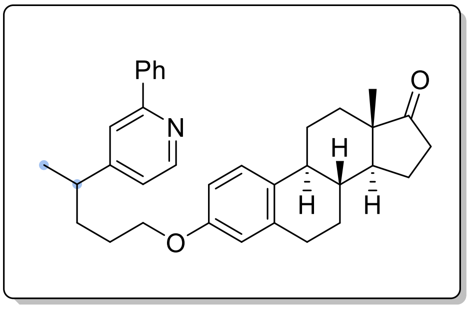

(8R,9S,13S,14S,17S)-13-methyl-17-((4-(2-phenylpyridin-4-yl)pentyl)oxy)-7,8,9,11,12,13,14,15,-16,17-decahydro-6H-cyclopenta[a]phenanthren-3-ol (8f). Using $Fe(acac)_3$ 10 mol%. 48h reaction. Yield 68% (33.7 mg). Colourless oil. ^{1}H NMR (500 MHz, $CDCl_3$) δ 8.59 (d, J = 5.1 Hz, 1H), 8.09 – 7.81 (m, 2H), 7.56 (d, J = 1.6 Hz, 1H), 7.47 (dd, J = 8.3, 6.6 Hz, 2H), 7.45 – 7.35 (m, 1H), 7.22 – 7.13 (m, 1H), 7.09 (dd, J = 5.1, 1.6 Hz, 1H), 6.68 (dd, J = 8.6, 2.7 Hz, 1H), 6.61 (d, J = 2.7 Hz, 1H), 3.91 (tt, J = 6.1, 1.5 Hz, 2H), 2.97 – 2.72 (m, 3H), 2.58 – 2.43 (m, 1H), 2.42 – 2.32 (m, 1H), 2.28 – 2.20 (m, 1H), 2.14 (dt, J = 19.1, 8.9 Hz, 1H), 2.09 – 1.90 (m, 3H), 1.85 – 1.79 (m, 2H), 1.79 – 1.73 (m, 2H), 1.71 – 1.55 (m, 3H), 1.55 – 1.50 (m, 1H), 1.49 – 1.46 (m, 1H), 1.46 – 1.37 (m, 1H), 1.33 (d, J = 6.9 Hz, 3H), 0.90 (s, 3H).; ^{13}C NMR (150 MHz, $CDCl_3$) δ 221.0, 157.8, 157.1, 157.0, 149.9, 139.8, 137.9, 132.2, 129.0, 128.8, 127.1, 126.4, 121.2, 119.7, 114.7, 112.2, 67.7, 50.5, 48.1, 44.1, 39.6, 38.5, 36.0, 34.1, 31.7, 29.8, 27.5, 26.7, 26.0, 21.8, 21.7.; HRMS (ESI) m/z calcd. for $[C_{34}H_{40}NO_2]^+$: 494.3054, found: 494.3060

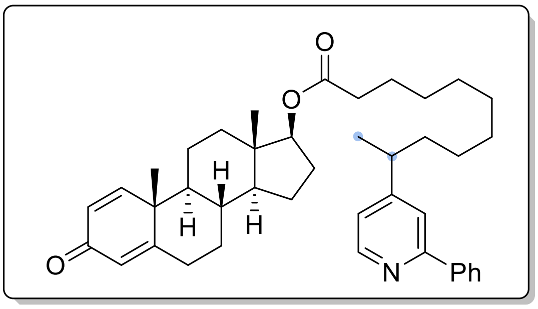

(8R,9S,10R,13S,14S,17S)-10,13-dimethyl-3-oxo-6,7,8,9,10,11,12,13,14,15,16,17-dodecahydro-3H-cyclopenta[a]phenanthren-17-yl 10-(2-phenylpyridin-4-yl)undecanoate (8g). 48 h reaction. Yield 59% (35.9 mg). White solid. ^{1}H NMR (500 MHz, $CDCl_3$) δ 8.57 (d, J = 5.0 Hz, 1H), 8.06 – 7.87 (m, 2H), 7.55 – 7.51 (m, 1H), 7.47 (dd, J = 8.3, 6.7 Hz, 2H), 7.43 – 7.36 (m, 1H), 7.05 (dd, J = 5.1, 1.6 Hz, 1H), 7.03 (d, J = 10.2 Hz, 1H), 6.22 (dd, J = 10.1, 1.9 Hz, 1H), 6.07 (s, 1H), 4.58 (dd, J = 9.2, 7.8 Hz, 1H), 2.73 (h, J = 7.0 Hz, 1H), 2.46 (tdd, J = 13.4, 5.1, 1.6 Hz, 1H), 2.36 (ddd, J = 13.3, 4.4, 2.5 Hz, 1H), 2.27 (t, J = 7.5 Hz, 2H), 2.16 (dtd, J = 13.6, 9.4, 6.2 Hz, 1H), 1.98 – 1.87 (m, 1H), 1.81 – 1.70 (m, 2H), 1.68 (s, 3H), 1.66 – 1.54 (m, 6H), 1.53 – 1.44 (m, 1H), 1.41 – 1.32 (m, 1H), 1.28 (d, J = 7.0 Hz, 3H), 1.27 – 1.23 (m, 7H), 1.22 (s, 3H), 1.21 – 1.13 (m, 2H), 1.09 – 0.97 (m, 3H), 0.84 (s, 3H).; ^{13}C NMR (100 MHz, $CDCl_3$) δ 186.4, 173.9, 168.9, 157.7, 155.8, 149.7, 139.9, 128.9, 128.8, 127.7, 127.1, 124.1, 121.2, 119.7, 82.2, 52.3, 50.0, 43.6, 42.9, 39.9, 37.8, 36.7, 35.5, 34.6, 33.2, 32.9, 29.7, 29.4, 29.3, 29.2, 27.6, 27.6, 25.2, 23.8, 22.5, 21.7, 18.9, 12.3.; HRMS (ESI) m/z calcd. for $[C_{41}H_{54}NO_3]^+$: 608.4098, found: 608.4104

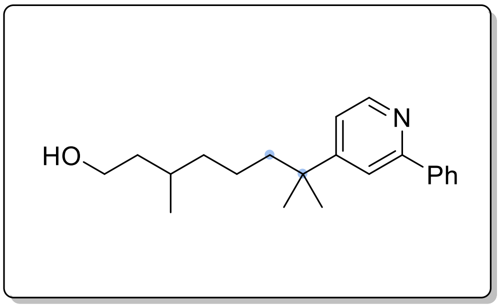

3,7-dimethyl-7-(2-phenylpyridin-4-yl)octan-1-ol (8h). Using $Fe(acac)_3$ 10 mol%. Yield 69% (21.5 mg). White solid. ^1H NMR (500 MHz, $CDCl_3$) δ 8.59 (dd, J = 5.3, 0.8 Hz, 1H), 8.00 – 7.94 (m, 2H), 7.65 (dd, J = 1.9, 0.8 Hz, 1H), 7.54 – 7.45 (m, 2H), 7.44 – 7.37 (m, 1H), 7.18 (dd, J = 5.2, 1.8 Hz, 1H), 3.62 (qt, J = 10.5, 6.2 Hz, 2H), 1.67 – 1.57 (m, 2H), 1.56 – 1.43 (m, 2H), 1.34 (s, 6H), 1.32 – 1.26 (m, 1H), 1.23 (m, 1H), 1.16 – 1.01 (m, 3H), 0.81 (d, J = 6.6 Hz, 3H).; ^{13}C NMR (100 MHz, $CDCl_3$) δ 159.7, 157.6, 149.6, 140.1, 128.9, 128.8, 127.2, 120.0, 118.4, 61.2, 44.2, 40.0, 38.1, 37.8, 29.4, 28.4, 22.1, 19.6.; HRMS (ESI) m/z calcd. for $[C_{21}H_{30}NO]^+$: 312.2322, found: 312.2329

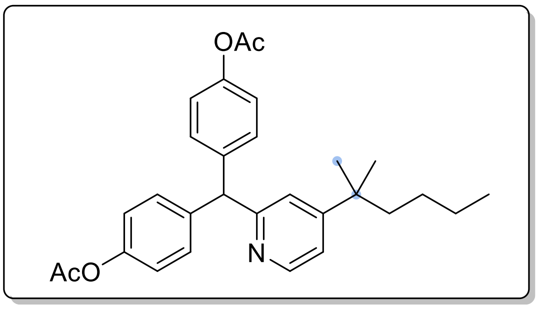

((4-(2-methylhexan-2-yl)pyridin-2-yl)methylene)bis(4,1-phenylene) diacetate (8i). Using $Fe(acac)_3$ 10 mol%. 48 h reaction. Yield 67% (30.8 mg). White solid. ^1H NMR (500 MHz, $CDCl_3$) δ 8.49 (d, J = 5.3 Hz, 1H), 7.22 – 7.16 (m, 4H), 7.10 (dd, J = 5.3, 1.8 Hz, 1H), 7.06 (d, J = 1.9 Hz, 1H), 7.01 (d, J = 6.6 Hz, 4H), 5.64 (s, 1H), 2.27 (s, 6H), 1.55 – 1.48 (m, 2H), 1.22 (s, 6H), 1.18 (q, J = 7.4 Hz, 2H), 1.01 – 0.91 (m, 2H), 0.80 (t, J = 7.3 Hz, 3H).; ^{13}C NMR (125 MHz, $CDCl_3$) δ 169.5, 162.1, 160.0, 149.4, 149.3, 140.3, 130.3, 121.6, 121.5, 119.5, 58.1, 43.6, 37.9, 28.2, 26.9, 23.3, 21.2, 14.1.; HRMS (ESI) m/z calcd. for $[C_{29}H_{34}NO_4]^+$: 460.2482, found: 460.2489

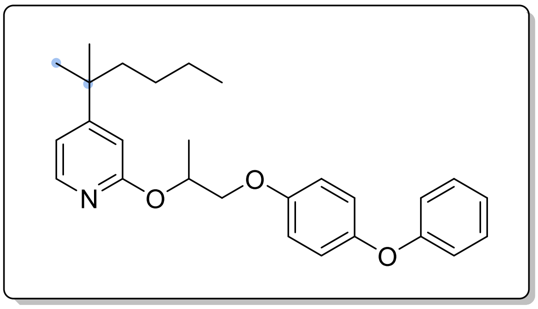

4-(2-methylhexan-2-yl)-2-((1-(4-phenoxyphenoxy)propan-2-yl)oxy)pyridine (8j). Using $Fe(acac)_3$ 10 mol%. Using methanol solvent. Yield 48% (19.9 mg). White solid. ^1H NMR (500 MHz, $CDCl_3$) δ 8.05 (d, J = 5.5 Hz, 1H), 7.33 – 7.25 (m, 2H), 7.04 (tt, J = 7.4, 1.2 Hz, 1H), 7.00 – 6.90 (m, 6H), 6.83

(dd, J = 5.5, 1.7 Hz, 1H), 6.69 (d, J = 1.6 Hz, 1H), 5.58 (dtd, J = 11.4, 6.4, 4.9 Hz, 1H), 4.15 (ddd, J = 51.4, 9.9, 5.0 Hz, 2H), 1.60 – 1.53 (m, 2H), 1.50 (d, J = 6.4 Hz, 3H), 1.25 (s, 6H), 1.24 – 1.19 (m, 2H), 1.09 – 0.99 (m, 2H), 0.84 (t, J = 7.3 Hz, 3H).; ^{13}C NMR (125 MHz, $CDCl_3$) δ 163.7, 162.3, 158.6, 155.4, 150.4, 146.3, 129.7, 122.5, 120.9, 117.7, 115.9, 115.3, 109.0, 71.2, 69.3, 43.6, 37.9, 28.3, 26.9, 23.4, 17.2, 14.1.; HRMS (ESI) m/z calcd. for $[C_{27}H_{34}NO_3]^+$: 420.2533, found: 420.2539

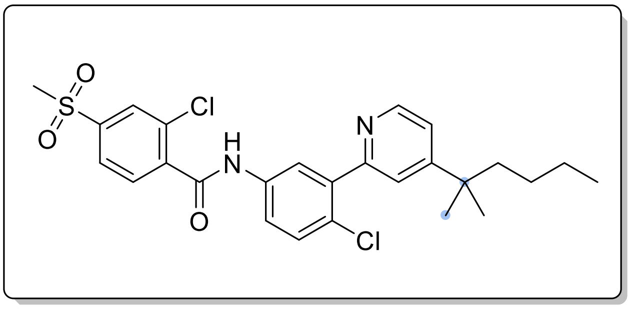

2-chloro-N-(4-chloro-3-(4-(2-methylhexan-2-yl)pyridin-2-yl)phenyl)-4-(methylsulfonyl)benzamide (8k). Using Fe(acac)$_3$ 10 mol%. Yield 61% (31.7 mg). Colourless oil. ^{1}H NMR (400 MHz, $CDCl_3$) δ 10.21 (s, 1H), 8.16 (d, J = 5.3 Hz, 1H), 8.00 (dd, J = 8.8, 2.6 Hz, 1H), 7.86 (d, J = 1.7 Hz, 1H), 7.73 – 7.63 (m, 2H), 7.57 (d, J = 1.8 Hz, 1H), 7.53 (d, J = 7.9 Hz, 1H), 7.46 (d, J = 8.7 Hz, 1H), 7.10 (dd, J = 5.4, 1.9 Hz, 1H), 2.98 (s, 3H), 1.62 – 1.52 (m, 2H), 1.28 (s, 6H), 1.26 – 1.18 (m, 2H), 1.04 (tdd, J = 8.1, 6.7, 5.3, 3.4 Hz, 2H), 0.83 (t, J = 7.3 Hz, 3H).; ^{13}C NMR (100 MHz, $CDCl_3$) δ 164.1, 160.2, 155.4, 148.3, 142.7, 141.0, 139.0, 137.2, 132.4, 131.0, 130.1, 128.9, 127.5, 125.8, 123.7, 123.1, 121.9, 120.6, 44.5, 43.5, 38.2, 28.2, 26.9, 23.3, 14.1.; HRMS (ESI) m/z calcd. for $[C_{15}H_{18}NO]^+$: 519.1270, found: 519.1277

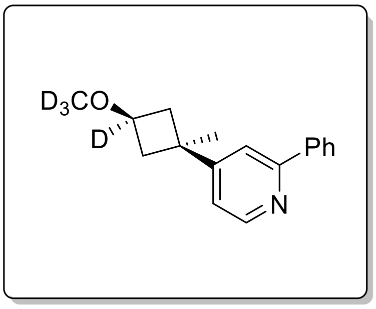

4-((1s,3s)-3-(methoxy-d3)-1-methylcyclobutyl-3-d)-2-phenylpyridine (3b-D$_4$). Yield 68% (17.5 mg). Colourless oil. ^{1}H NMR (500 MHz, $CDCl_3$) δ 8.60 (d, J = 5.1 Hz, 1H), 7.97 (d, J = 7.3 Hz, 2H), 7.50 (s, 1H), 7.47 (t, J = 7.5 Hz, 2H), 7.44 – 7.38 (m, 1H), 7.04 (d, J = 5.2 Hz, 1H), 2.62 – 2.47 (m, 2H), 2.39 – 2.21 (m, 2H), 1.46 (s, 3H).; ^{13}C NMR (150 MHz, $CDCl_3$) δ 161.3, 159.7, 157.8, 157.8, 149.8, 149.8, 139.8, 129.0, 129.0, 128.9, 128.8, 127.2, 119.8, 119.0, 118.2, 117.4, 70.5 – 68.8 (m), 41.9, 41.4, 37.0, 34.8, 32.7, 30.2.; HRMS (ESI) m/z calcd. for $[C_{17}H_{16}D_4NO]^+$: 258.1790, found: 258.1796

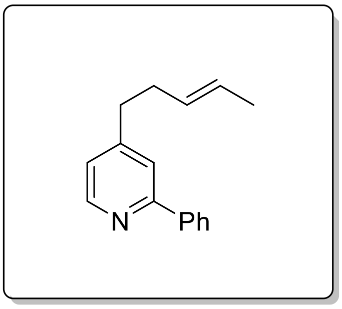

4-(pent-3-en-1-yl)-2-phenylpyridine (6ax). Mixture product. Yield 44% (9.8 mg). Colourless oil. ^{1}H NMR (500 MHz, $CDCl_3$) δ 8.57 (d, *J* = 4.9 Hz, 1H), 8.04 – 7.93 (m, 2H), 7.54 (s, 1H), 7.47 (t, *J* = 7.5 Hz, 2H), 7.41 (t, *J* = 7.3 Hz, 1H), 7.06 (dd, *J* = 5.0, 1.5 Hz, 1H), 5.47 (d, *J* = 5.6 Hz, 2H), 2.73 (t, *J* = 7.8 Hz, 2H), 2.41 – 2.31 (m, 2H), 1.65 (d, *J* = 4.5 Hz, 3H).; ^{13}C NMR (100 MHz, $CDCl_3$) δ 157.6, 151.9, 149.6, 139.8, 129.8, 128.9, 128.8, 127.1, 126.5, 125.5, 122.6, 121.0, 35.7, 35.4, 33.4, 27.8, 18.1, 12.9.; HRMS (ESI) m/z calcd. for $[C_{16}H_{18}N]^+$: 224.1434, found: 224.1440

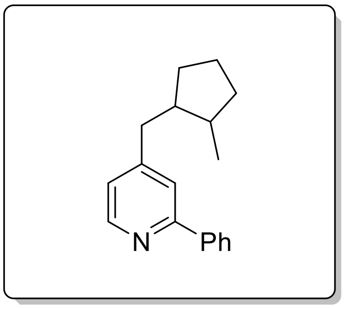

4-((2-methylcyclopentyl)methyl)-2-phenylpyridine (6ay). Mixture product. Yield 44% (11.1 mg). Colourless oil. ^{1}H NMR (500 MHz, $CDCl_3$) δ 8.56 (d, *J* = 5.0 Hz, 1H), 8.04 – 7.79 (m, 2H), 7.55 (s, 1H), 7.47 (t, *J* = 7.5 Hz, 2H), 7.40 (t, *J* = 7.3 Hz, 1H), 7.07 (dd, *J* = 5.1, 1.6 Hz, 1H), 2.77 (dd, *J* = 13.6, 6.0 Hz, 1H), 2.48 (dd, *J* = 13.6, 9.5 Hz, 1H), 2.24 – 2.00 (m, 2H), 1.87 – 1.66 (m, 2H), 1.65 – 1.44 (m, 2H), 1.41 – 1.15 (m, 2H), 0.92 (d, *J* = 7.0 Hz, 3H).; ^{13}C NMR (100 MHz, $CDCl_3$) δ 157.5, 152.5, 152.0, 149.6, 139.8, 128.9, 128.8, 127.1, 123.0, 122.9, 121.4, 121.3, 48.7, 44.4, 40.6, 40.6, 36.5, 36.4, 34.6, 33.4, 32.3, 29.6, 23.3, 22.5, 19.5, 15.4.; HRMS (ESI) m/z calcd. for $[C_{18}H_{22}N]^+$: 252.1747, found: 252.1753

Appendix Ⅰ

Sepctral Copies of ^{1}H, ^{13}C, ^{11}B, and ^{19}F NMr Data

Obtained in this study

4-((1s,3s)-3-ethoxy-1-methylcyclobutyl)-2-phenylpyridine (3a).

500 MHz, ^{1}H NMR in $CDCl_3$

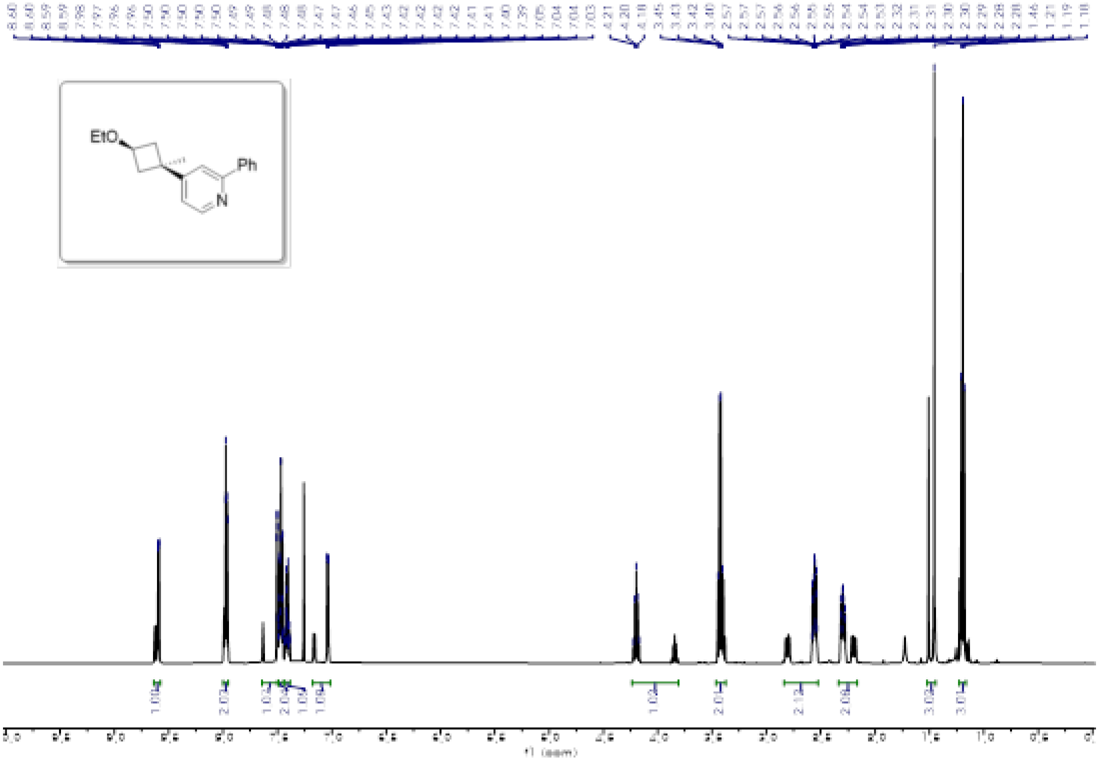

125 MHz, ^{13}C NMR in $CDCl_3$

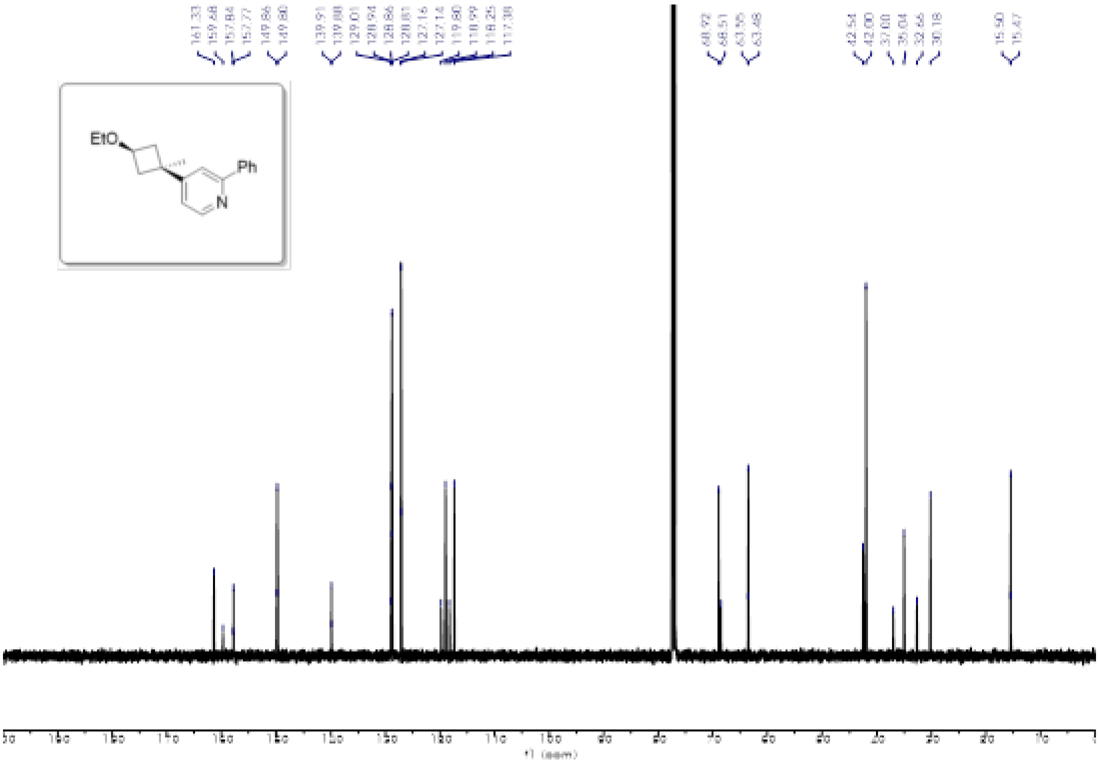

4-((1s,3s)-3-methoxy-1-methylcyclobutyl)-2-phenylpyridine (3b).

400 MHz, ^{1}H NMR in CDCl$_3$

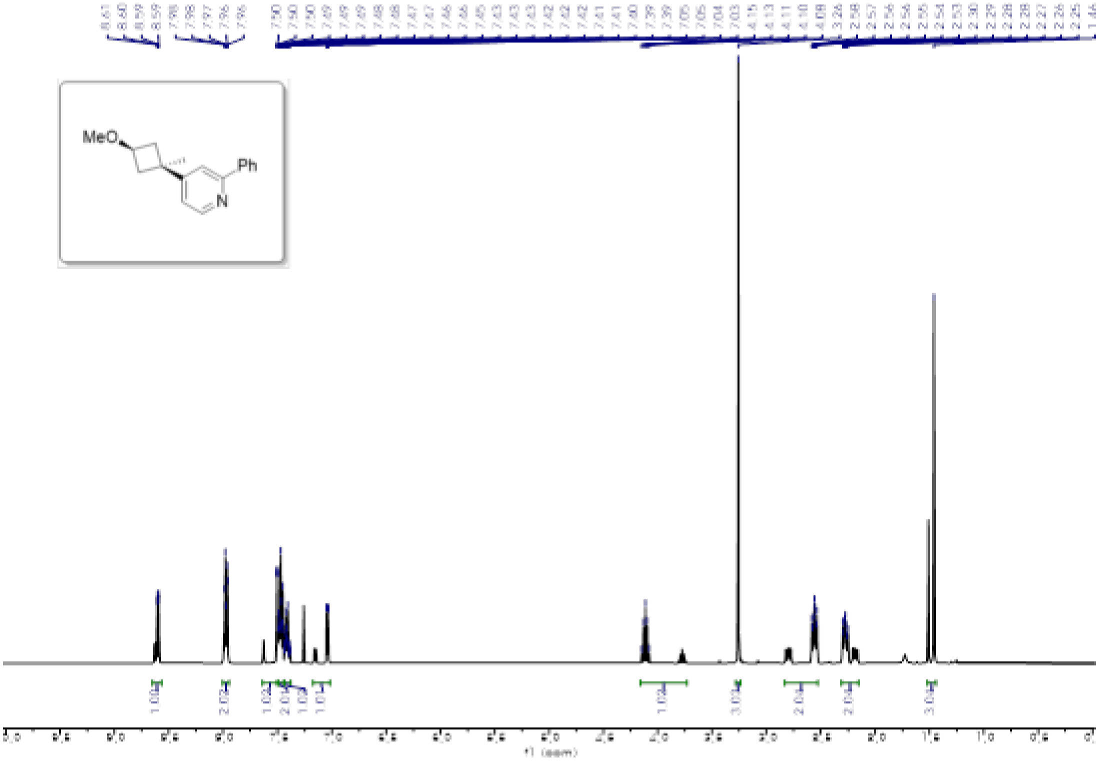

100 MHz, ^{13}C NMR in CDCl$_3$

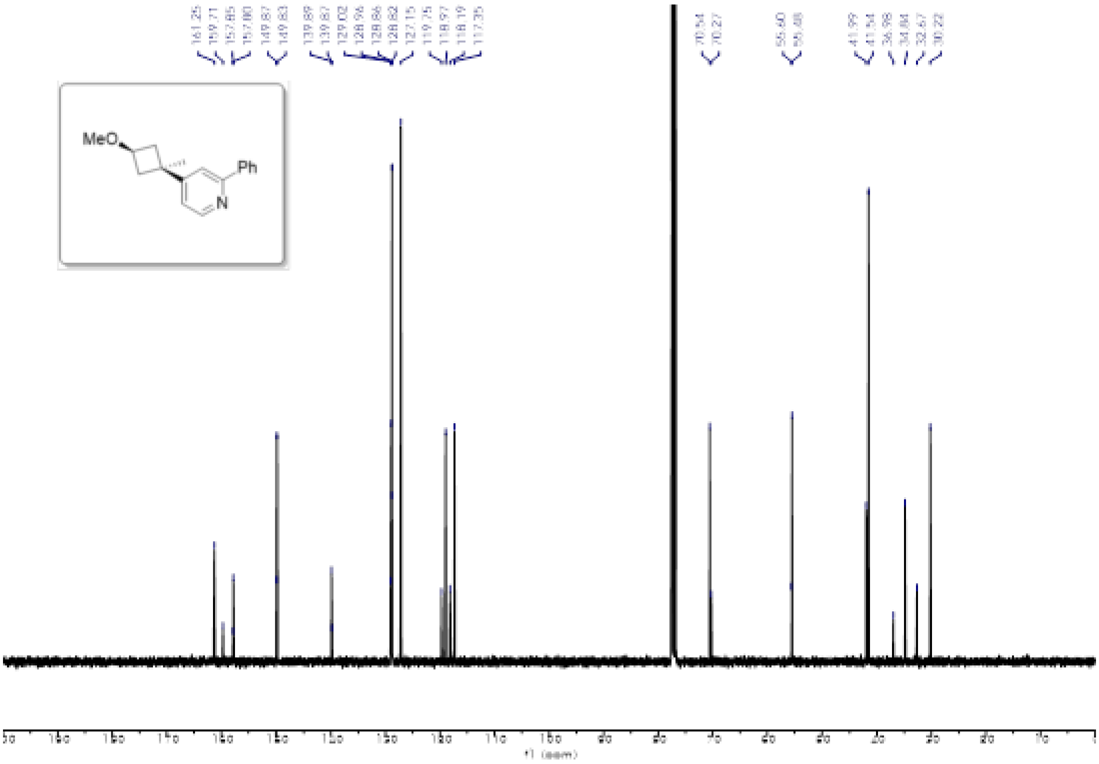

4-((1s,3s)-3-isopropoxy-1-methylcyclobutyl)-2-phenylpyridine (3c).

500 MHz, ^{1}H NMR in $CDCl_3$

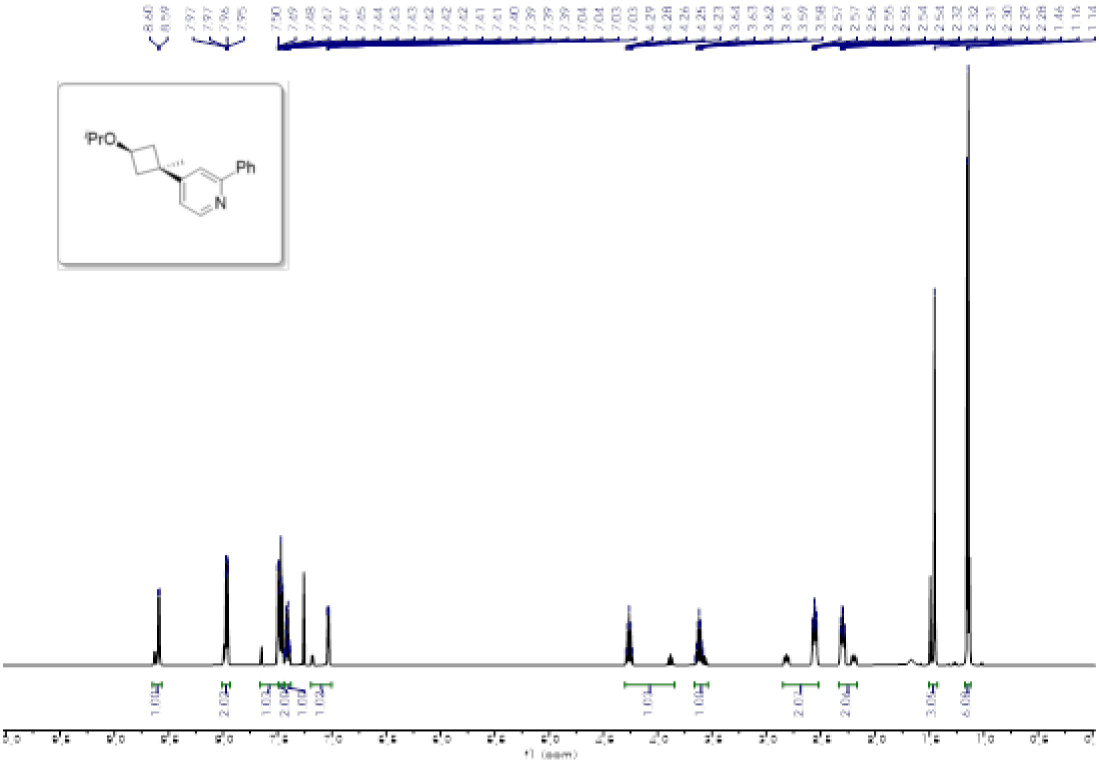

100 MHz, ^{13}C NMR in $CDCl_3$

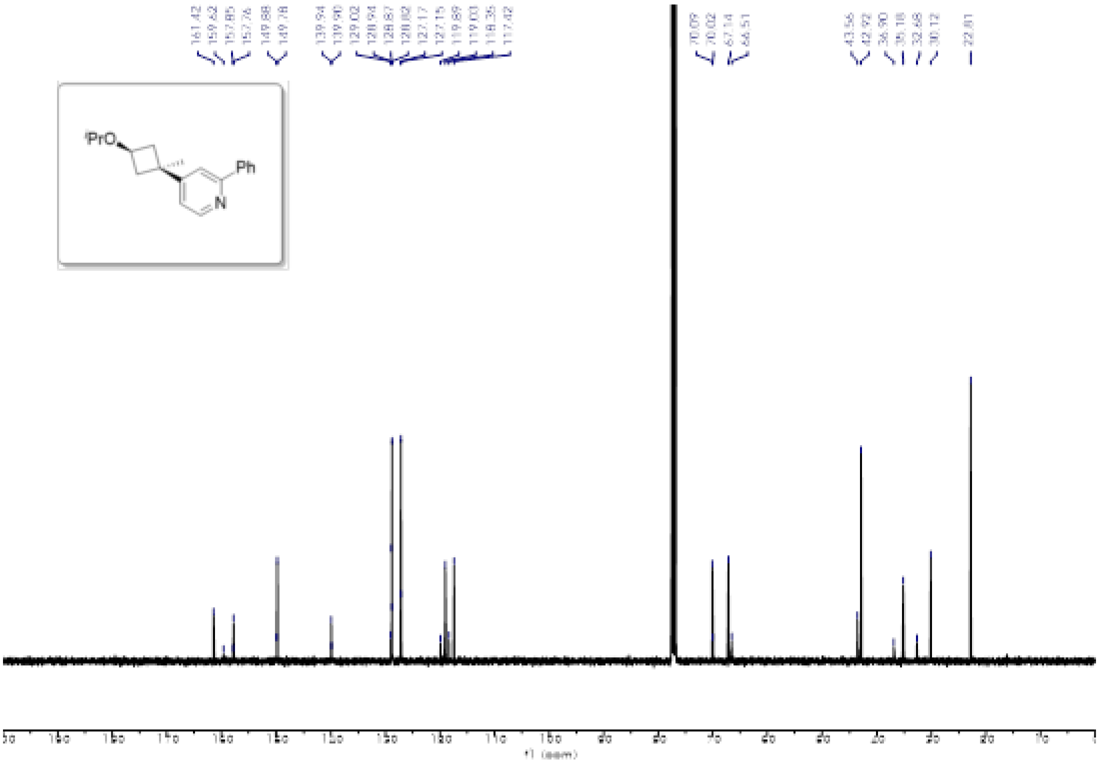

4-((1s,3s)-3-(cyclohexyloxy)-1-methylcyclobutyl)-2-phenylpyridine (3d).

500 MHz, ^{1}H NMR in $CDCl_3$

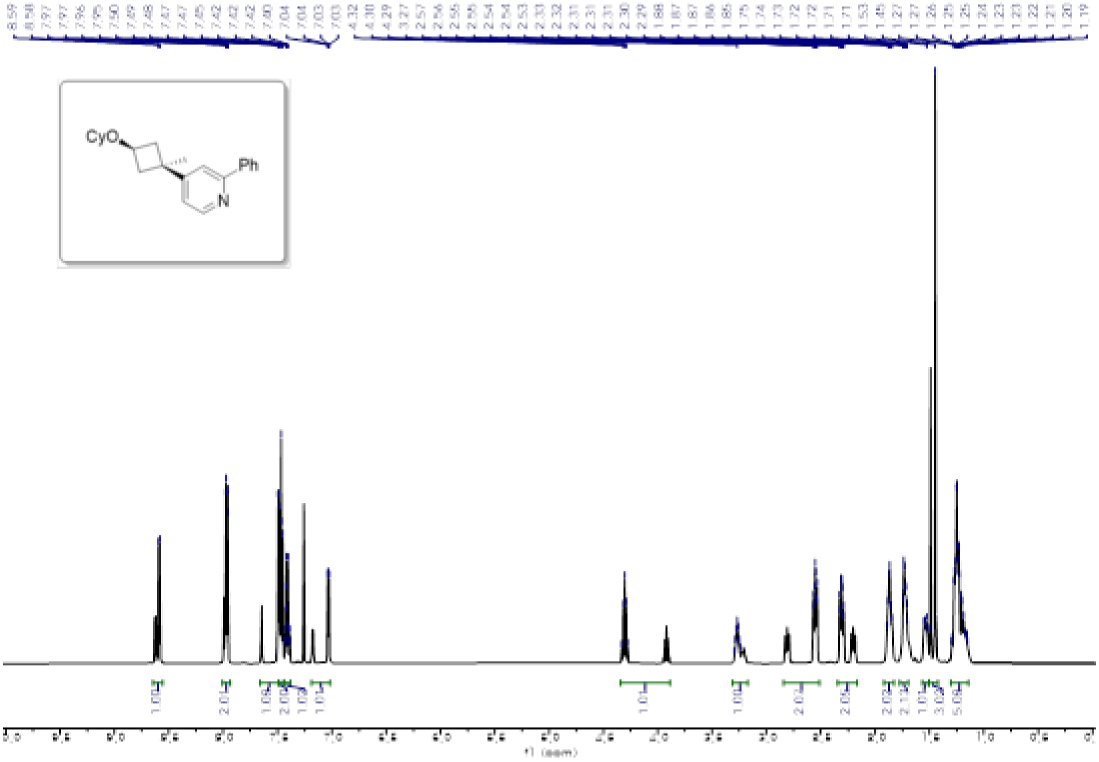

100 MHz, ^{13}C NMR in $CDCl_3$

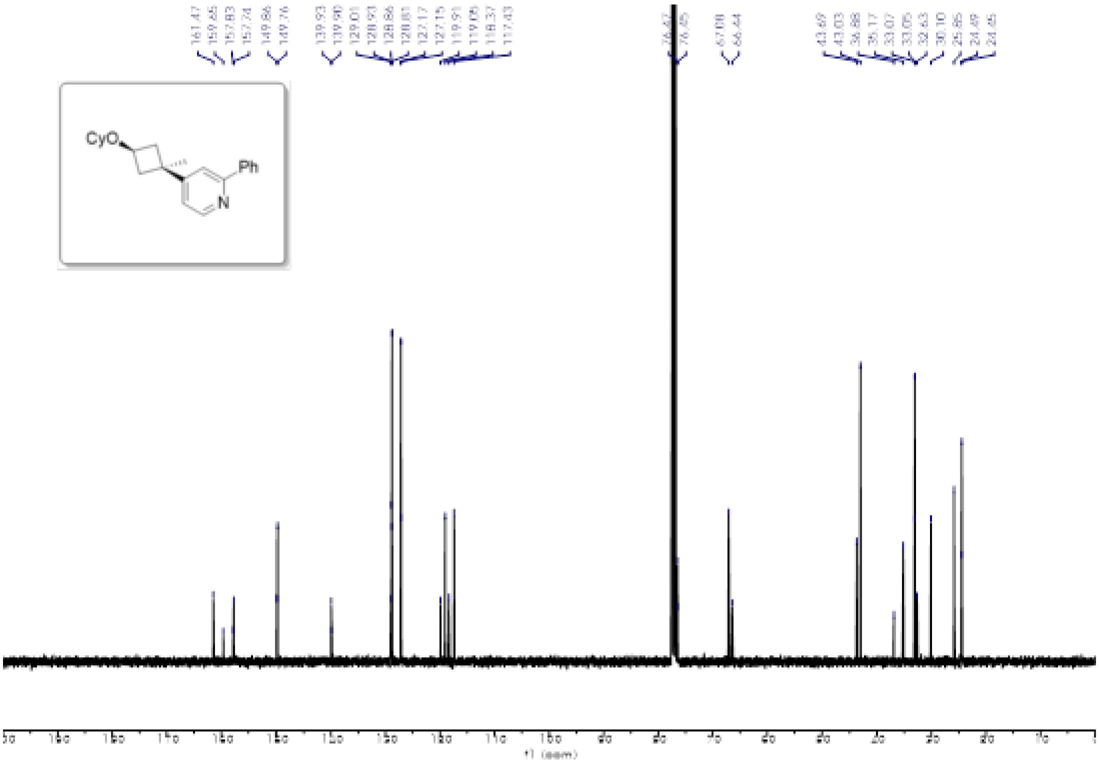

4-((1s,3s)-3-(tert-butoxy)-1-methylcyclobutyl)-2-phenylpyridine (3e).

500 MHz, ^{1}H NMR in CDCl$_3$

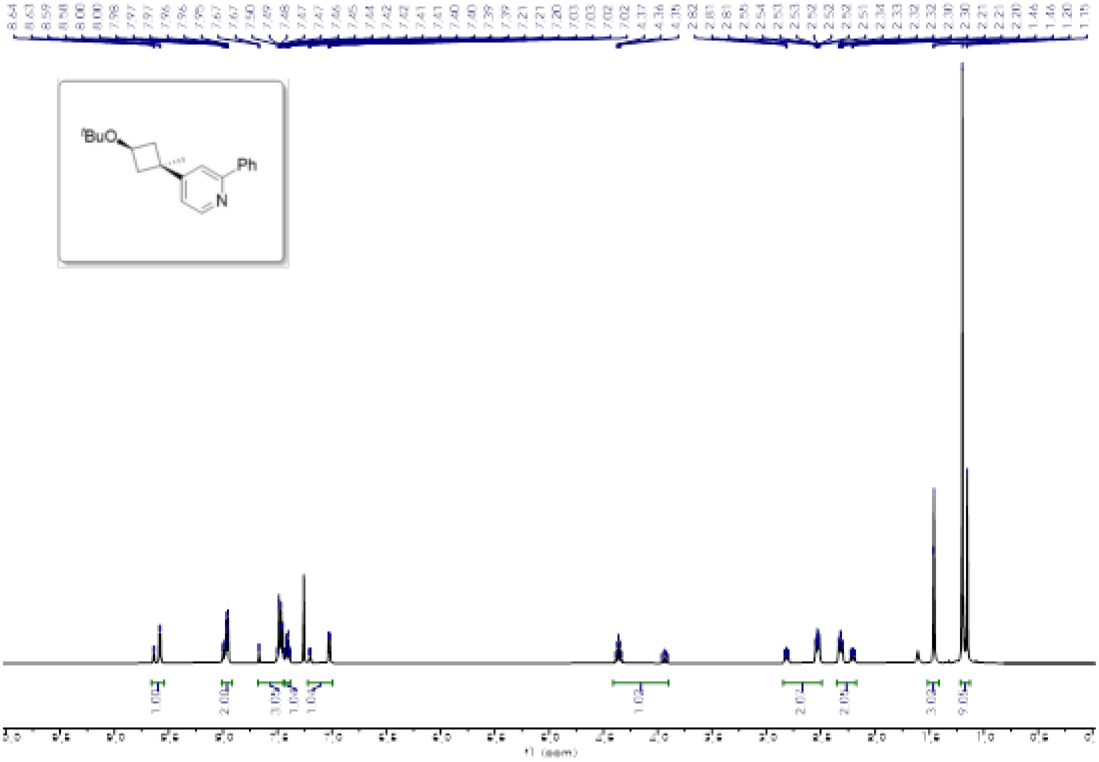

125 MHz, ^{13}C NMR in CDCl$_3$

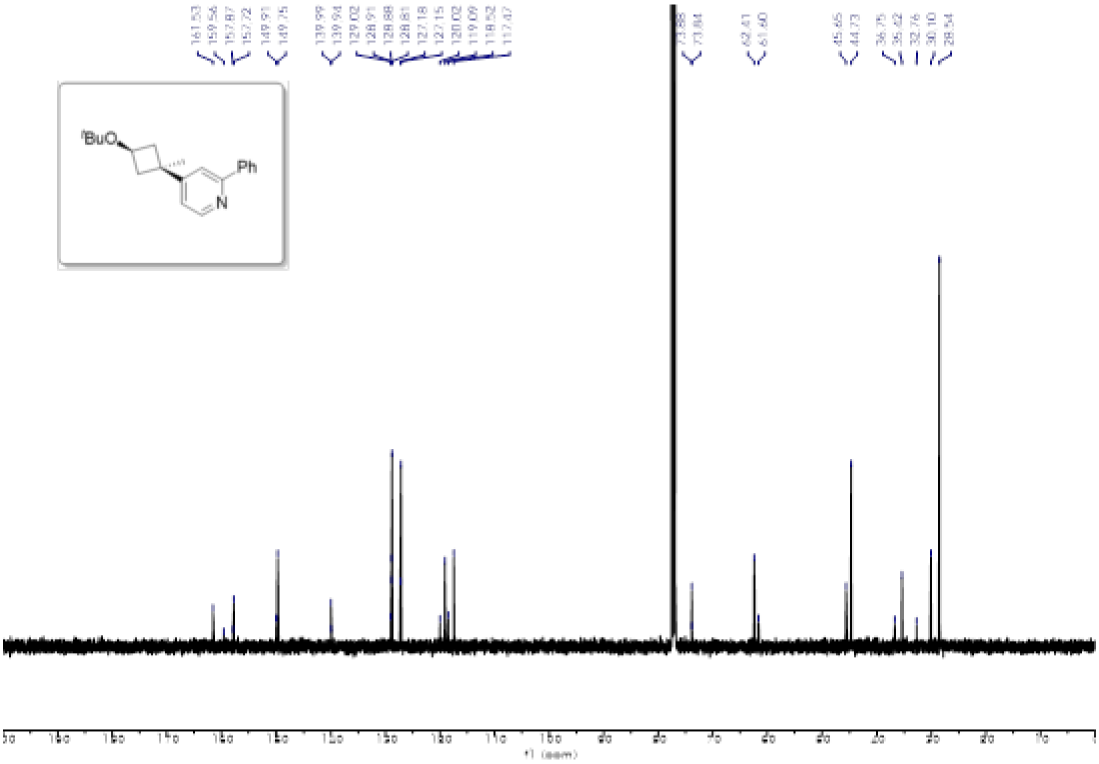

4-((1s,3s)-1-methyl-3-(tert-pentyloxy)cyclobutyl)-2-phenylpyridine (3f).

500 MHz, ^{1}H NMR in CDCl$_3$

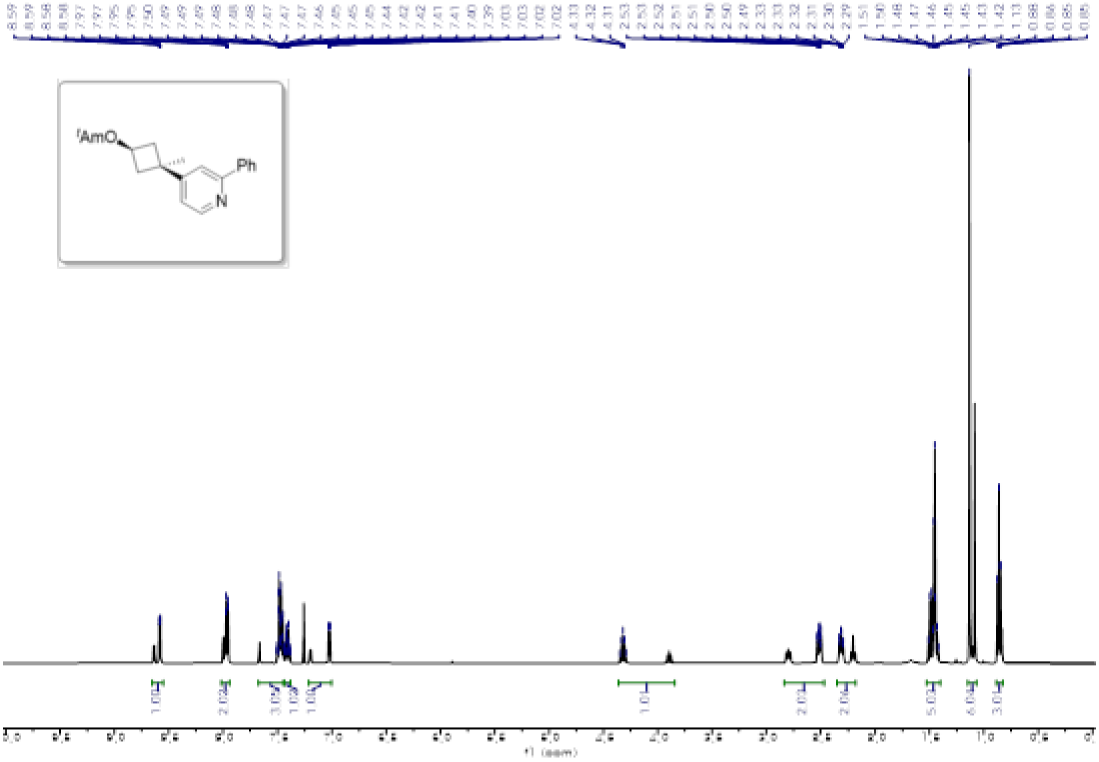

100 MHz, ^{13}C NMR in CDCl$_3$

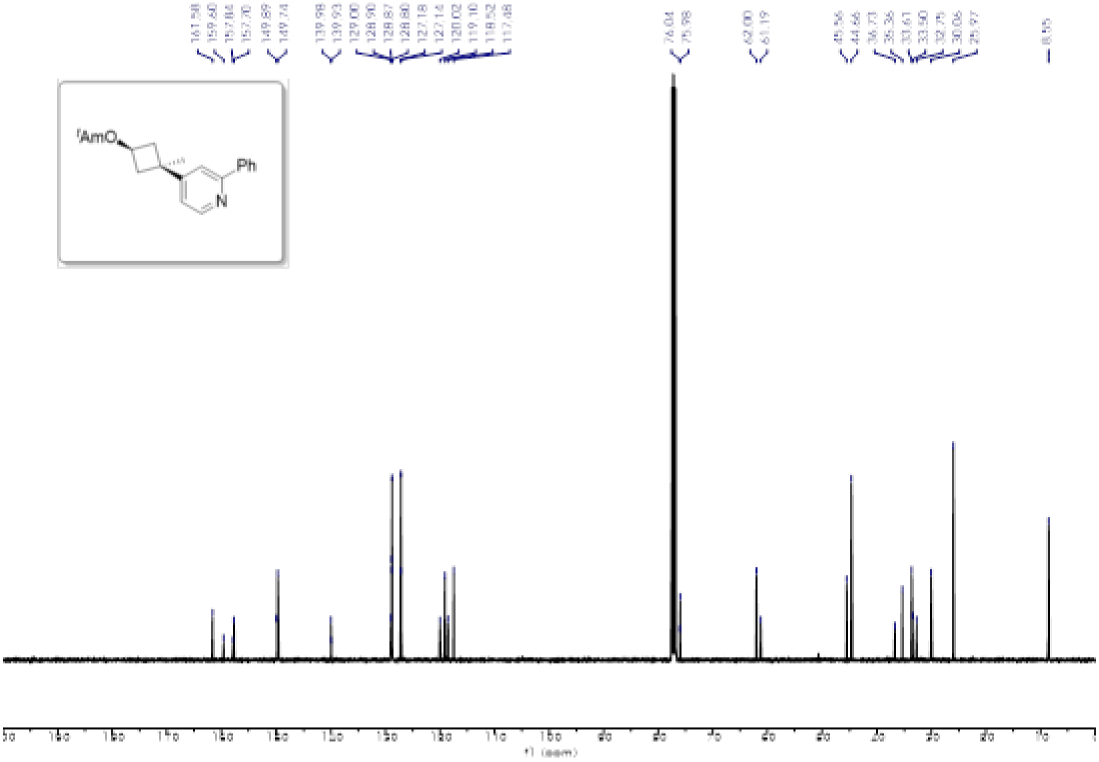

4-((1s,3s)-1-methyl-3-(2-propoxyethoxy)cyclobutyl)-2-phenylpyridine (3g).

500 MHz, ^{1}H NMR in $CDCl_3$

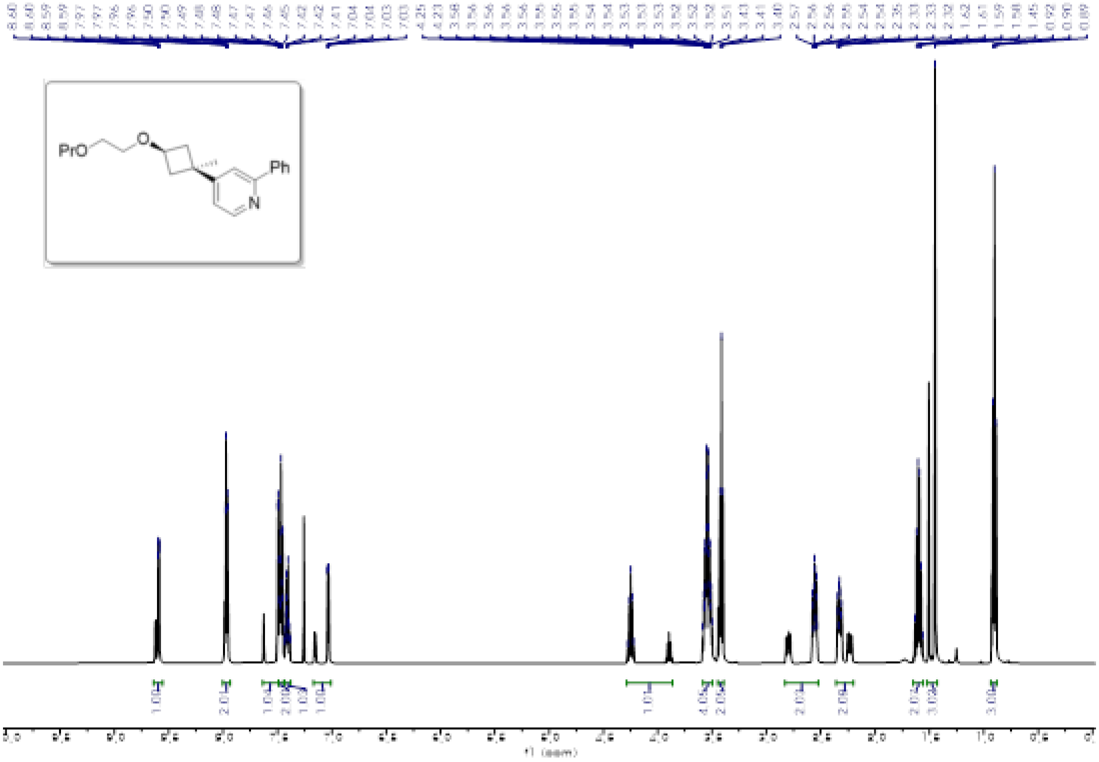

100 MHz, ^{13}C NMR in $CDCl_3$

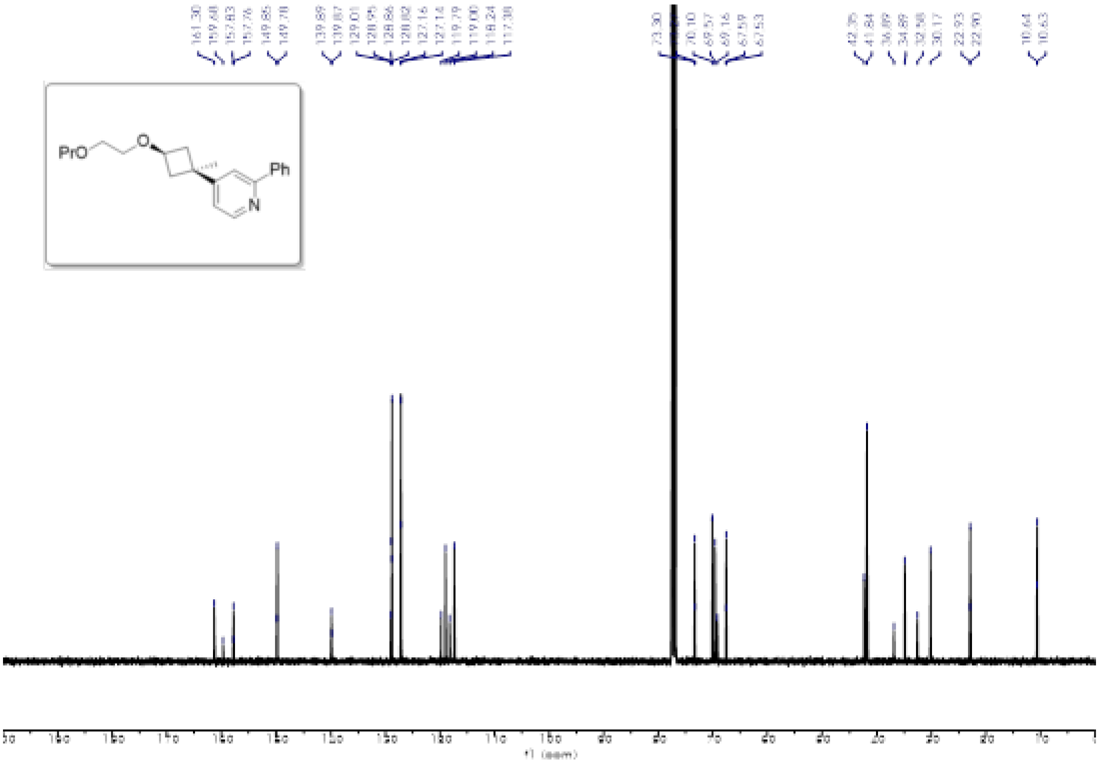

4-((1s,3s)-3-(cyclohexyloxy)-1-methylcyclobutyl)-2-phenylpyridine (3h).

500 MHz, ^{1}H NMR in CD_2Cl_2

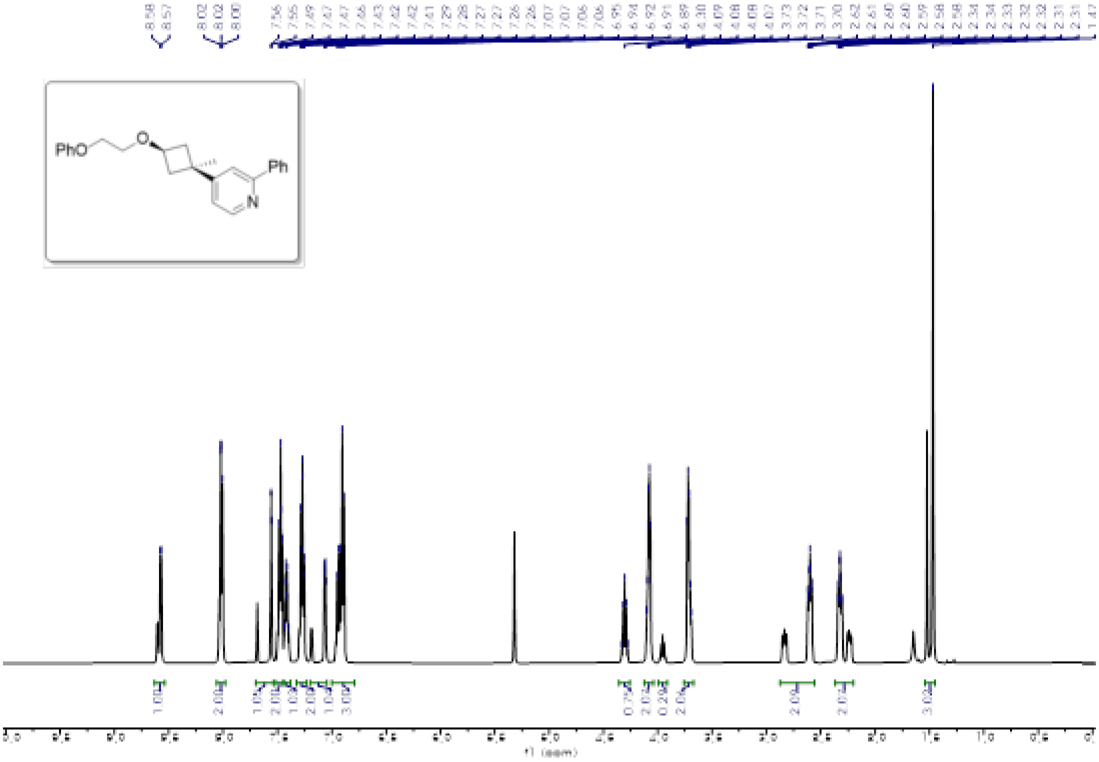

100 MHz, ^{13}C NMR in CD_2Cl_2

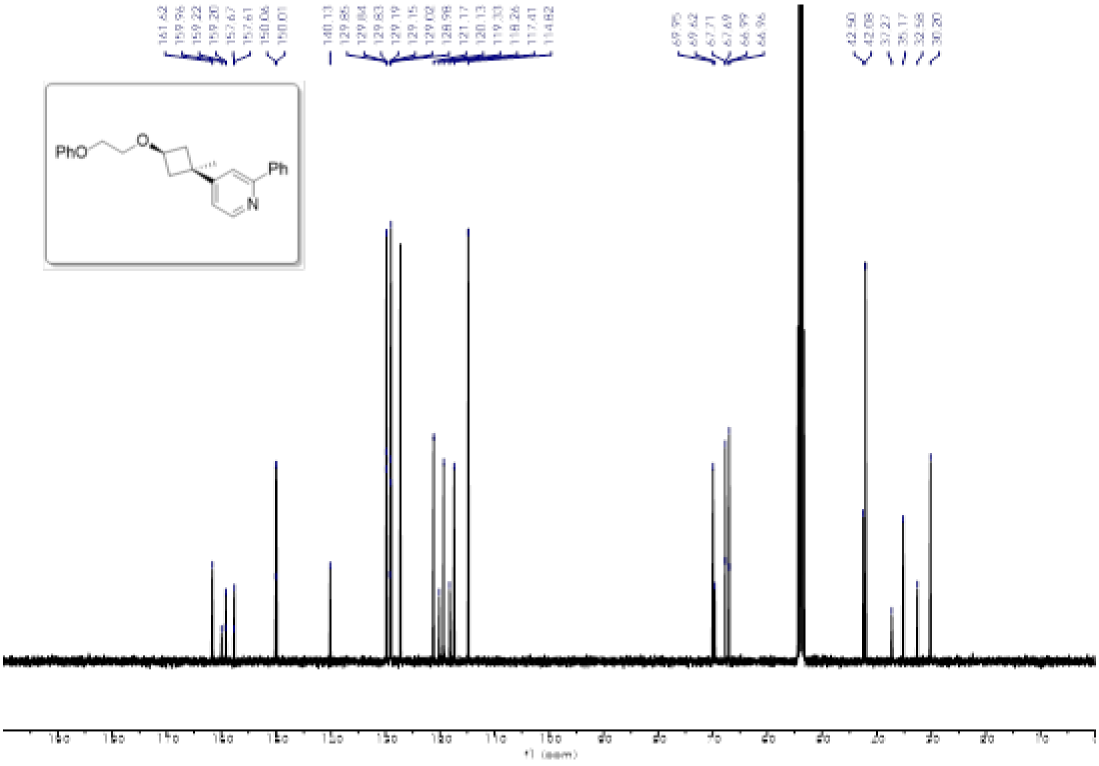

2-(4-bromophenyl)-4-((1s,3s)-3-ethoxy-1-methylcyclobutyl)pyridine (3i).

500 MHz, ^{1}H NMR in $CDCl_3$

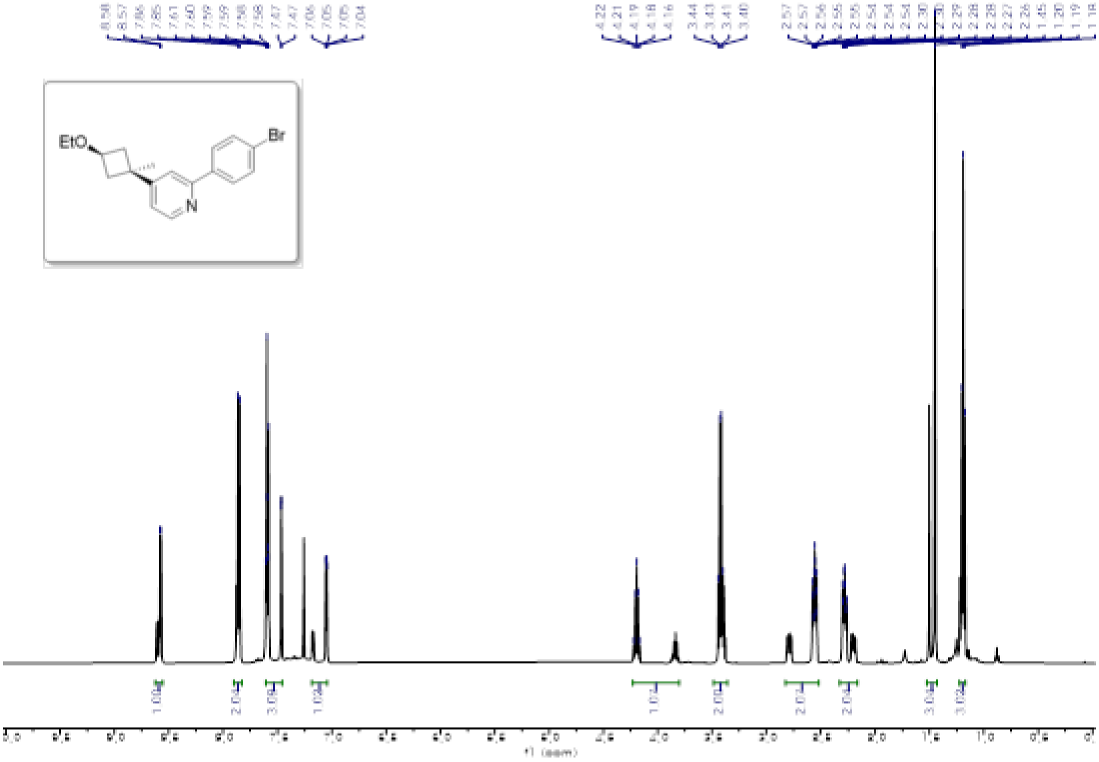

100 MHz, ^{13}C NMR in $CDCl_3$

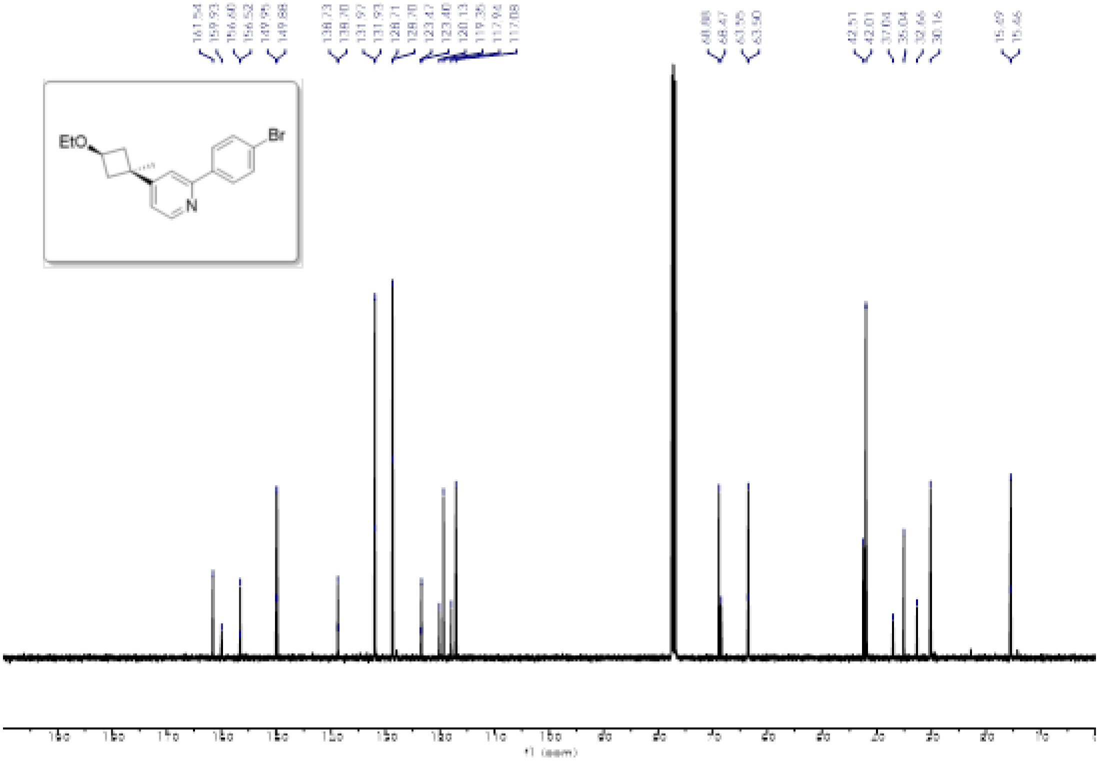

4-((1s,3s)-3-ethoxy-1-methylcyclobutyl)-2-(4-methoxyphenyl)pyridine (3j).

500 MHz, ^{1}H NMR in CDCl$_3$

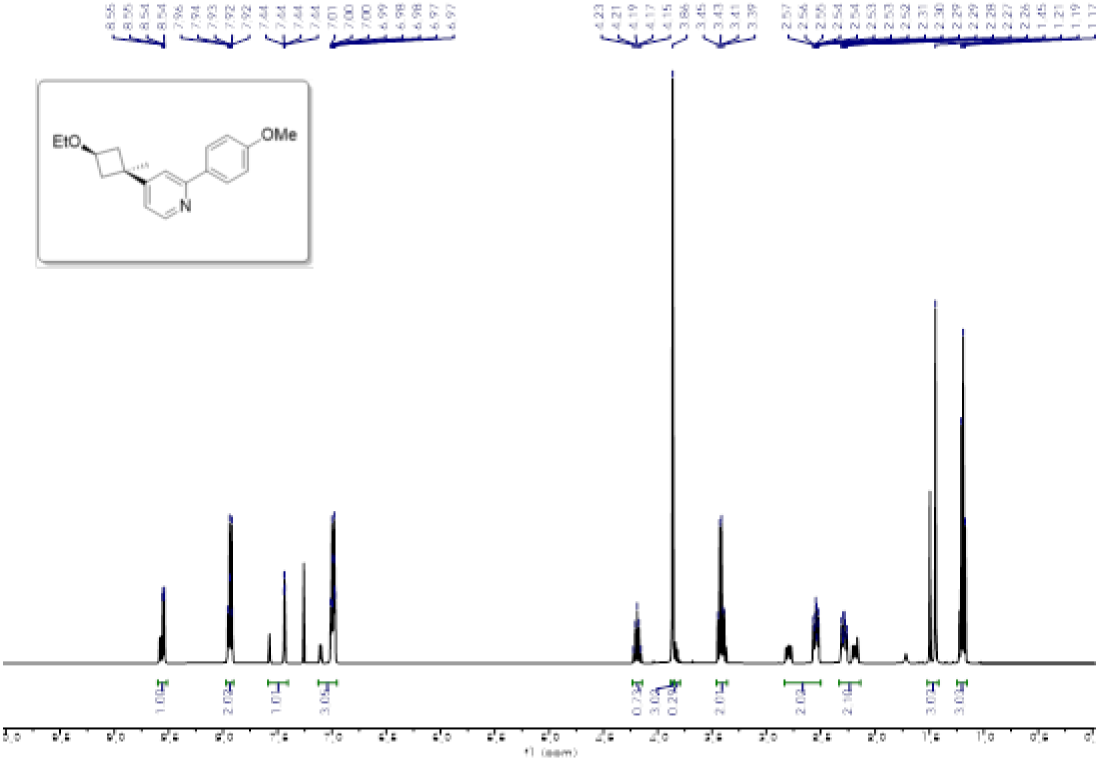

100 MHz, ^{13}C NMR in CDCl$_3$

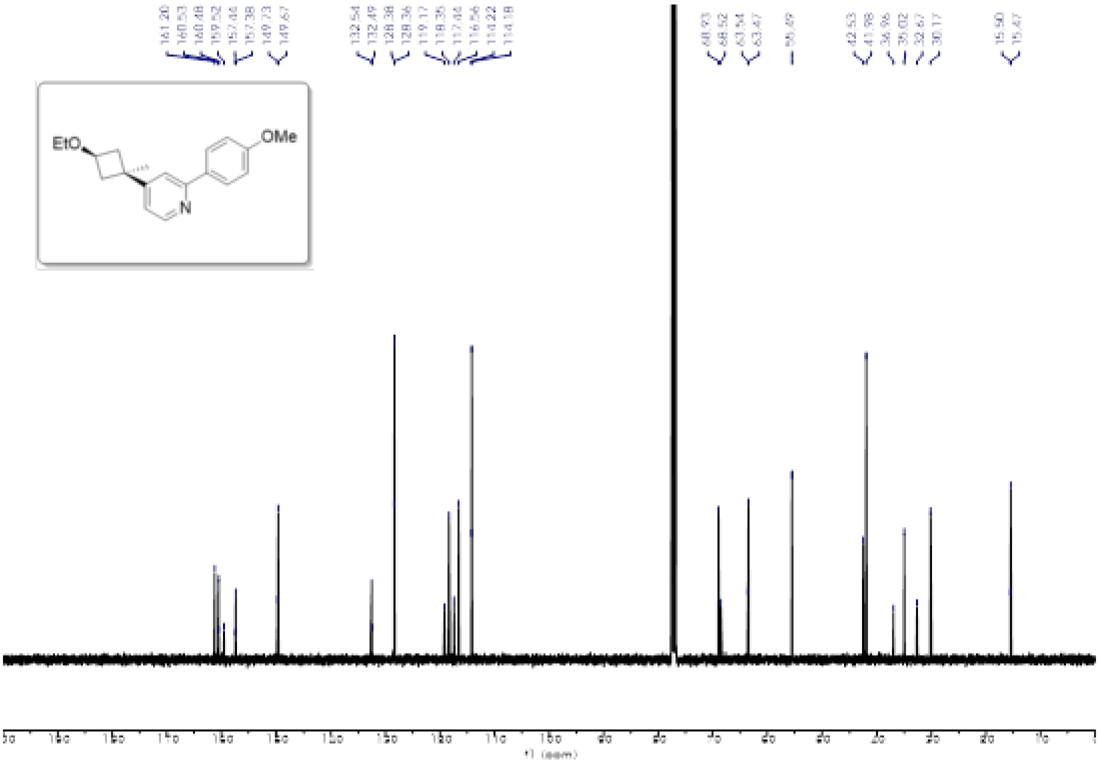

4-((1s,3s)-3-ethoxy-1-methylcyclobutyl)-2-(p-tolyl)pyridine (3k).

400 MHz, ^{1}H NMR in CD_2Cl_2

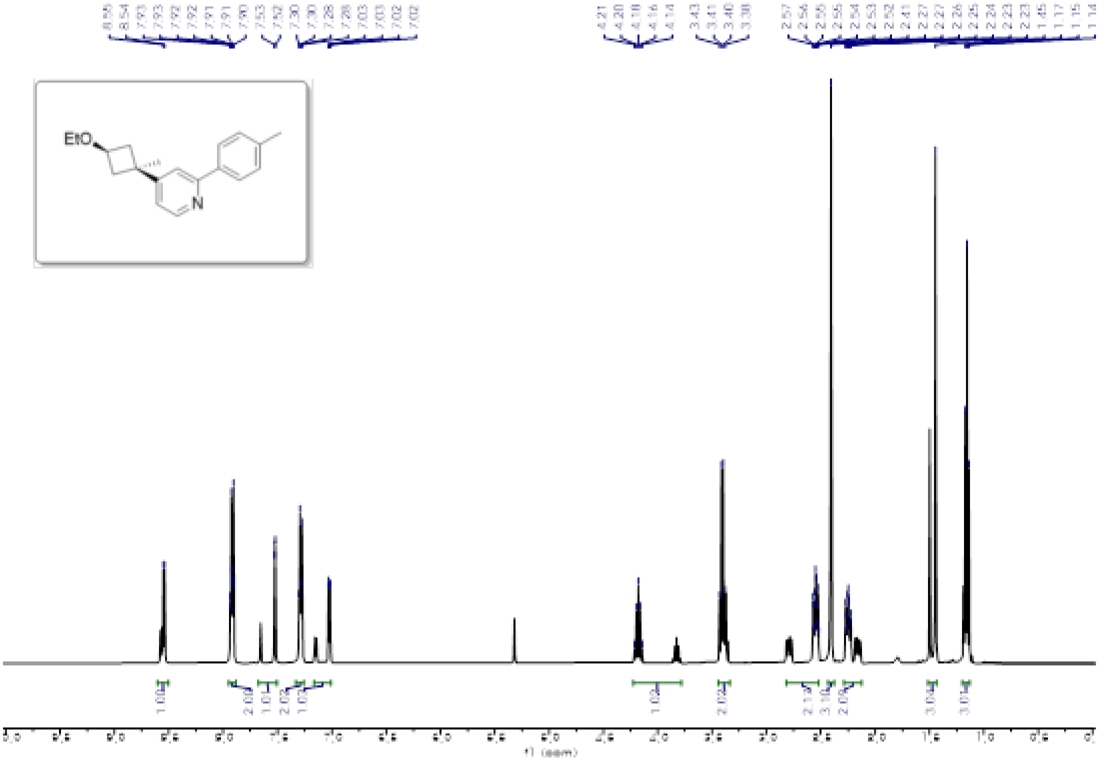

100 MHz, ^{13}C NMR in CD_2Cl_2

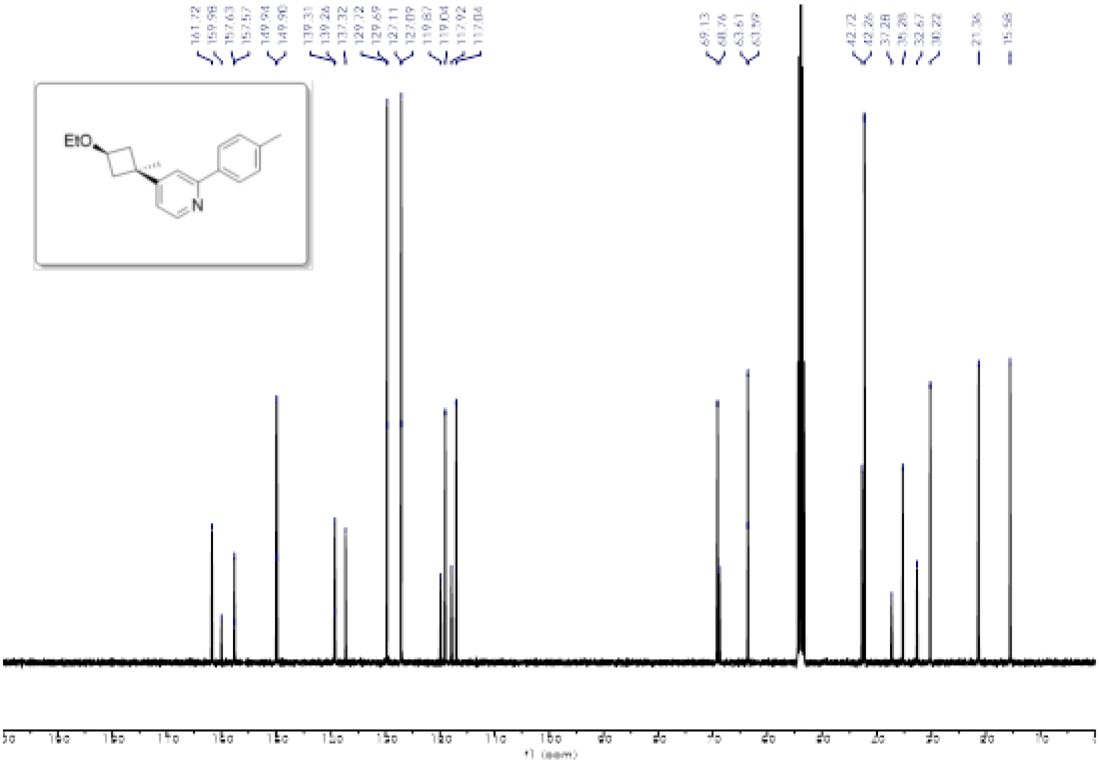

4-((1s,3s)-3-(cyclohexyloxy)-1-methylcyclobutyl)-2-phenylpyridine (3l).

500 MHz, ^{1}H NMR in $CDCl_3$

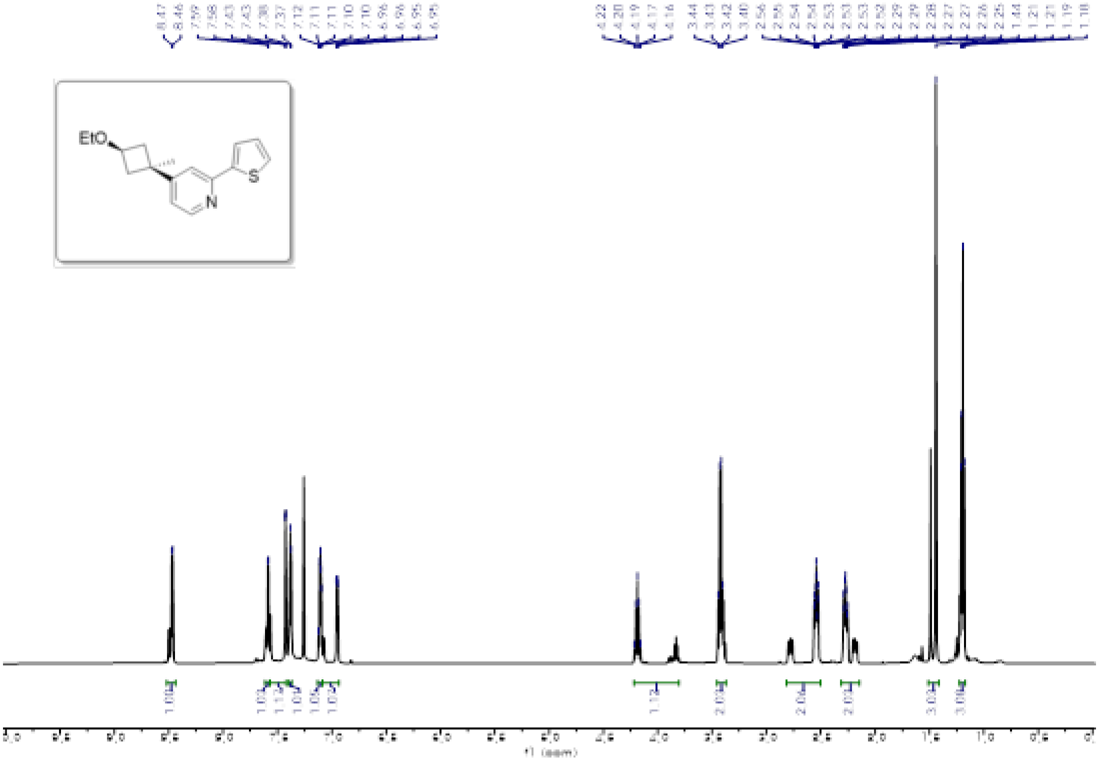

100 MHz, ^{13}C NMR in $CDCl_3$

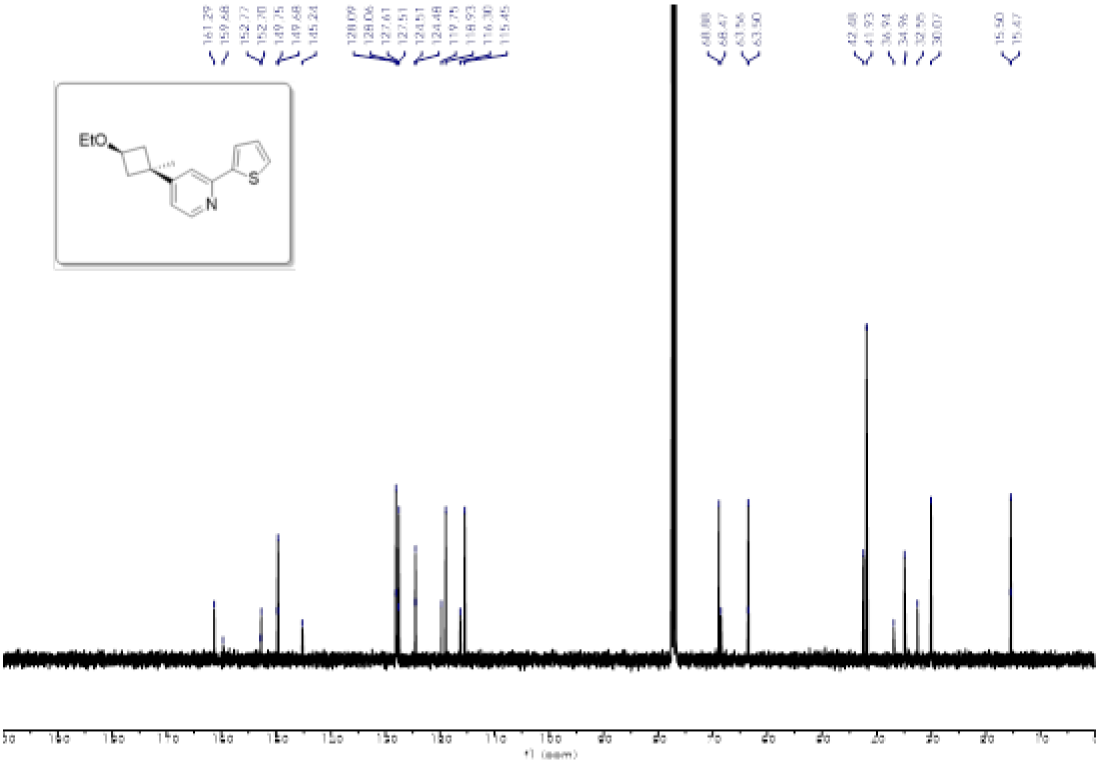

4-((1s,3s)-3-ethoxy-1-methylcyclobutyl)-2-(trifluoromethyl)pyridine (3m).

500 MHz, ^{1}H NMR in $CDCl_3$

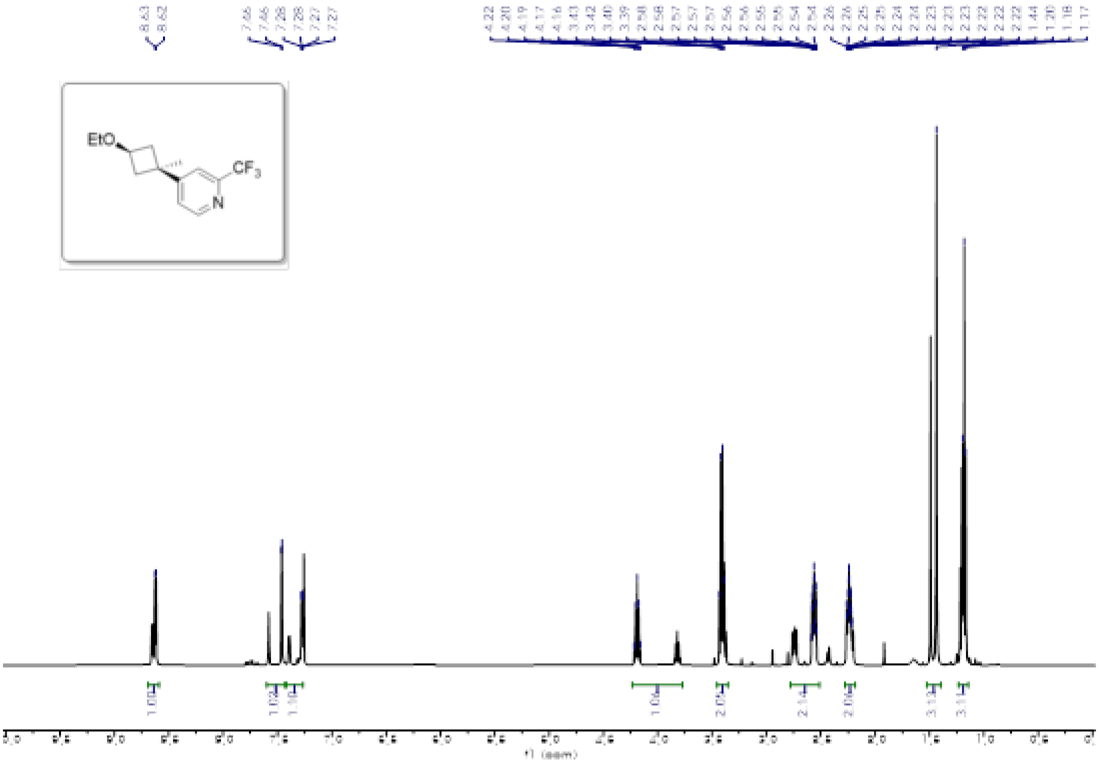

100 MHz, ^{13}C NMR in $CDCl_3$

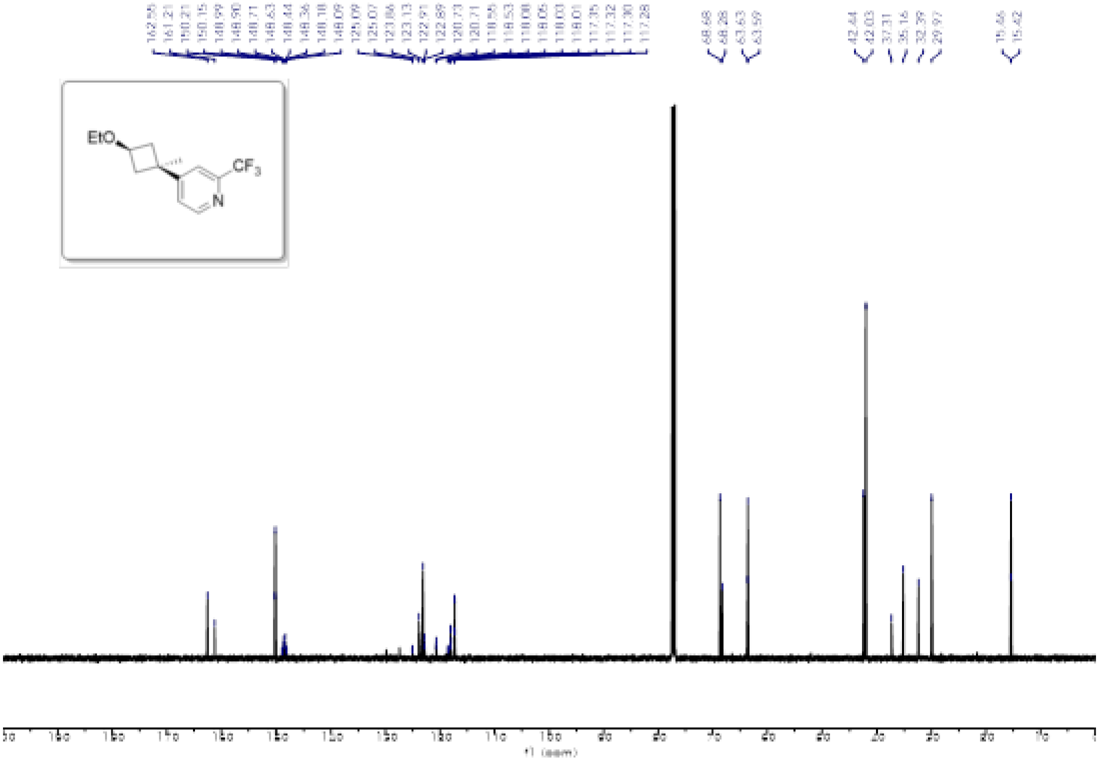

4-((1s,3s)-3-ethoxy-1-methylcyclobutyl)nicotinonitrile (3n).

400 MHz, ^{1}H NMR in $CDCl_3$

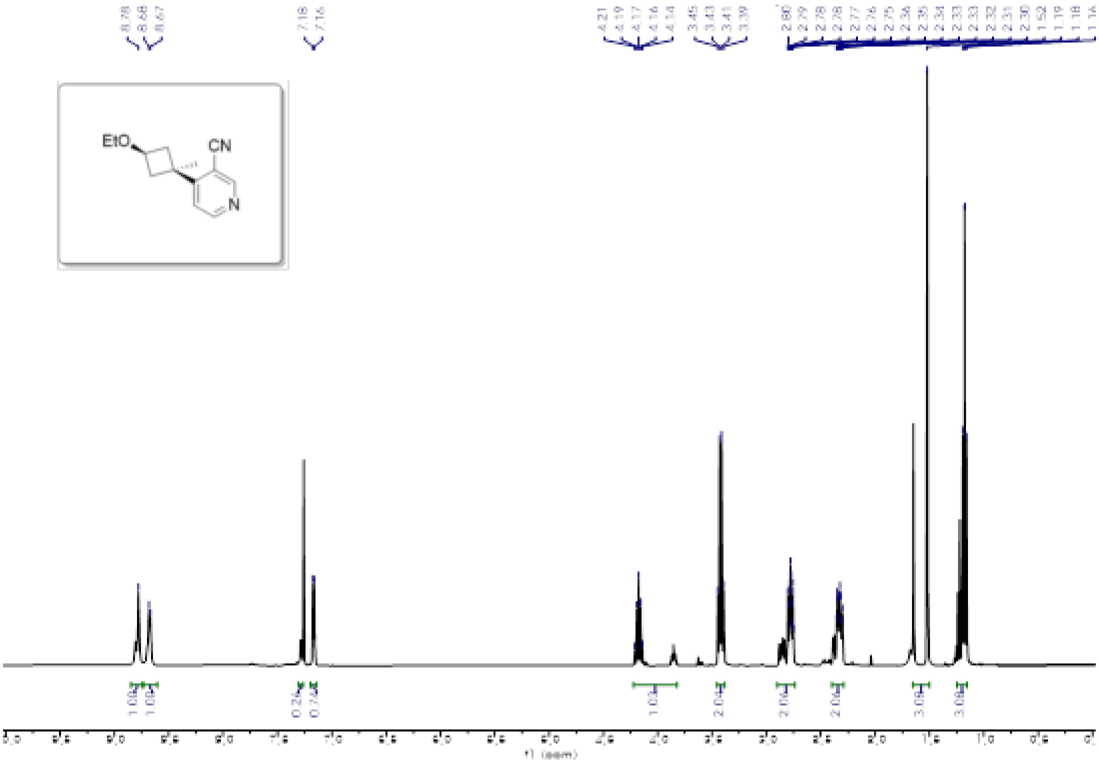

100 MHz, ^{13}C NMR in $CDCl_3$

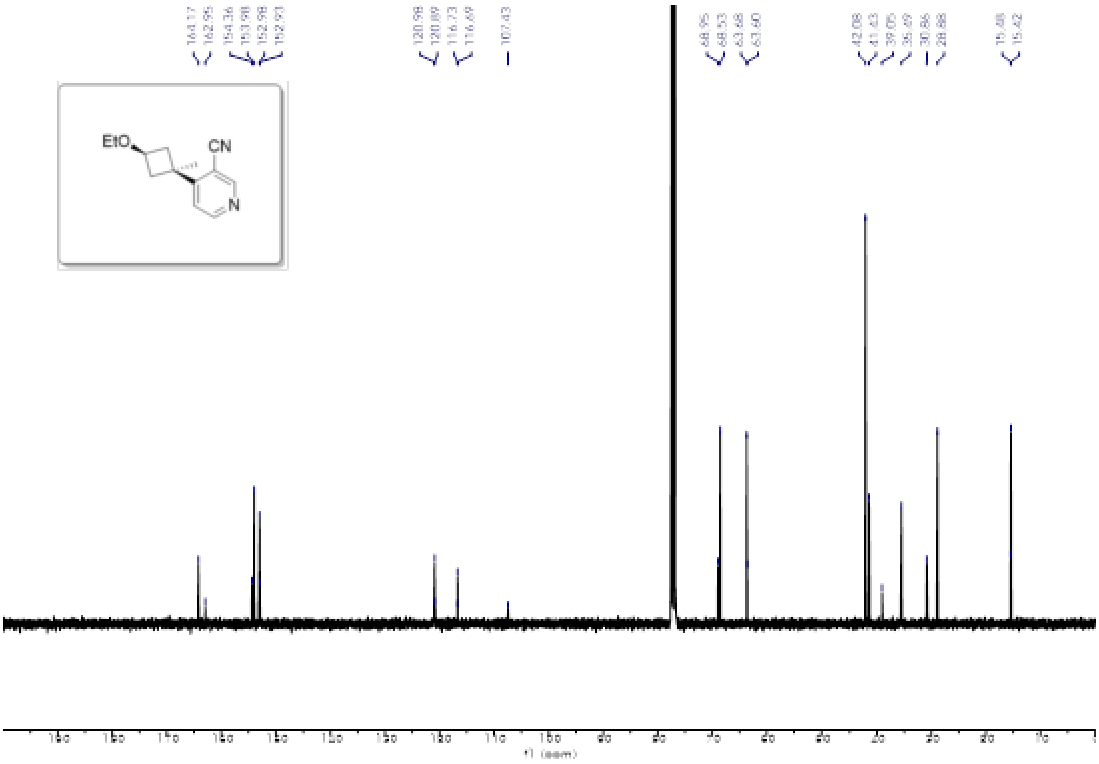

4-((1s,3s)-3-ethoxy-1-methylcyclobutyl)-2,6-dimethylpyridine (3o).

400 MHz, ^{1}H NMR in $CDCl_3$

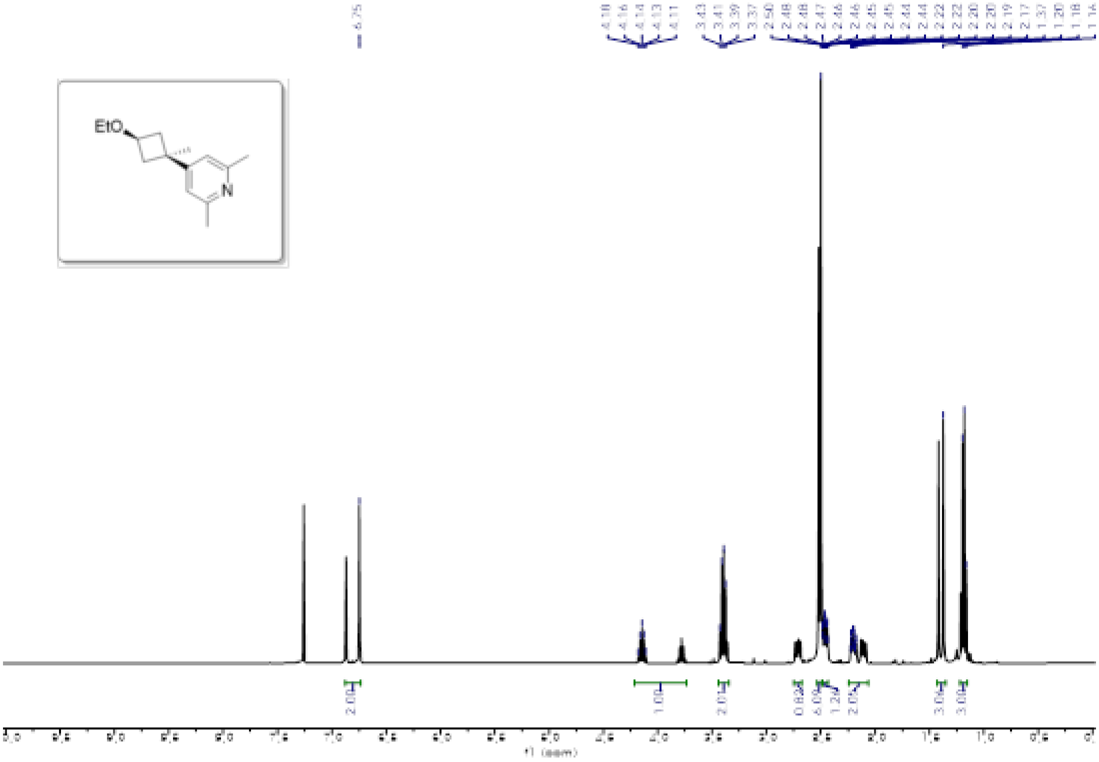

100 MHz, ^{13}C NMR in $CDCl_3$

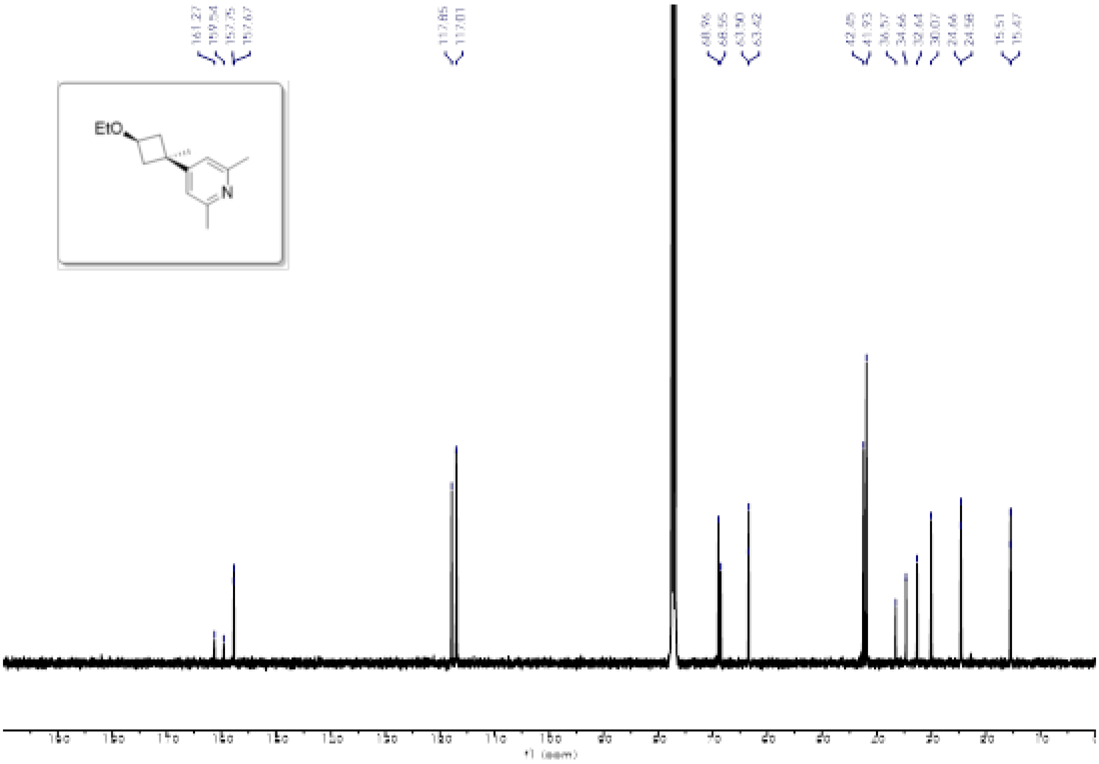

4-((1s,3s)-3-ethoxy-1-methylcyclobutyl)-3-methyl-2-phenylpyridine (3p).

400 MHz, ^{1}H NMR in CDCl$_3$

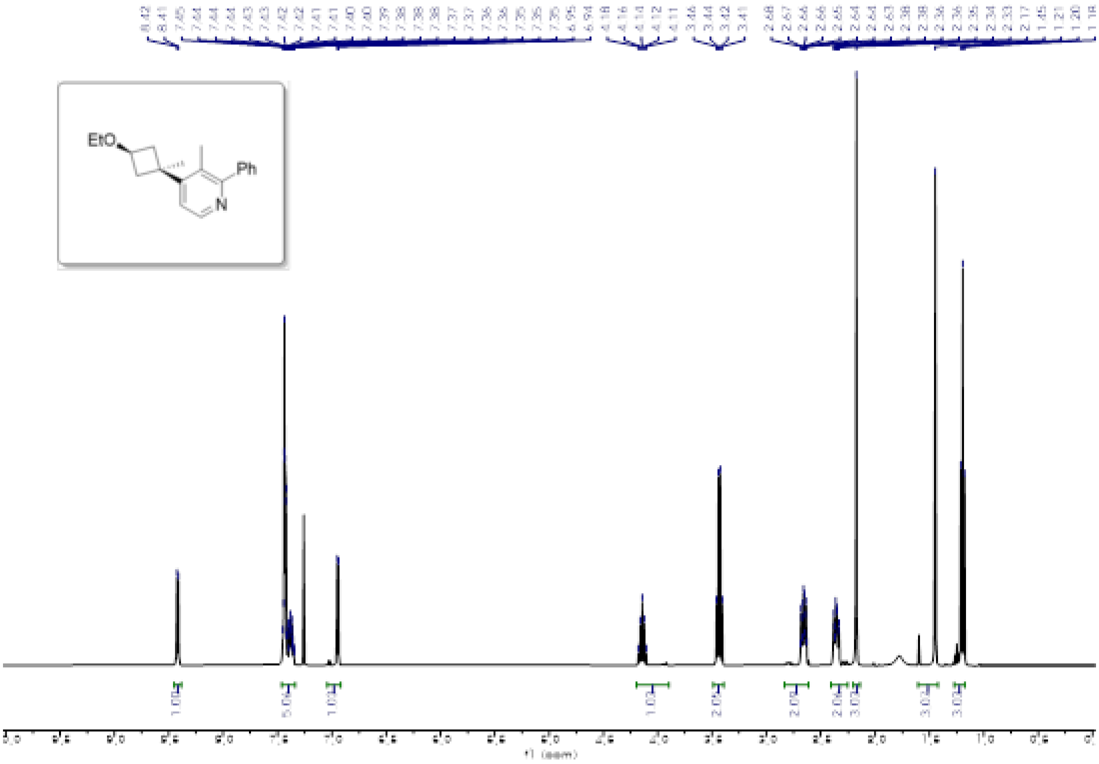

100 MHz, ^{13}C NMR in CDCl$_3$

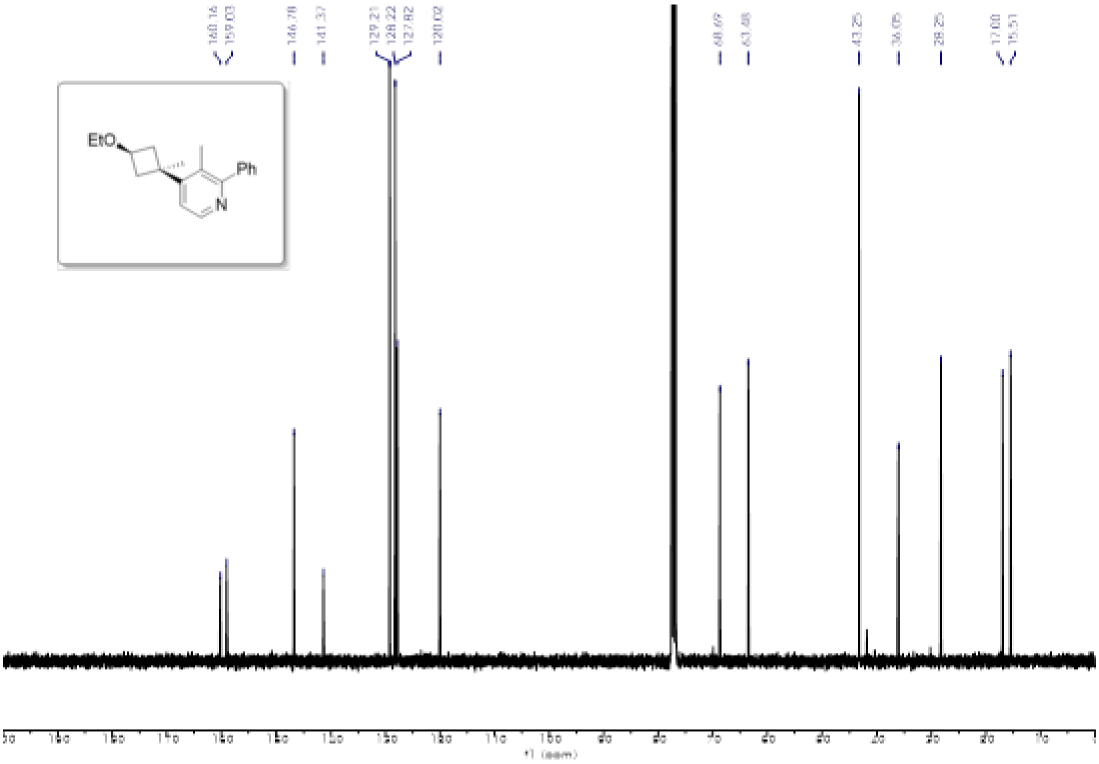

4-((1s,3s)-3-ethoxy-1-methylcyclobutyl)-6,7-dihydro-5H-cyclopenta[b]pyridine (3q).

400 MHz, ^{1}H NMR in $CDCl_3$

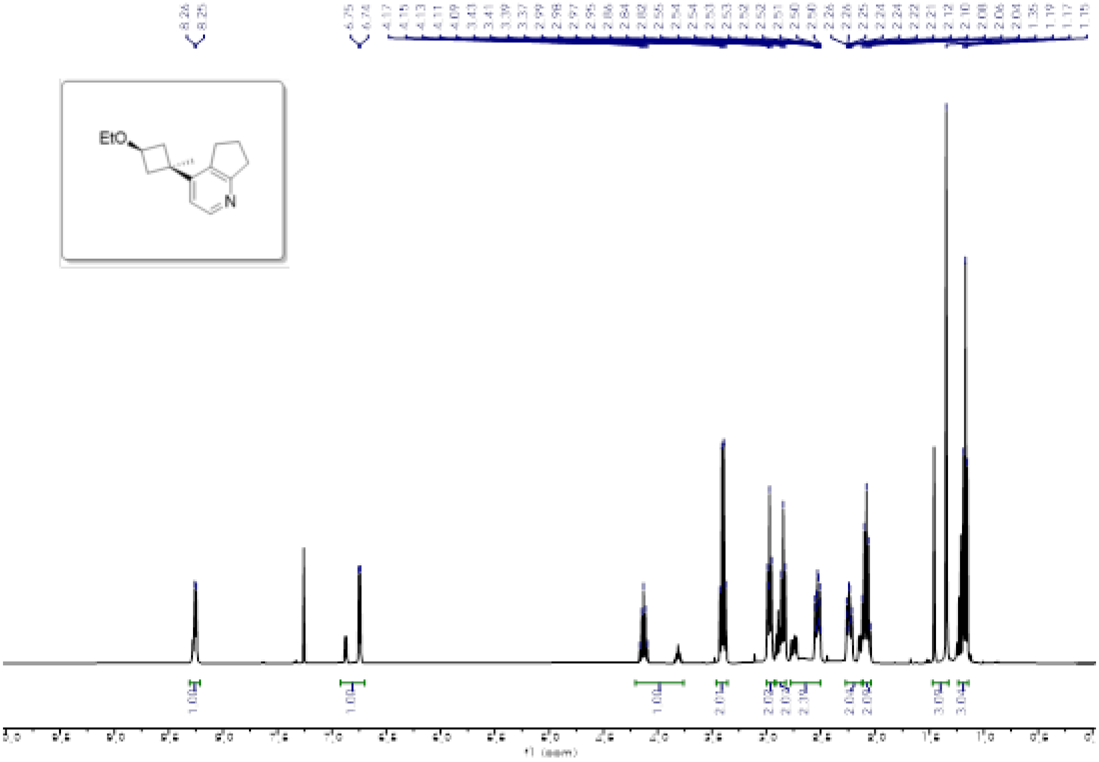

100 MHz, ^{13}C NMR in $CDCl_3$

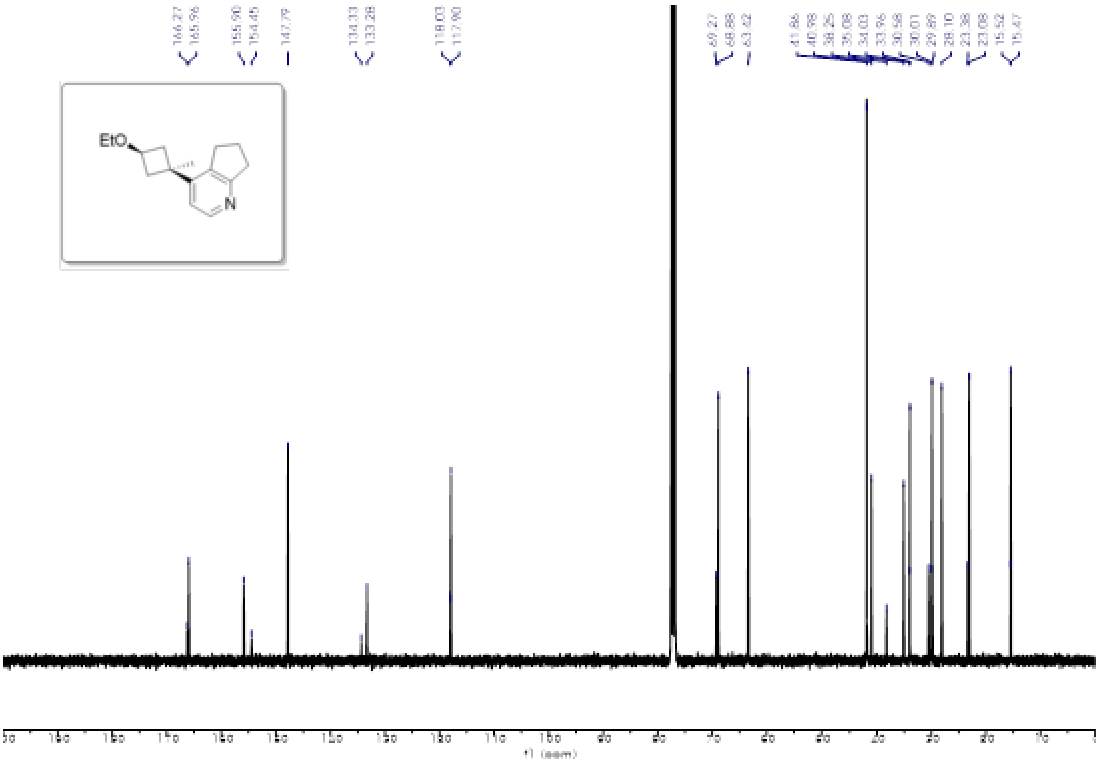

4-((1s,3s)-3-ethoxy-1-methylcyclobutyl)pyridine (3r).

400 MHz, ^1H NMR in $CDCl_3$

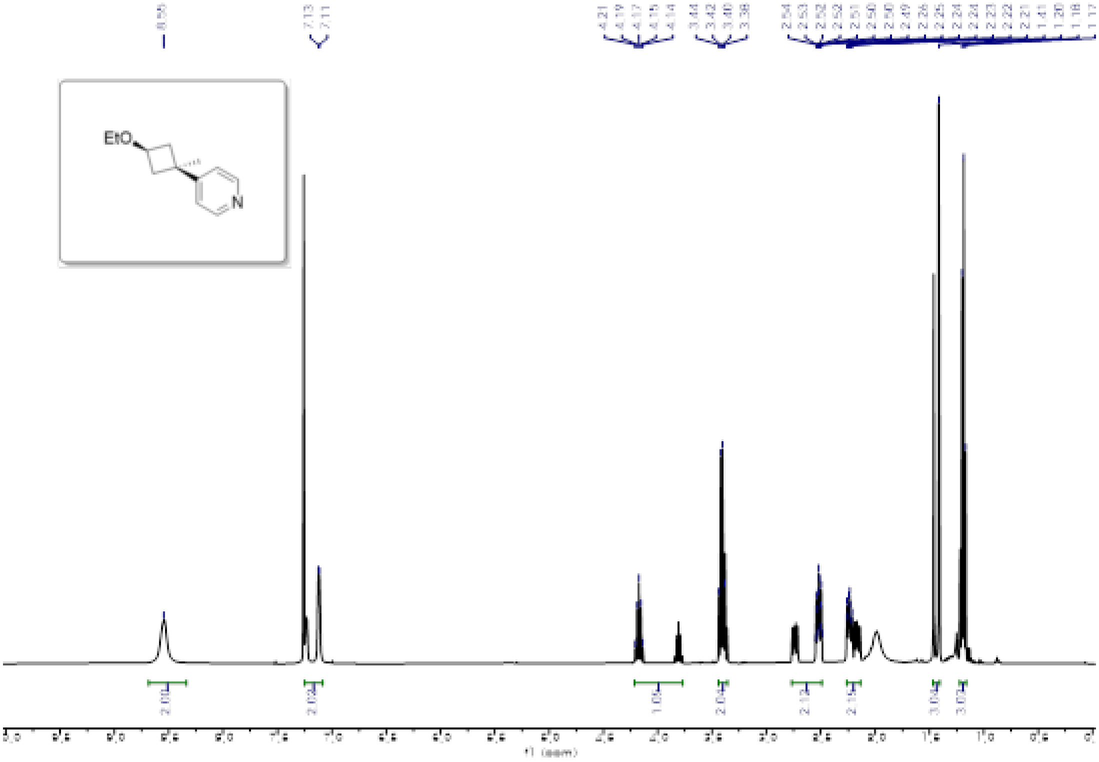

100 MHz, ^{13}C NMR in $CDCl_3$

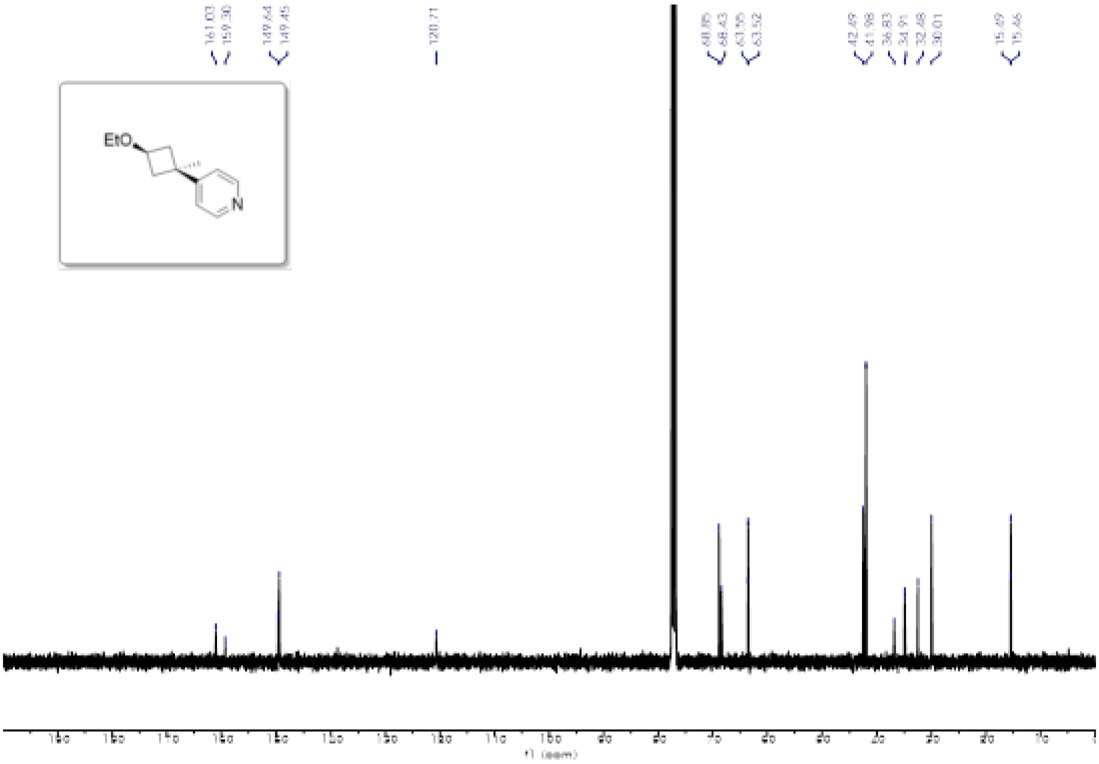

2-chloro-N-(4-chloro-3-(4-((1s,3s)-3-ethoxy-1-methylcyclobutyl)pyridin-2-yl)phenyl)-4-(methylsulfonyl)benzamide (3s).

500 MHz, ^{1}H NMR in CD_2Cl_2

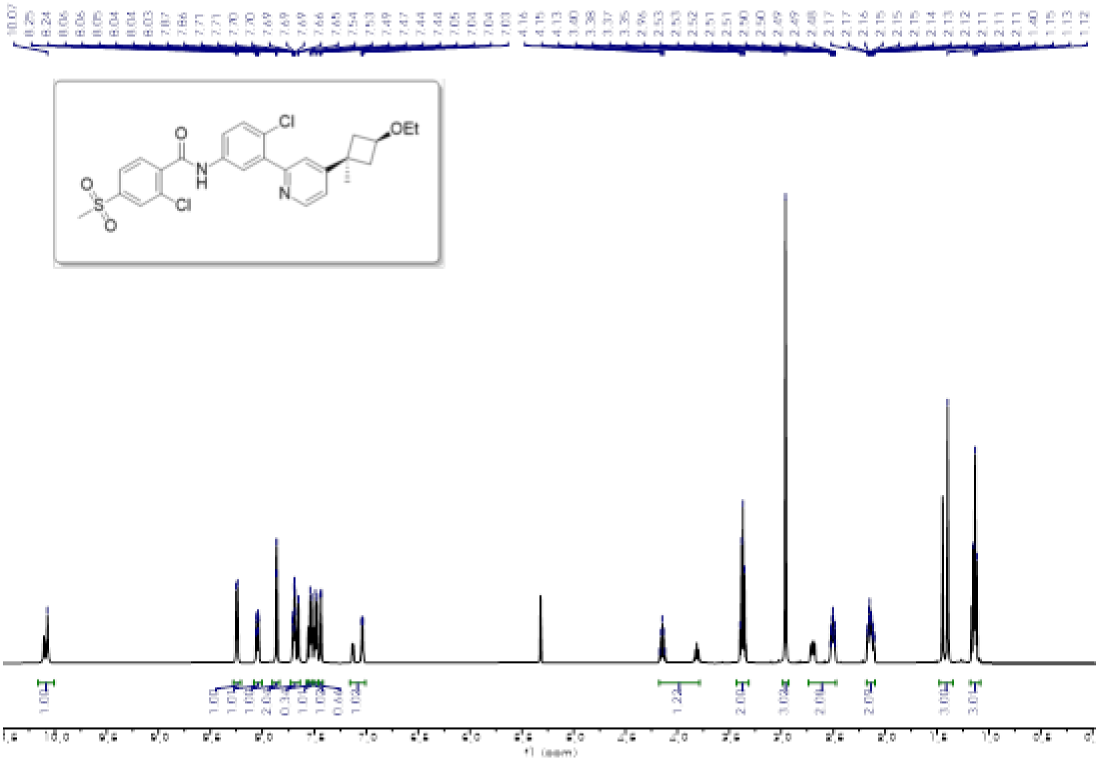

100 MHz, ^{13}C NMR in CD_2Cl_2

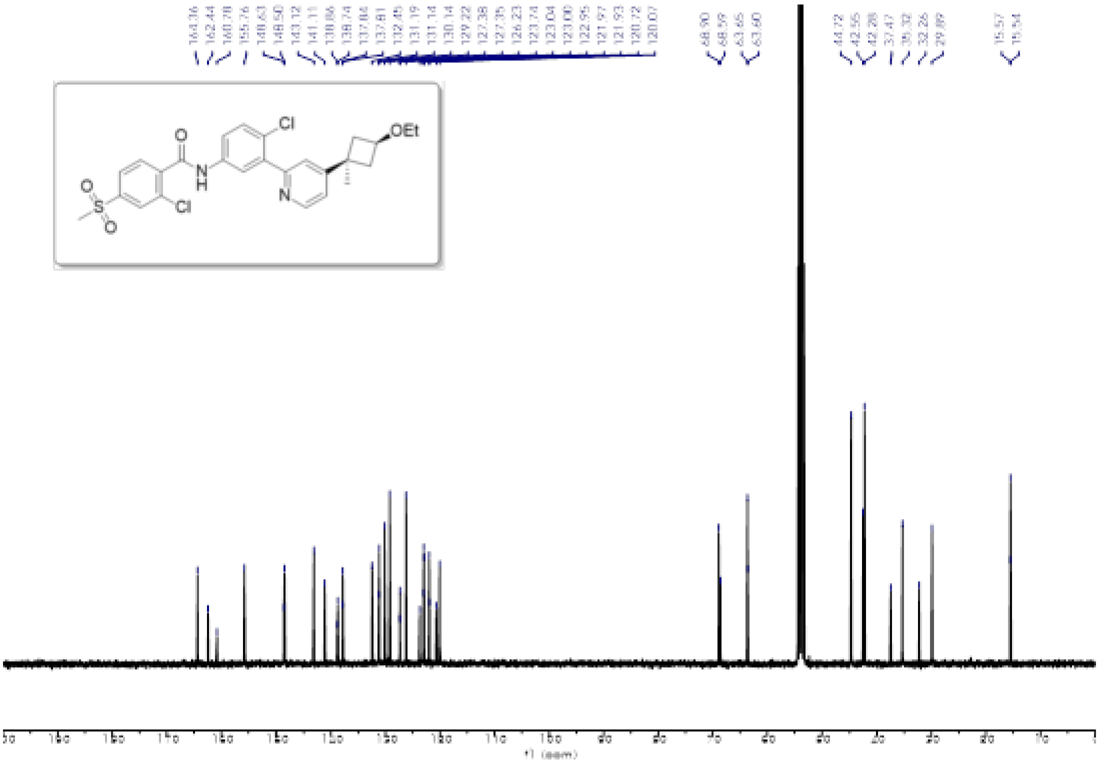

((4-((1s,3s)-3-ethoxy-1-methylcyclobutyl)pyridin-2-yl)methylene)bis(4,1-phenylene) diacetate (3t).

400 MHz, ^{1}H NMR in $CDCl_3$

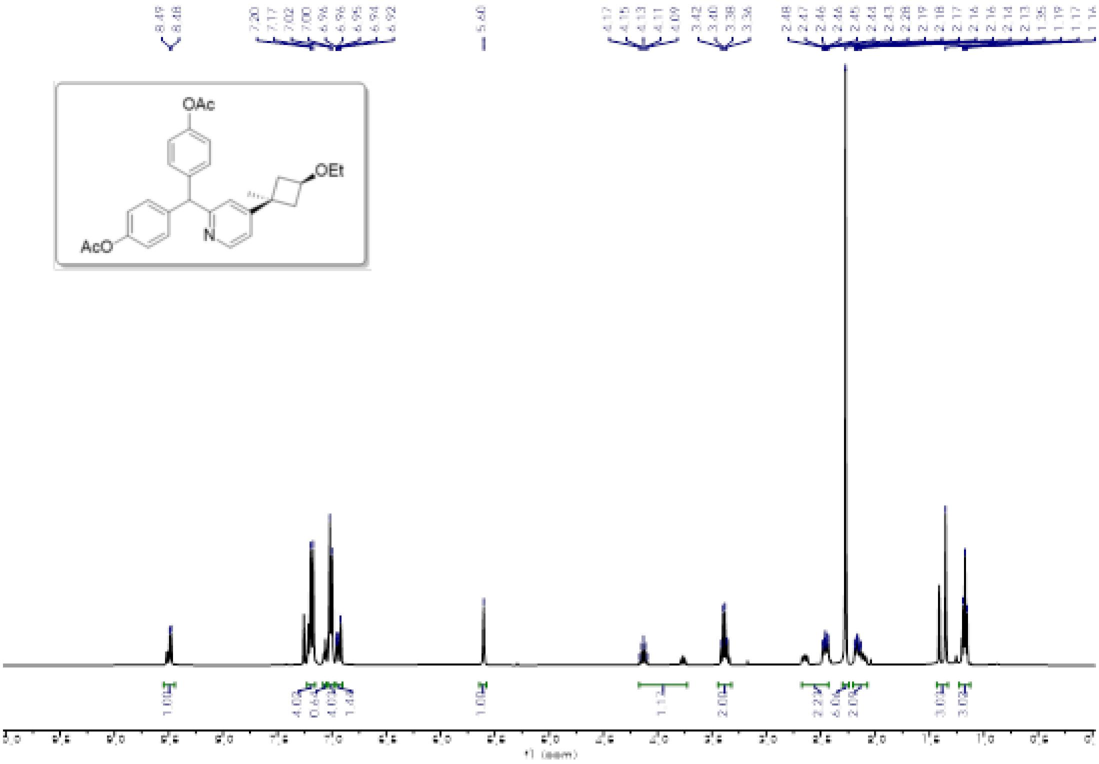

100 MHz, ^{13}C NMR in $CDCl_3$

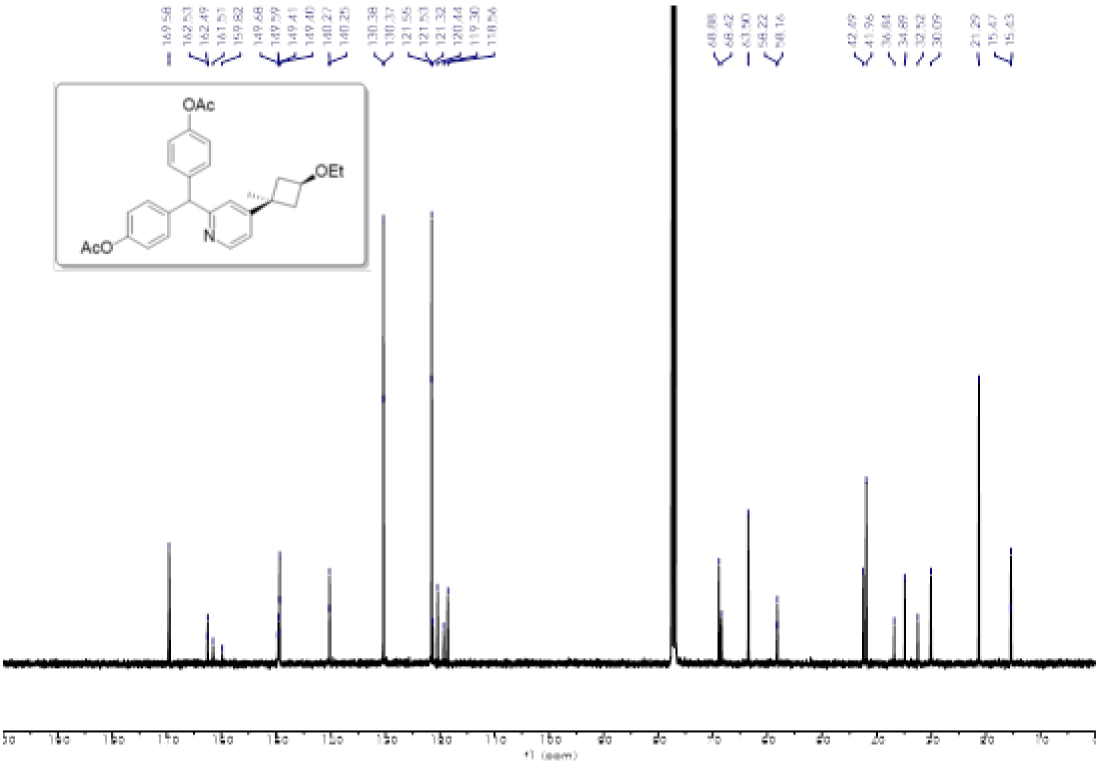

1-methoxy-3-methylenecyclobutane (4a).

500 MHz, ^{1}H NMR in CDCl$_3$

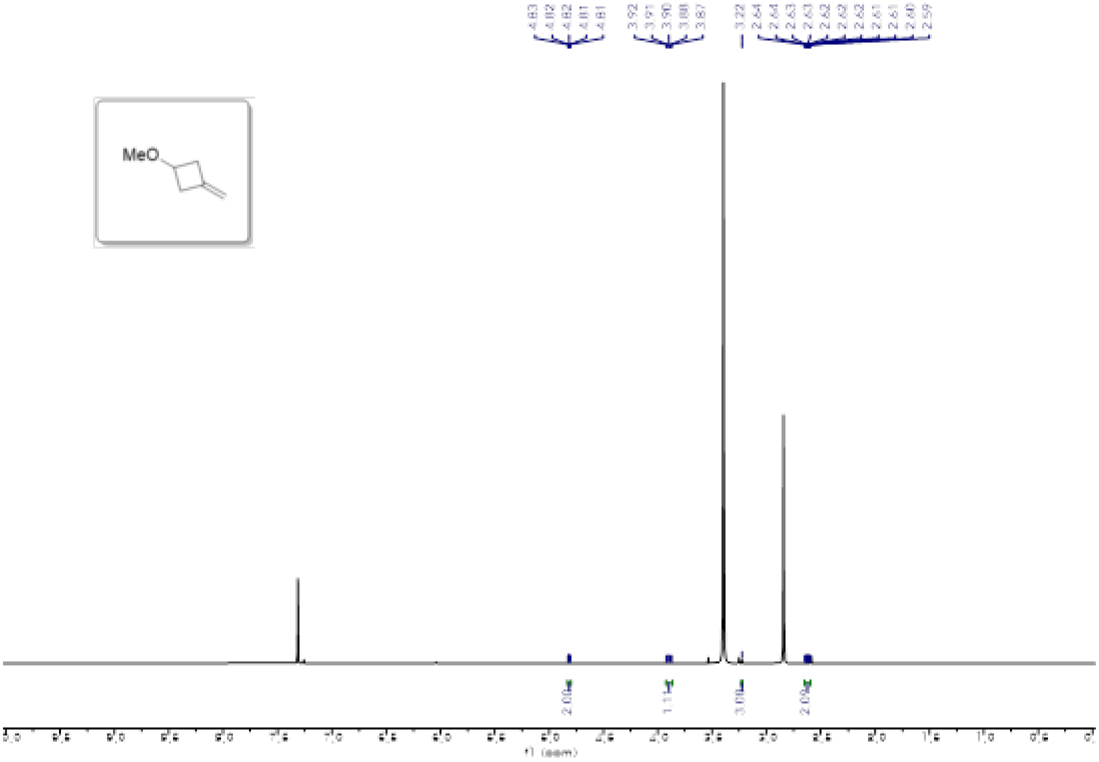

Enlarged(5.0 ppm~2.0 ppm)

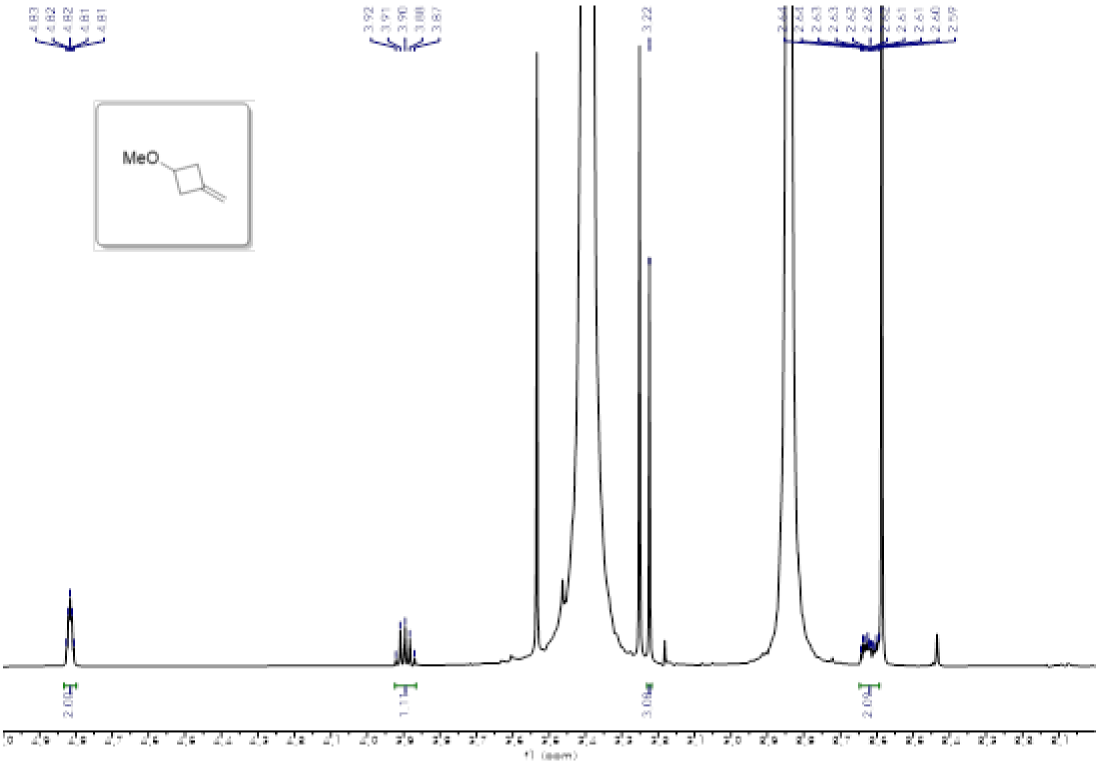

1-ethoxy-3-methylenecyclobutane (4b).

500 MHz, ^{1}H NMR in $CDCl_3$

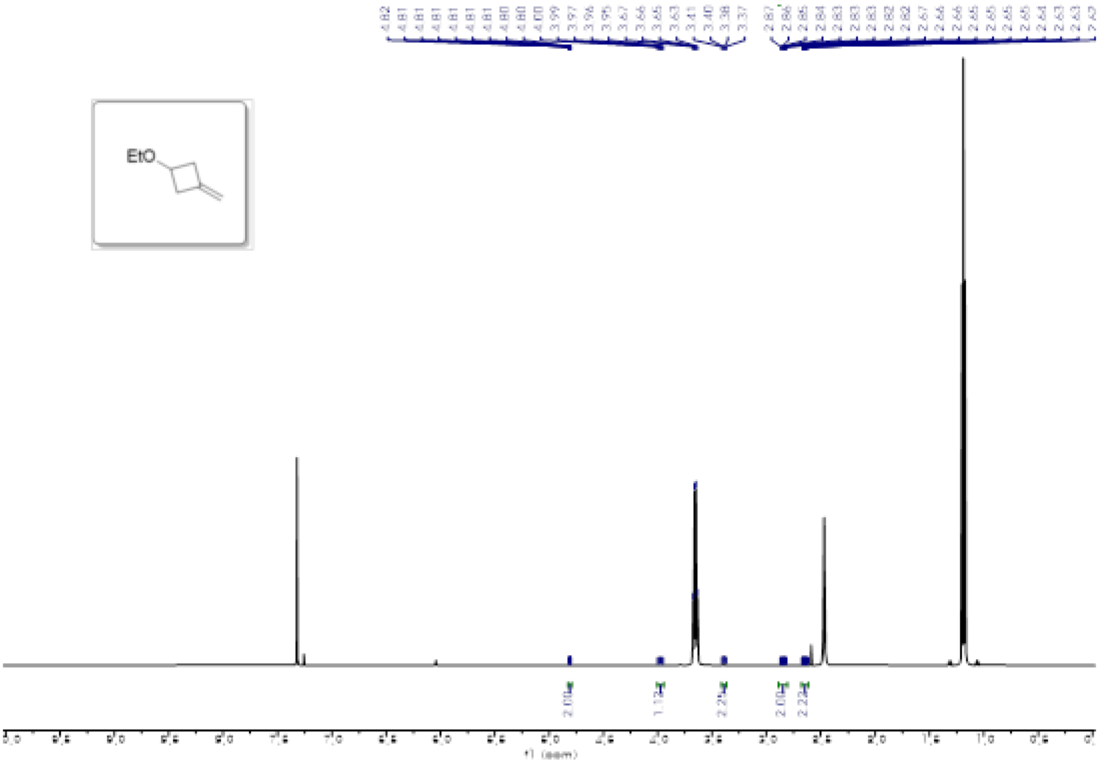

Enlarged(5.0 ppm~2.0 ppm)

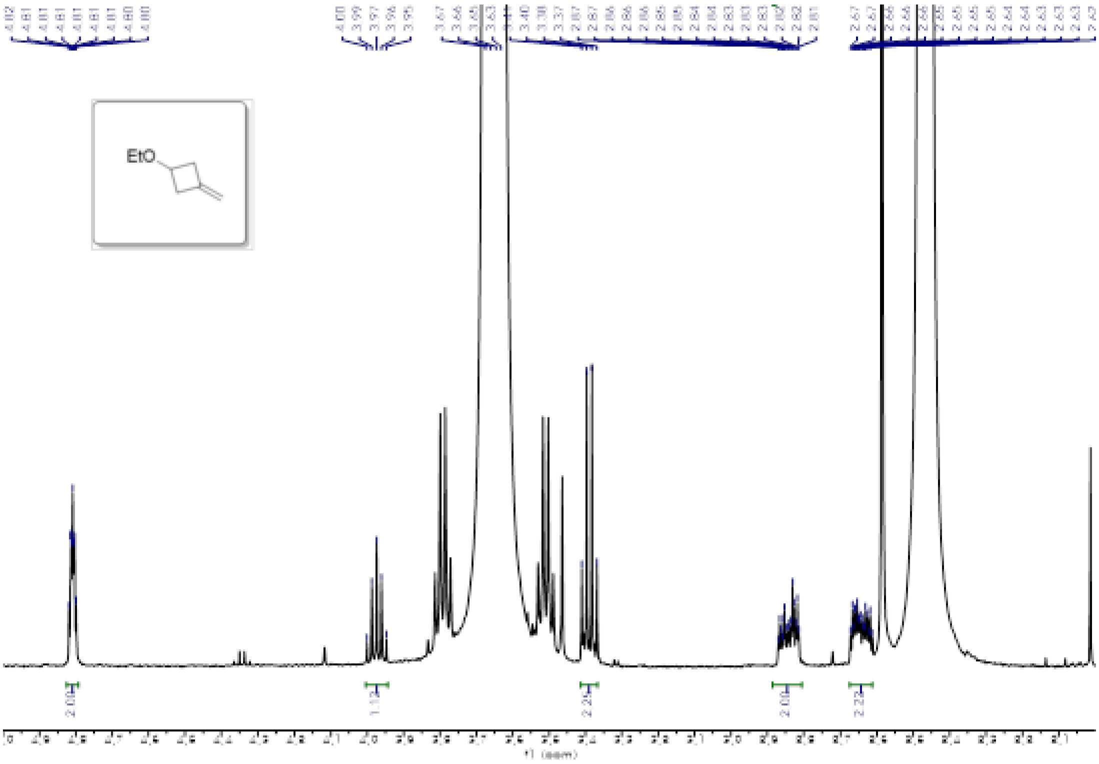

1-isopropoxy-3-methylenecyclobutane (4c).

500 MHz, ^{1}H NMR in $CDCl_3$

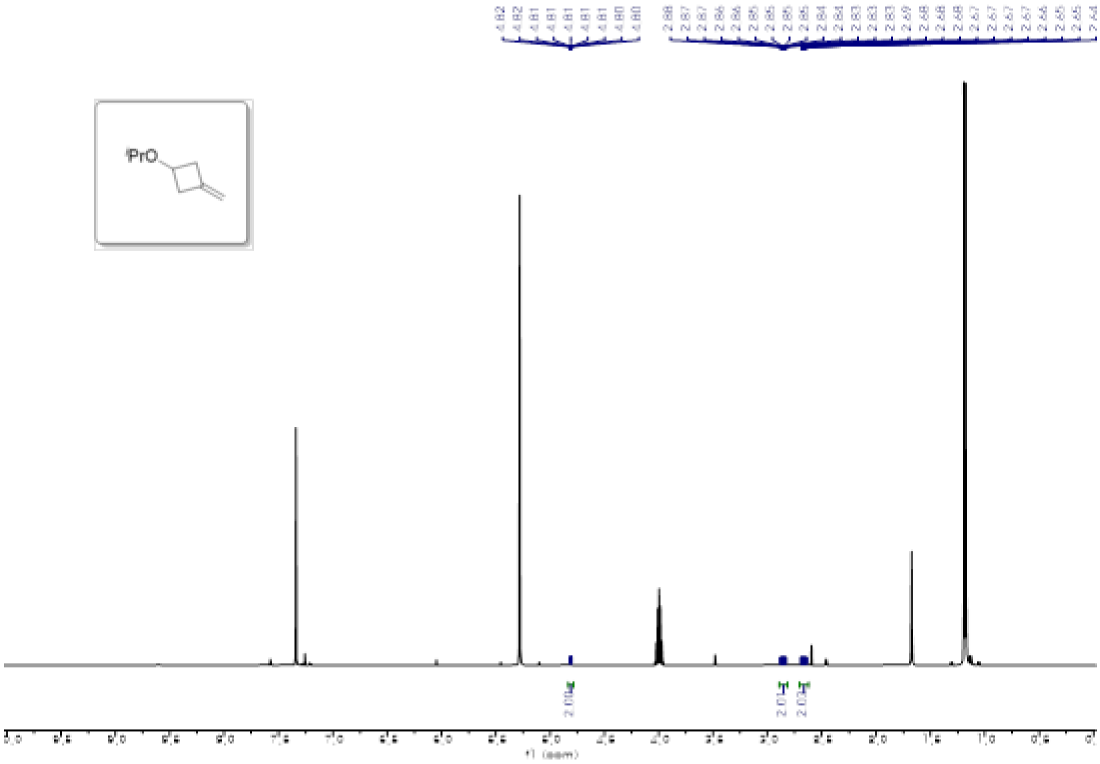

Enlarged(5.0 ppm~2.0 ppm)

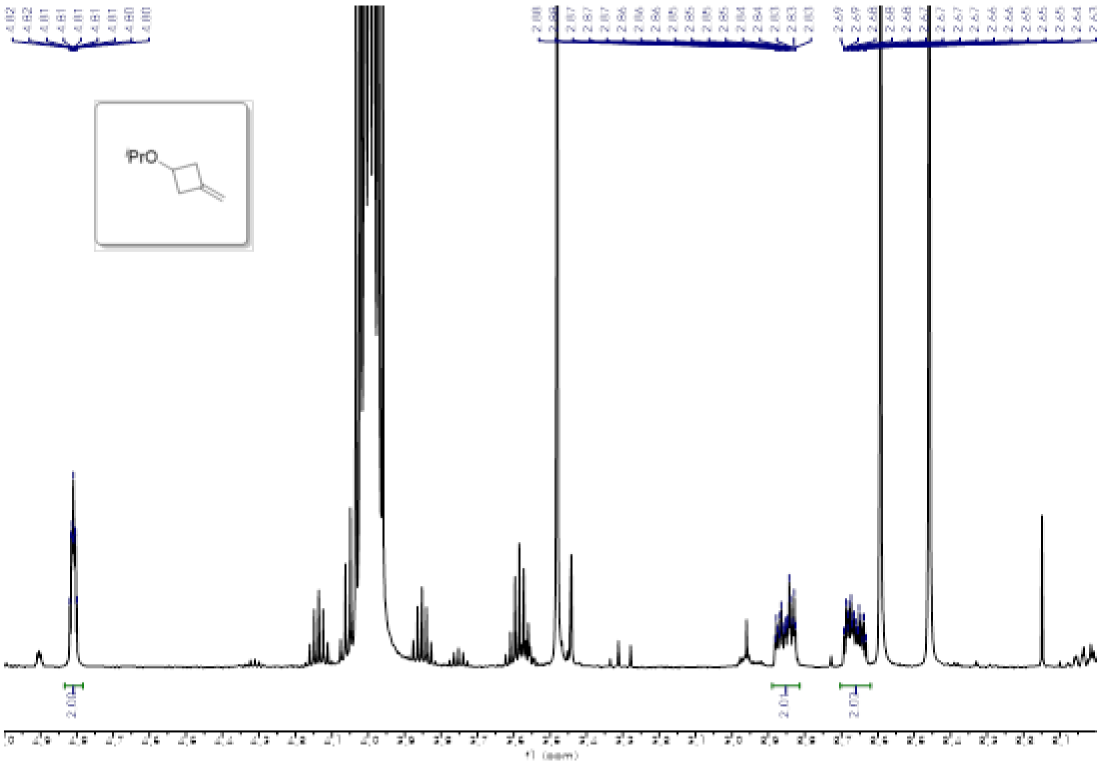

1-methylene-3-(tert-pentyloxy)cyclobutane (4d).

500 MHz, ^{1}H NMR in $CDCl_3$

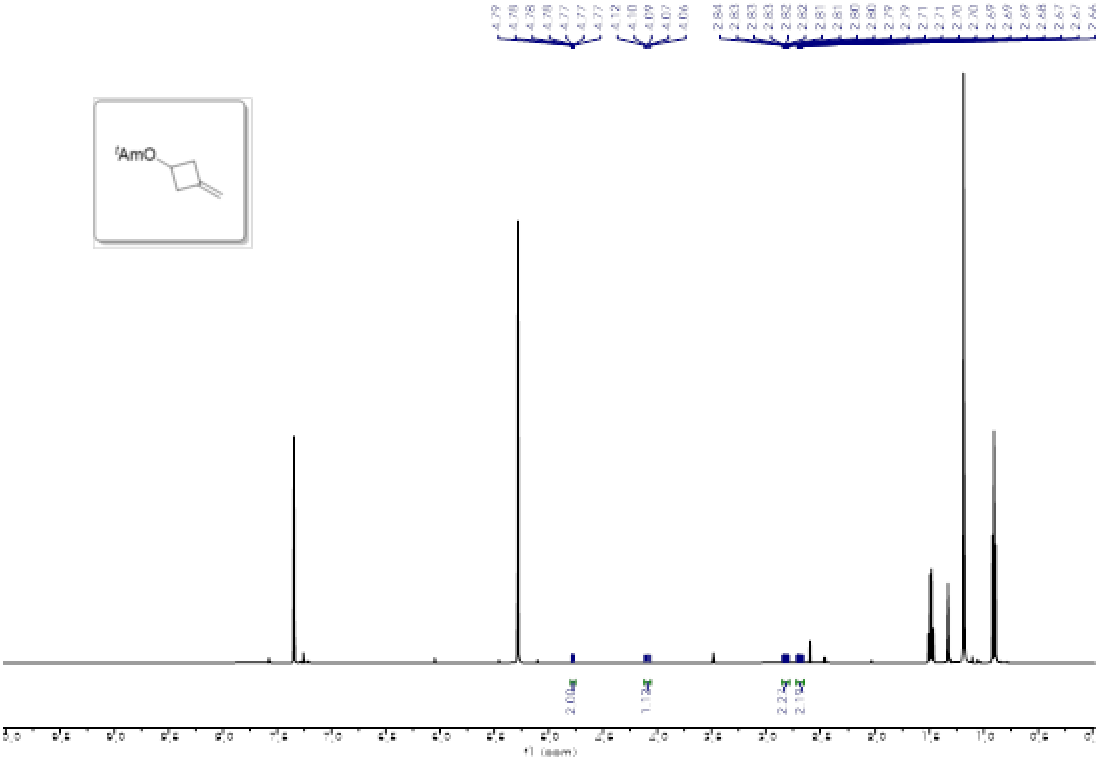

Enlarged(5.0 ppm~2.0 ppm)

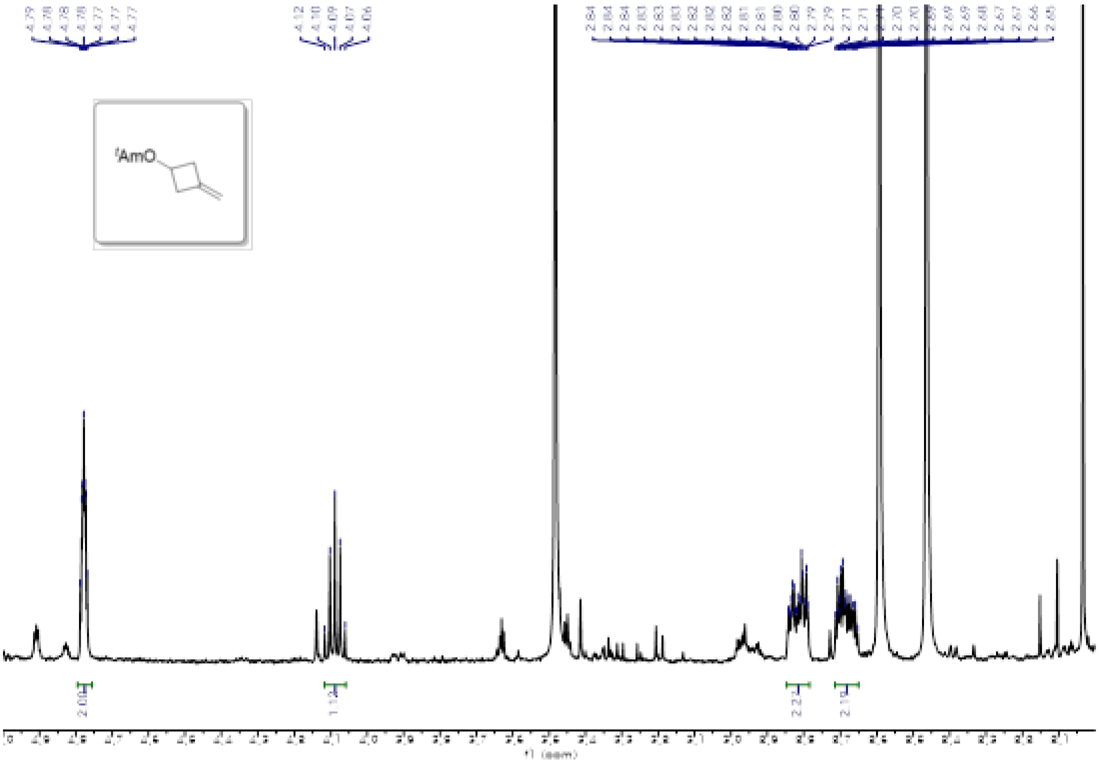

(2-(3-methylenecyclobutoxy)ethoxy)benzene (4e).

500 MHz, ^{1}H NMR in CD_2Cl_2

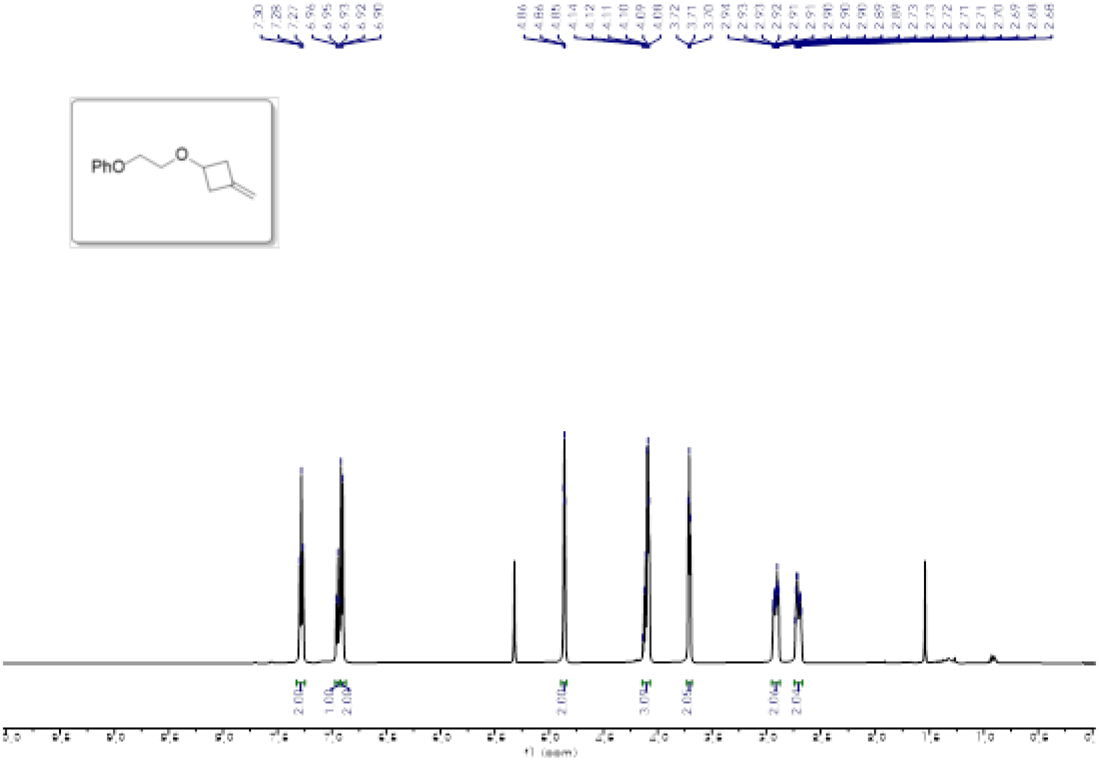

125 MHz, ^{13}C NMR in CD_2Cl_2

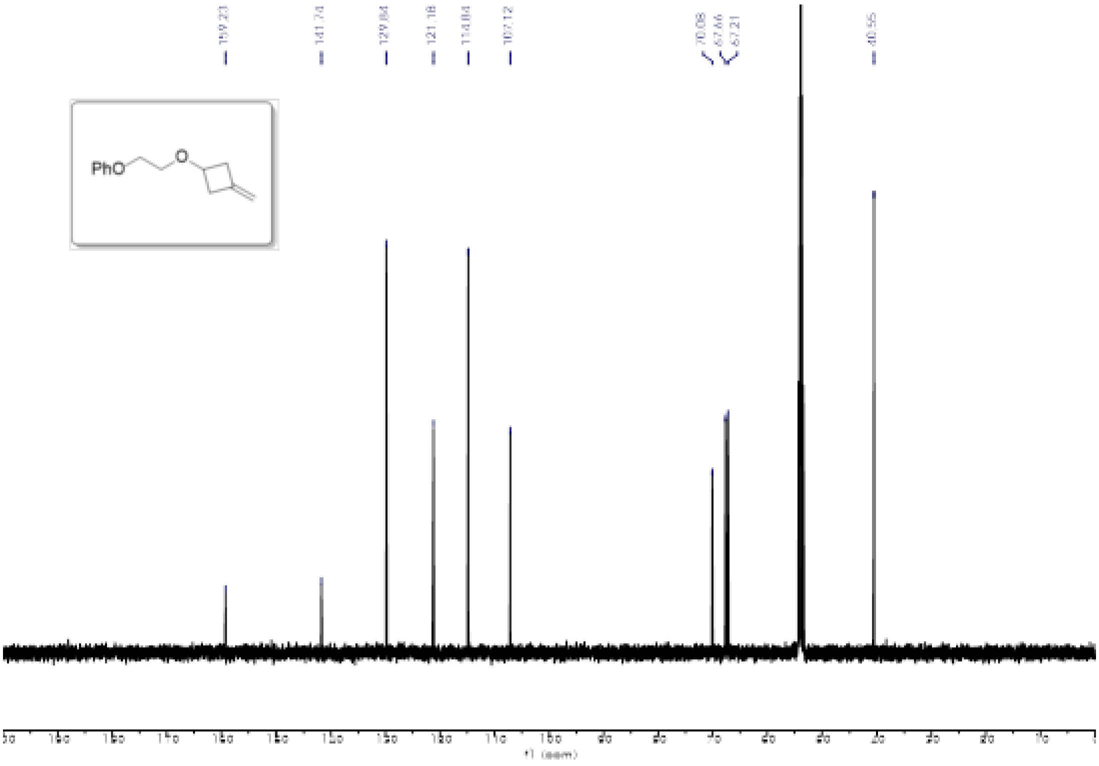

((3-methylenecyclobutoxy)methyl)benzene (4f).

600 MHz, ^{1}H NMR in $CDCl_3$

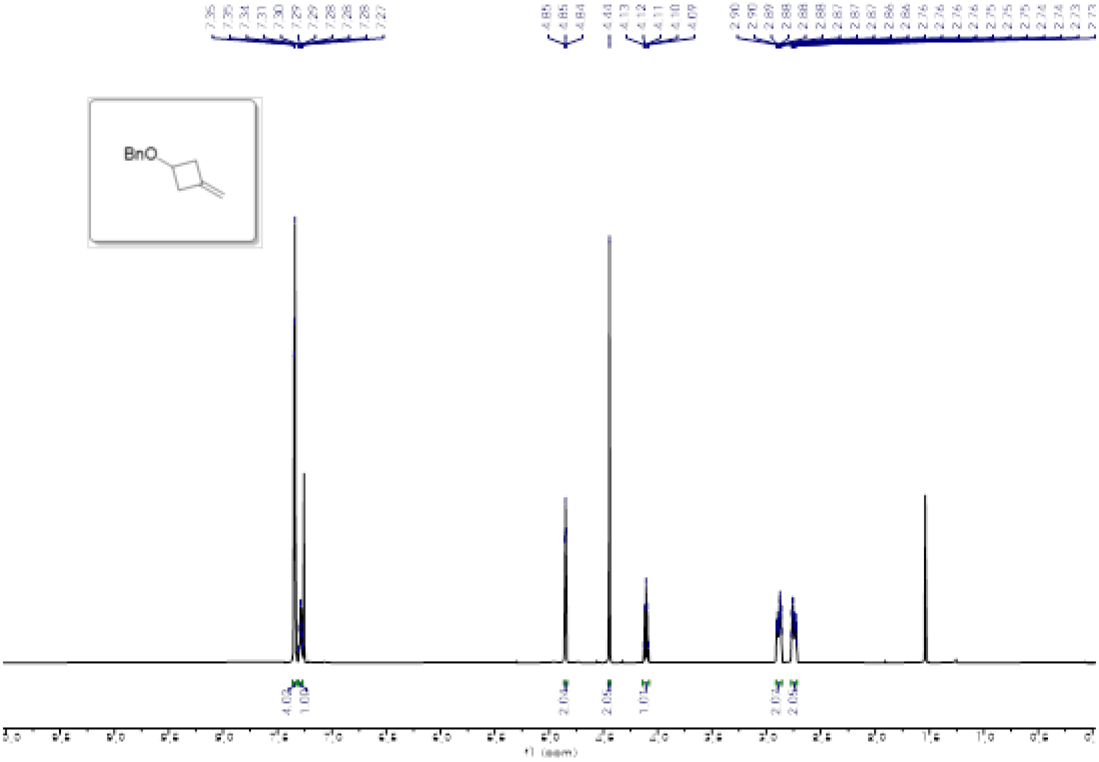

150 MHz, ^{13}C NMR in $CDCl_3$

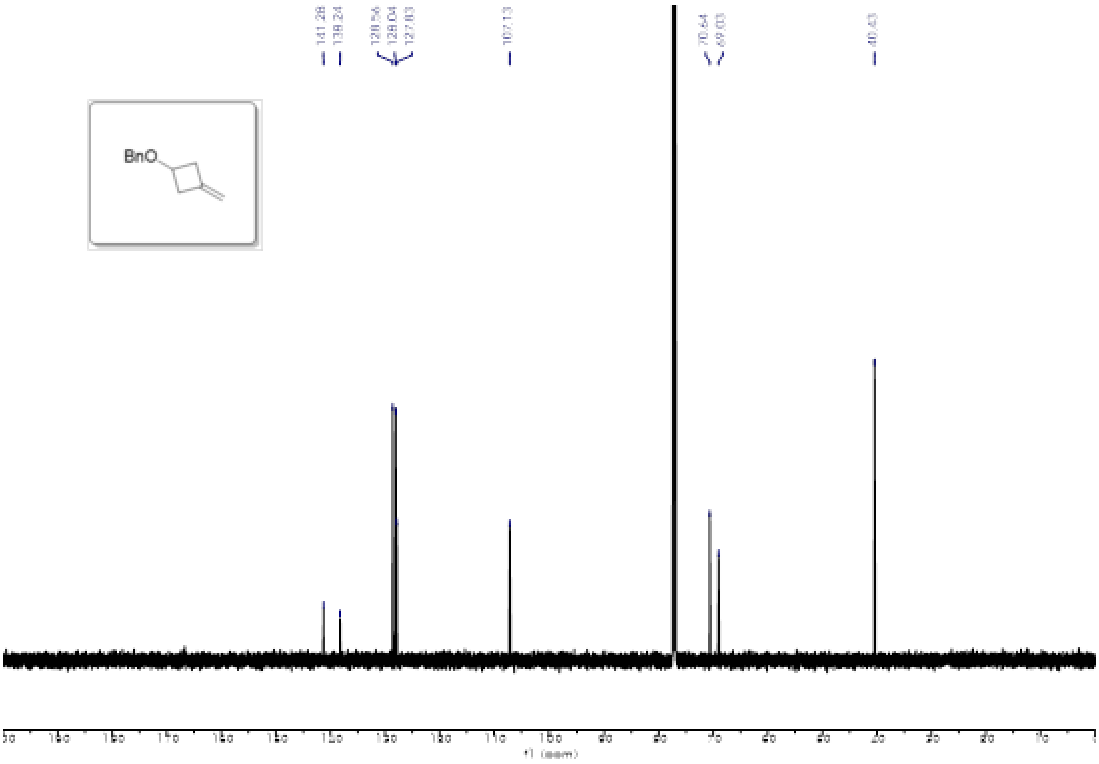

4-(2-methylhexan-2-yl)-2-phenylpyridine (6a).

400 MHz, ^{1}H NMR in $CDCl_3$

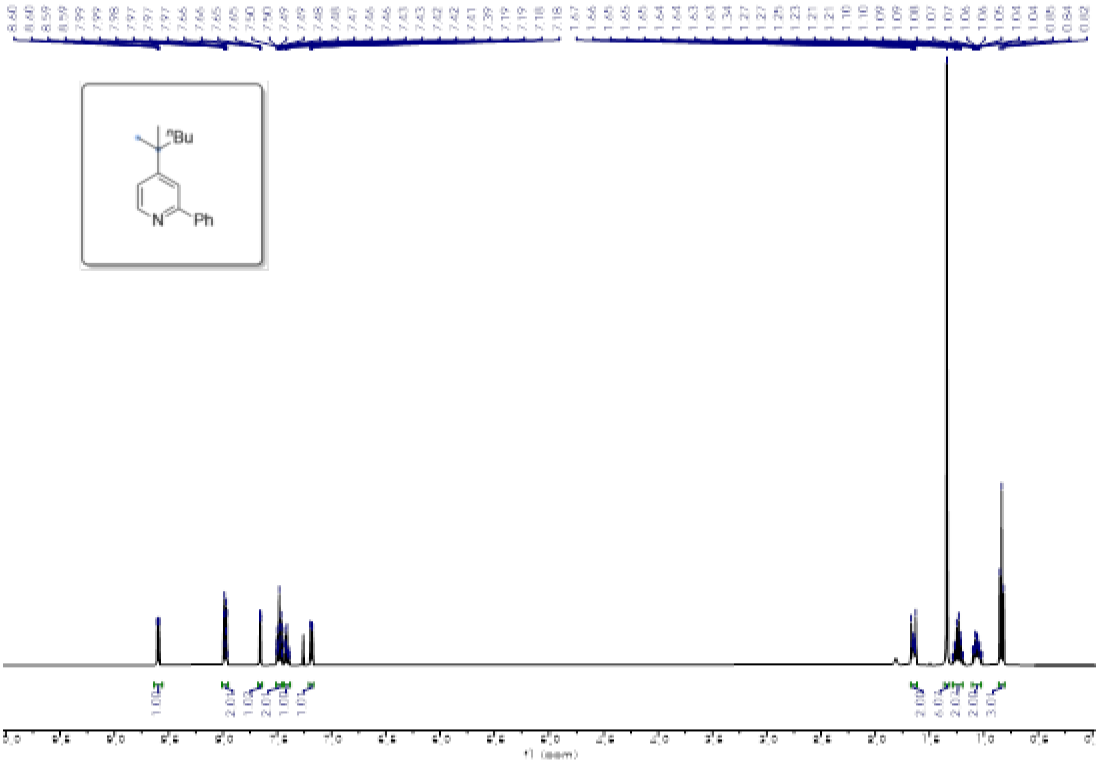

100 MHz, ^{13}C NMR in $CDCl_3$

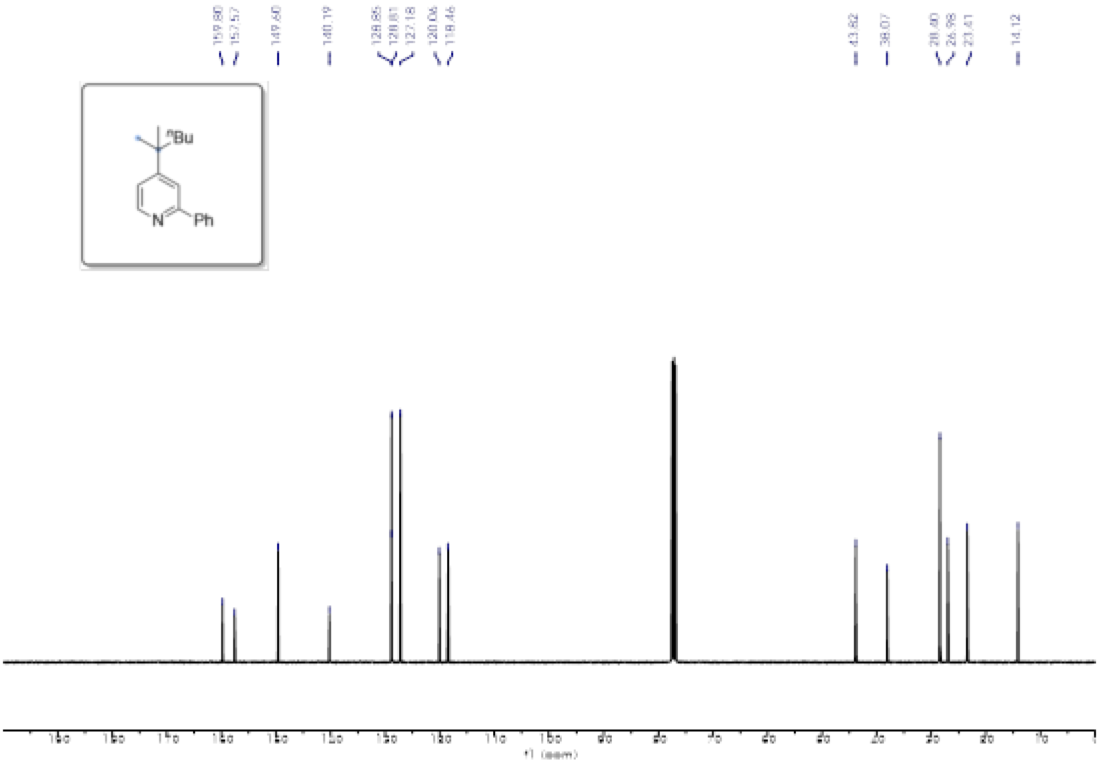

4-(3-methylpentan-3-yl)-2-phenylpyridine (6b).

400 MHz, ^{1}H NMR in $CDCl_3$

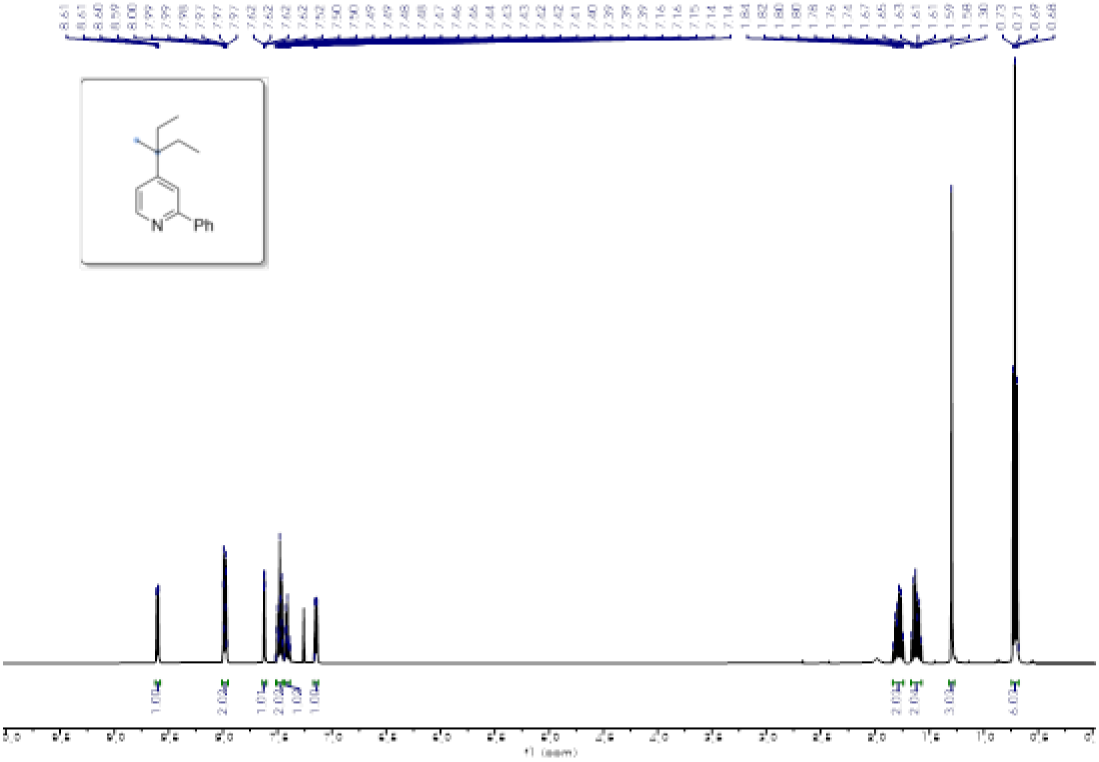

100 MHz, ^{13}C NMR in $CDCl_3$

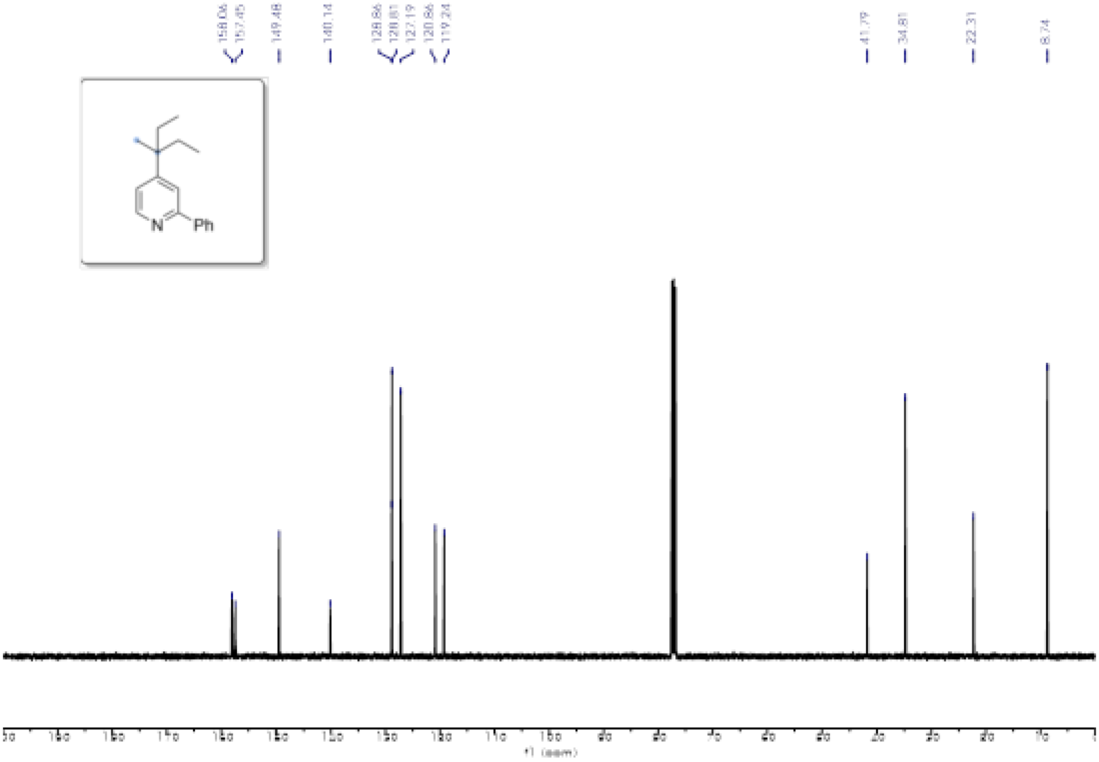

4-(1-methylcyclobutyl)-2-phenylpyridine (6c).

400 MHz, ^{1}H NMR in $CDCl_3$

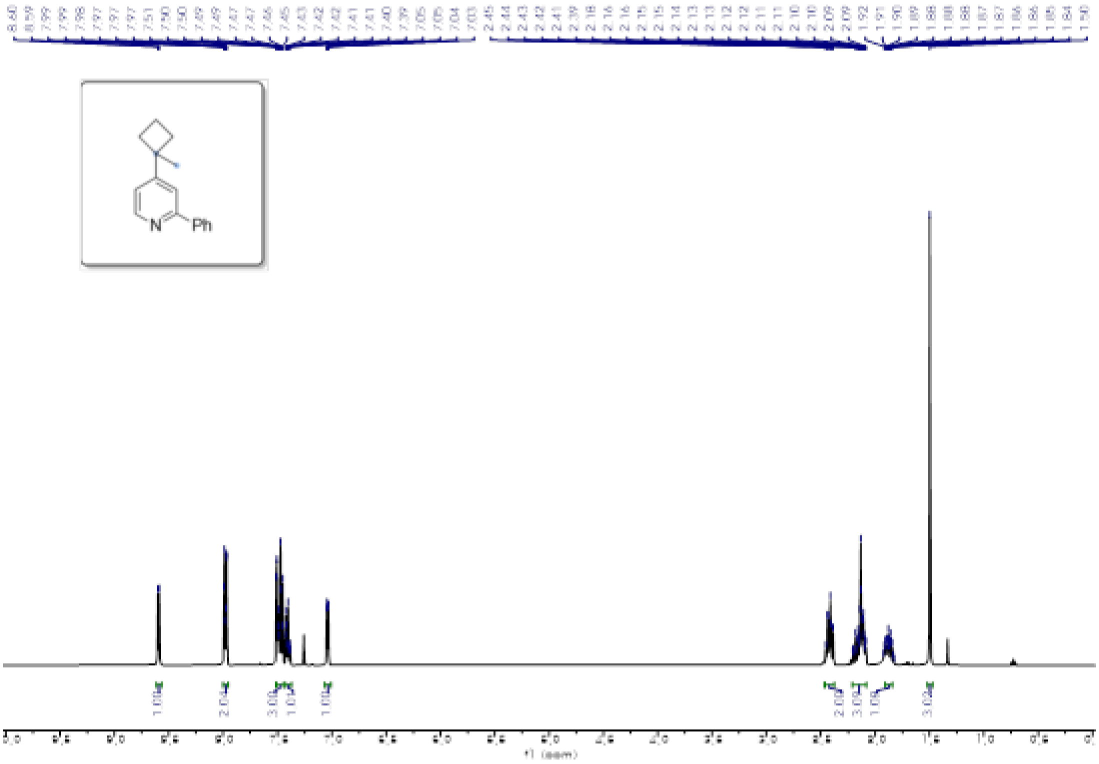

100 MHz, ^{13}C NMR in $CDCl_3$

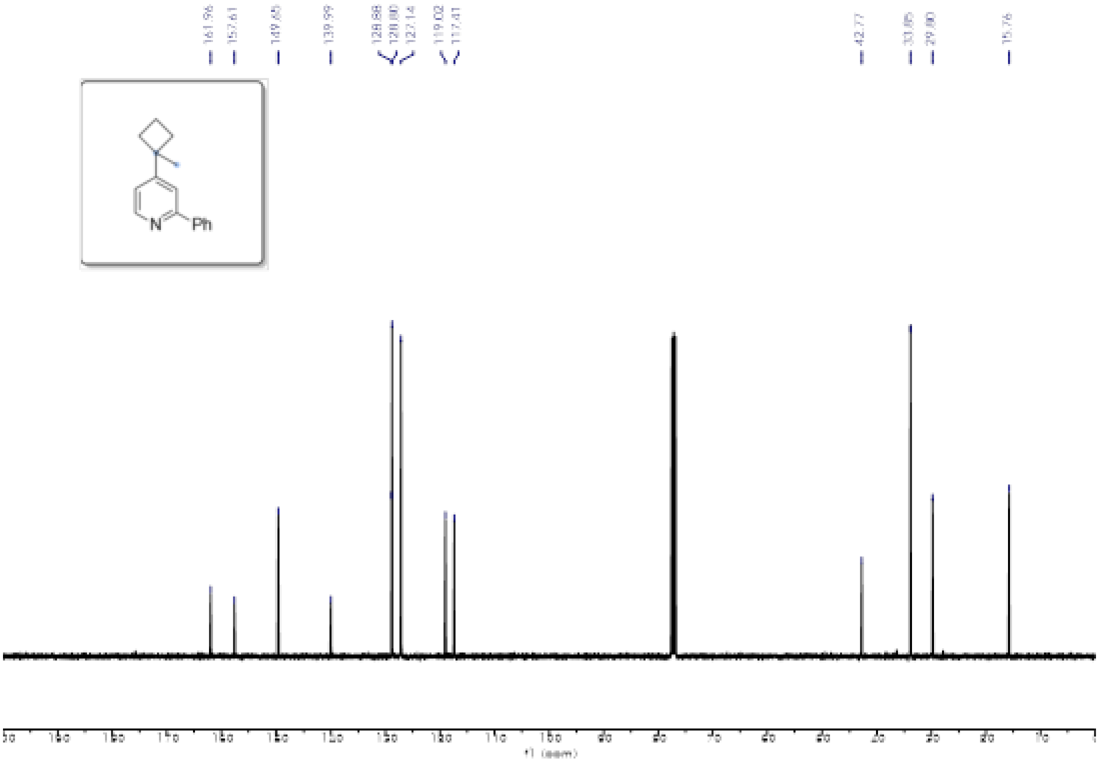

4-(1-methylcyclopentyl)-2-phenylpyridine (6d).

400 MHz, ^{1}H NMR in $CDCl_3$

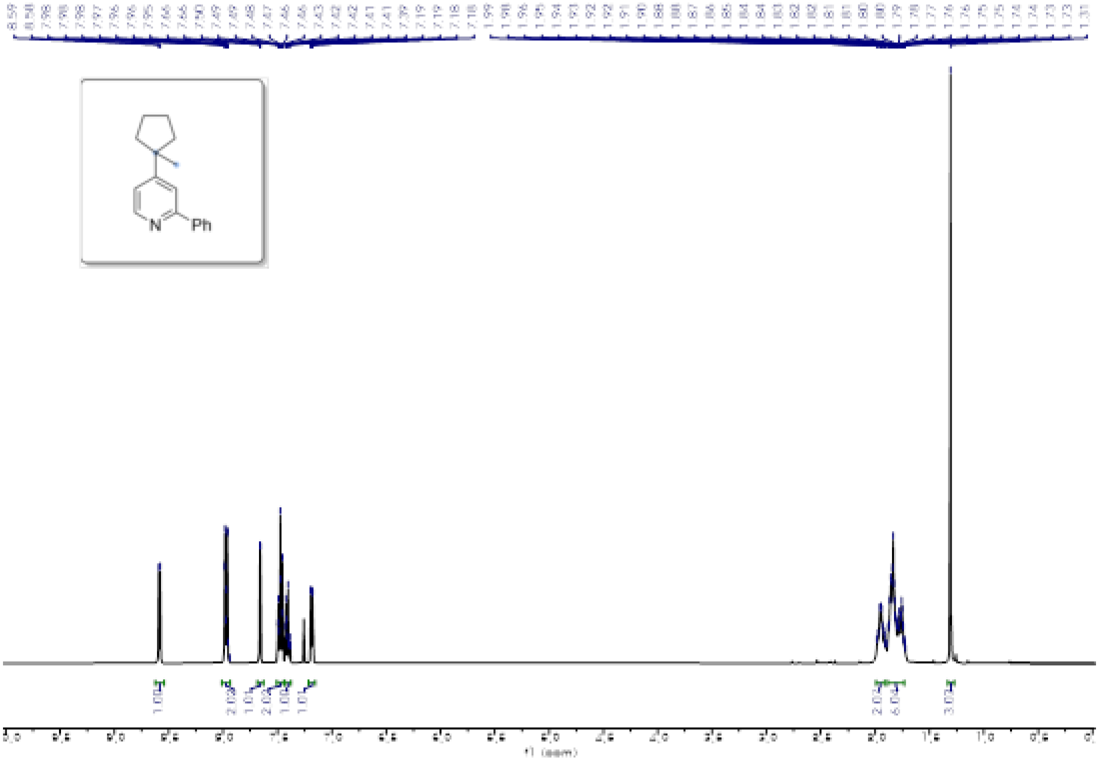

100 MHz, ^{13}C NMR in $CDCl_3$

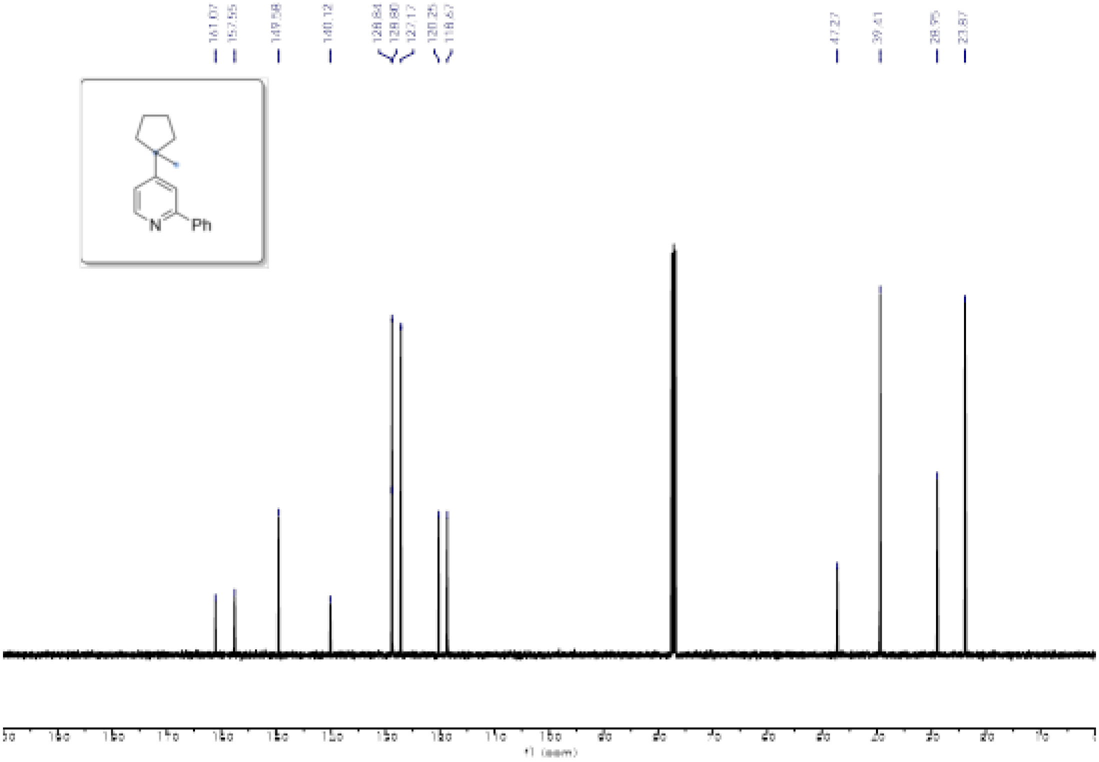

2-methyl-2-(2-phenylpyridin-4-yl)propan-1-ol (6e).

400 MHz, ^{1}H NMR in $CDCl_3$

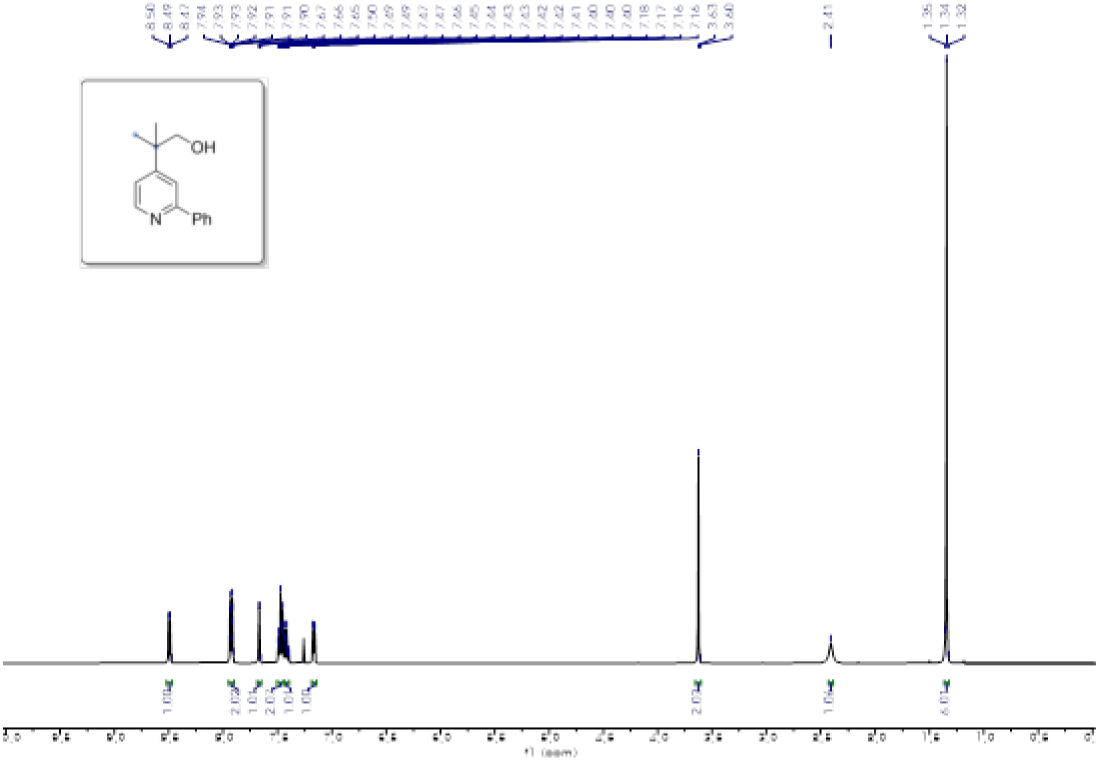

100 MHz, ^{13}C NMR in $CDCl_3$

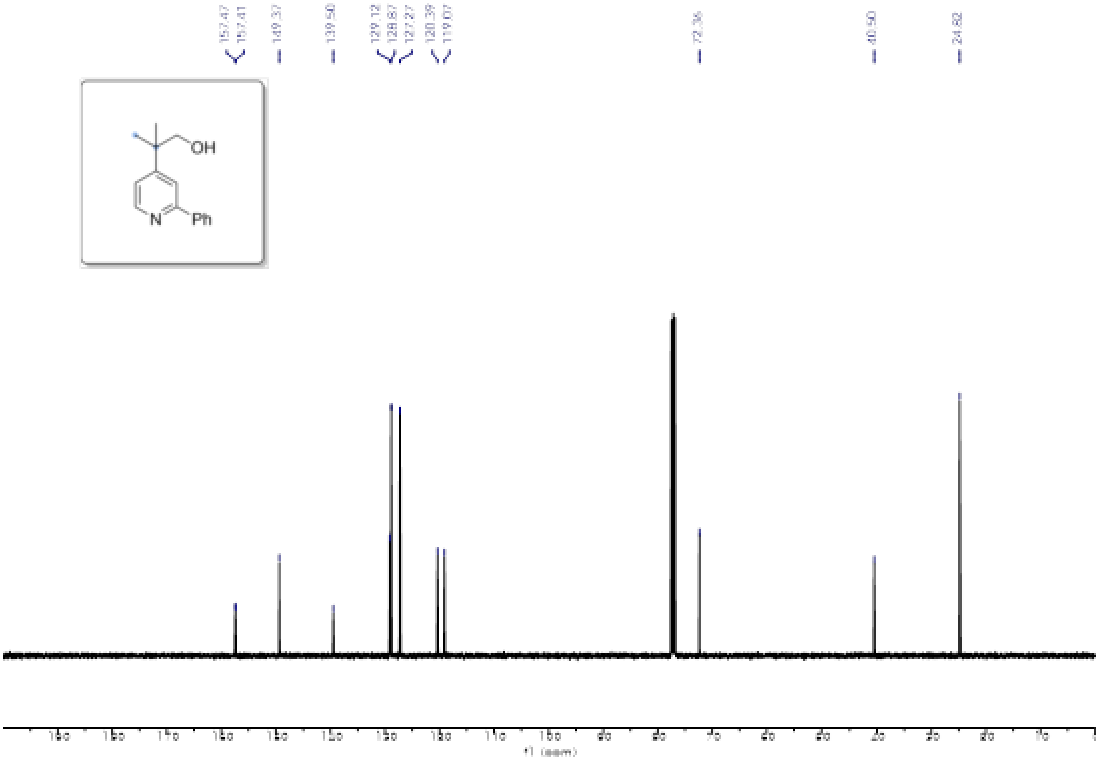

1-(4-methyl-4-(2-phenylpyridin-4-yl)piperidin-1-yl)ethan-1-one (6f).

400 MHz, ^{1}H NMR in $CDCl_3$

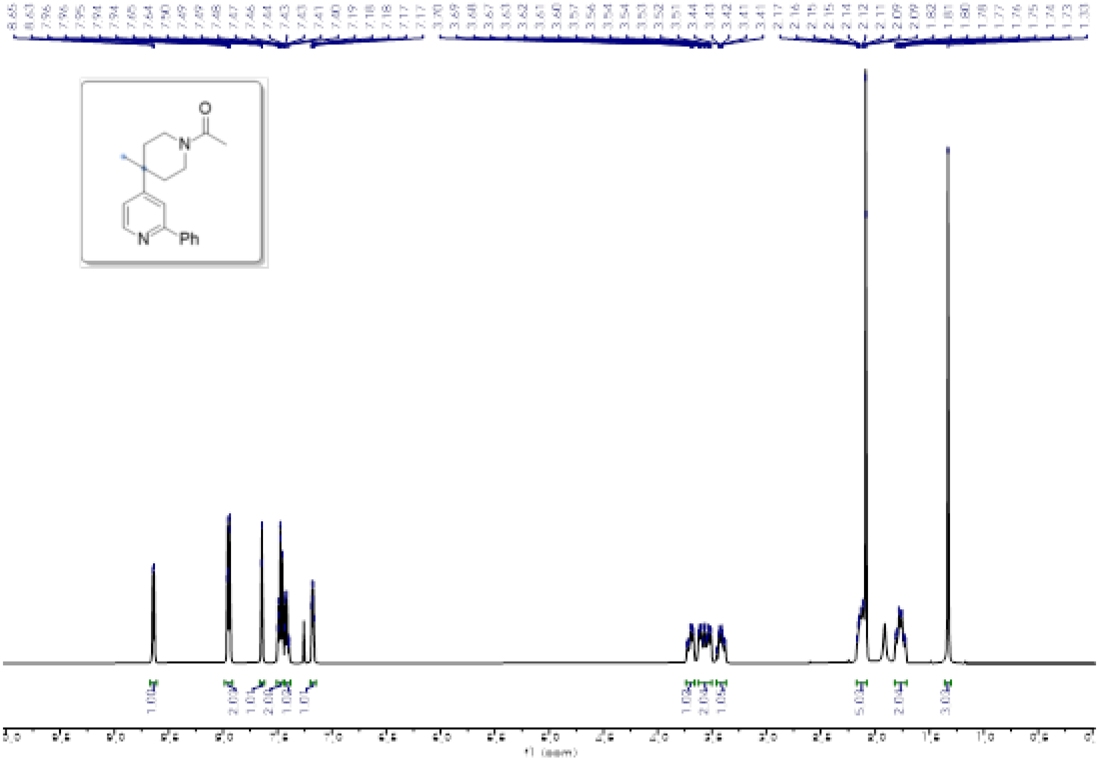

100 MHz, ^{13}C NMR in $CDCl_3$

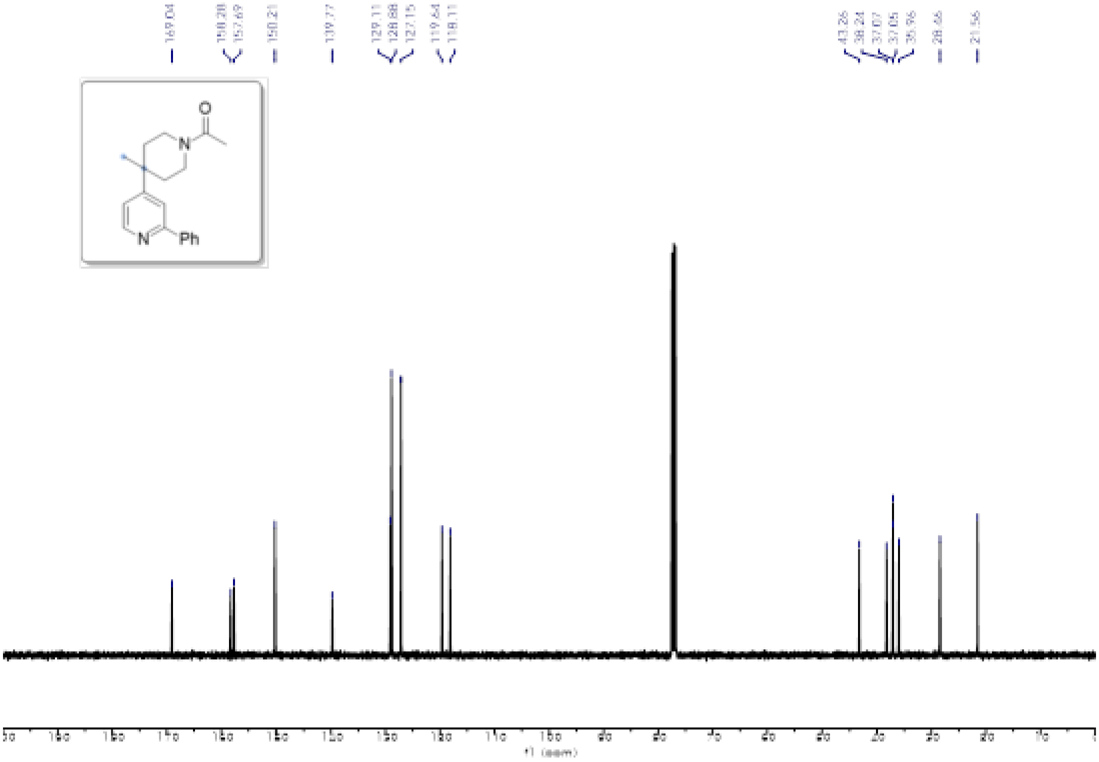

ethyl 4-methyl-4-(2-phenylpyridin-4-yl)pentanoate (6g).

400 MHz, ^{1}H NMR in $CDCl_3$

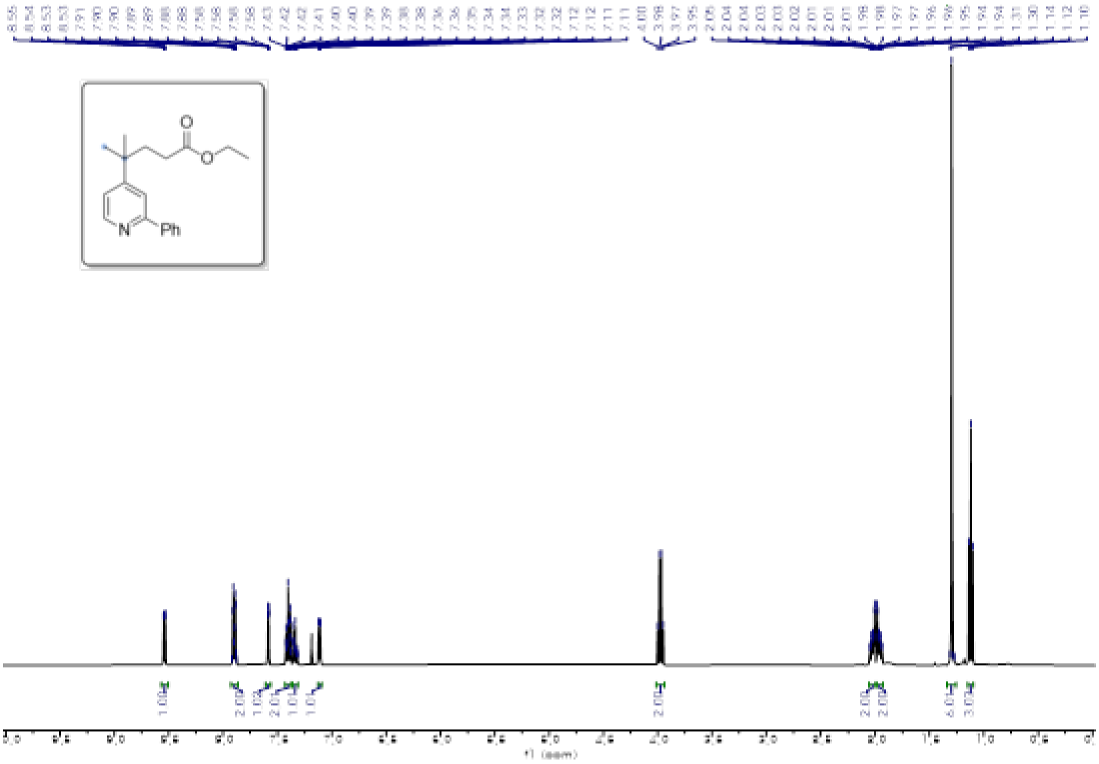

100 MHz, ^{13}C NMR in $CDCl_3$

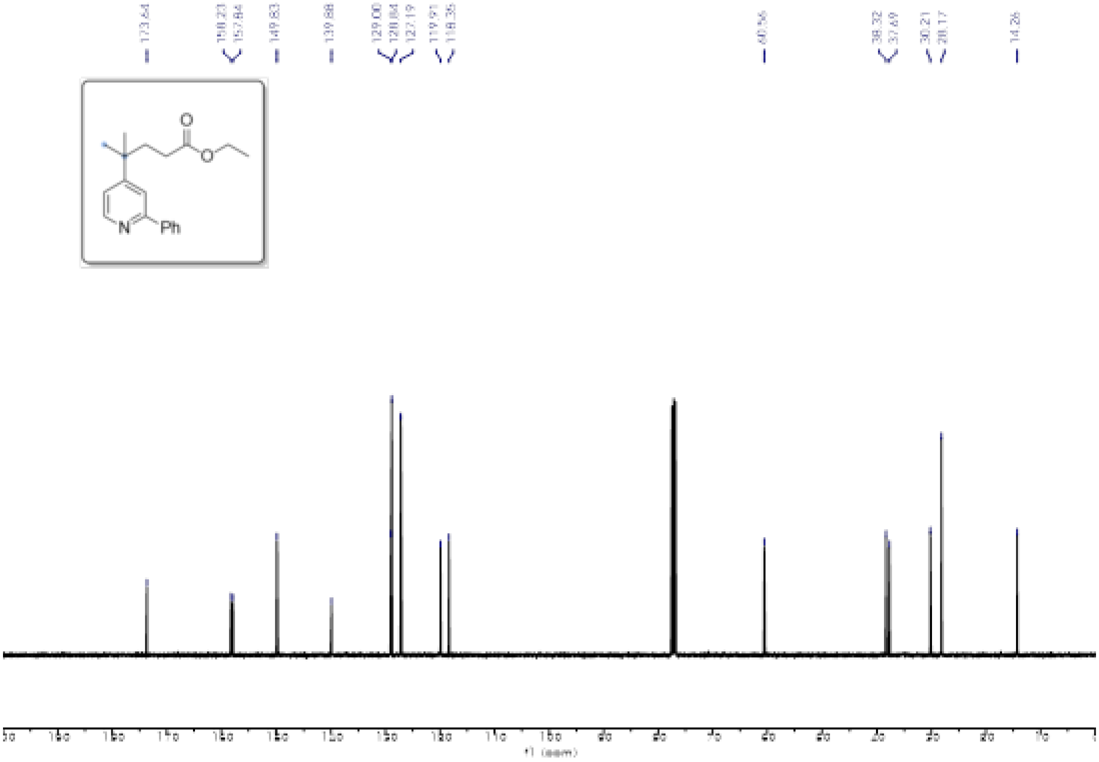

5-methyl-5-(2-phenylpyridin-4-yl)hexan-2-one (6h).

400 MHz, ^{1}H NMR in $CDCl_3$

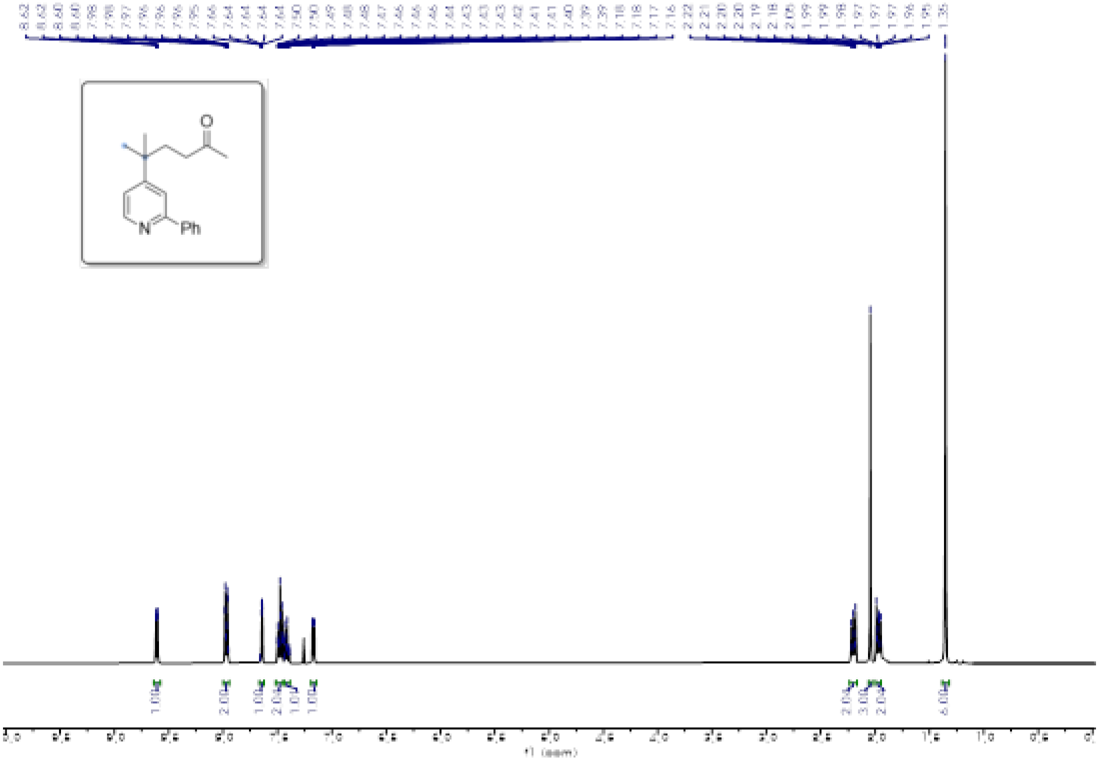

100 MHz, ^{13}C NMR in $CDCl_3$

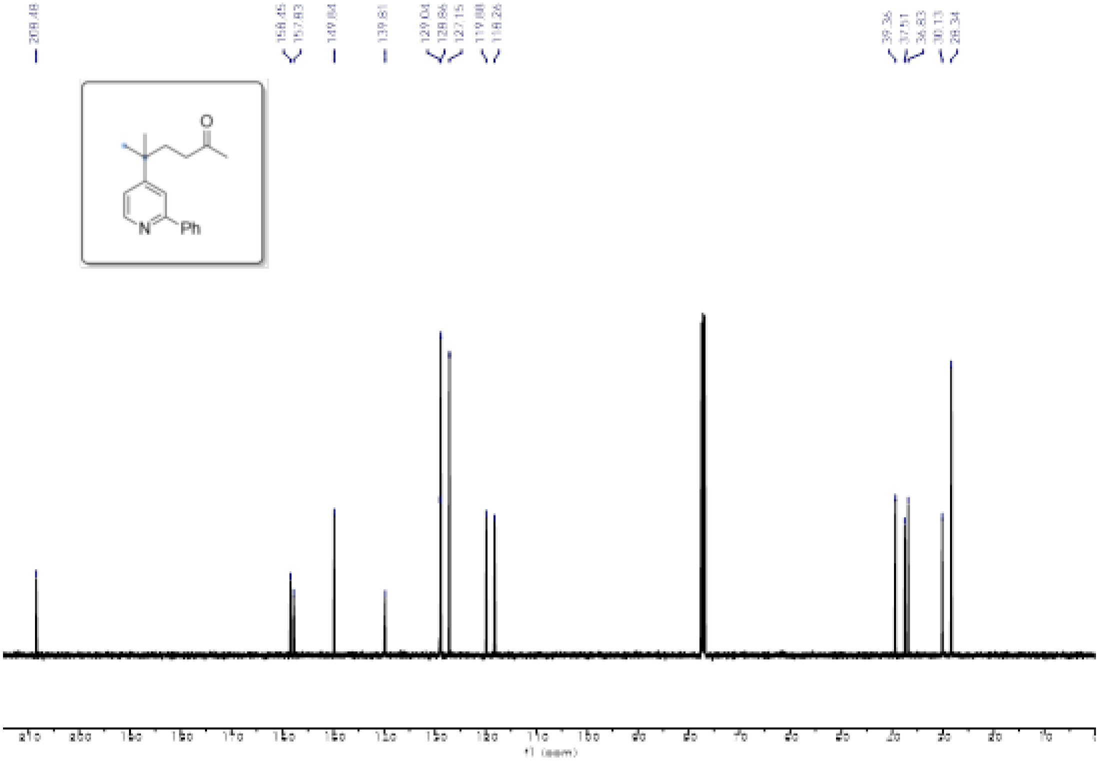

4-(4-methylpentan-2-yl)-2-phenylpyridine (6i).

500 MHz, ^{1}H NMR in $CDCl_3$

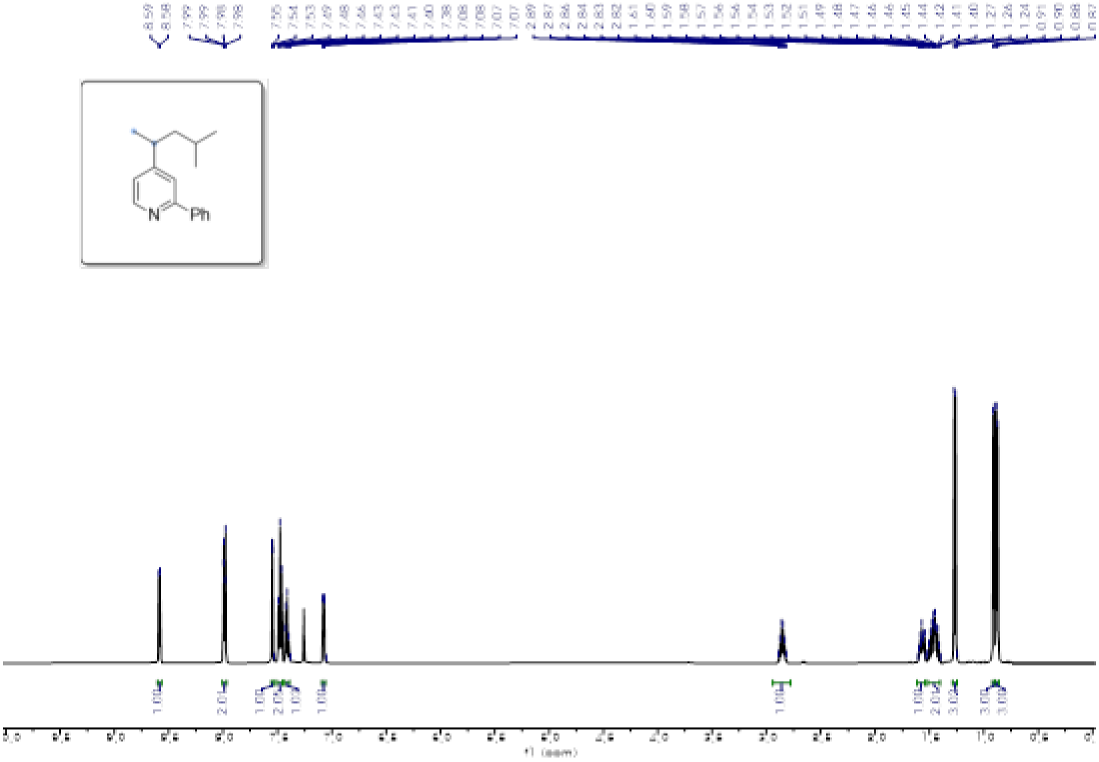

100 MHz, ^{13}C NMR in $CDCl_3$

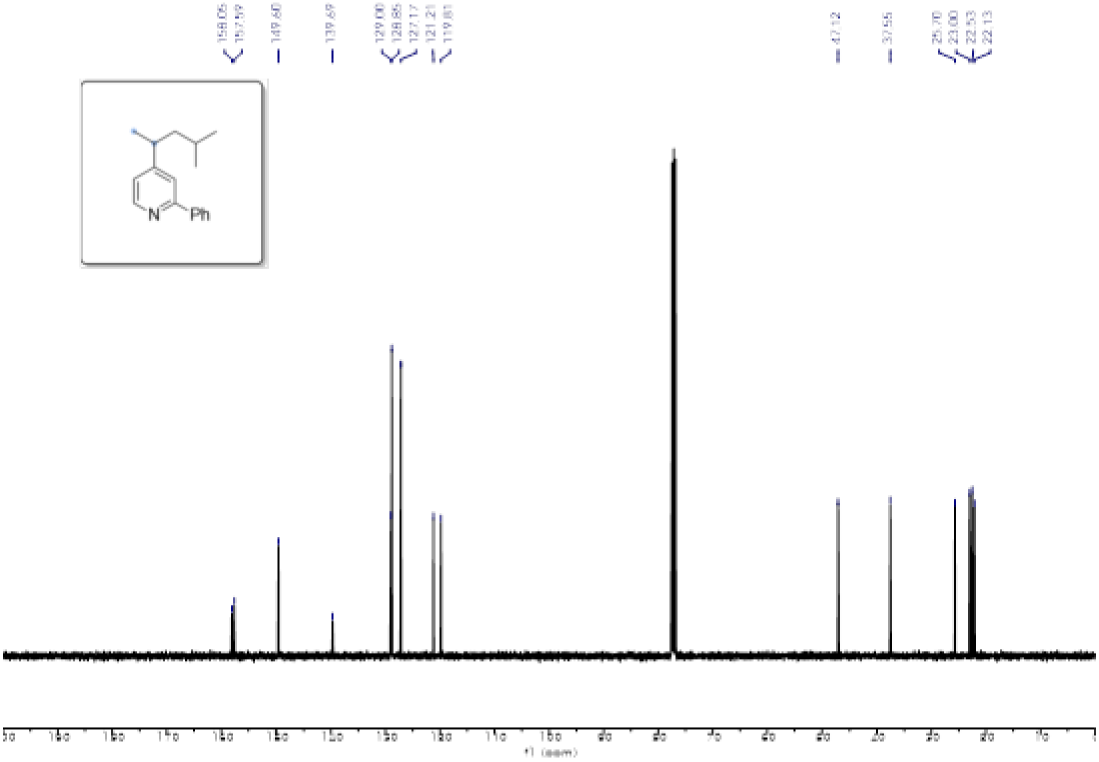

2-phenyl-4-(4-phenylbutan-2-yl)pyridine (6j).

500 MHz, ^{1}H NMR in CD_2Cl_2

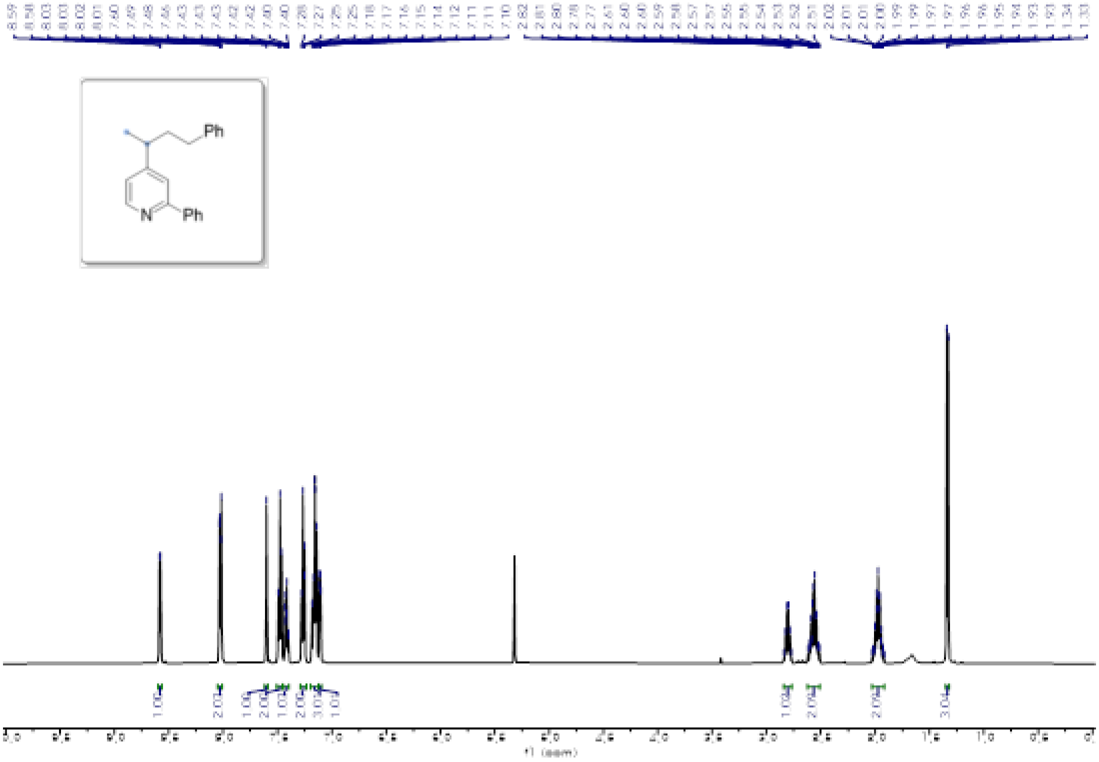

100 MHz, ^{13}C NMR in CD_2Cl_2

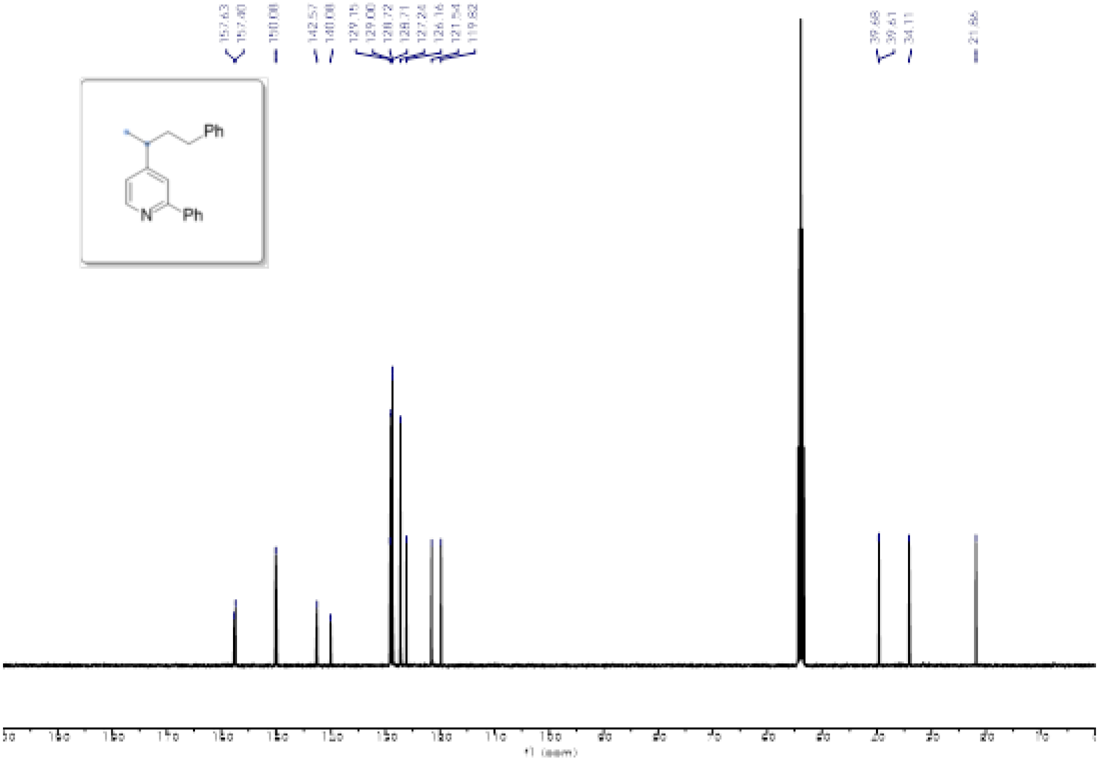

4-(4-(2-bromophenyl)butan-2-yl)-2-phenylpyridine (6k).

600 MHz, ^{1}H NMR in $CDCl_3$

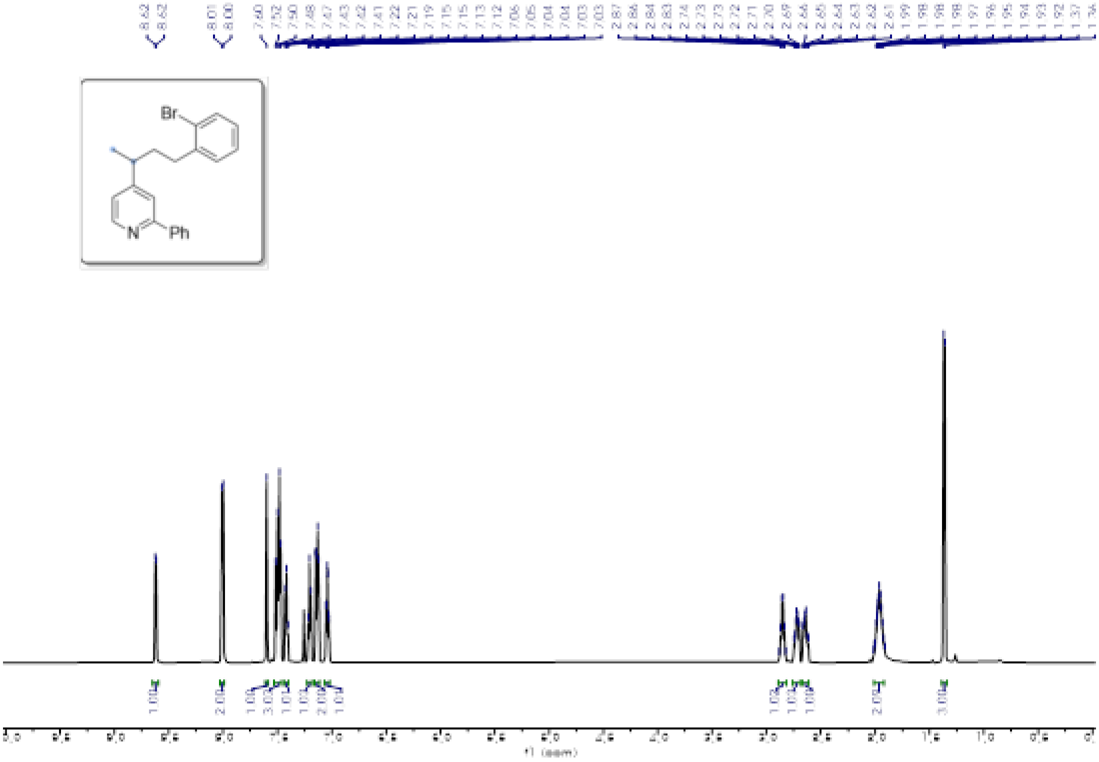

100 MHz, ^{13}C NMR in $CDCl_3$

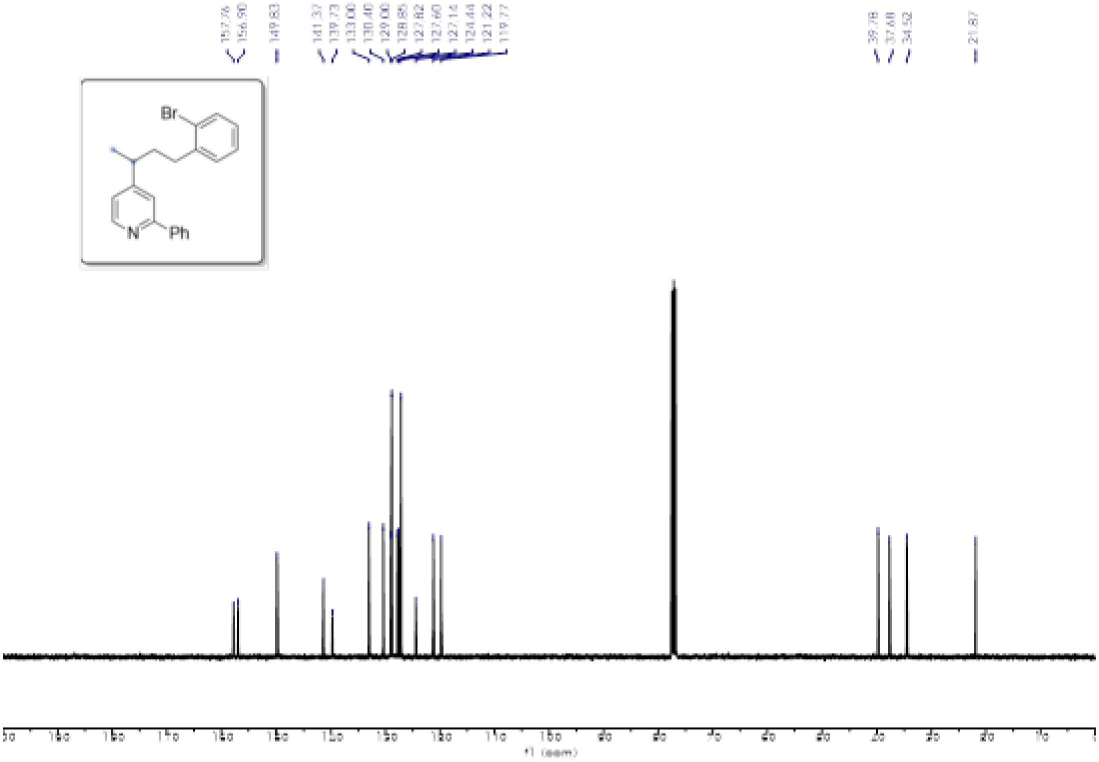

2-methyl-3-(2-phenylpyridin-4-yl)butan-2-ol (6l).

600 MHz, ^{1}H NMR in $CDCl_3$

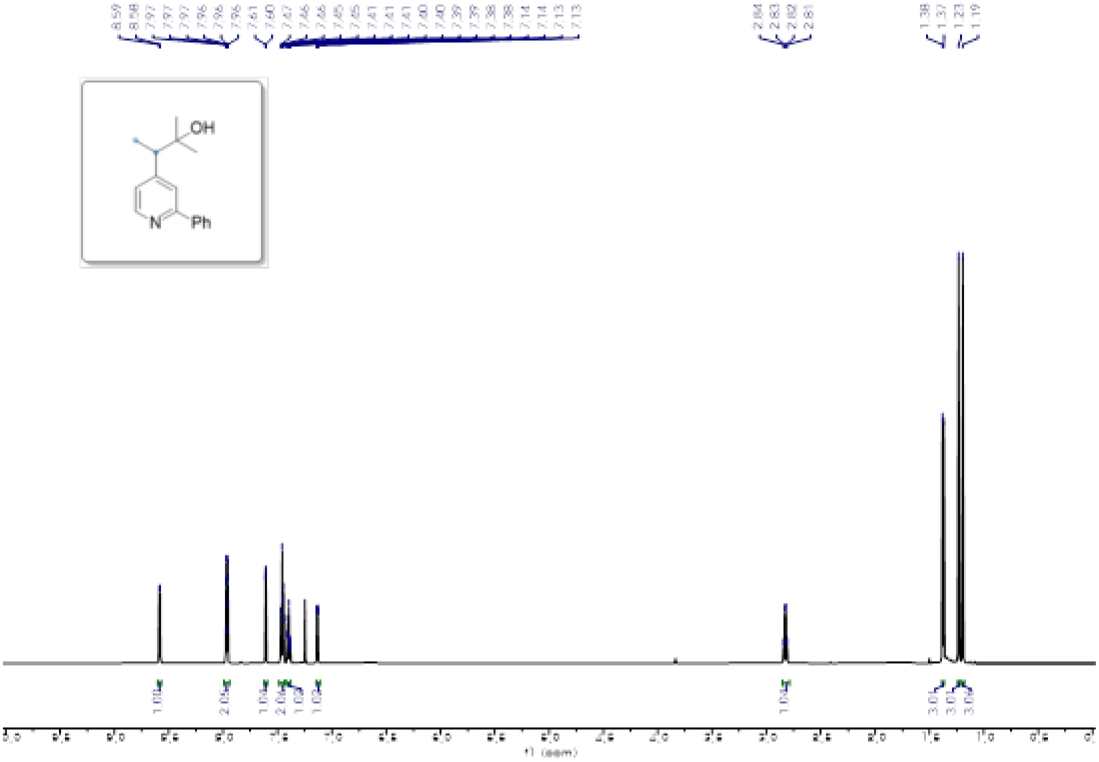

100 MHz, ^{13}C NMR in $CDCl_3$

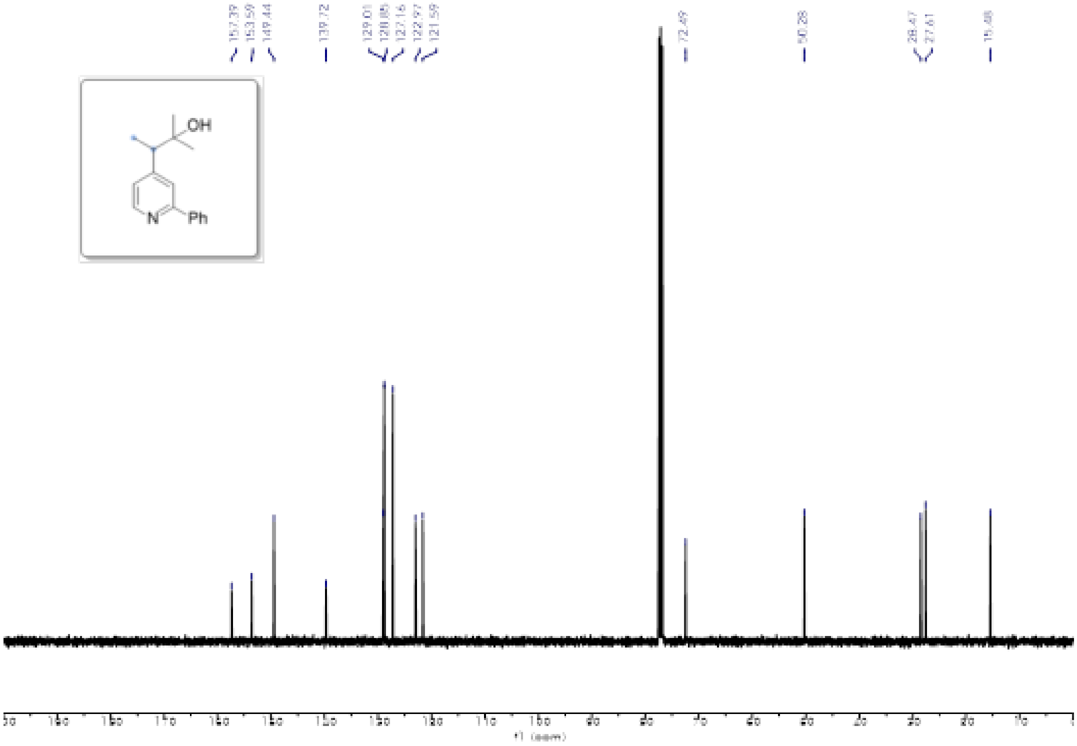

4-(2-phenylpyridin-4-yl)pentanoic acid (6m).

400 MHz, ^{1}H NMR in $CDCl_3$

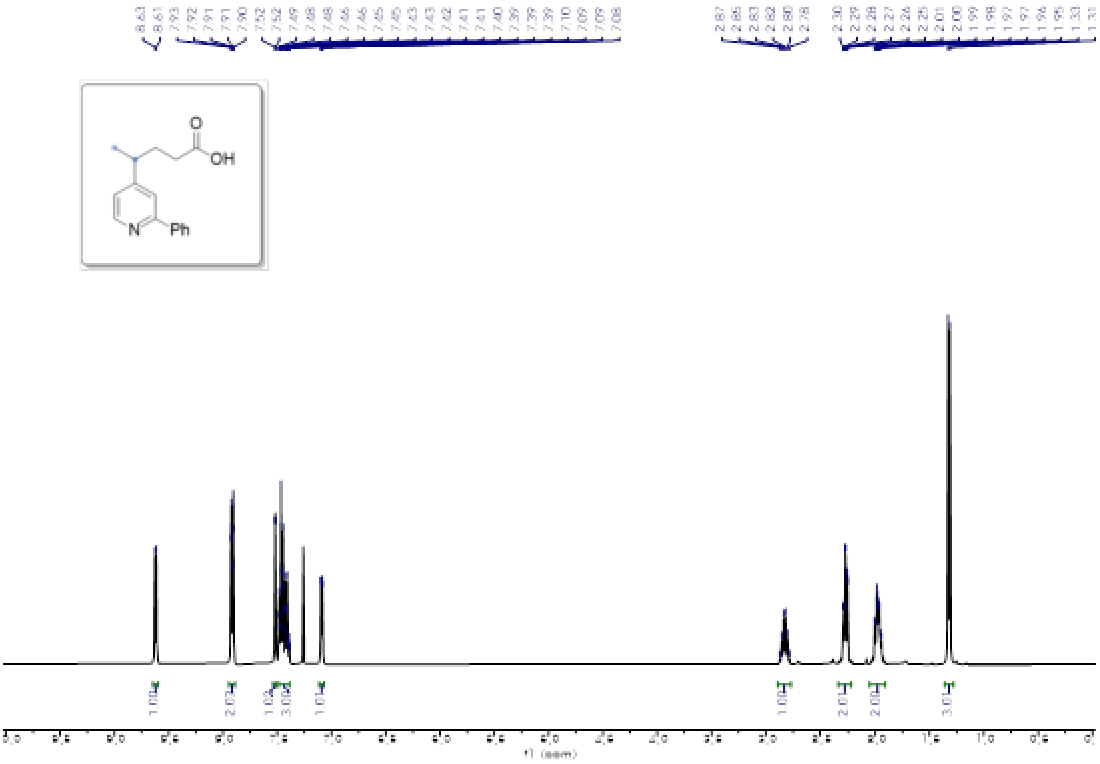

100 MHz, ^{13}C NMR in $CDCl_3$

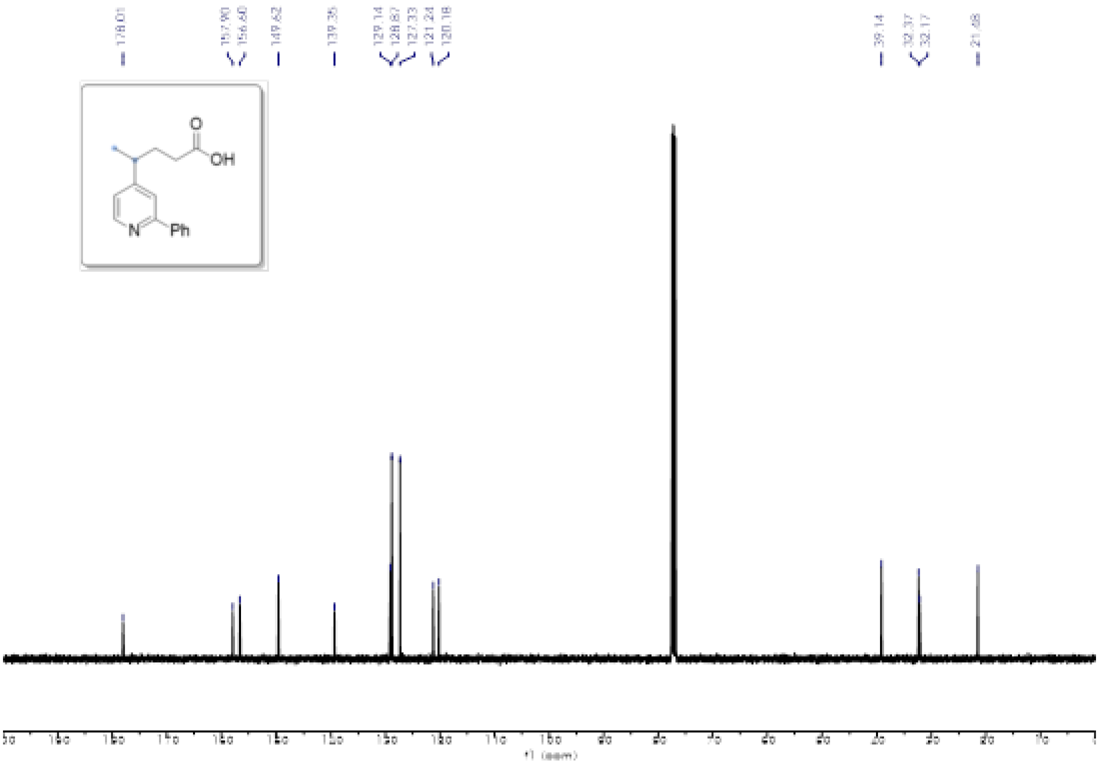

tert-butyl (2-(2-phenylpyridin-4-yl)propyl)carbamate (6n).

500 MHz, ¹H NMR in CDCl$_3$

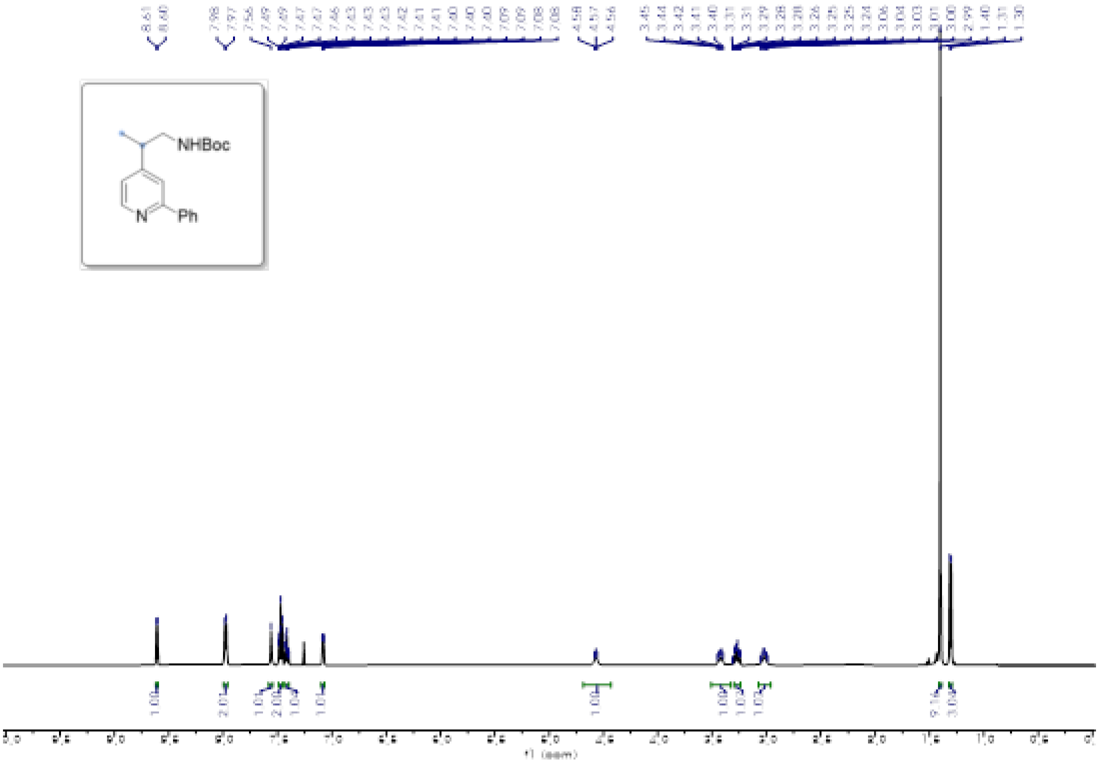

125 MHz, ¹³C NMR in CDCl$_3$

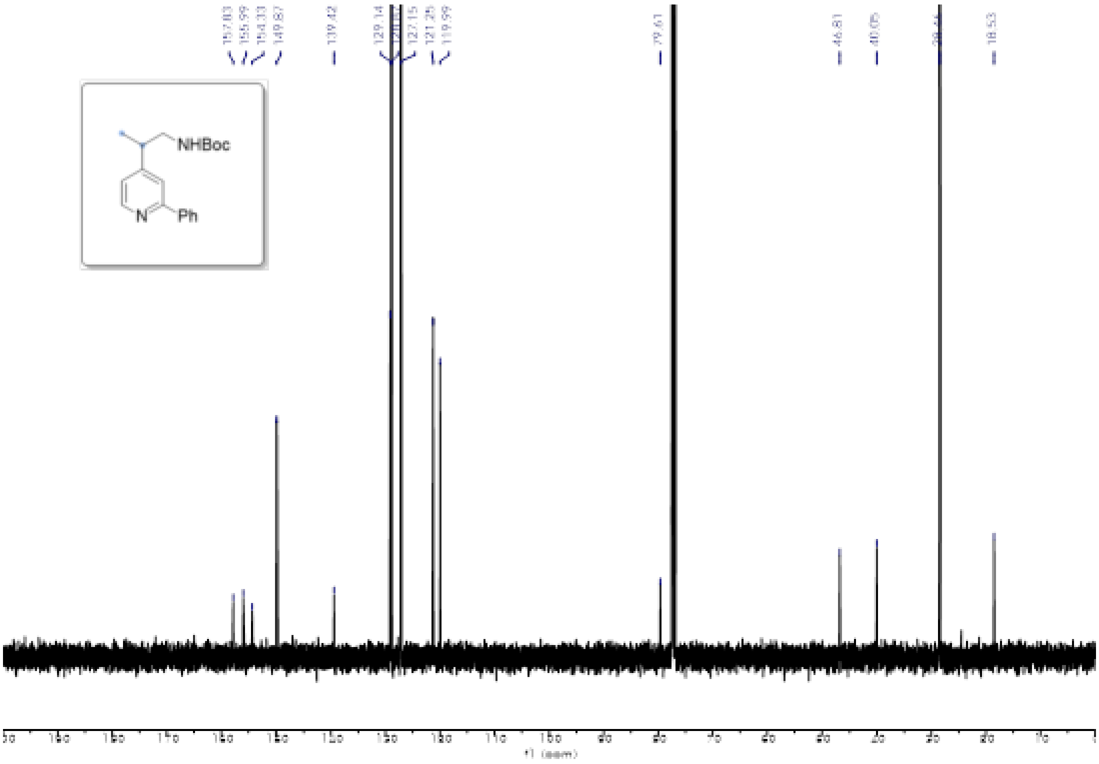

1-(2-(2-phenylpyridin-4-yl)propyl)urea (6o).

400 MHz, ^{1}H NMR in CD_2Cl_2

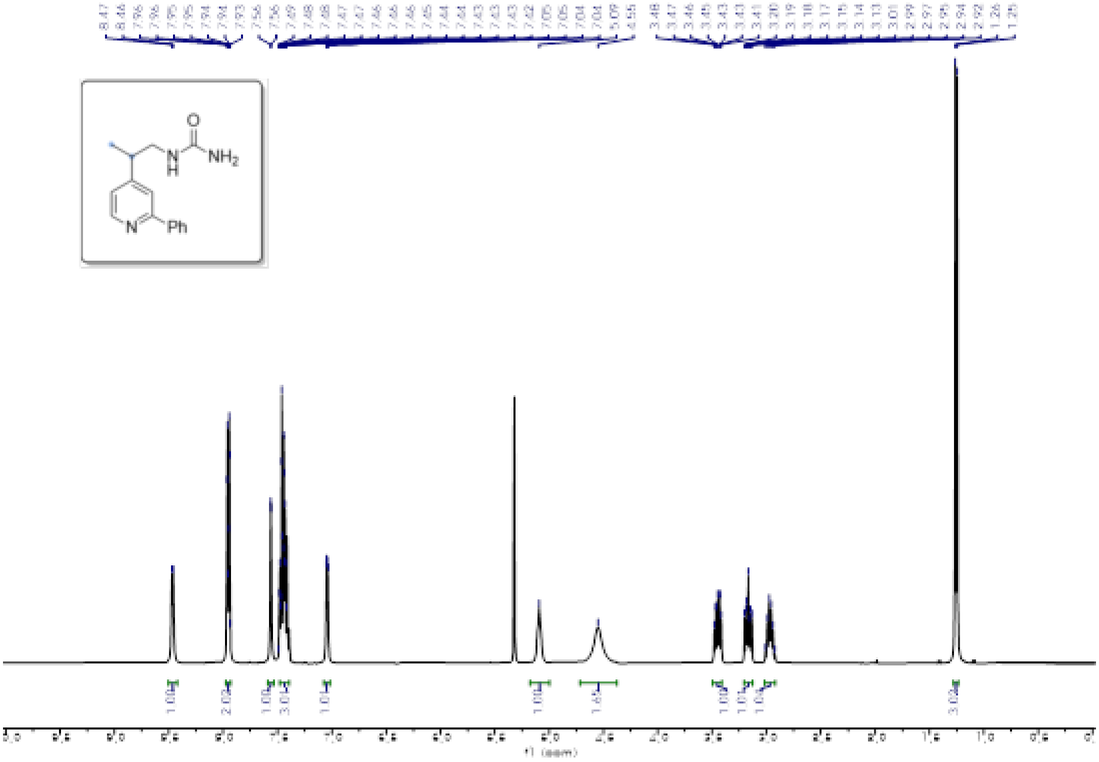

125 MHz, ^{13}C NMR in CD_2Cl_2

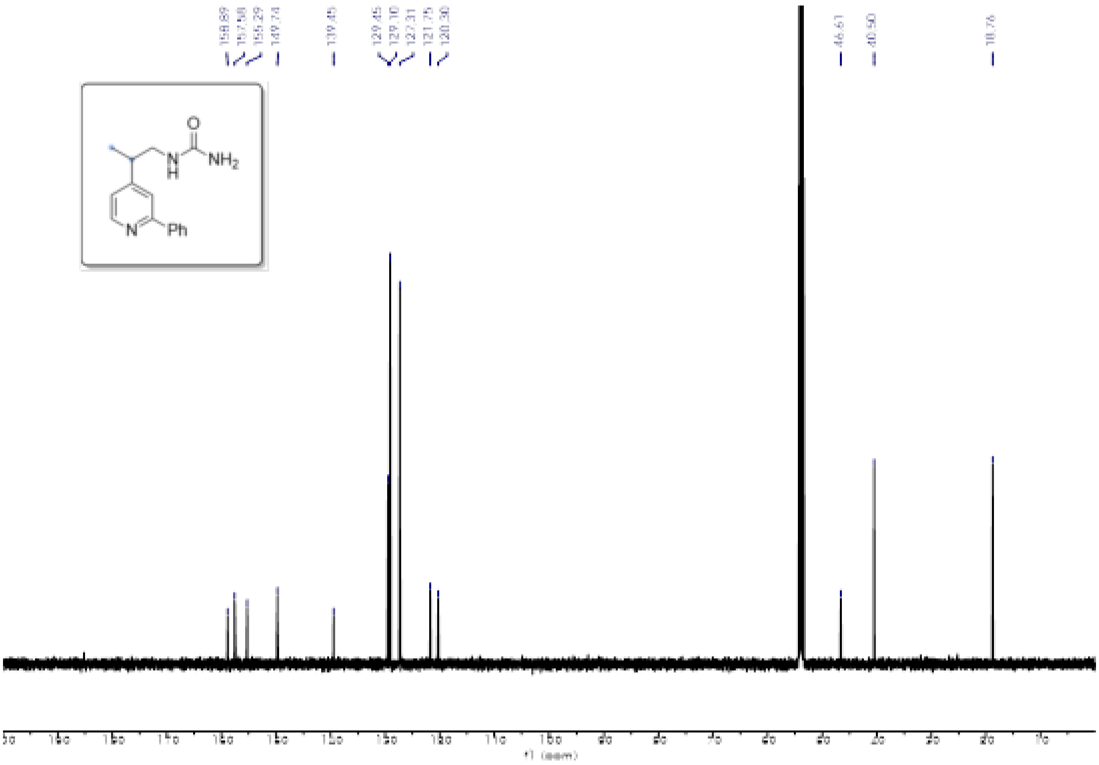

4-(2-phenylpyridin-4-yl)pentanenitrile (6p).

400 MHz, ^{1}H NMR in $CDCl_3$

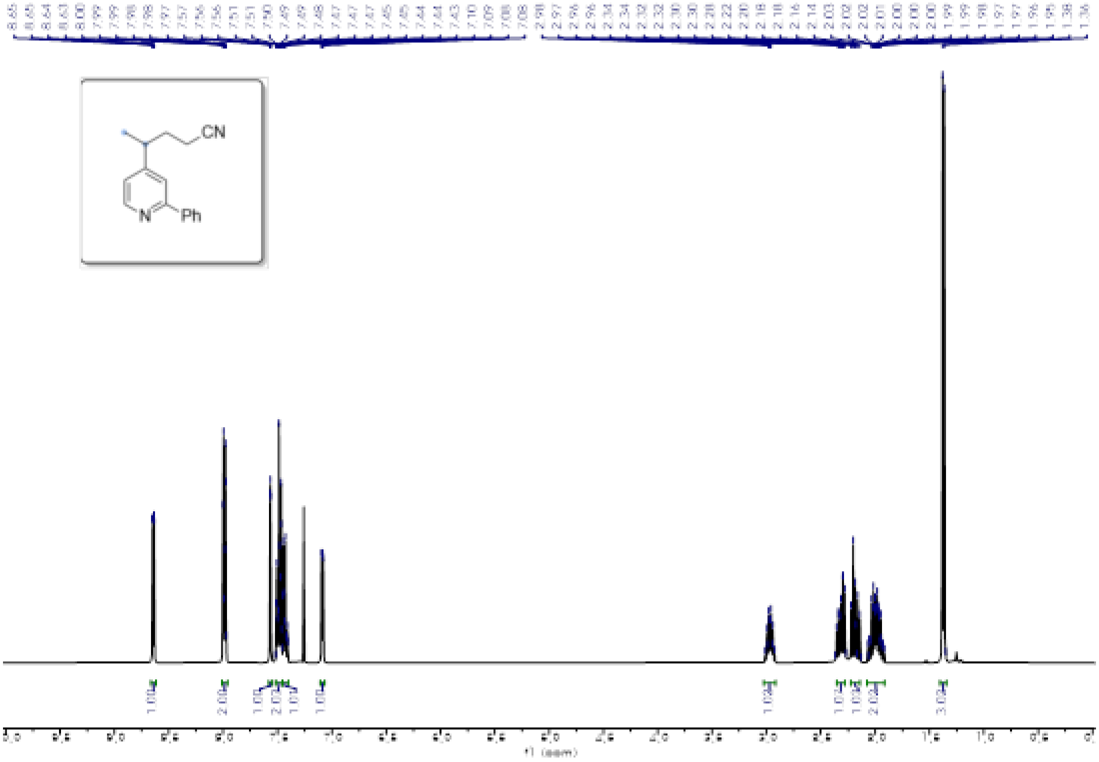

100 MHz, ^{13}C NMR in $CDCl_3$

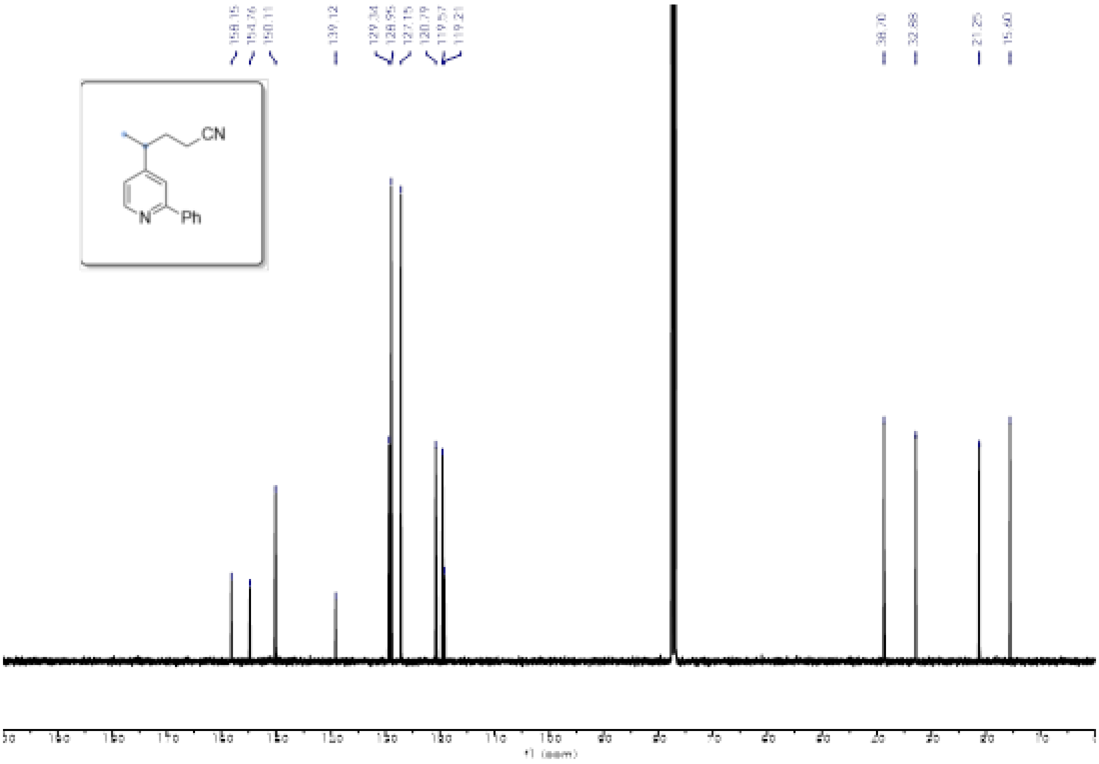

2-phenyl-4-(1-(trimethylsilyl)propan-2-yl)pyridine (6q).

400 MHz, ^{1}H NMR in $CDCl_3$

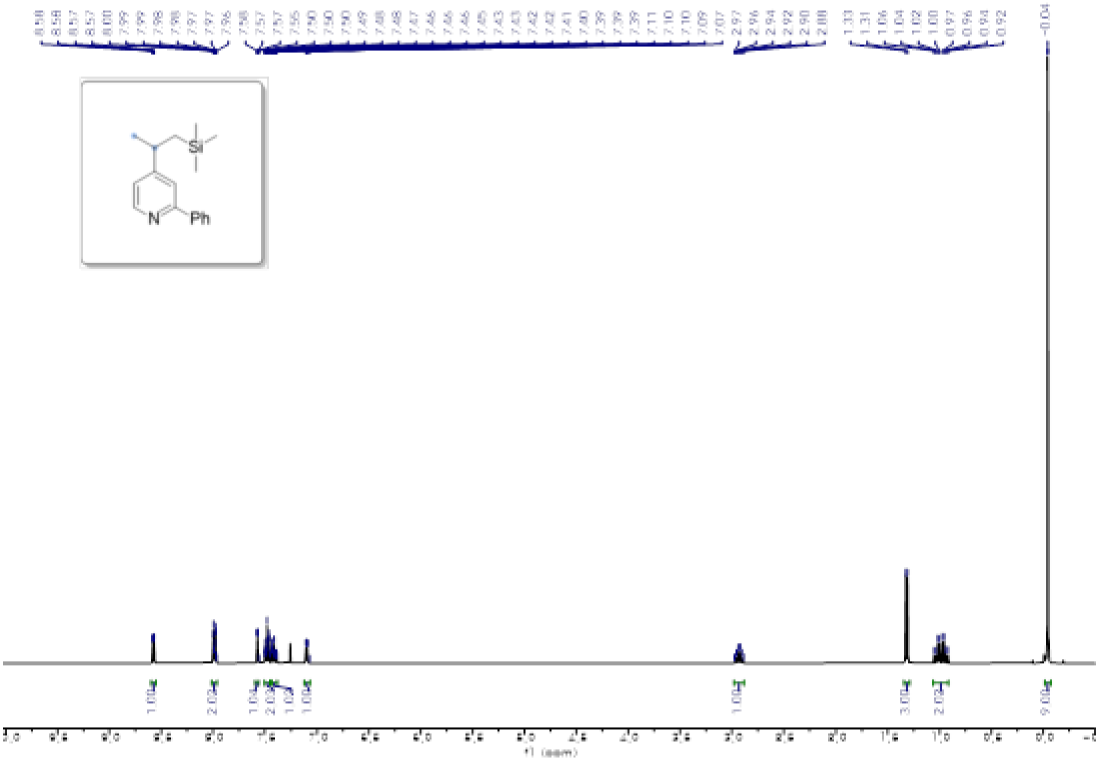

100 MHz, ^{13}C NMR in $CDCl_3$

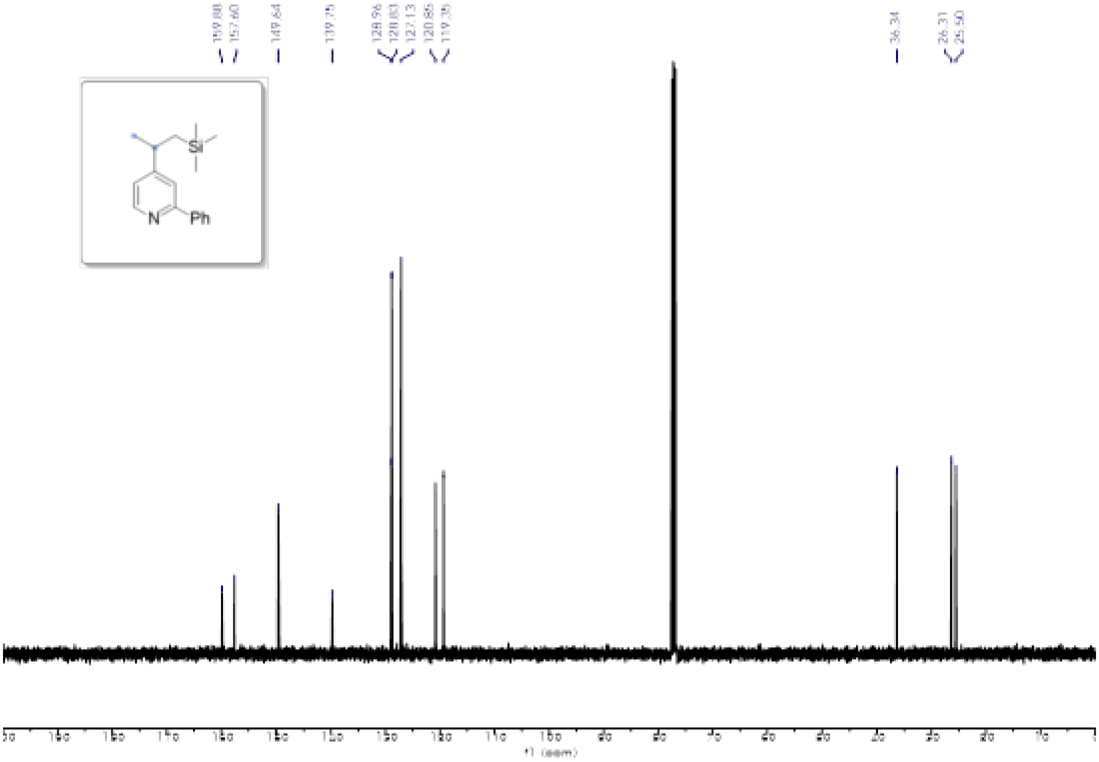

4-(hex-5-yn-2-yl)-2-phenylpyridine (6r).

400 MHz, ^1H NMR in $CDCl_3$

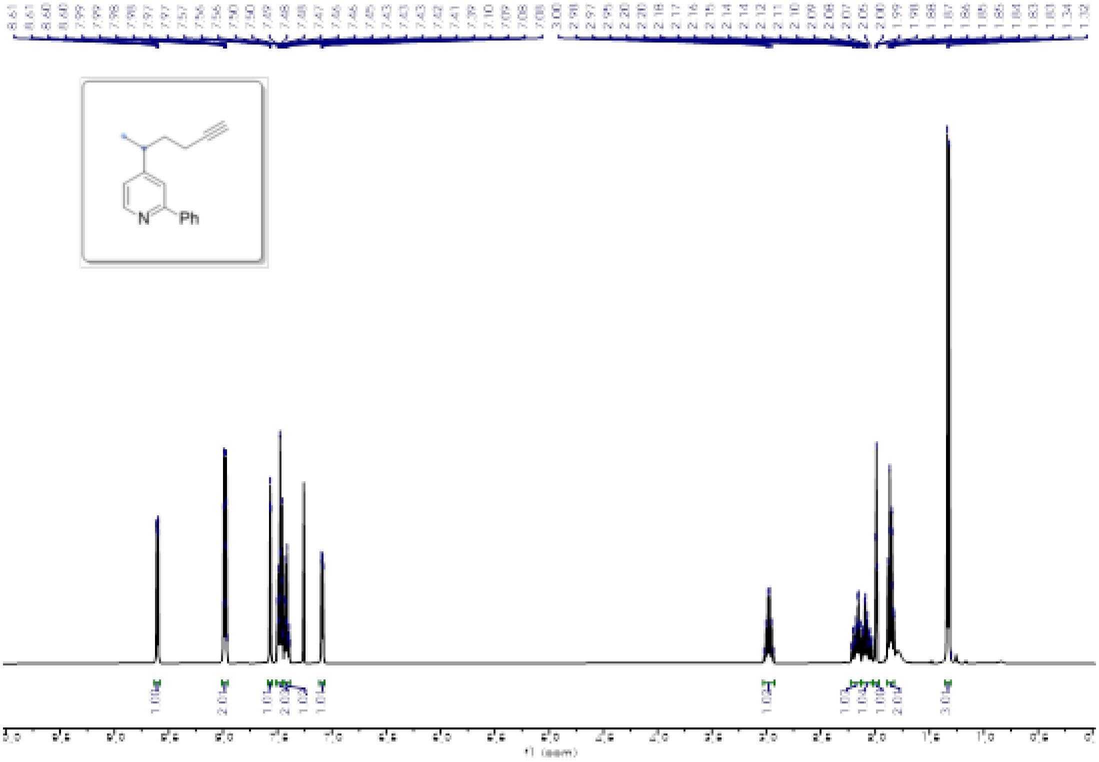

100 MHz, ^{13}C NMR in $CDCl_3$

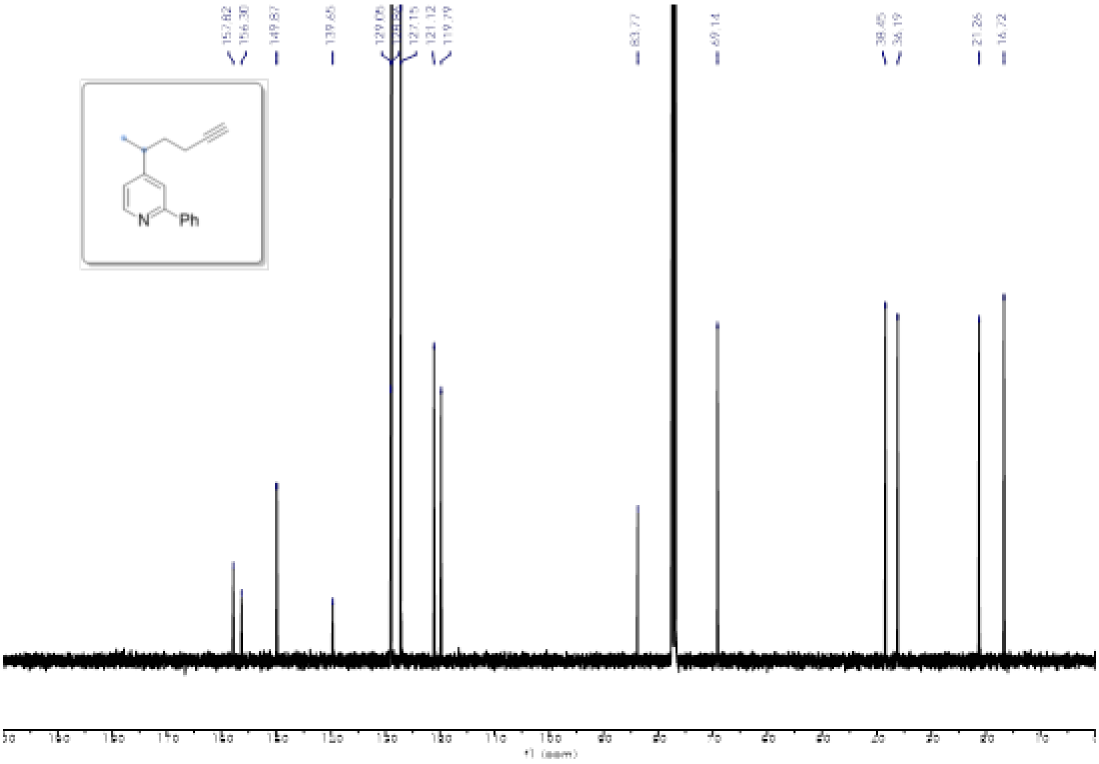

4-(2-phenylpyridin-4-yl)pentyl 4-methoxybenzoate (6s).

500 MHz, ^{1}H NMR in CD_2Cl_2

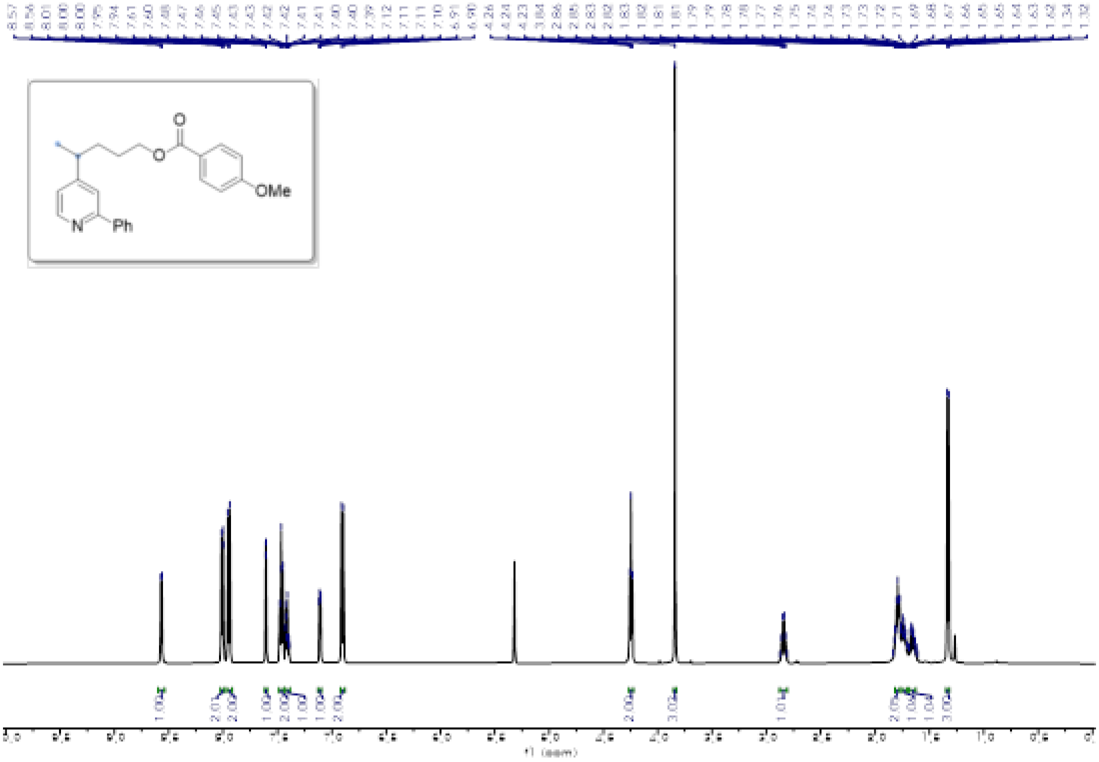

100 MHz, ^{13}C NMR in CD_2Cl_2

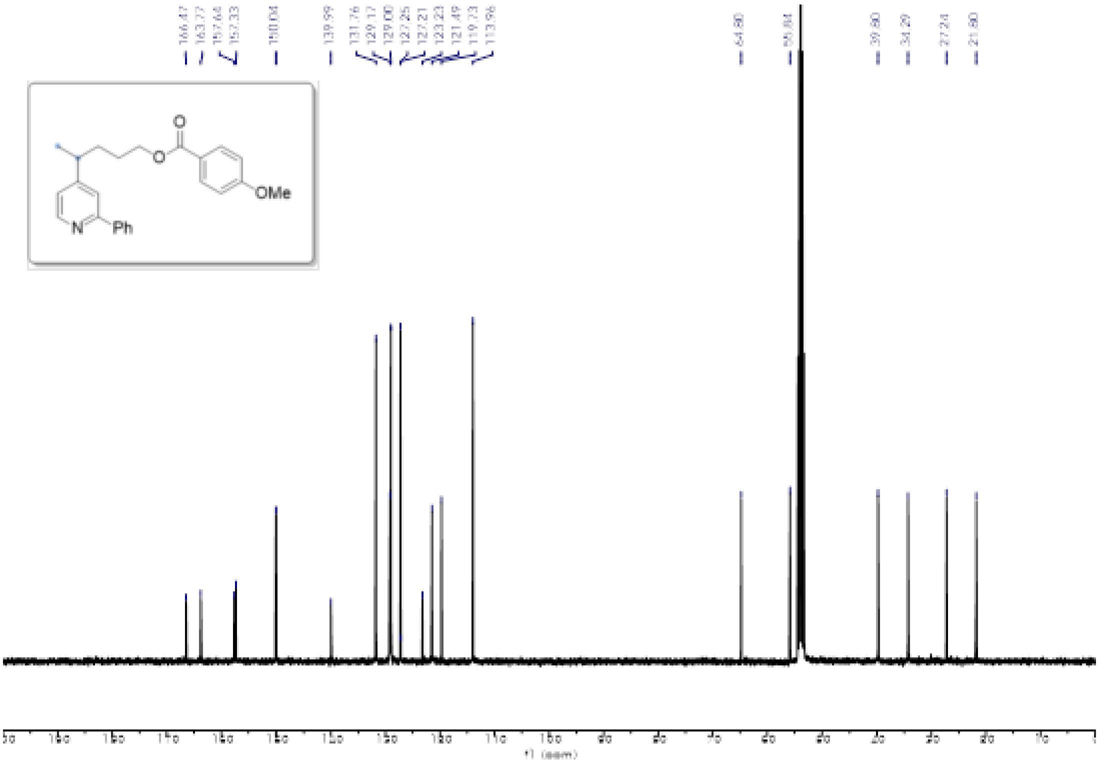

4-(2-phenylpyridin-4-yl)pentyl acetate (6t).

400 MHz, ^{1}H NMR in $CDCl_3$

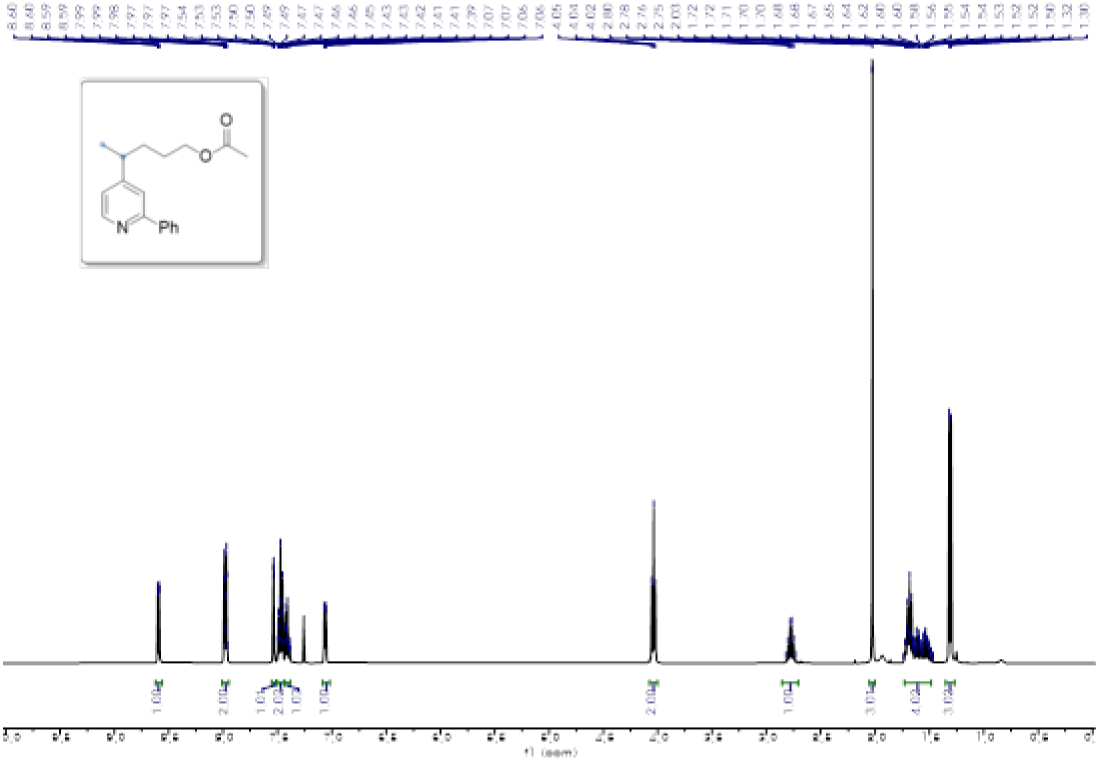

100 MHz, ^{13}C NMR in $CDCl_3$

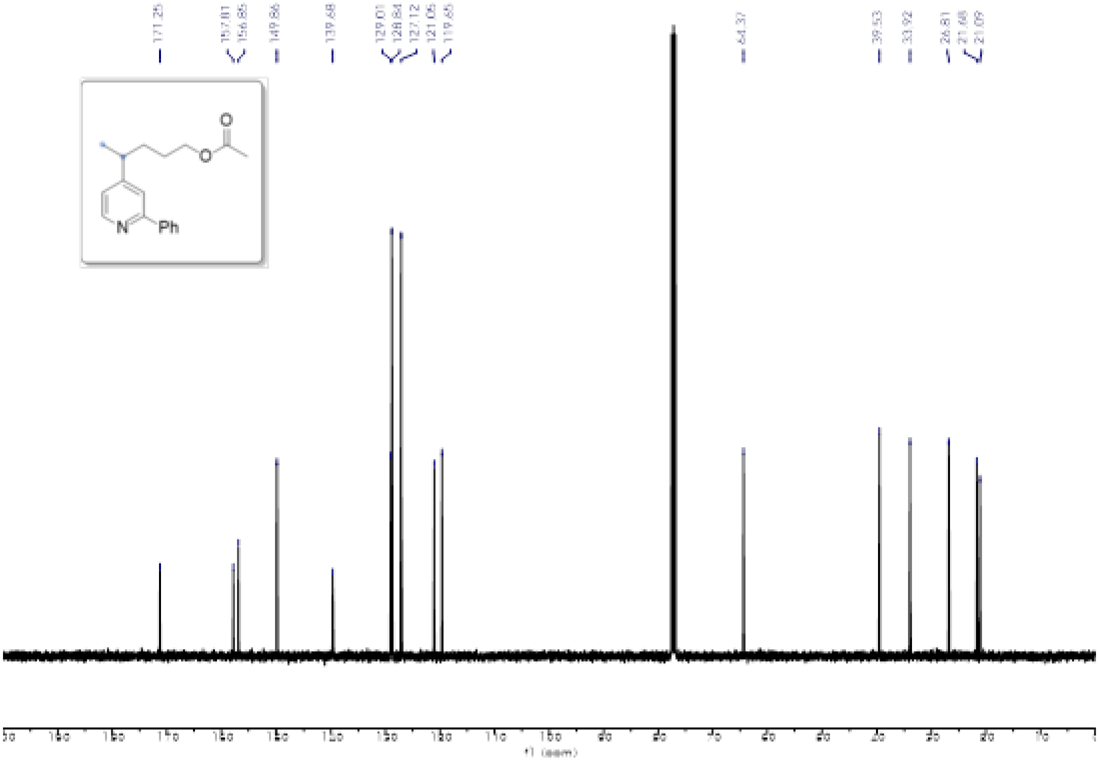

4-(5-bromopentan-2-yl)-2-phenylpyridine (6u).

600 MHz, ^{1}H NMR in $CDCl_3$

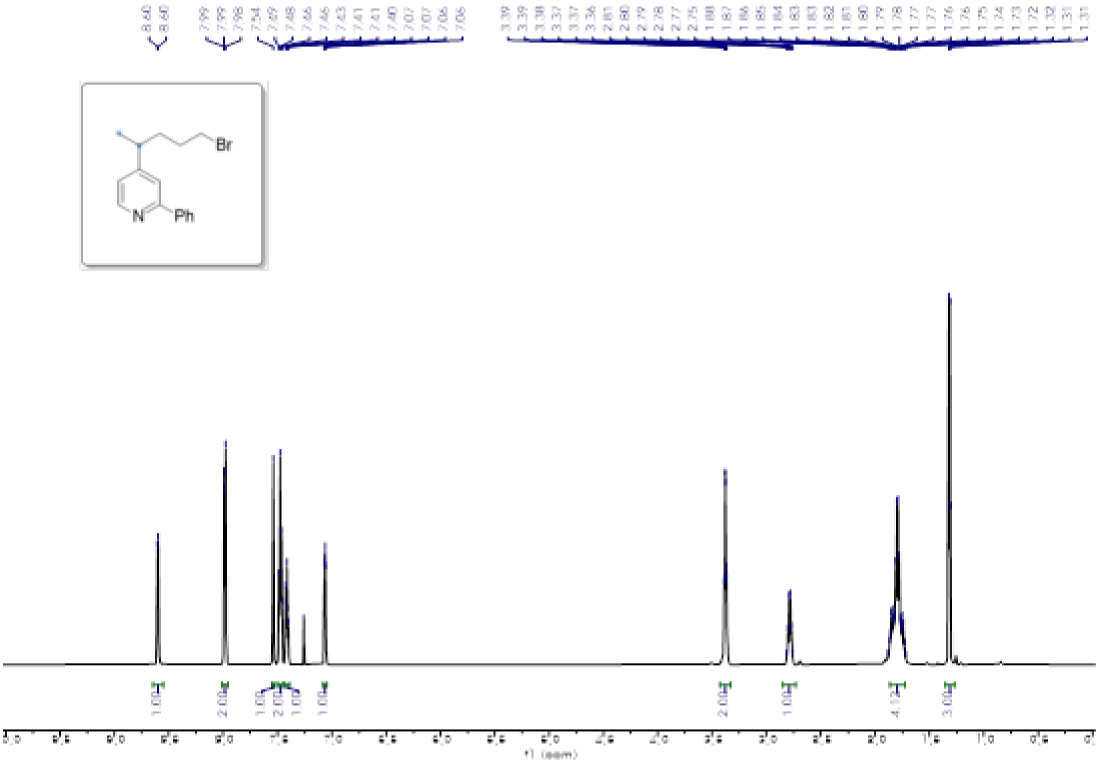

125 MHz, ^{13}C NMR in $CDCl_3$

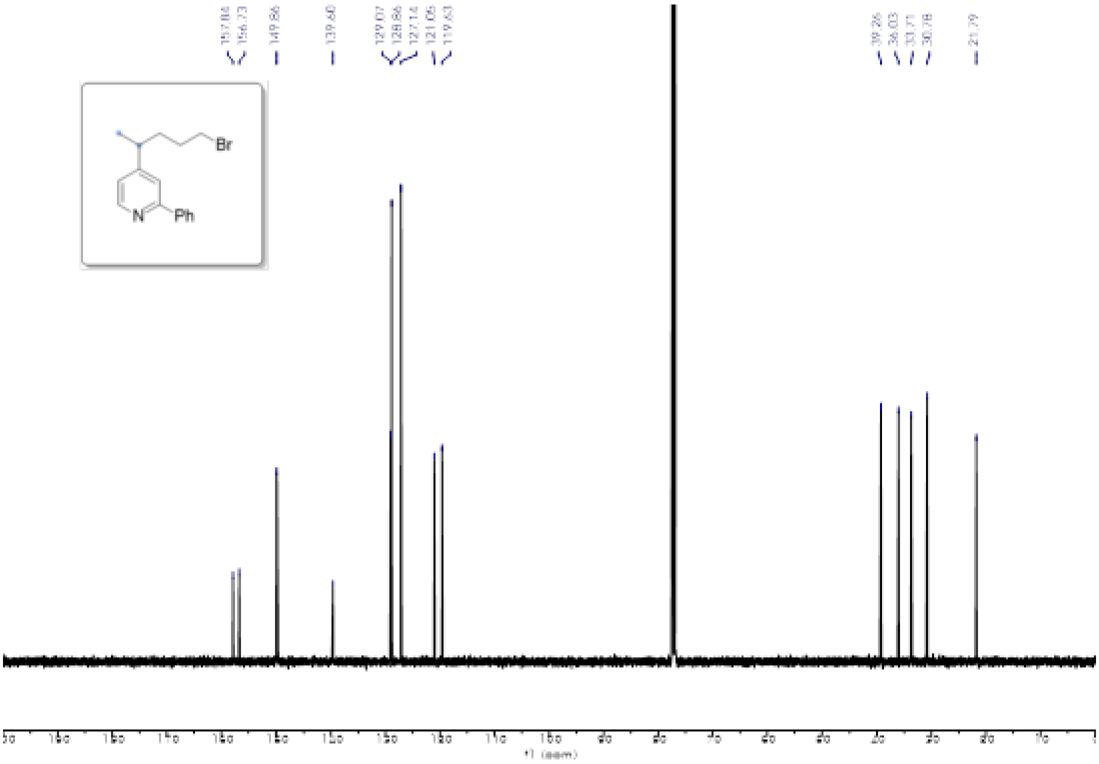

4-(4-chlorobutan-2-yl)-2-phenylpyridine (6v).

400 MHz, ^{1}H NMR in $CDCl_3$

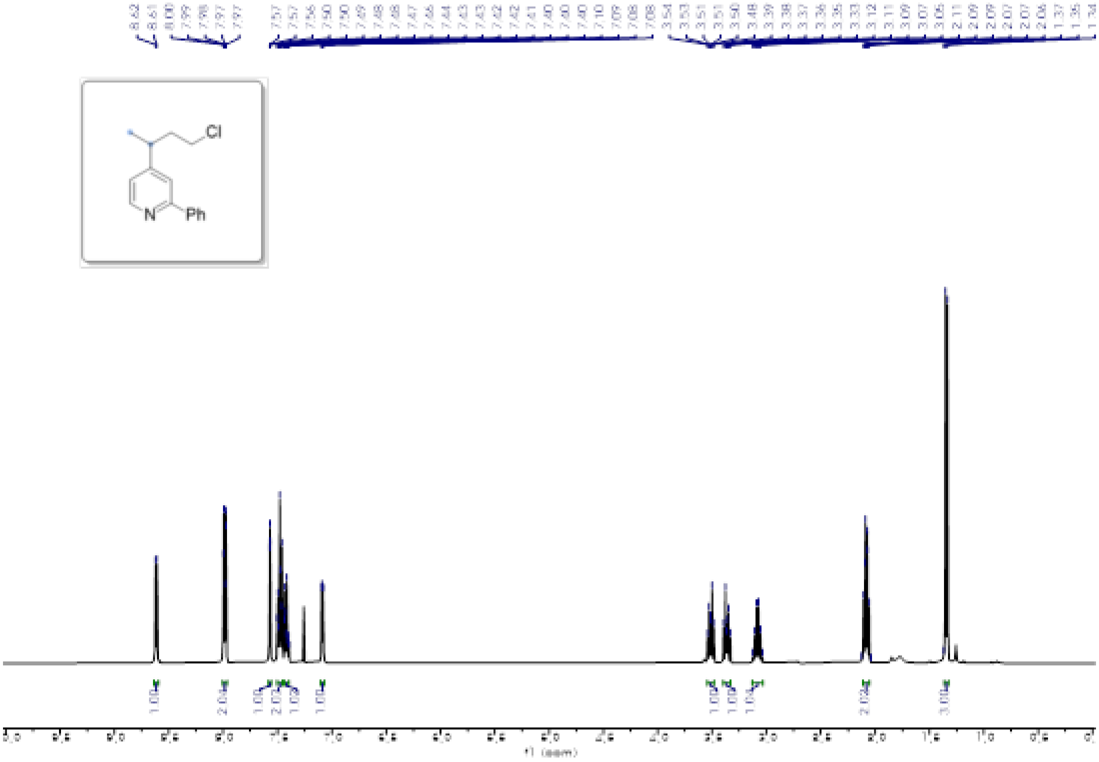

100 MHz, ^{13}C NMR in $CDCl_3$

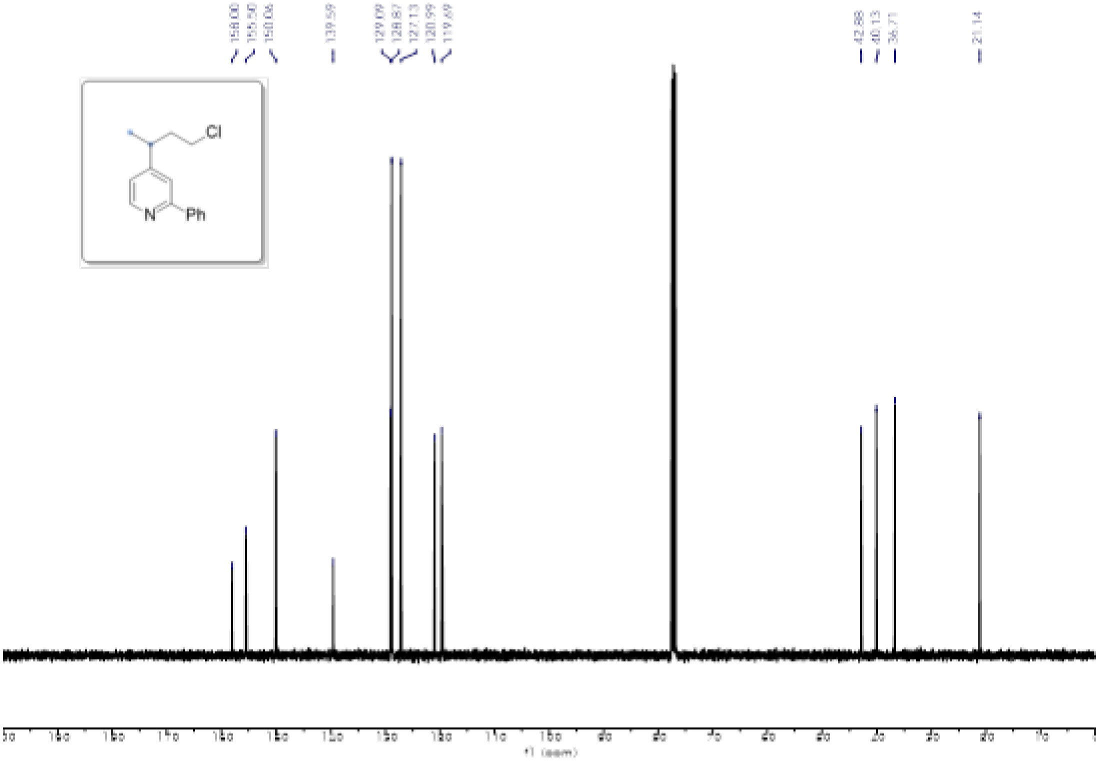

4-(pent-4-en-2-yl)-2-phenylpyridine (6w).

400 MHz, ^{1}H NMR in $CDCl_3$

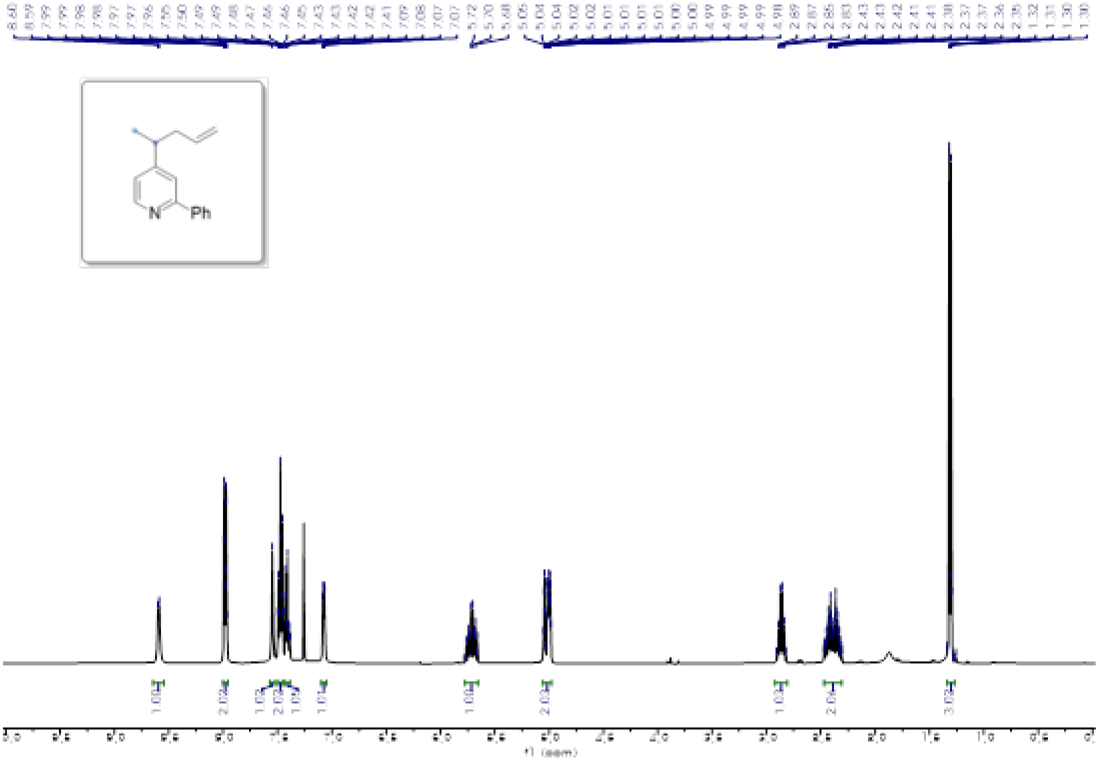

100 MHz, ^{13}C NMR in $CDCl_3$

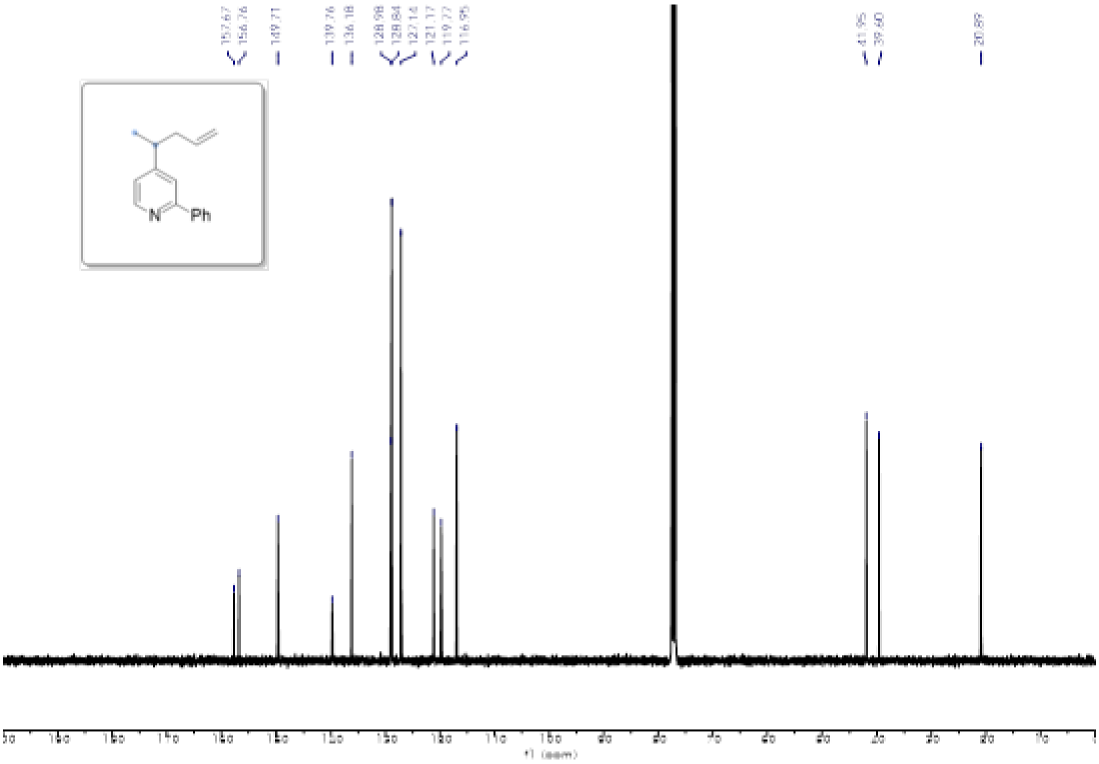

4-(2-methylhex-5-en-2-yl)-2-phenylpyridine (6x).

400 MHz, ^{1}H NMR in $CDCl_3$

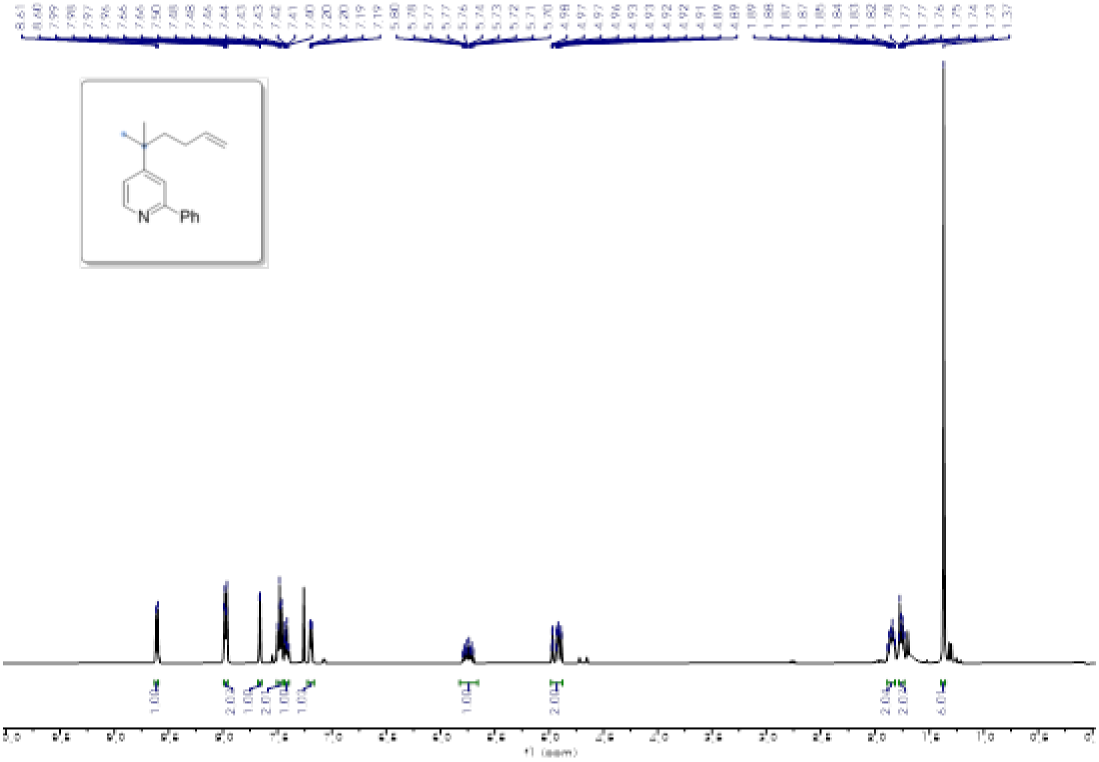

100 MHz, ^{13}C NMR in $CDCl_3$

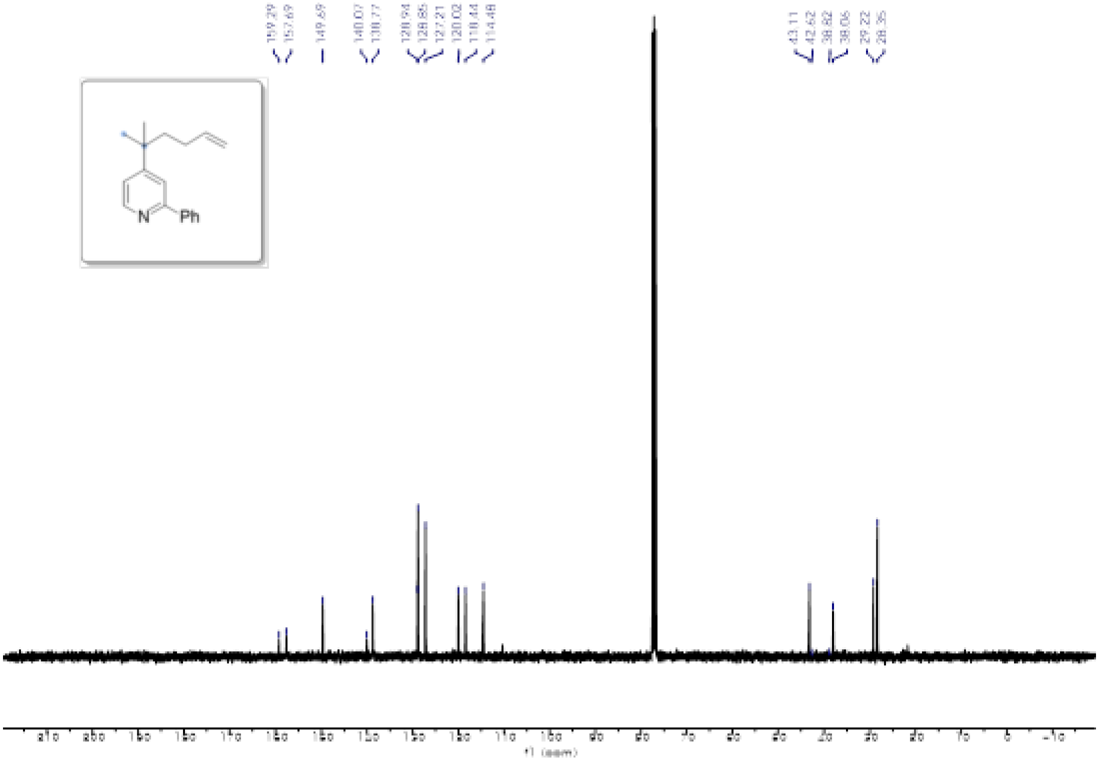

4-cyclopentyl-2-phenylpyridine (6y).

400 MHz, ^{1}H NMR in $CDCl_3$

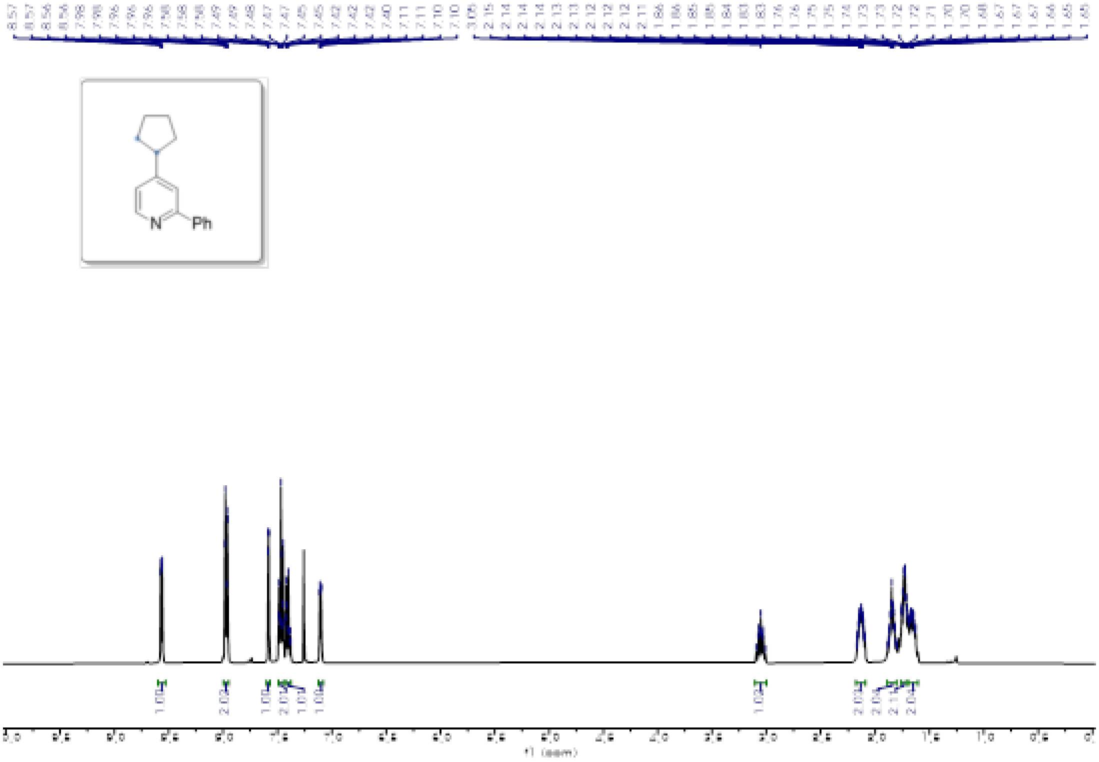

100 MHz, ^{13}C NMR in $CDCl_3$

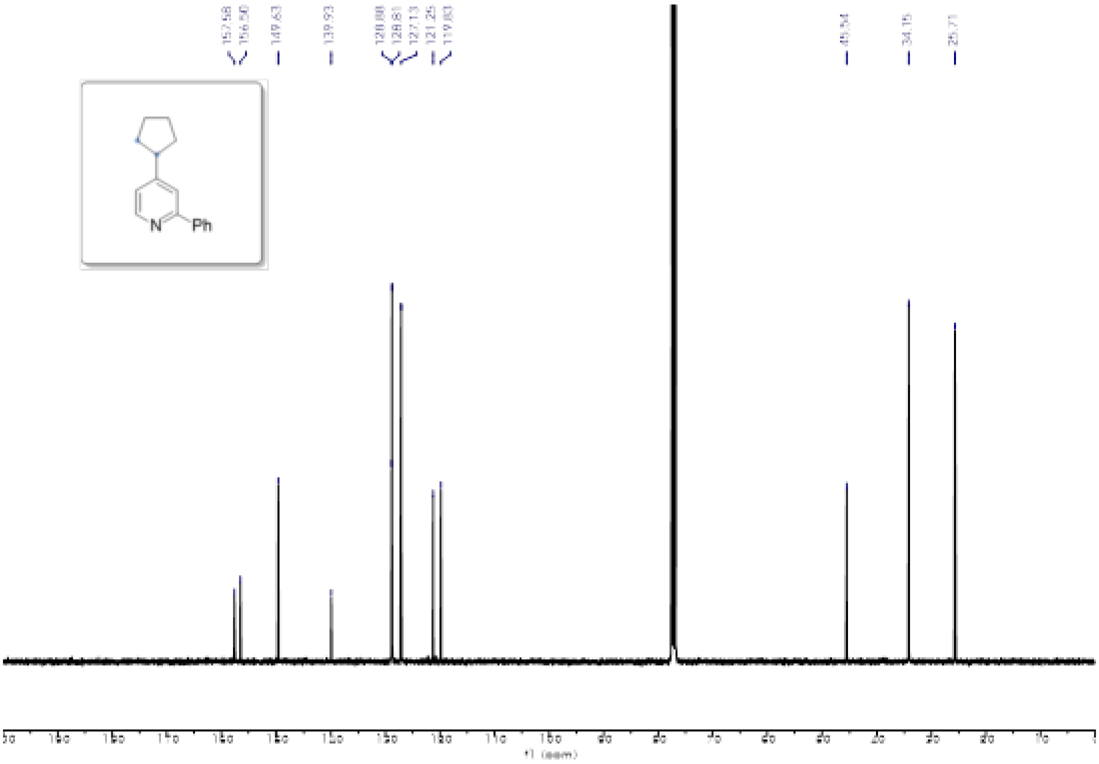

4-cyclohexyl-2-phenylpyridine (6z).

400 MHz, ^{1}H NMR in $CDCl_3$

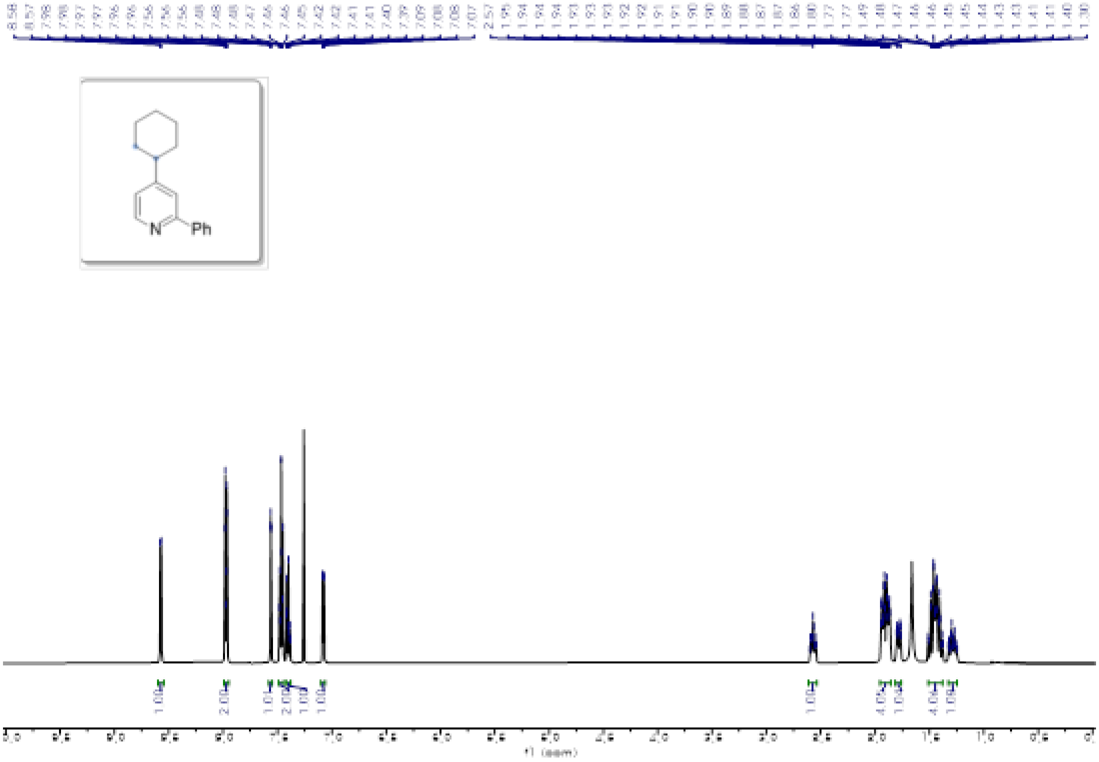

100 MHz, ^{13}C NMR in $CDCl_3$

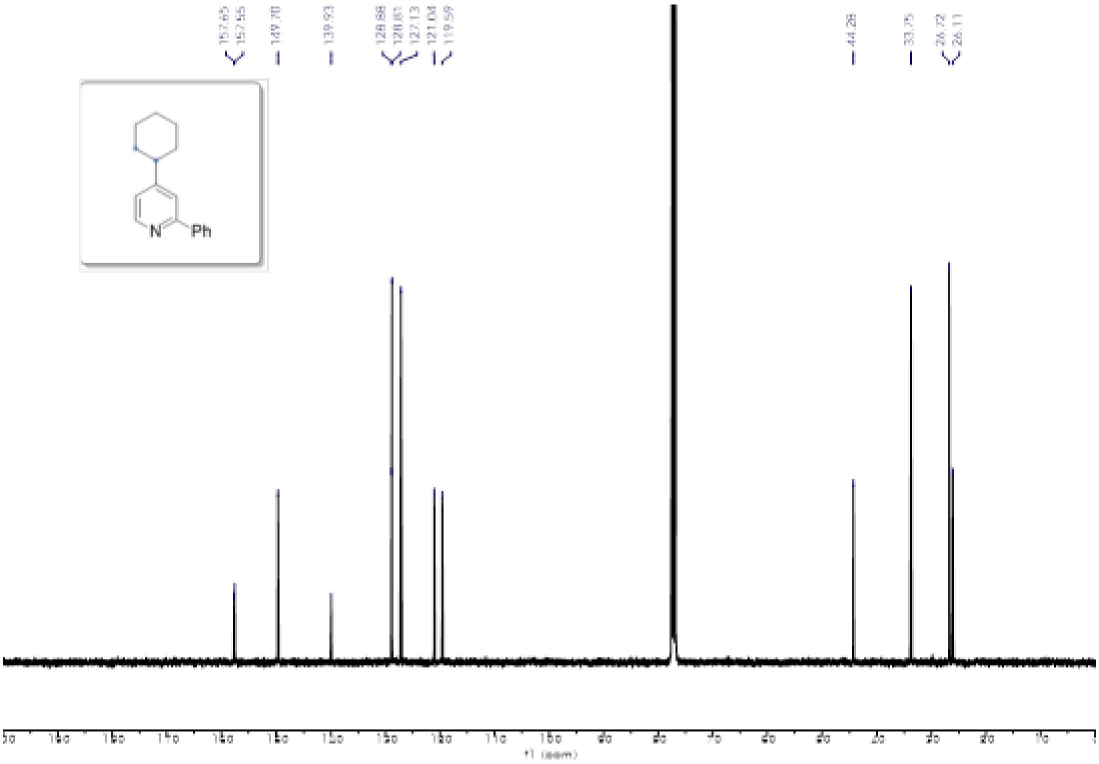

4-cycloheptyl-2-phenylpyridine (6aa).

500 MHz, ^{1}H NMR in $CDCl_3$

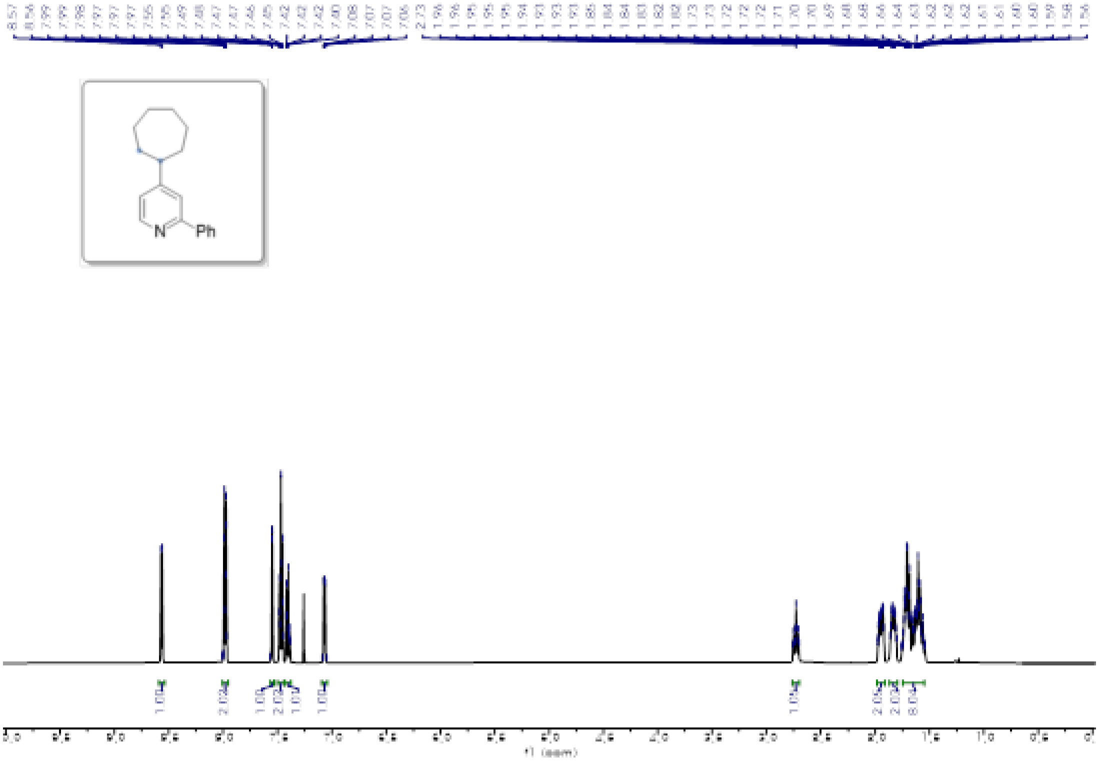

100 MHz, ^{13}C NMR in $CDCl_3$

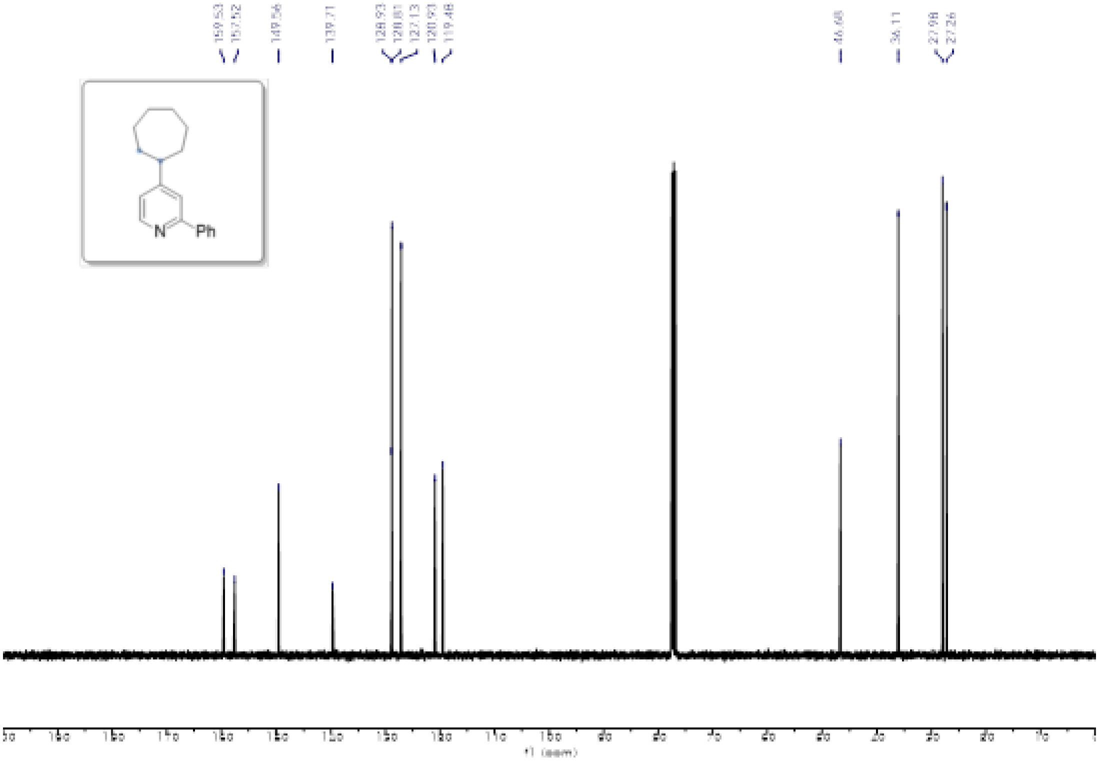

4-cyclooctyl-2-phenylpyridine (6ab).

400 MHz, ^{1}H NMR in $CDCl_3$

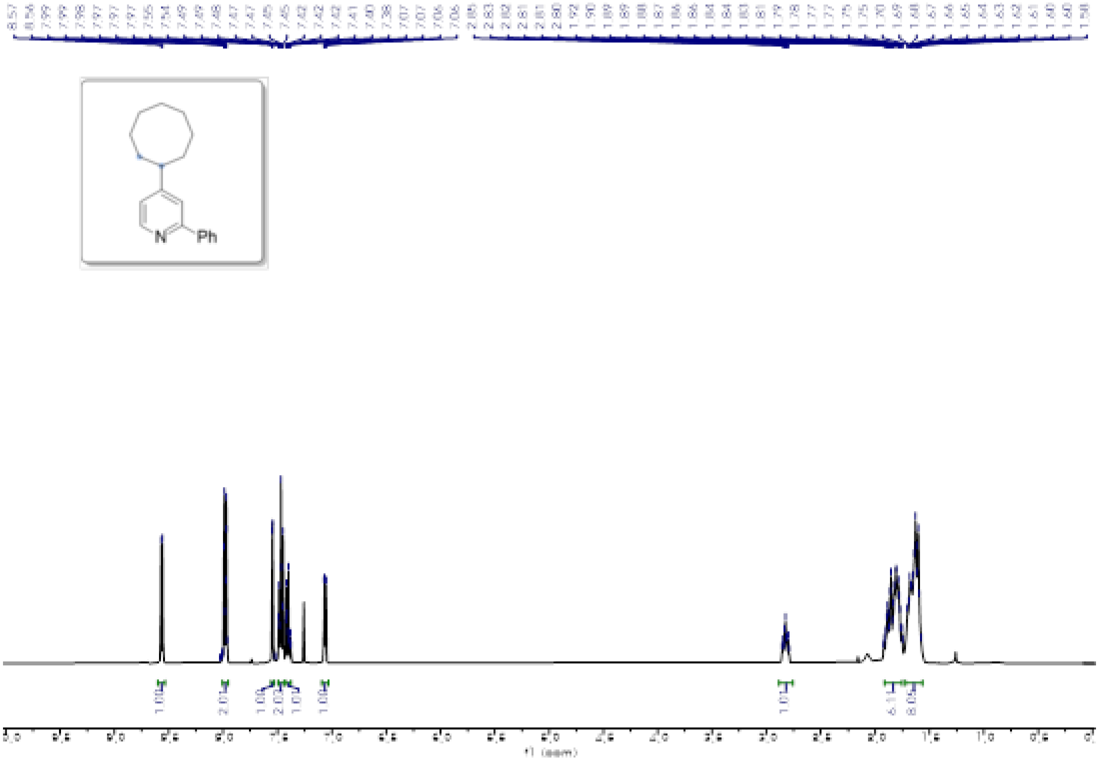

100 MHz, ^{13}C NMR in $CDCl_3$

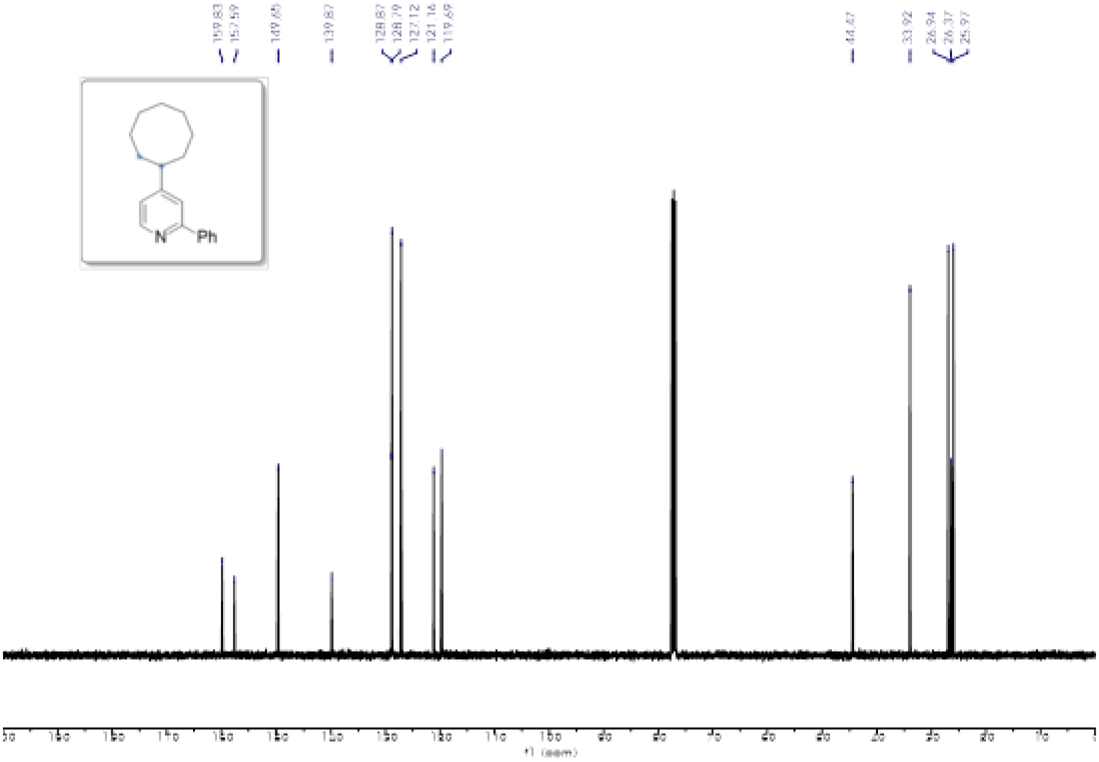

4-(octan-4-yl)-2-phenylpyridine (6ac).

500 MHz, ^{1}H NMR in $CDCl_3$

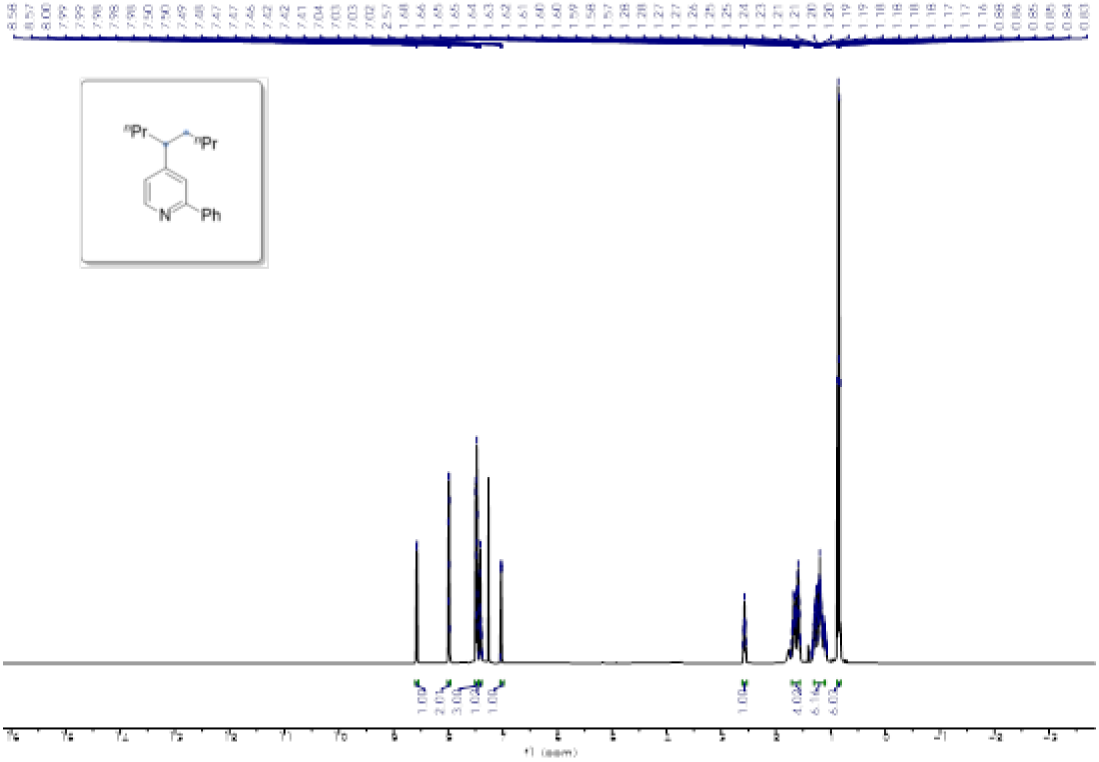

100 MHz, ^{13}C NMR in $CDCl_3$

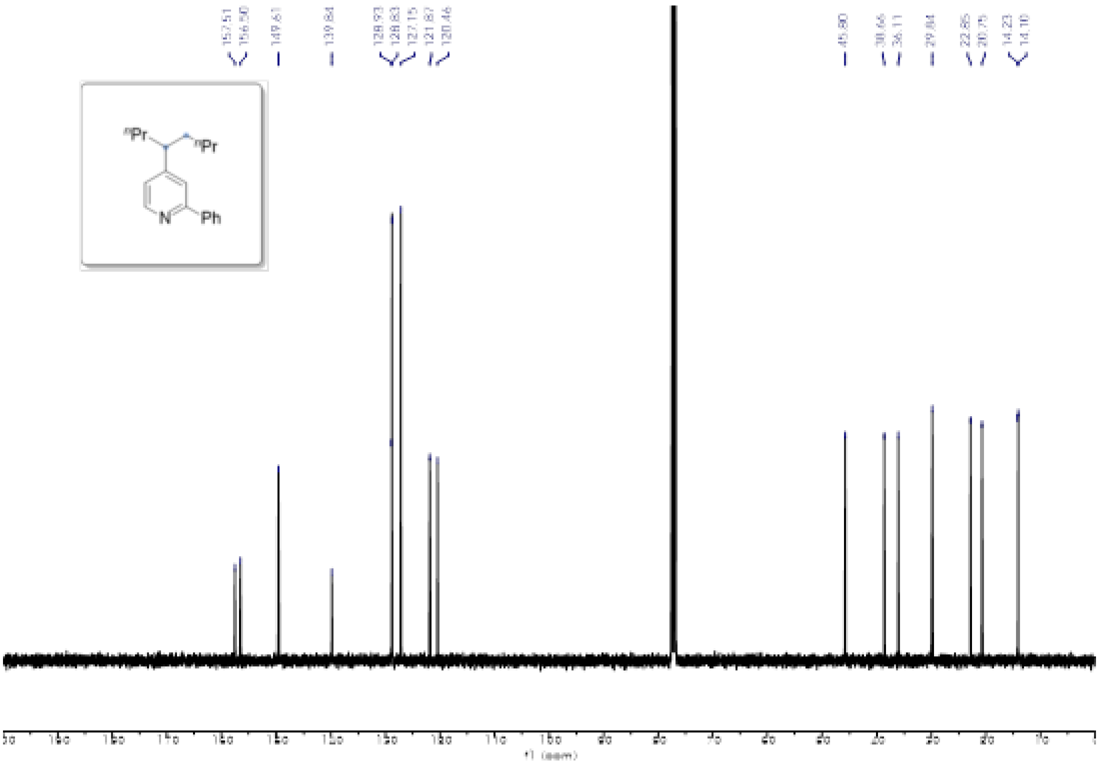

2-phenyl-4-(1,2,3,4-tetrahydro-1,4-epoxynaphthalen-2-yl)pyridine (6ad).

400 MHz, ^{1}H NMR in $CDCl_3$

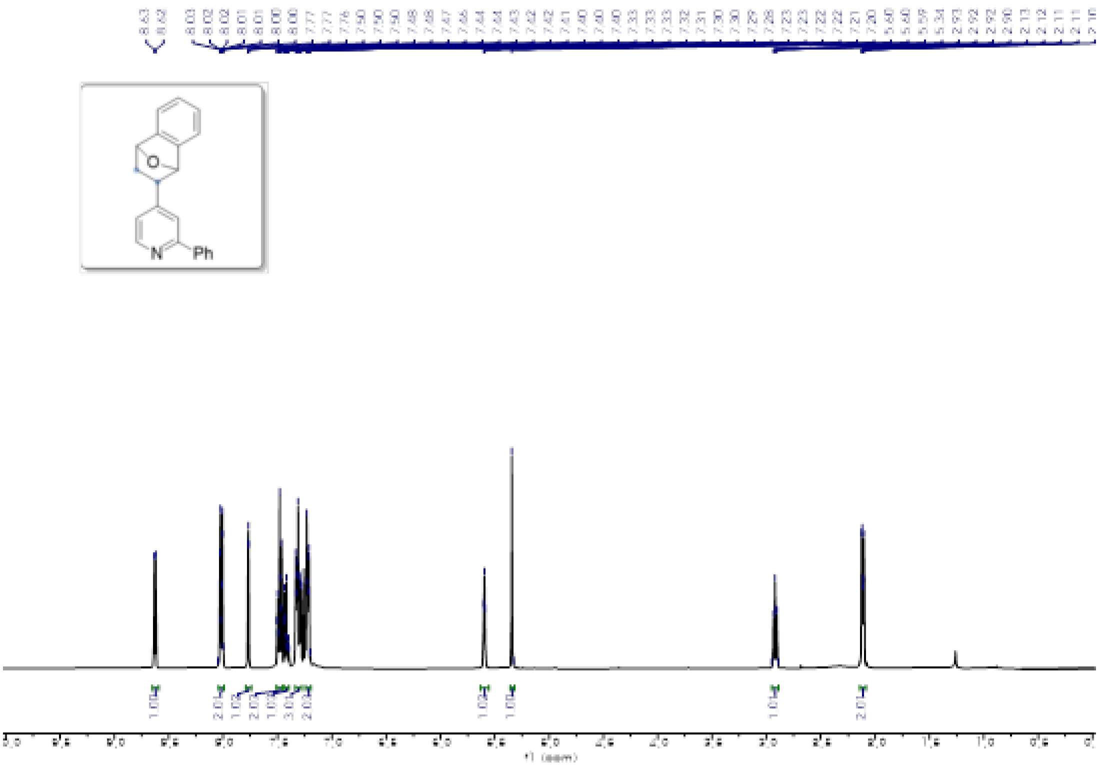

100 MHz, ^{13}C NMR in $CDCl_3$

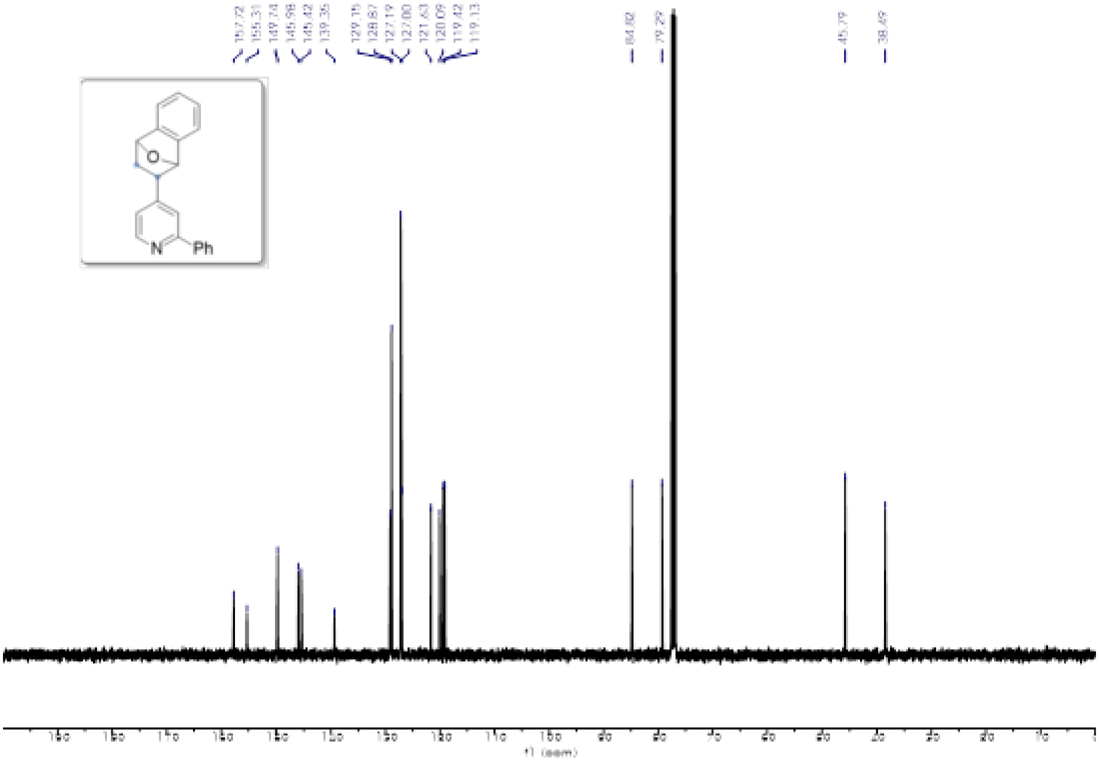

4-(tert-pentyl)-2-phenylpyridine (6ae).

400 MHz, ^{1}H NMR in $CDCl_3$

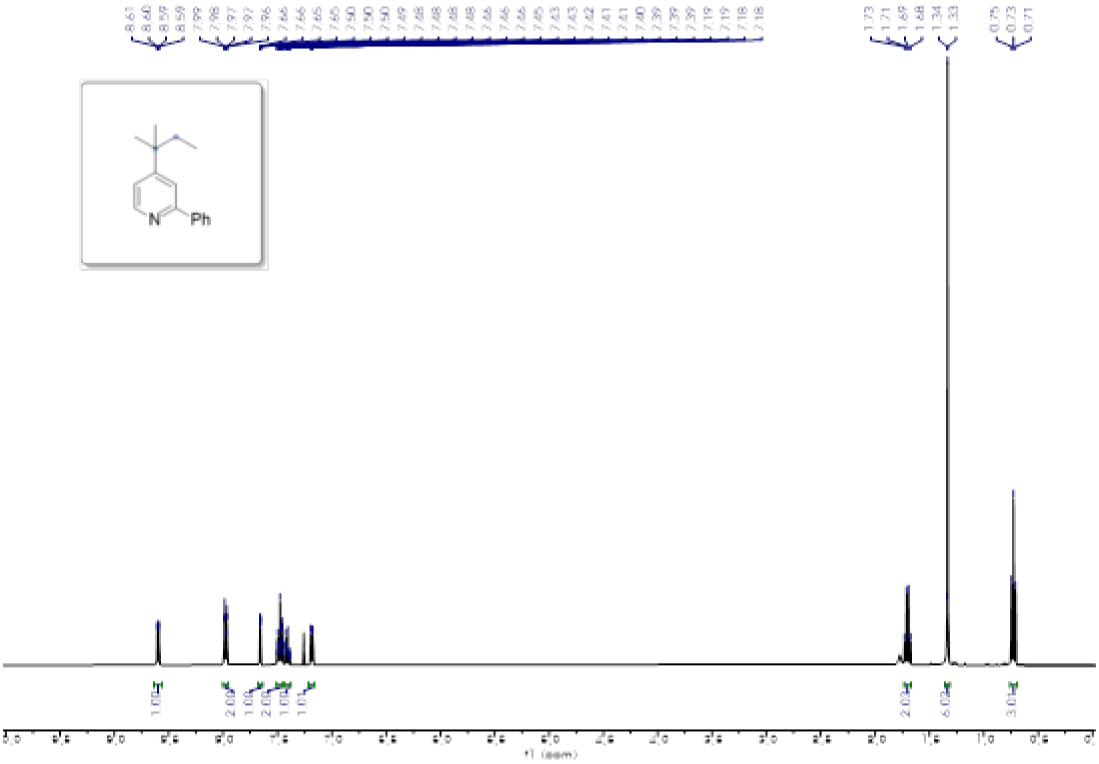

100 MHz, ^{13}C NMR in $CDCl_3$

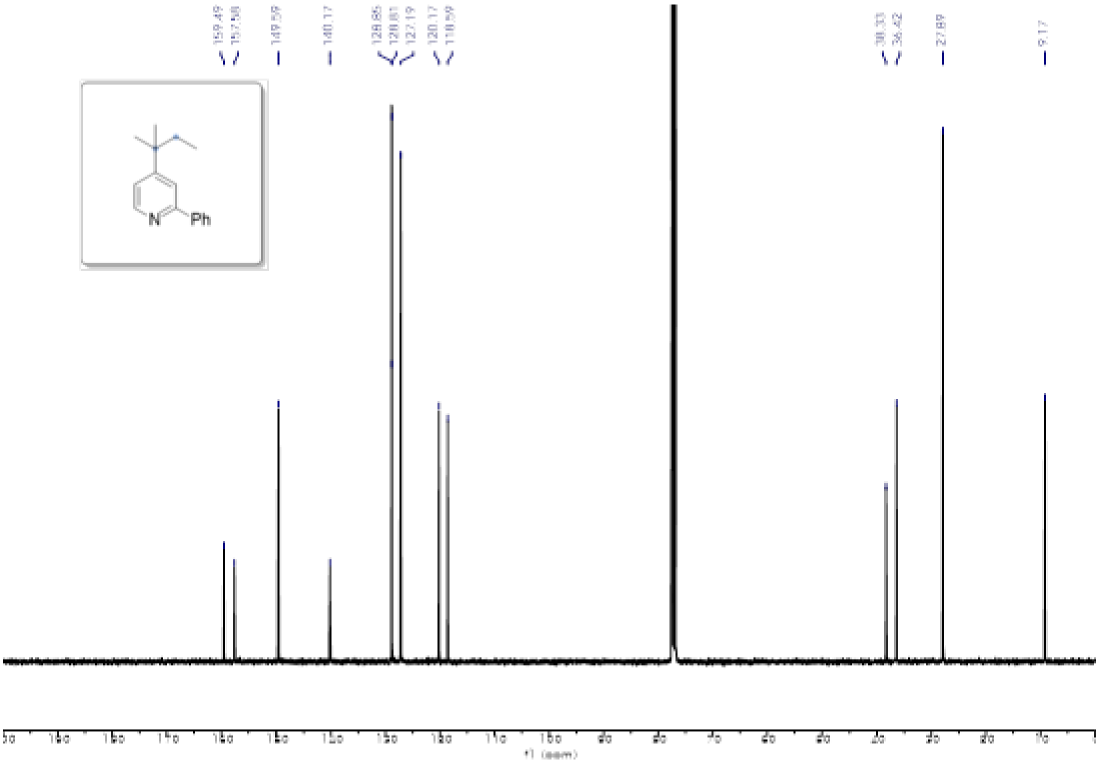

6-methyl-6-(2-phenylpyridin-4-yl)heptan-2-ol (6af).

400 MHz, ^{1}H NMR in $CDCl_3$

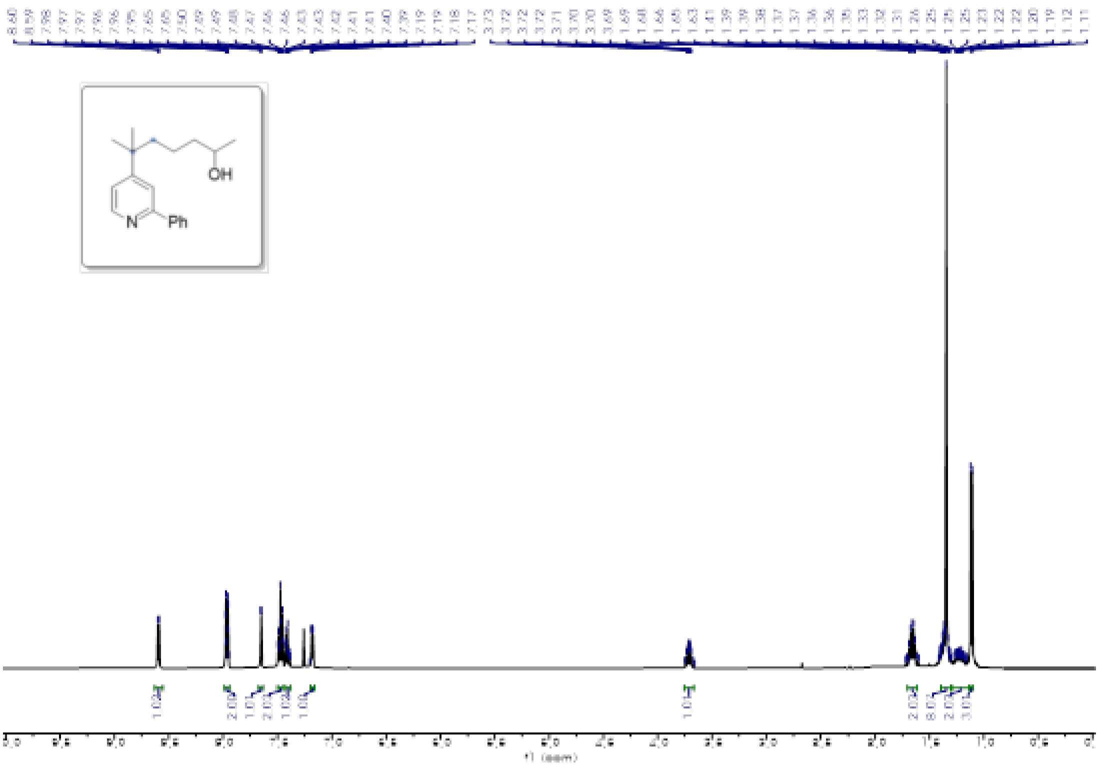

100 MHz, ^{13}C NMR in $CDCl_3$

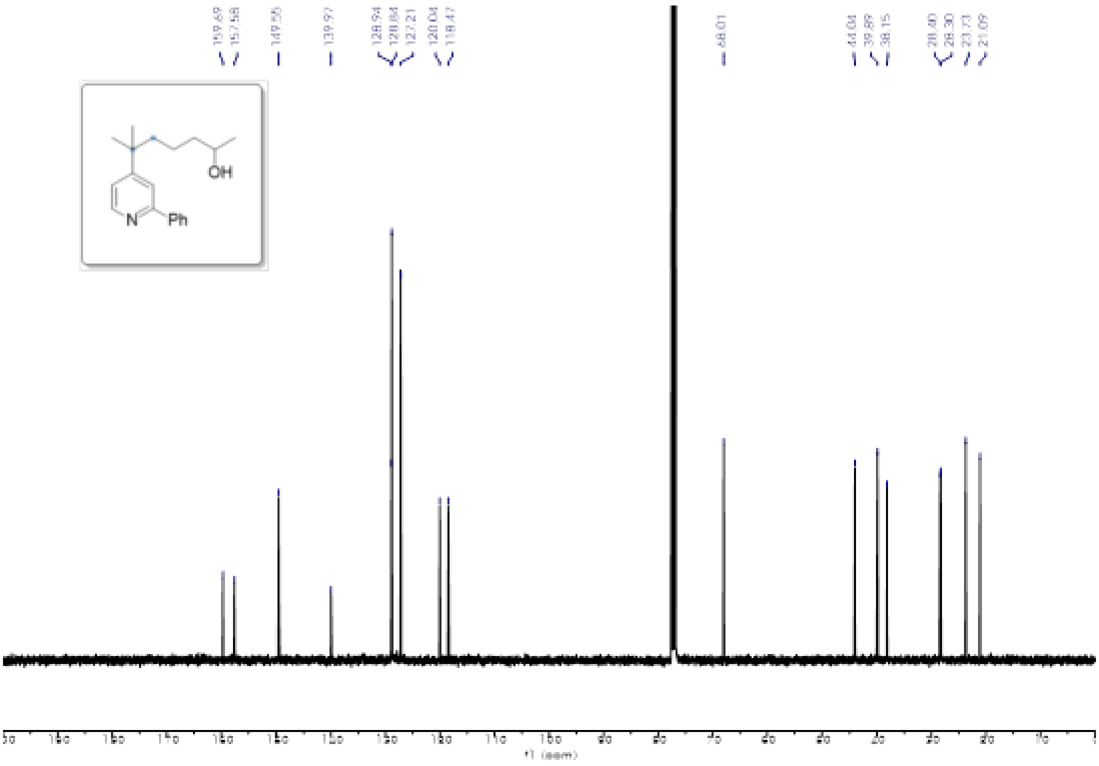

2,6-dimethyl-6-(2-phenylpyridin-4-yl)heptanal (6ag).

400 MHz, ^{1}H NMR in $CDCl_3$

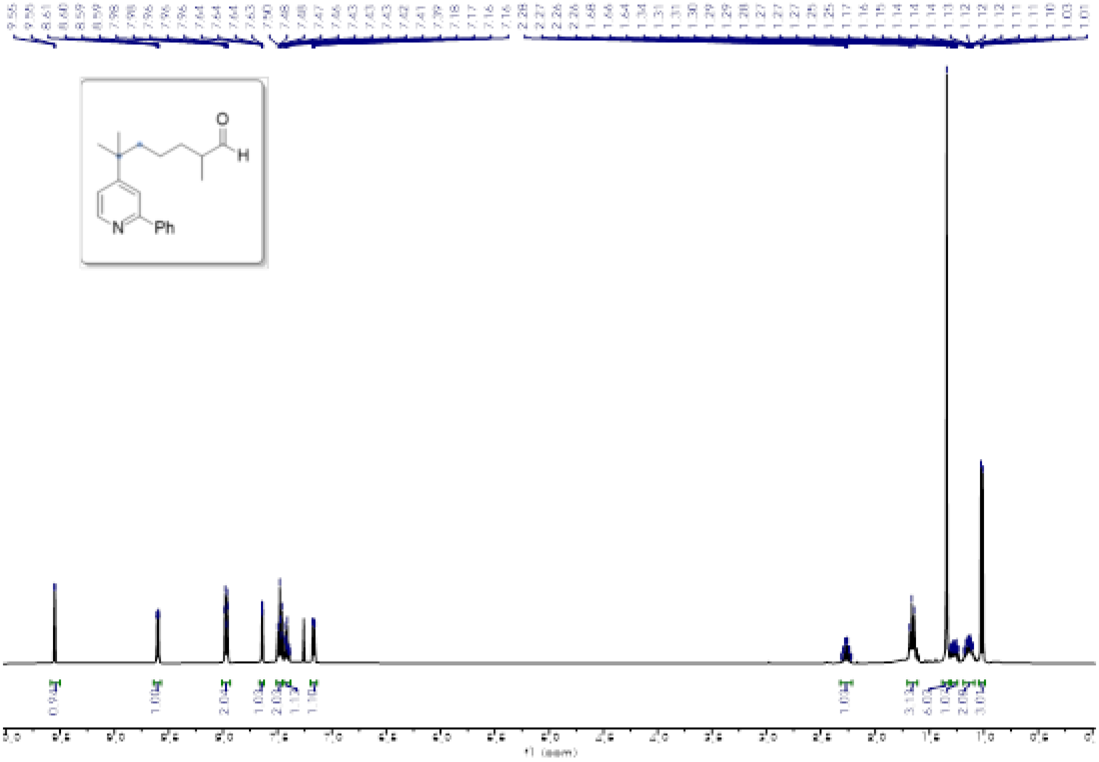

100 MHz, ^{13}C NMR in $CDCl_3$

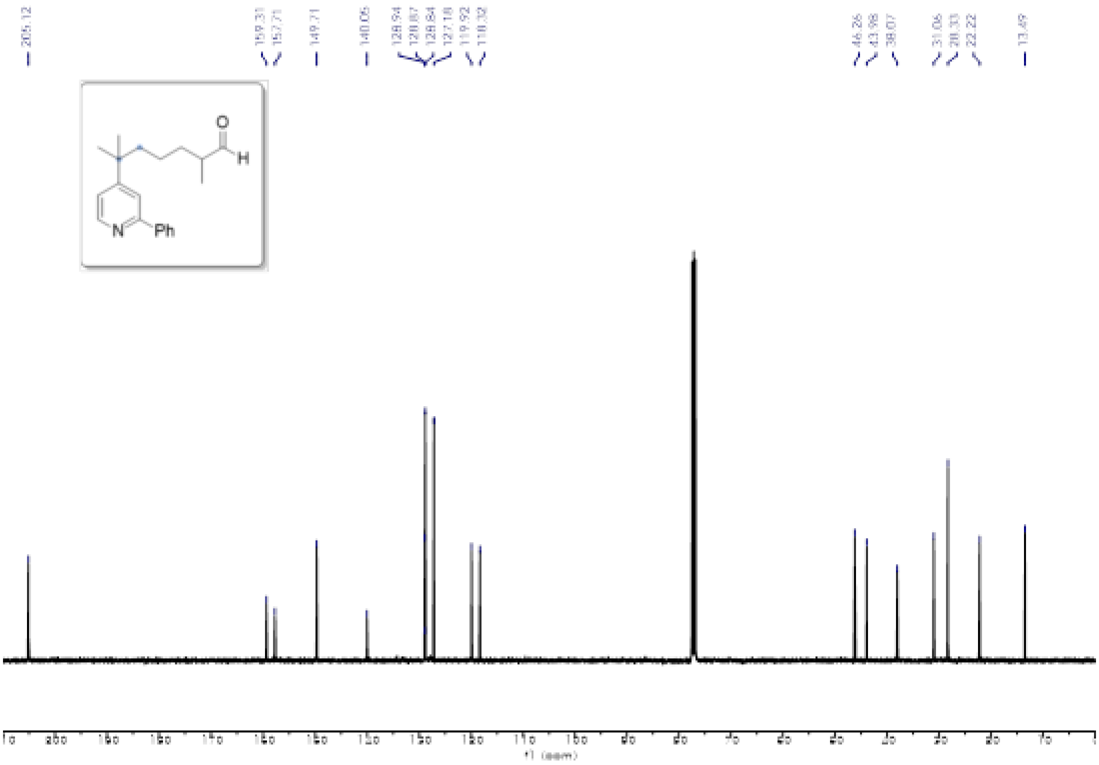

4-(2,3-dimethylbutan-2-yl)-2-phenylpyridine (6ah).

400 MHz, ^{1}H NMR in $CDCl_3$

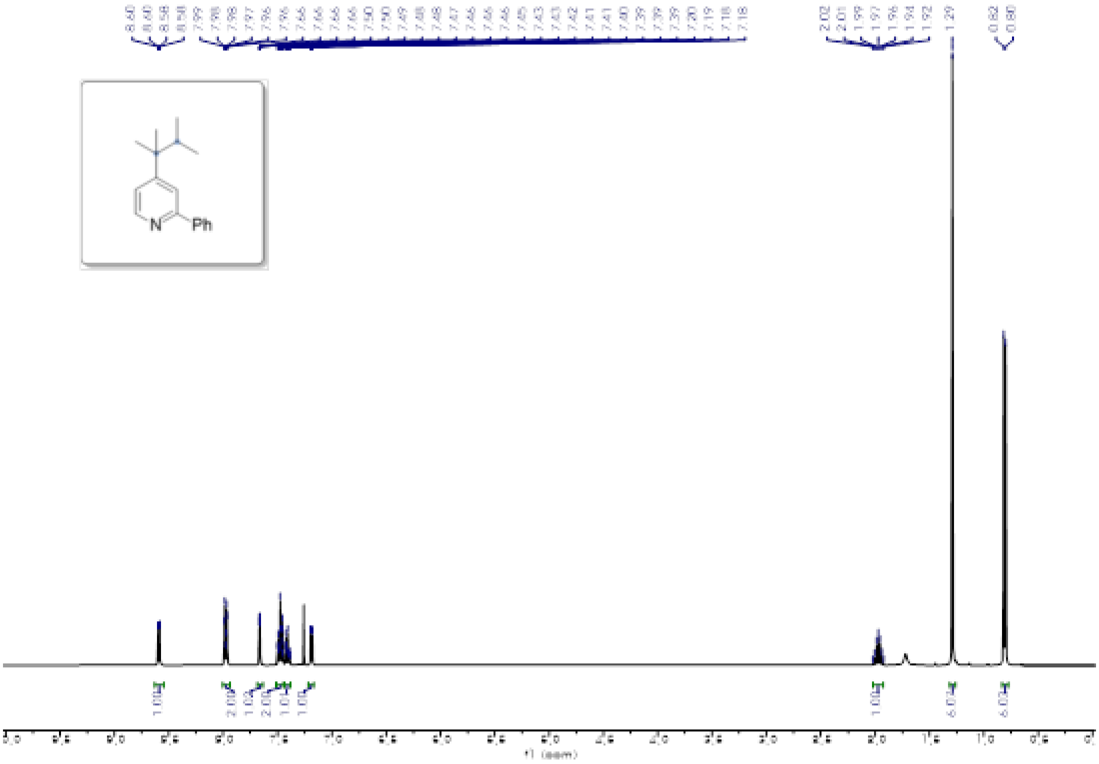

100 MHz, ^{13}C NMR in $CDCl_3$

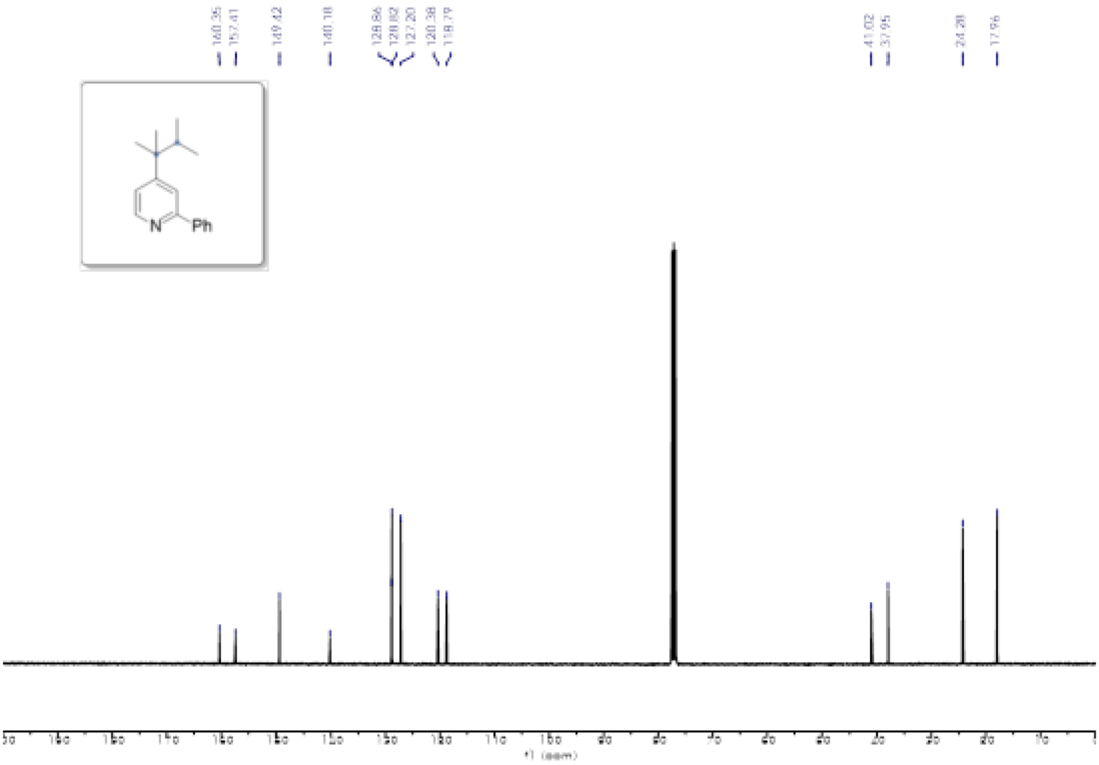

4-(1-isobutoxyethyl)-2-phenylpyridine (6ai).

500 MHz, ^{1}H NMR in CD_2Cl_2

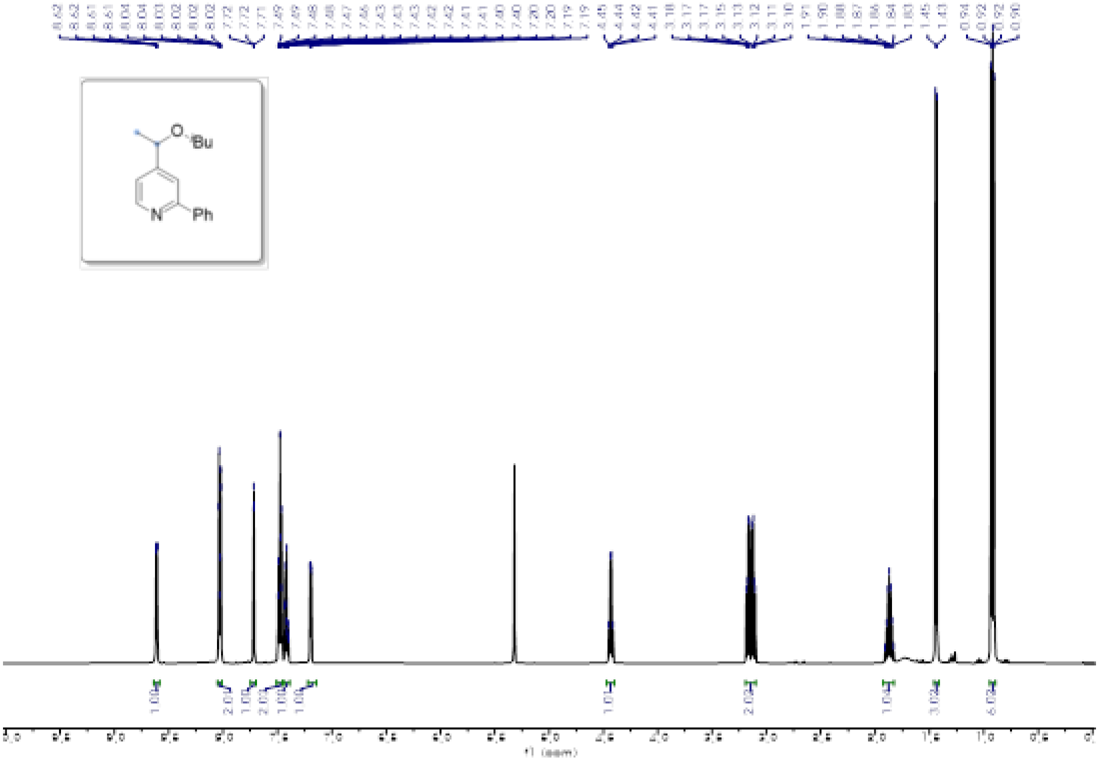

100 MHz, ^{13}C NMR in CD_2Cl_2

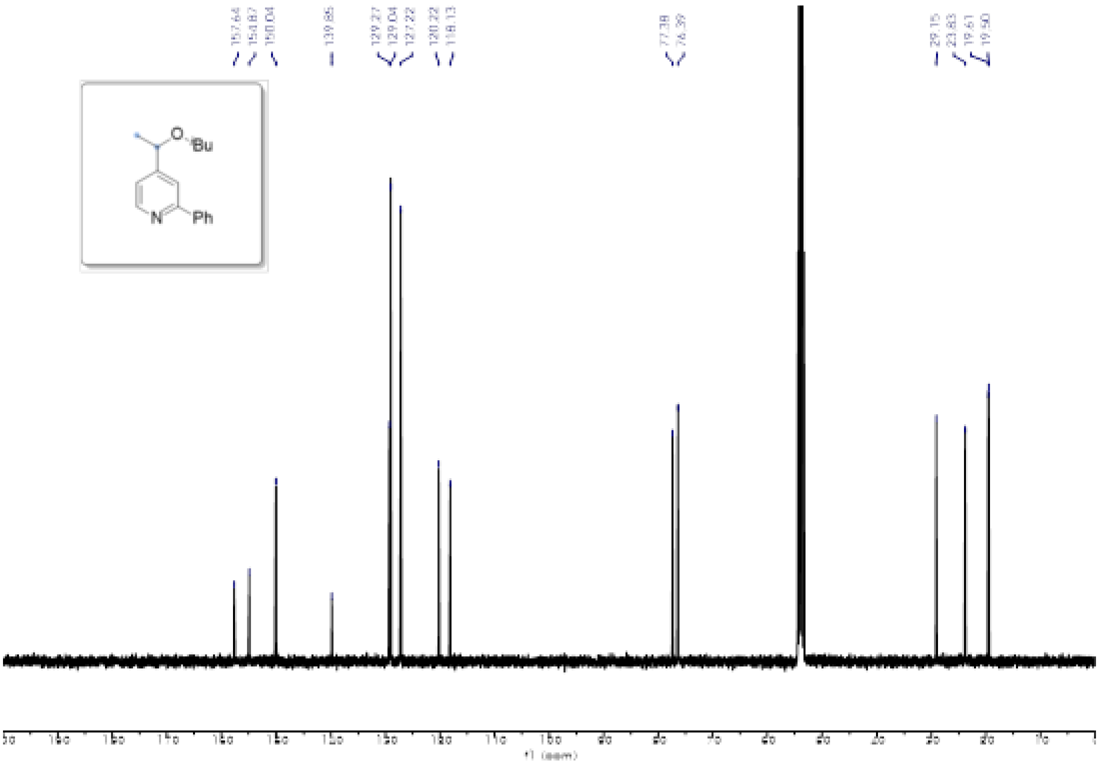

4-(1-butoxyethyl)-2-phenylpyridine (6aj).

500 MHz, ^{1}H NMR in $CDCl_3$

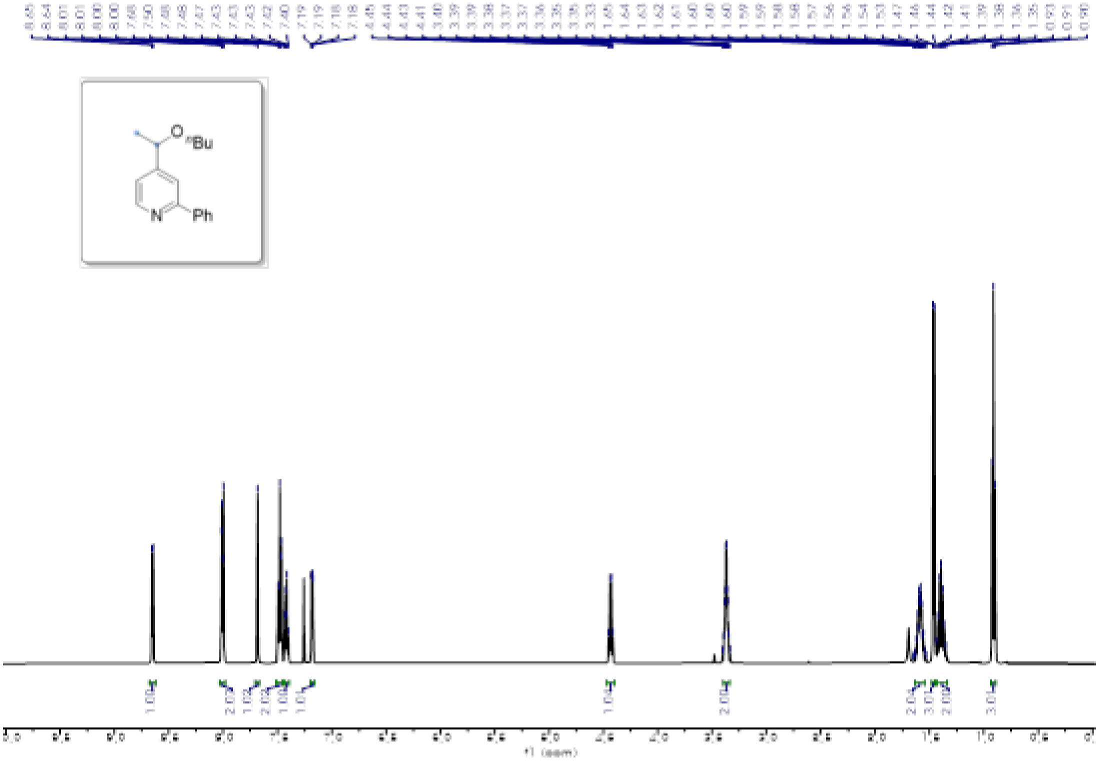

100 MHz, ^{13}C NMR in $CDCl_3$

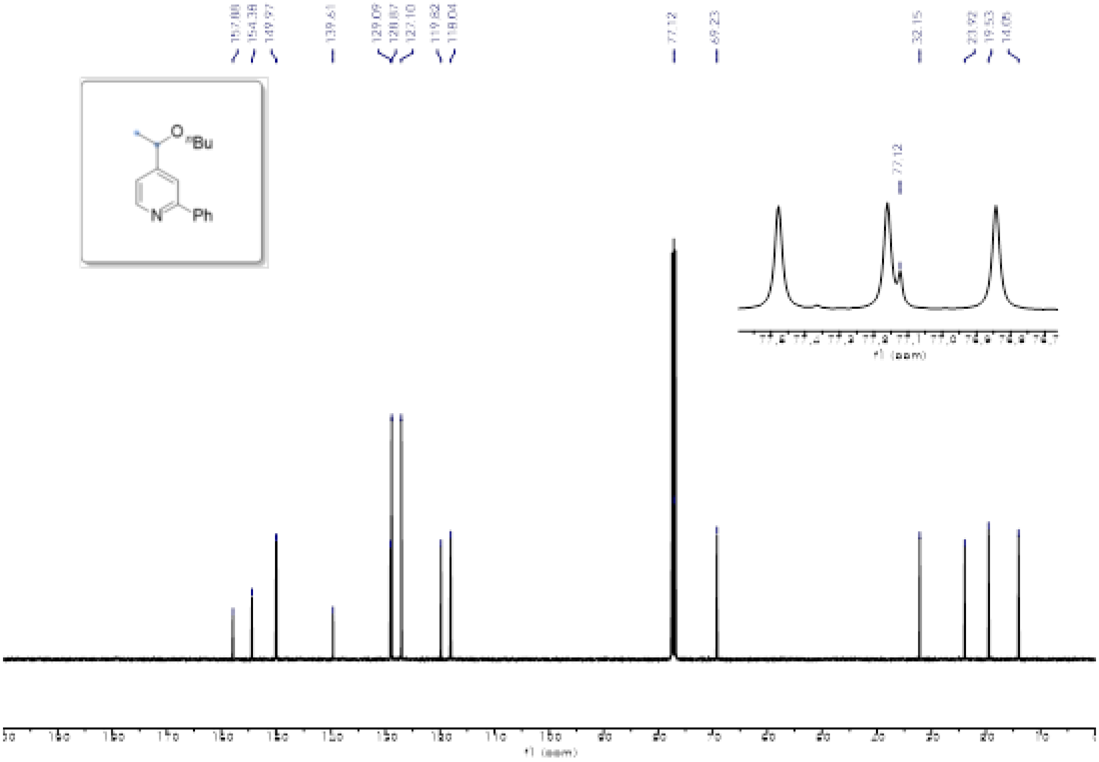

4-(1-(cyclohexyloxy)ethyl)-2-phenylpyridine (6ak).

500 MHz, ^1H NMR in $CDCl_3$

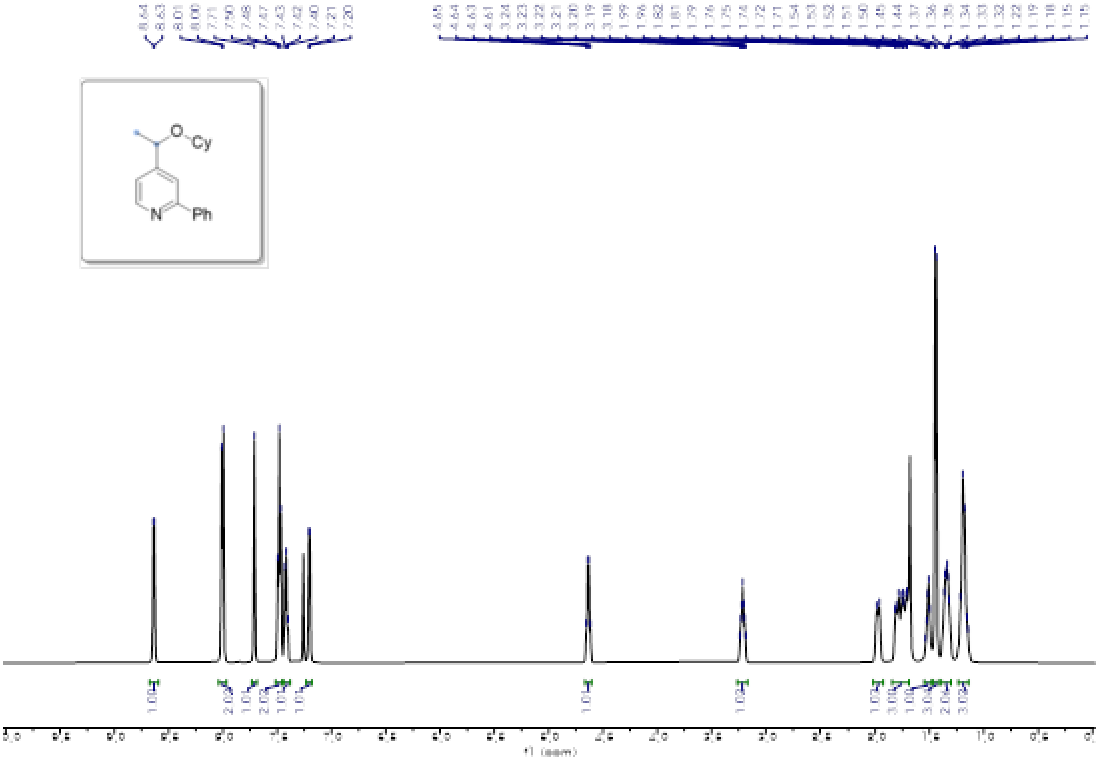

100 MHz, ^{13}C NMR in $CDCl_3$

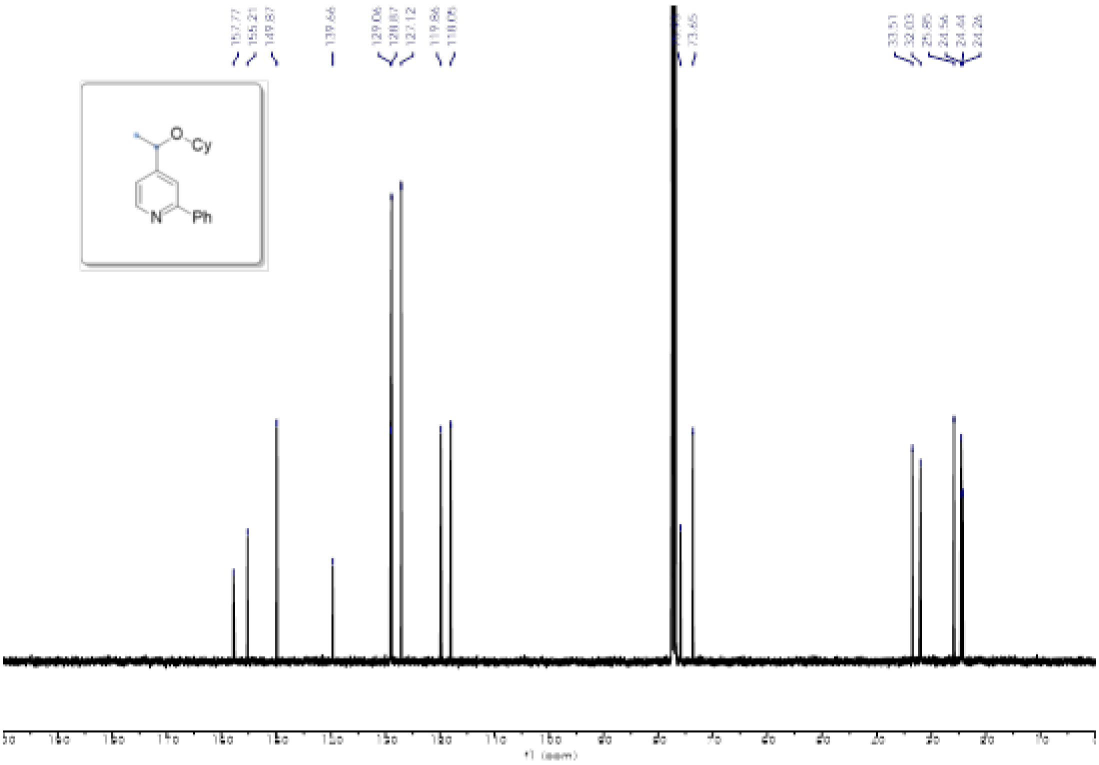

4-(1-phenoxyethyl)-2-phenylpyridine (6al).

500 MHz, ^{1}H NMR in $CDCl_3$

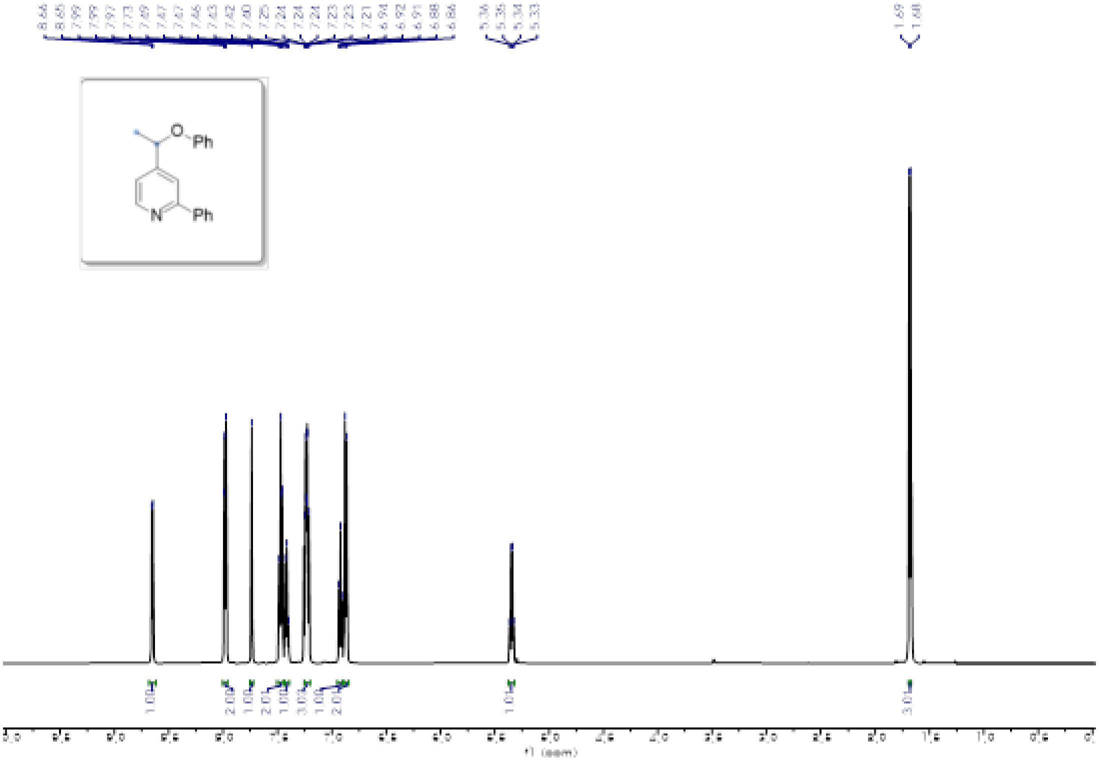

100 MHz, ^{13}C NMR in $CDCl_3$

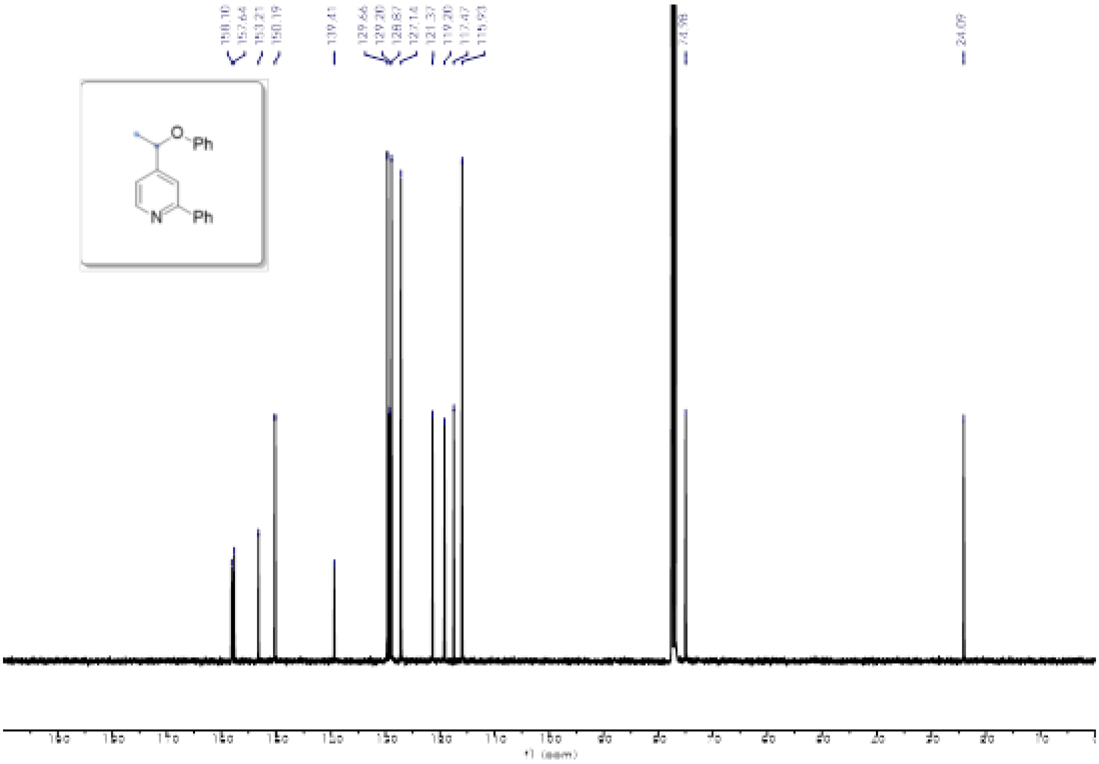

4-(1-(2-chloroethoxy)ethyl)-2-phenylpyridine (6am).

400 MHz, ^1H NMR in $CDCl_3$

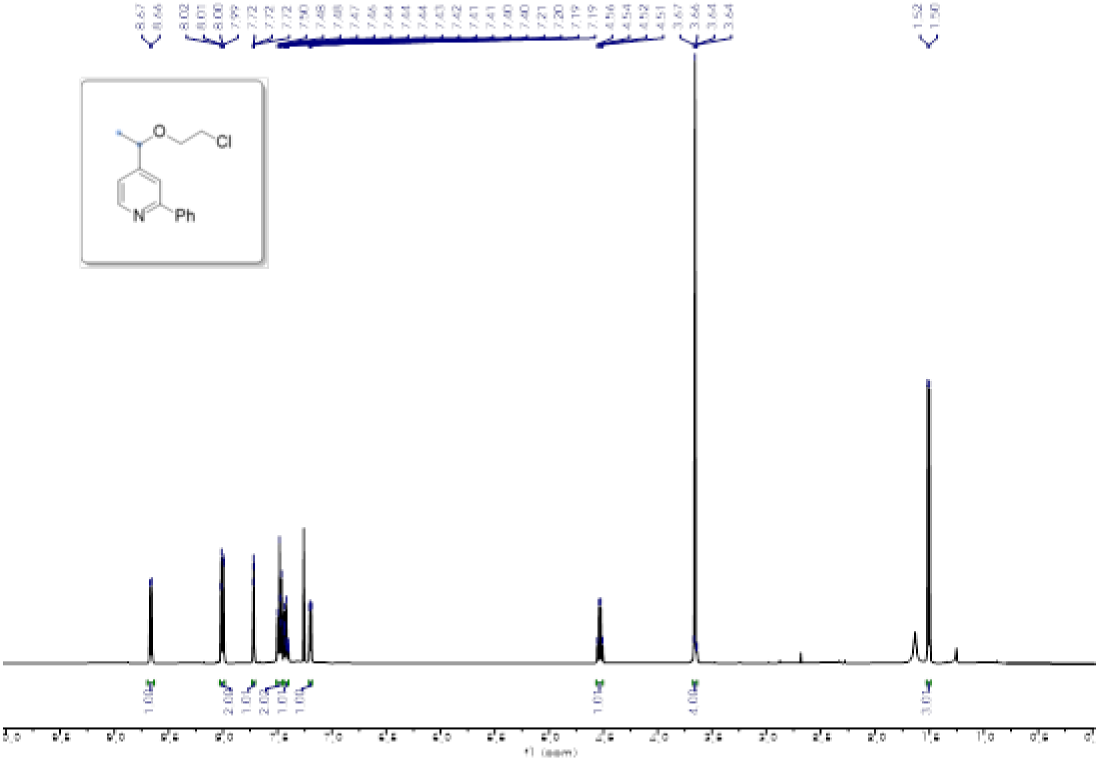

100 MHz, ^{13}C NMR in $CDCl_3$

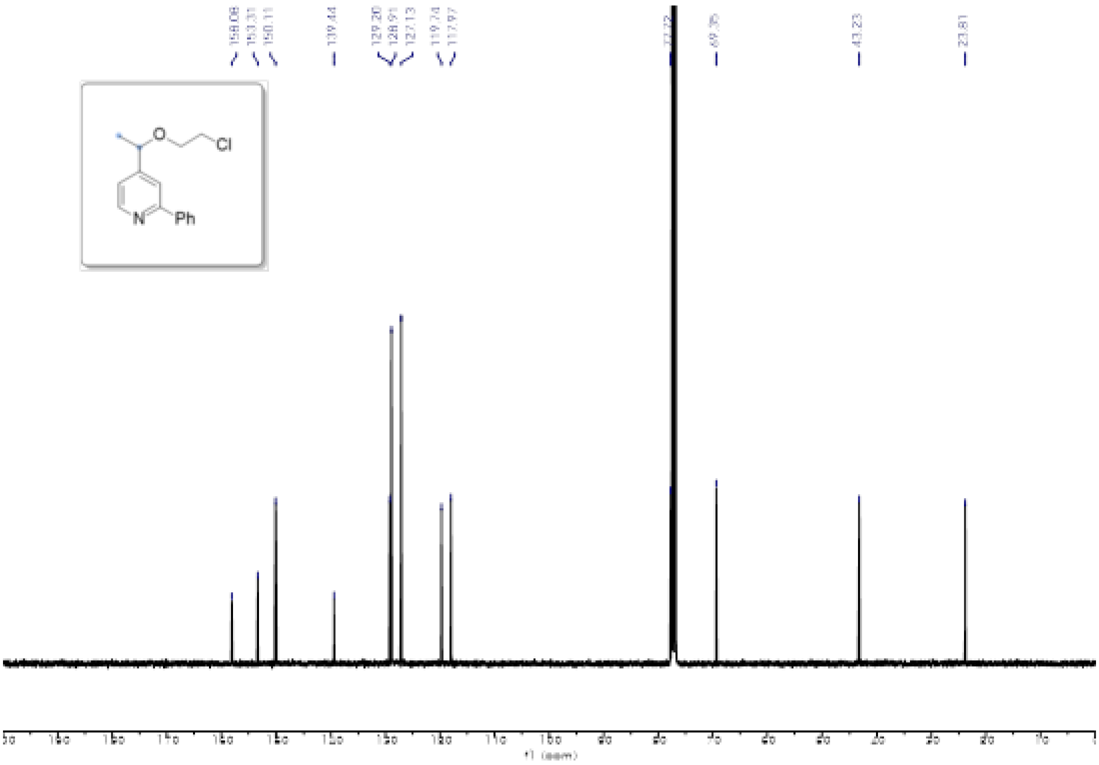

2-(1-(2-phenylpyridin-4-yl)ethoxy)ethan-1-ol (6an).

500 MHz, ^{1}H NMR in $CDCl_3$

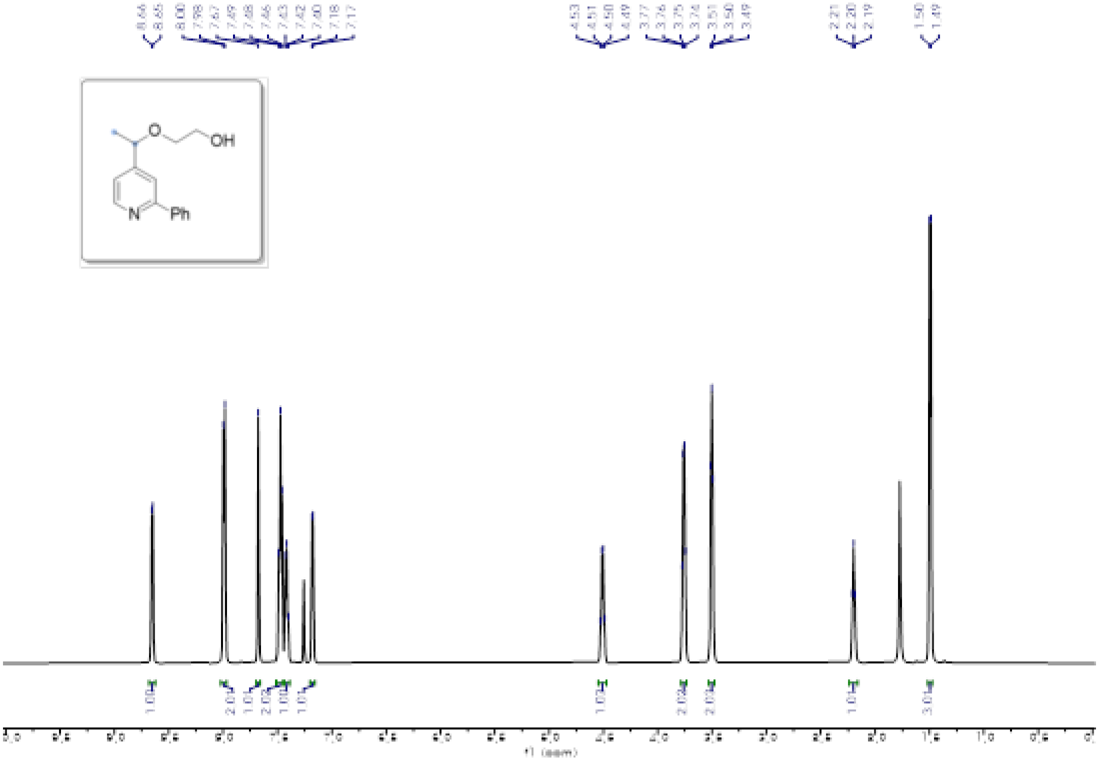

100 MHz, ^{13}C NMR in $CDCl_3$

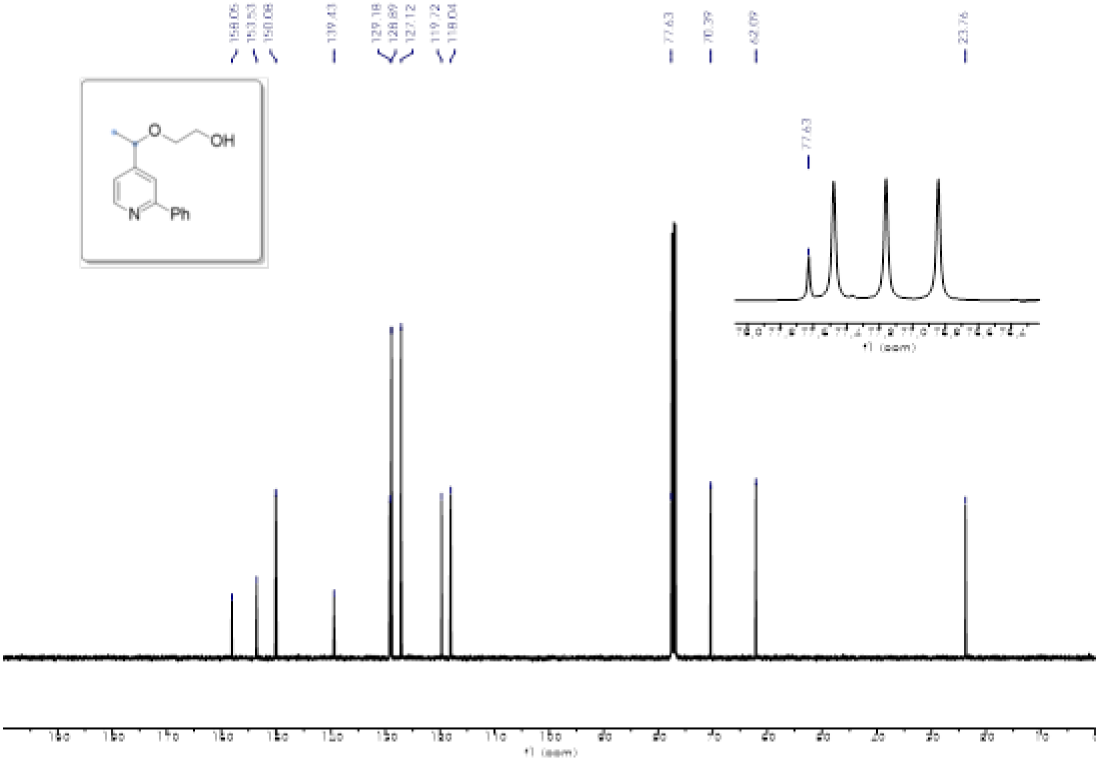

N-(1-(2-phenylpyridin-4-yl)ethyl)acetamide (6ao).

500 MHz, ^{1}H NMR in $CDCl_3$

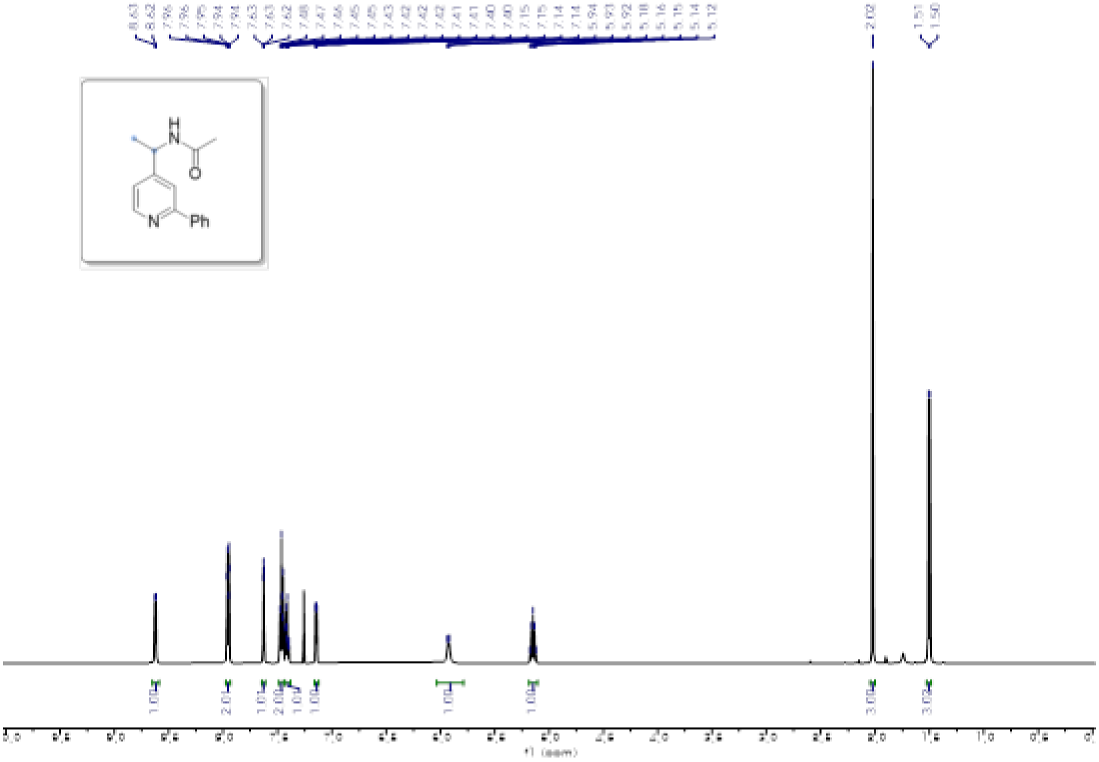

100 MHz, ^{13}C NMR in $CDCl_3$

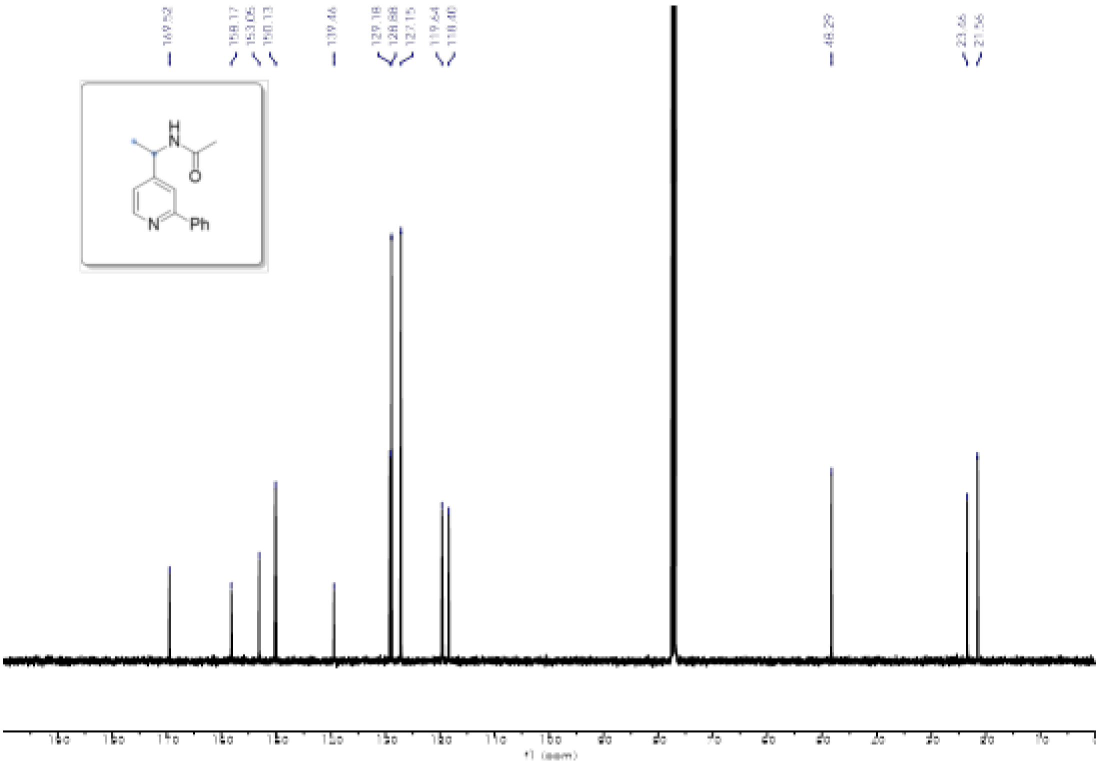

1-(1-(2-phenylpyridin-4-yl)ethyl)pyrrolidin-2-one (6ap).

500 MHz, ^{1}H NMR in $CDCl_3$

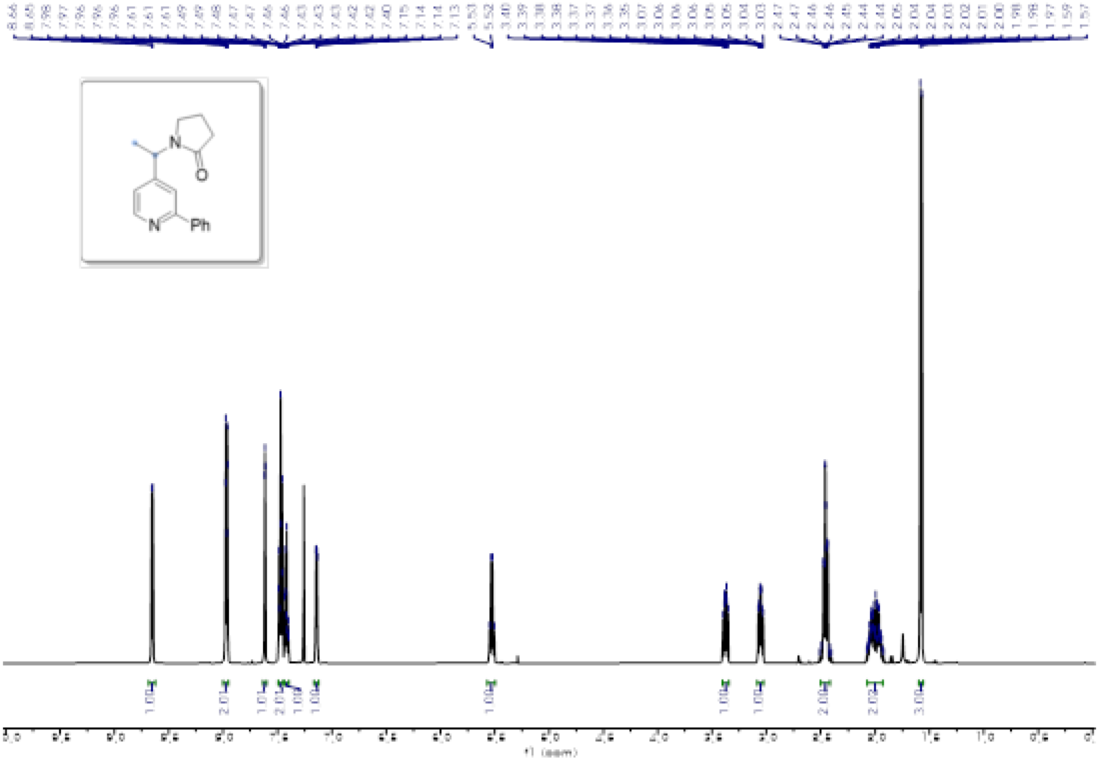

100 MHz, ^{13}C NMR in $CDCl_3$

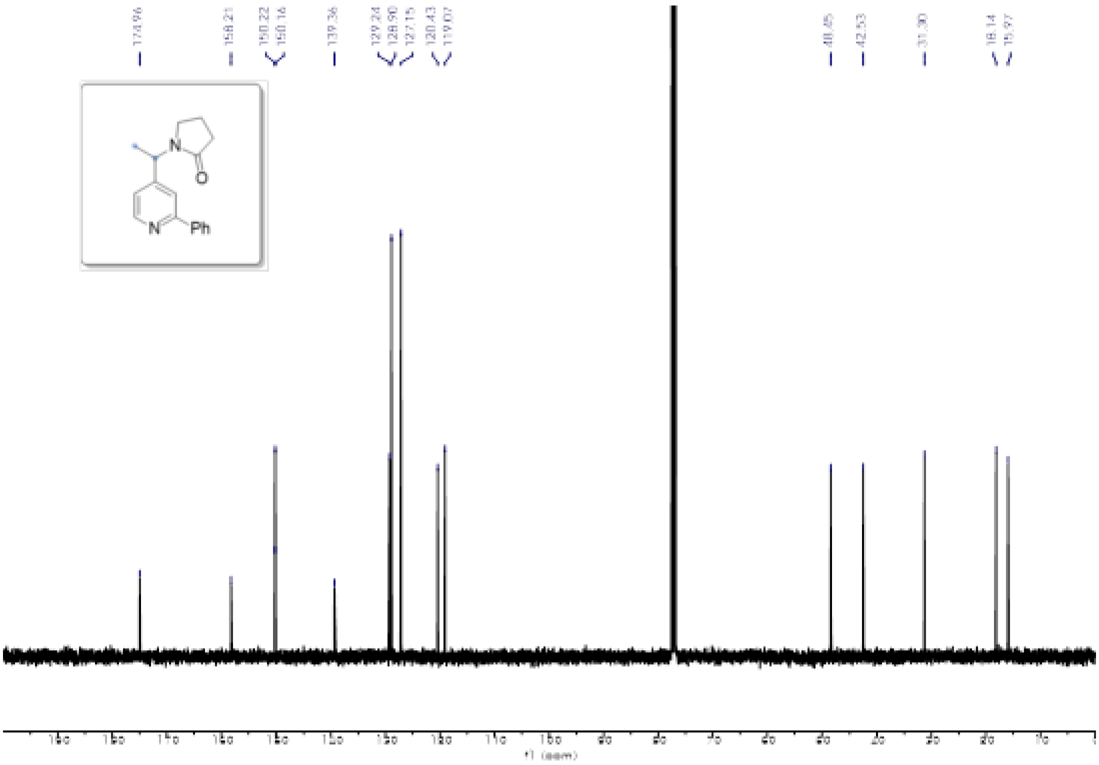

4-(1-(ethylthio)ethyl)-2-phenylpyridine (6aq).

500 MHz, ^{1}H NMR in $CDCl_3$

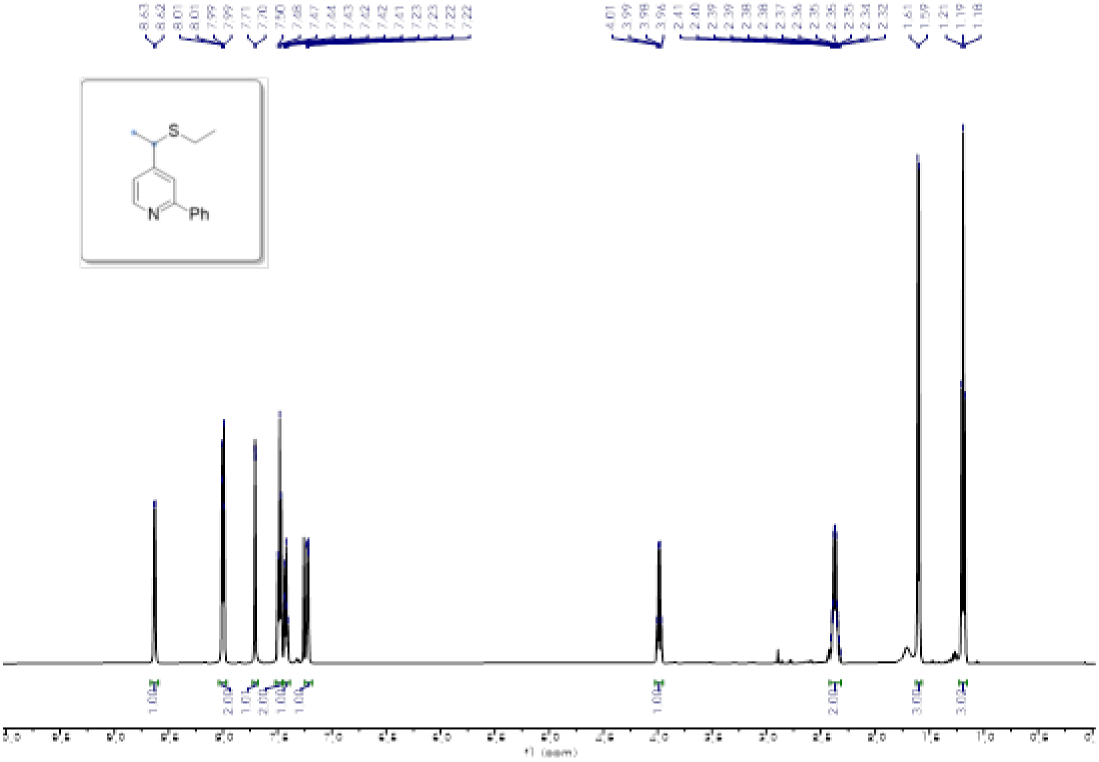

100 MHz, ^{13}C NMR in $CDCl_3$

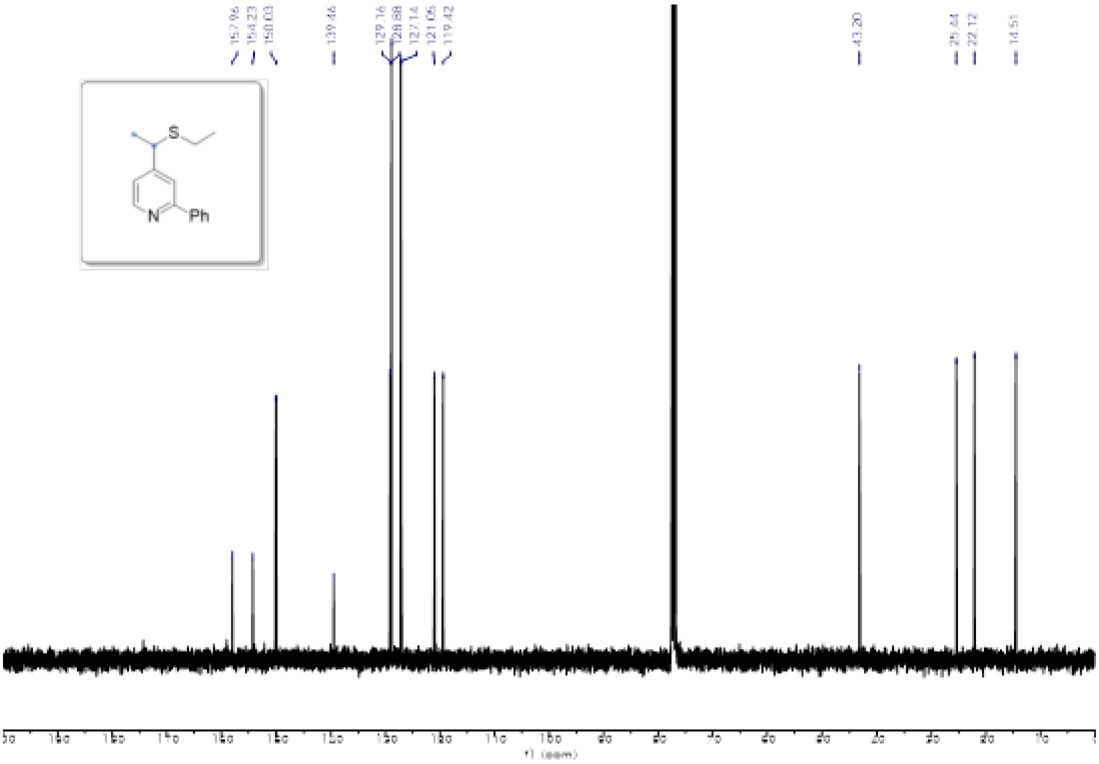

2-phenyl-4-(1-(4,4,5,5-tetramethyl-1,3,2-dioxaborolan-2-yl)hexan-2-yl)pyridine (6ar).

500 MHz, ^{1}H NMR in $CDCl_3$

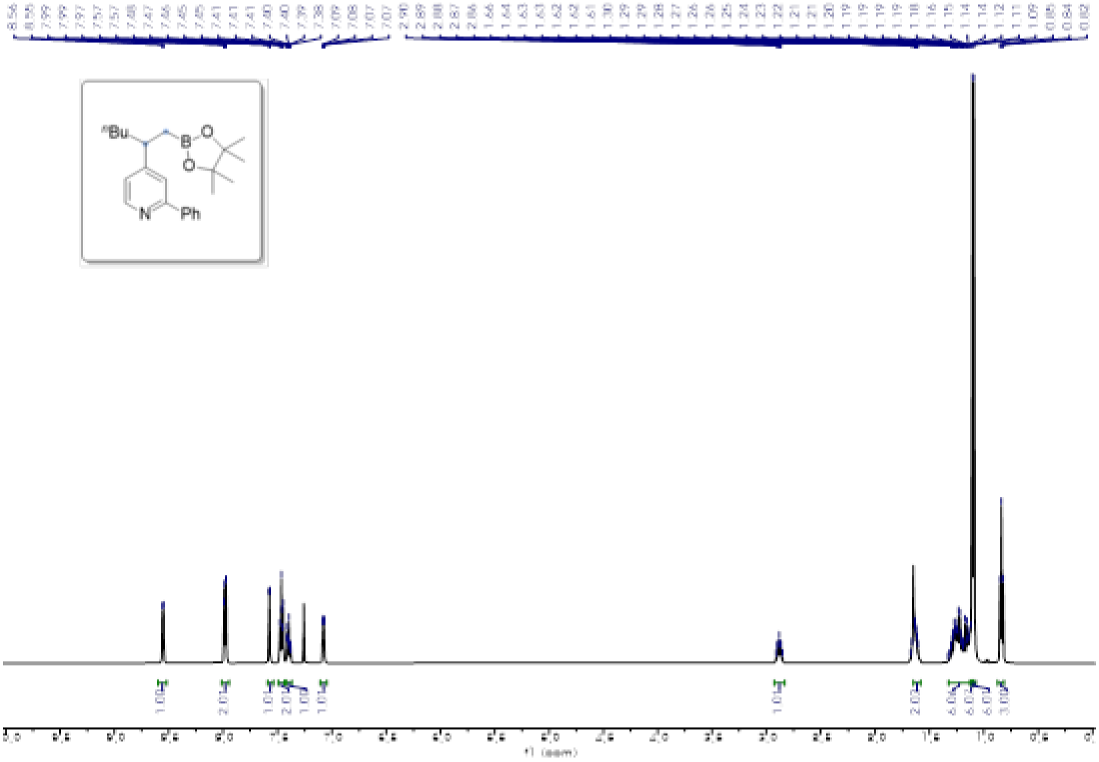

100 MHz, ^{13}C NMR in $CDCl_3$

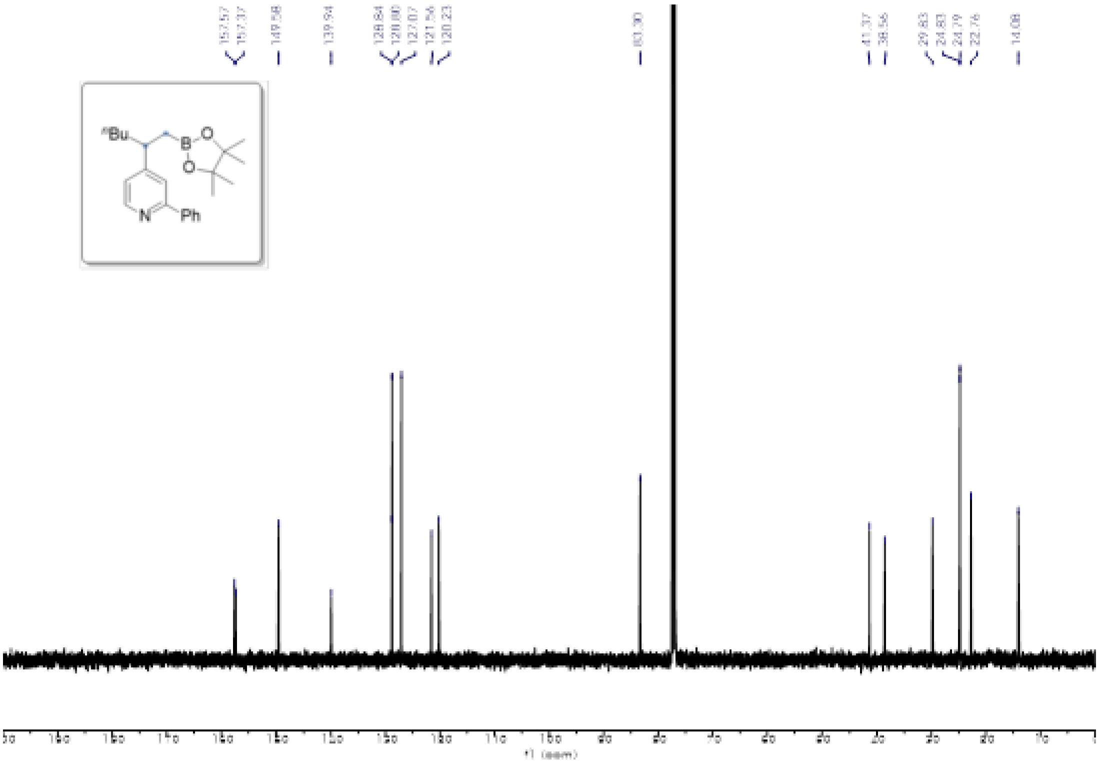

128 MHz, ^{11}B NMR in $CDCl_3$

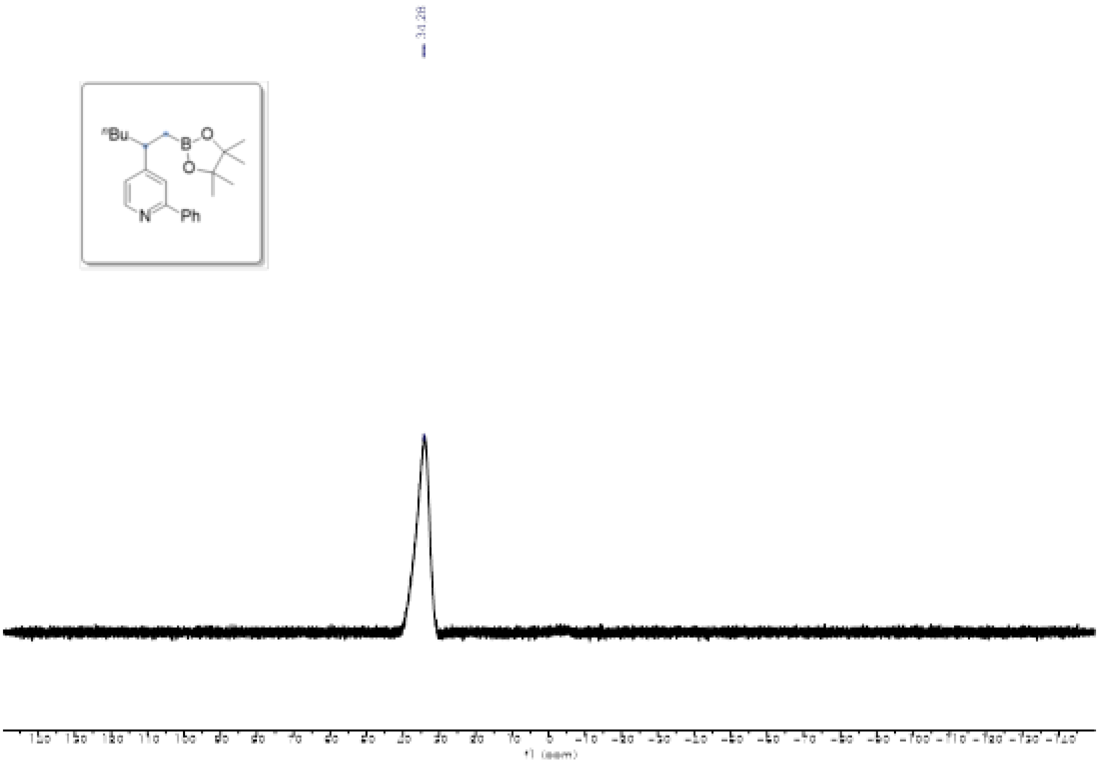

4-methyl-4-(2-phenylpyridin-4-yl)pentan-2-one (6as).

500 MHz, ^{1}H NMR in $CDCl_3$

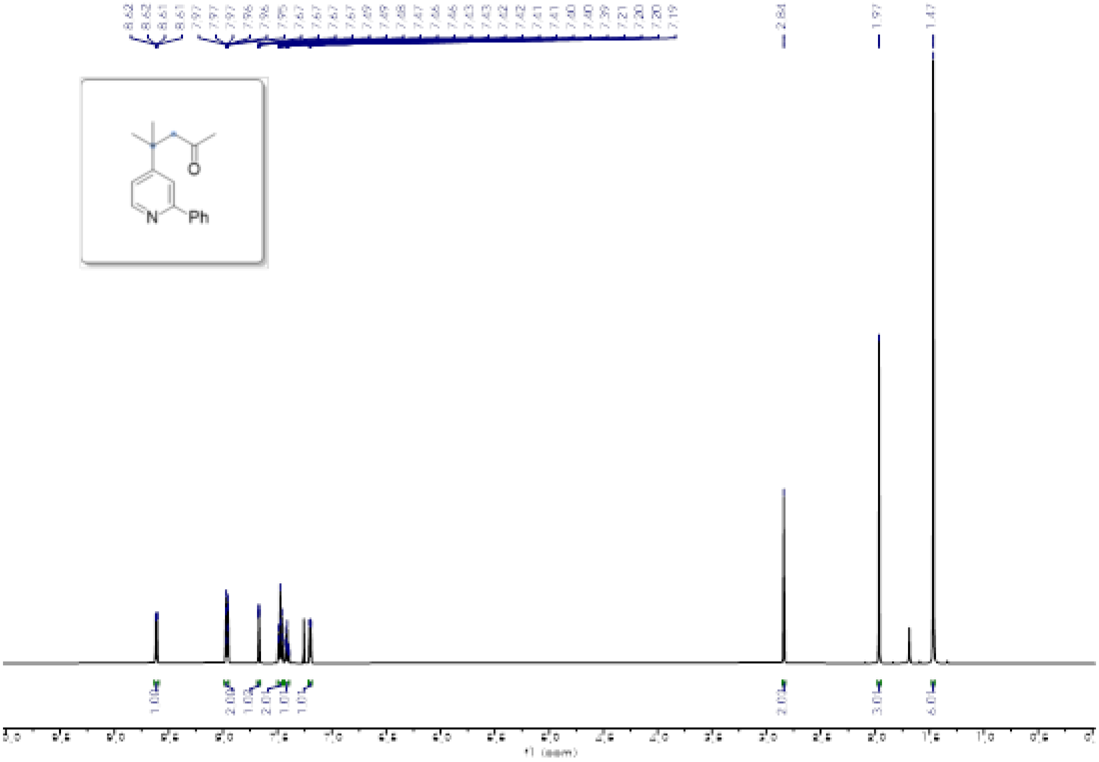

100 MHz, ^{13}C NMR in $CDCl_3$

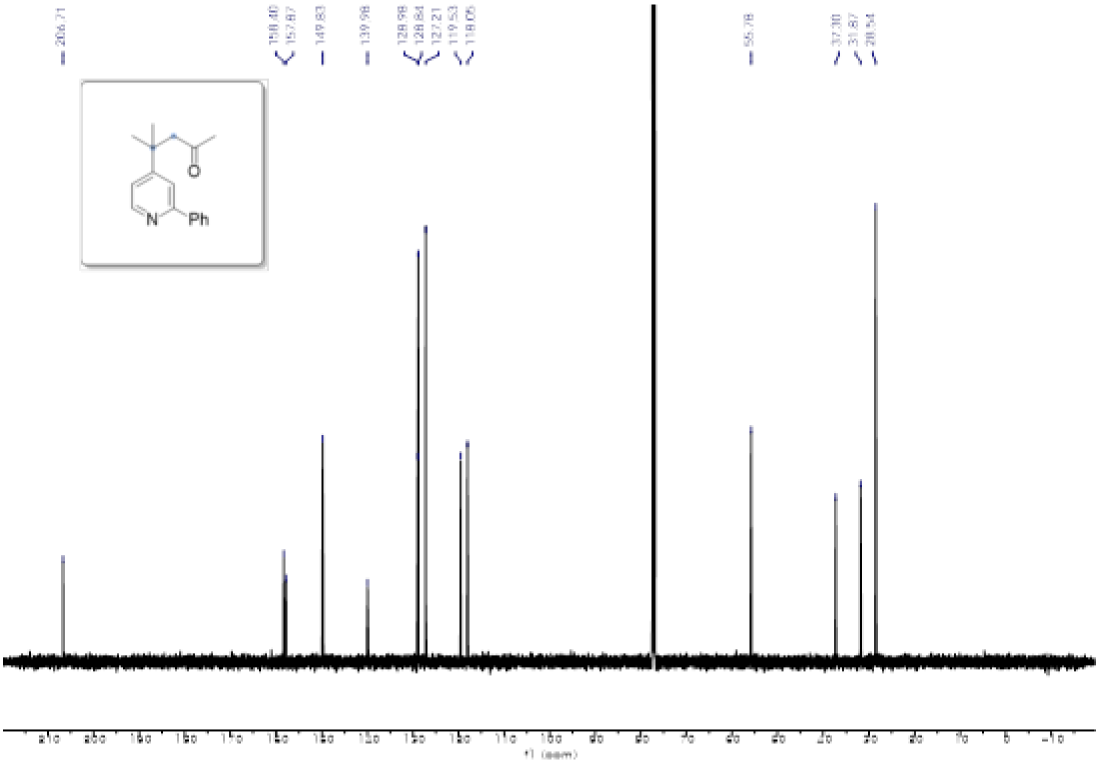

3-methyl-1-phenyl-3-(2-phenylpyridin-4-yl)butan-1-one (6at).

500 MHz, ^{1}H NMR in $CDCl_3$

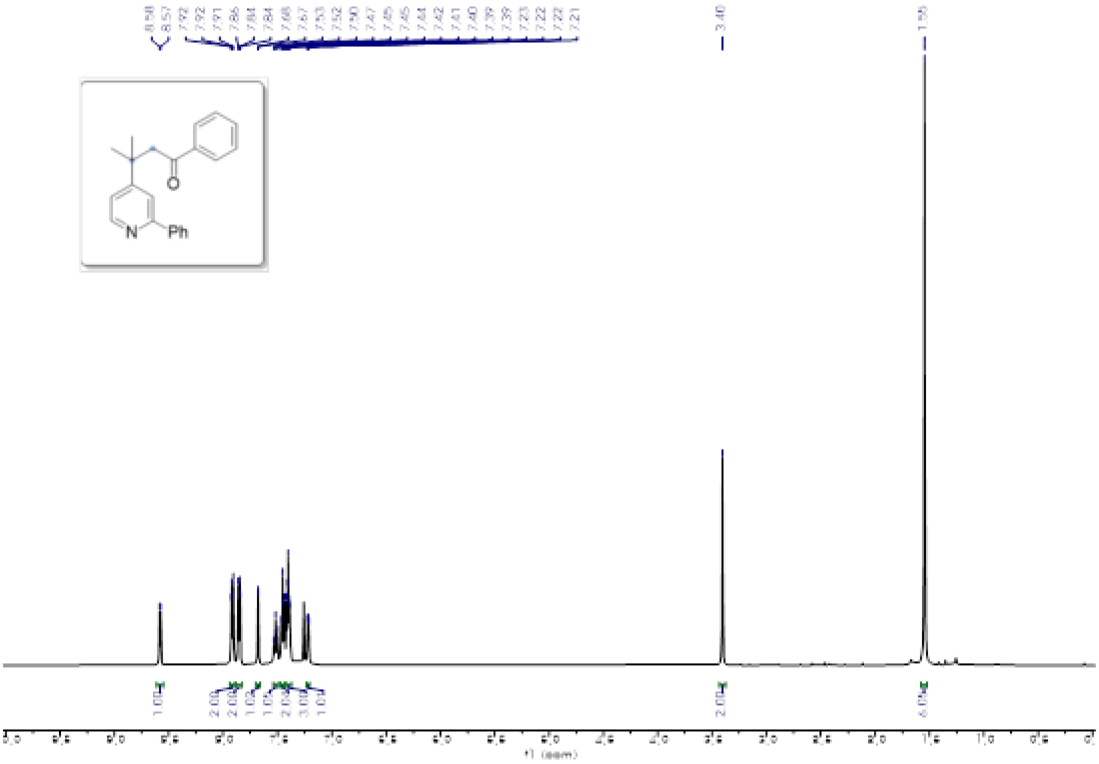

125 MHz, ^{13}C NMR in $CDCl_3$

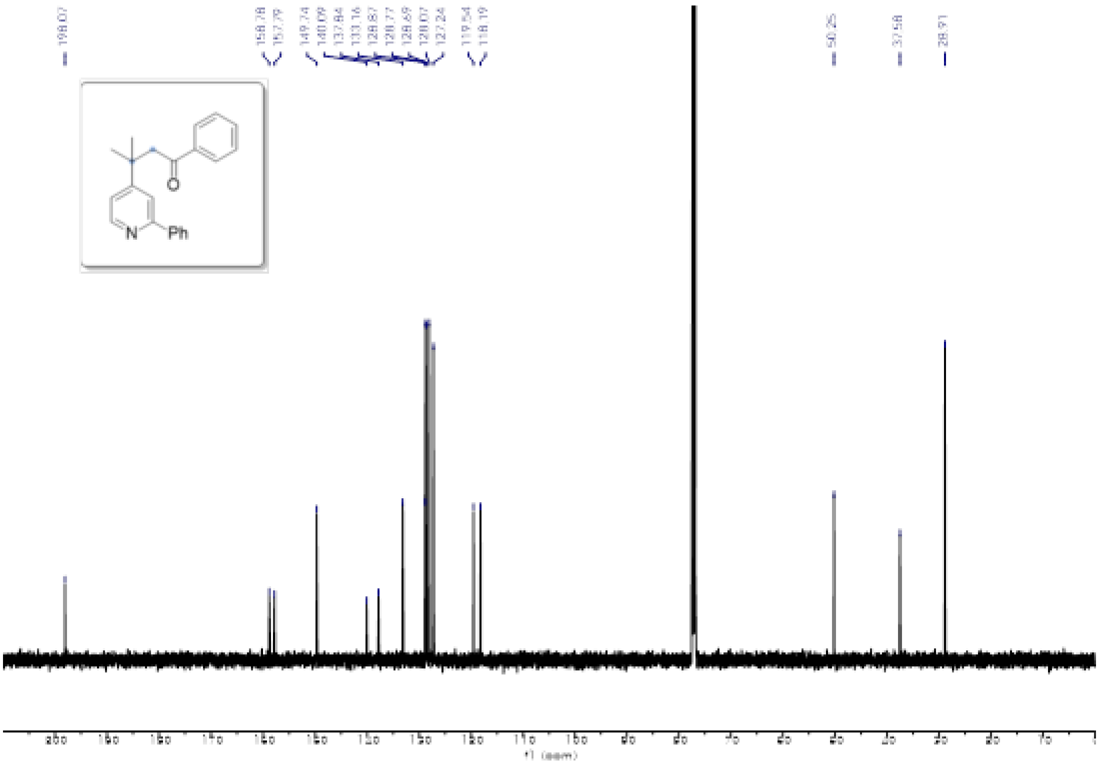

3-methyl-1-(1-methyl-1H-imidazol-2-yl)-3-(2-phenylpyridin-4-yl)butan-1-one (6au).

500 MHz, ^{1}H NMR in CD_2Cl_2

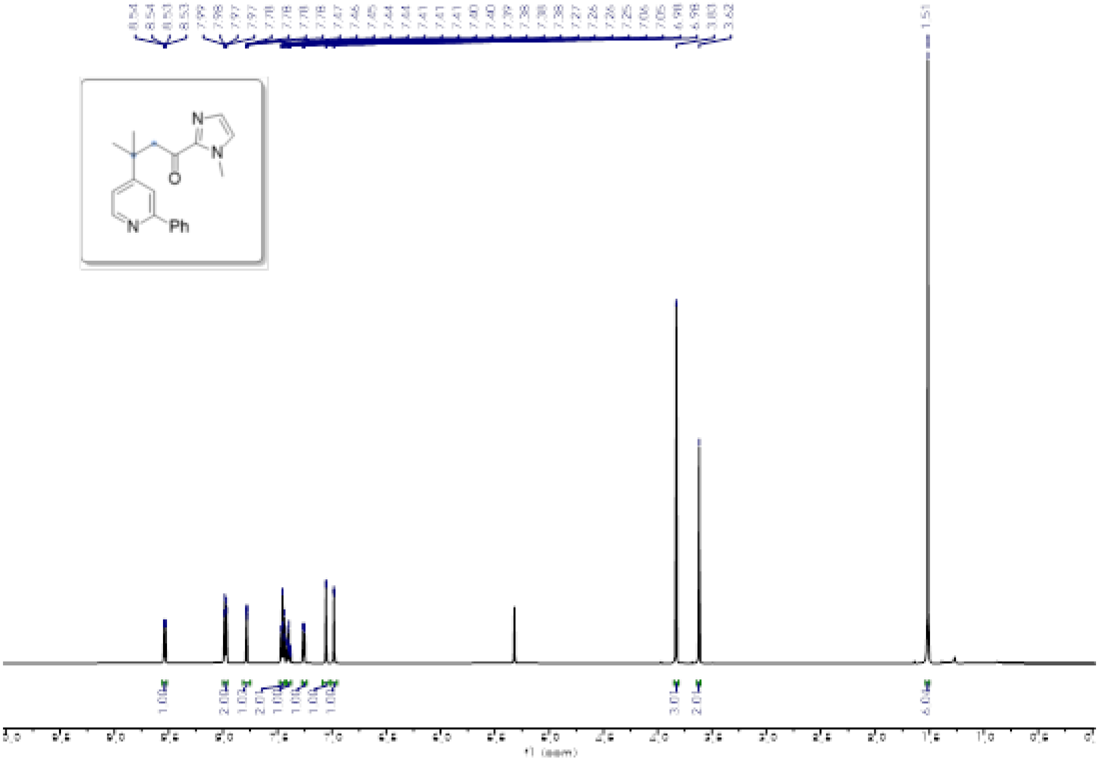

125 MHz, ^{13}C NMR in CD_2Cl_2

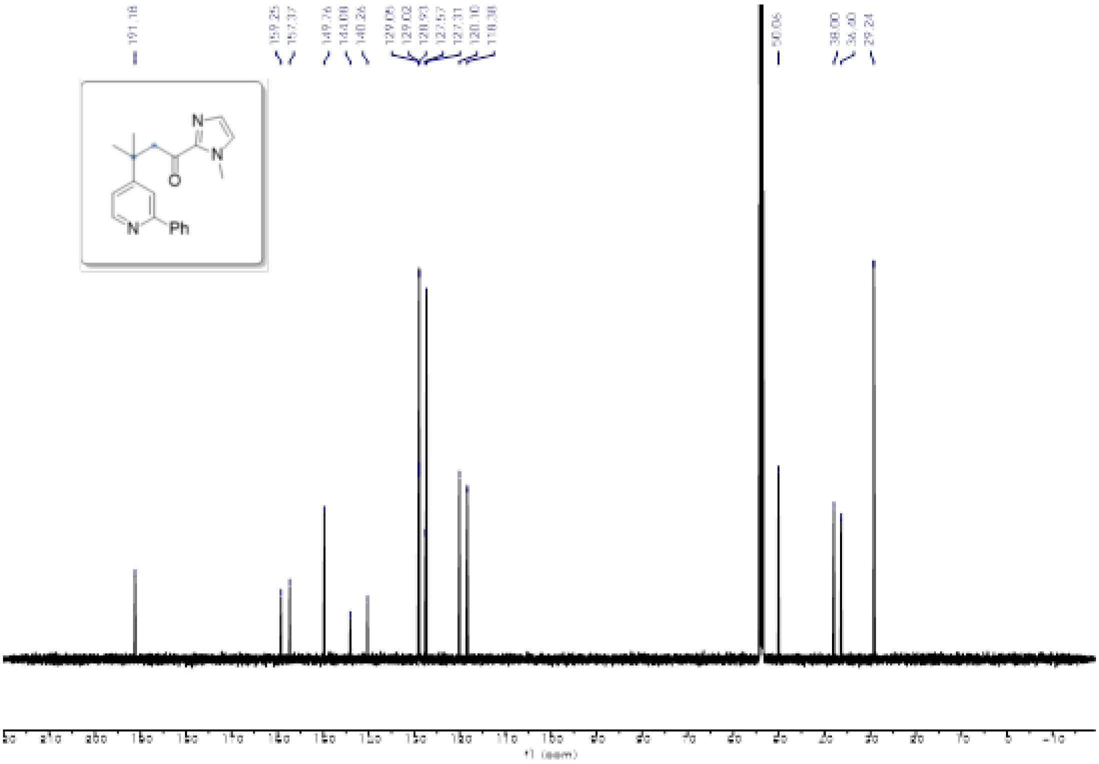

ethyl 3-methyl-3-(2-phenylpyridin-4-yl)butanoate (6av).

500 MHz, ^{1}H NMR in $CDCl_3$

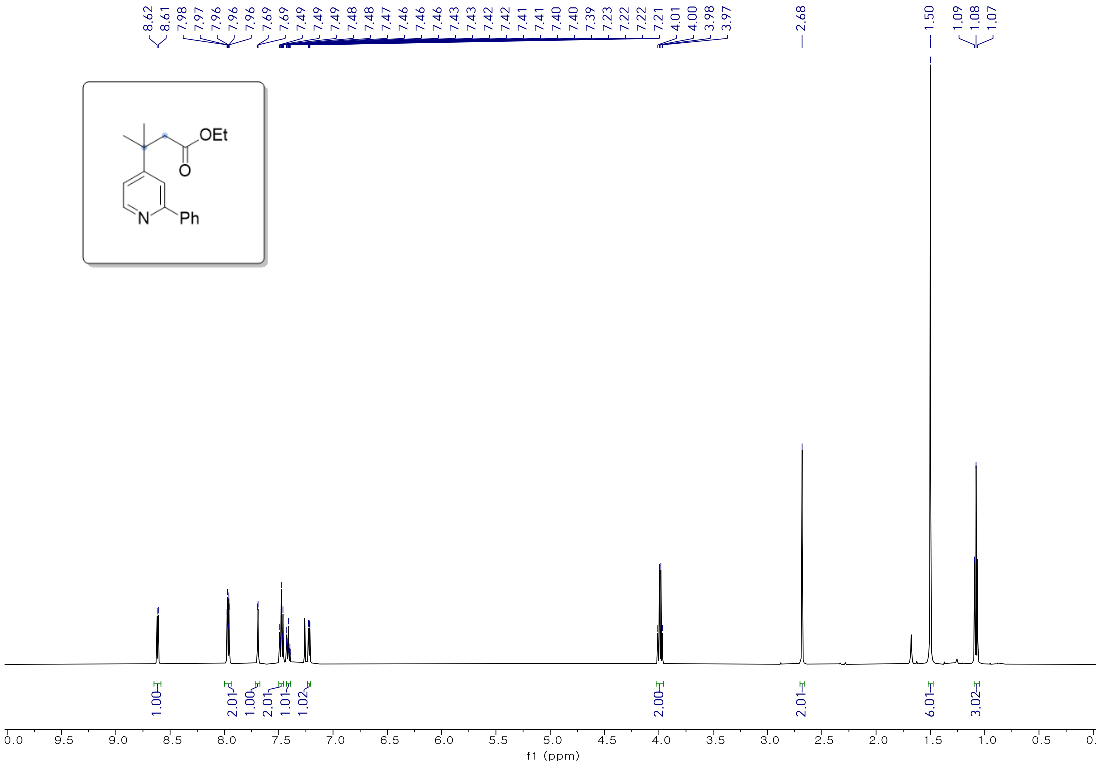

125 MHz, ^{13}C NMR in CD_2Cl_2

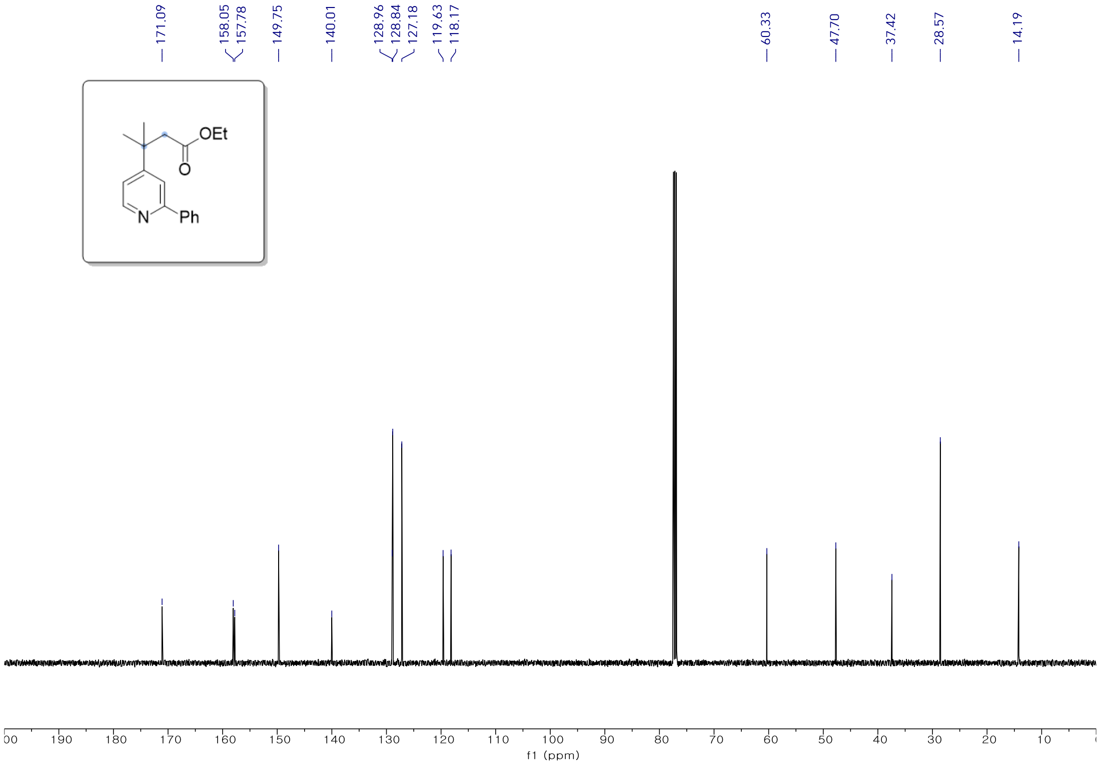

3-methyl-N-phenyl-3-(2-phenylpyridin-4-yl)butanamide (6aw).

500 MHz, ^{1}H NMR in CD_2Cl_2

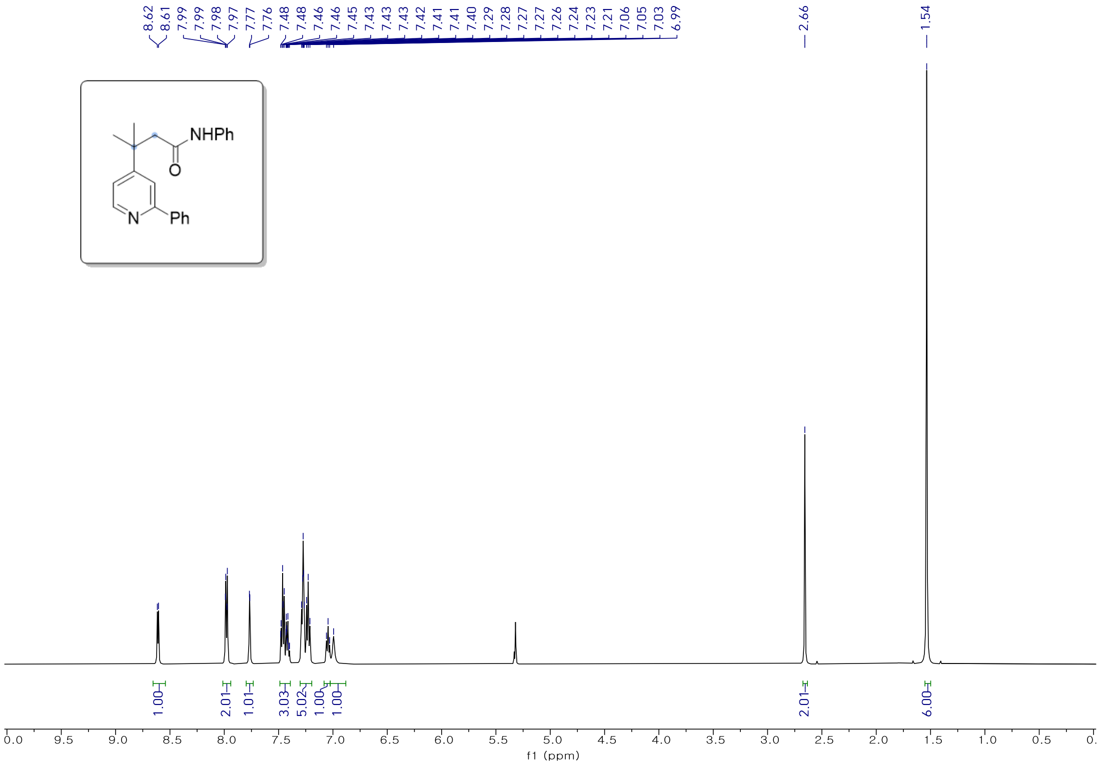

100 MHz, ^{13}C NMR in CD_2Cl_2

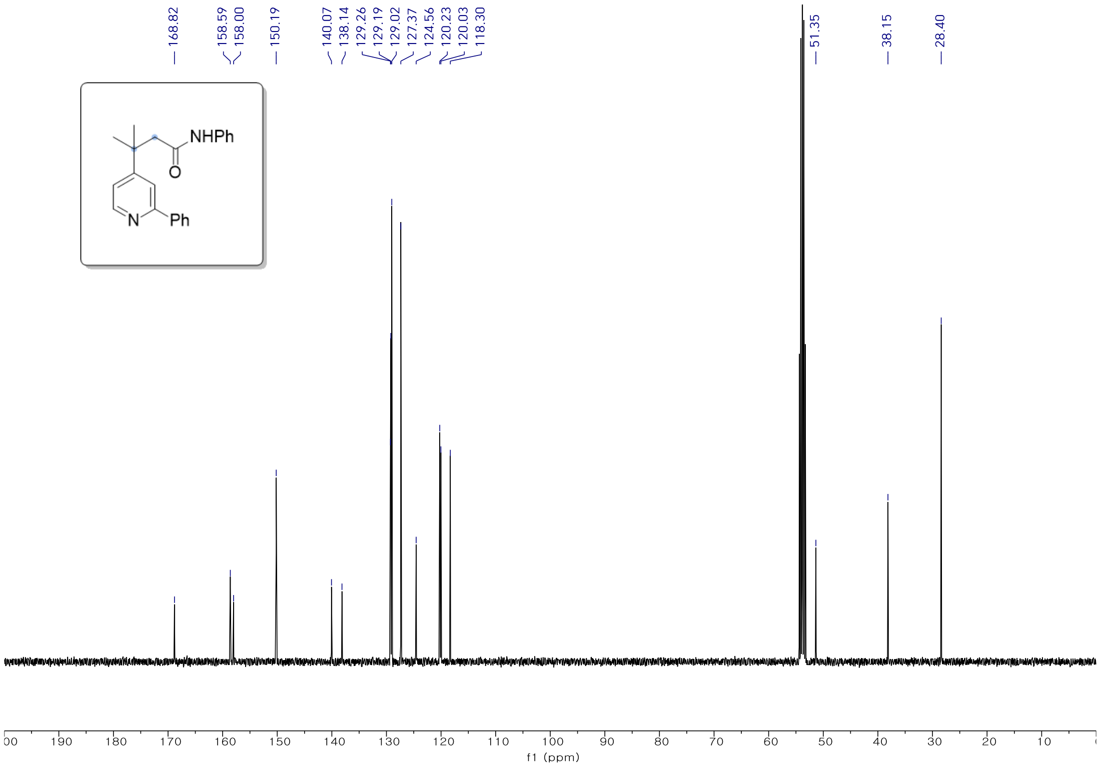

2-(4-bromophenyl)-4-(2-methylhexan-2-yl)pyridine (7a).

600 MHz, ^{1}H NMR in $CDCl_3$

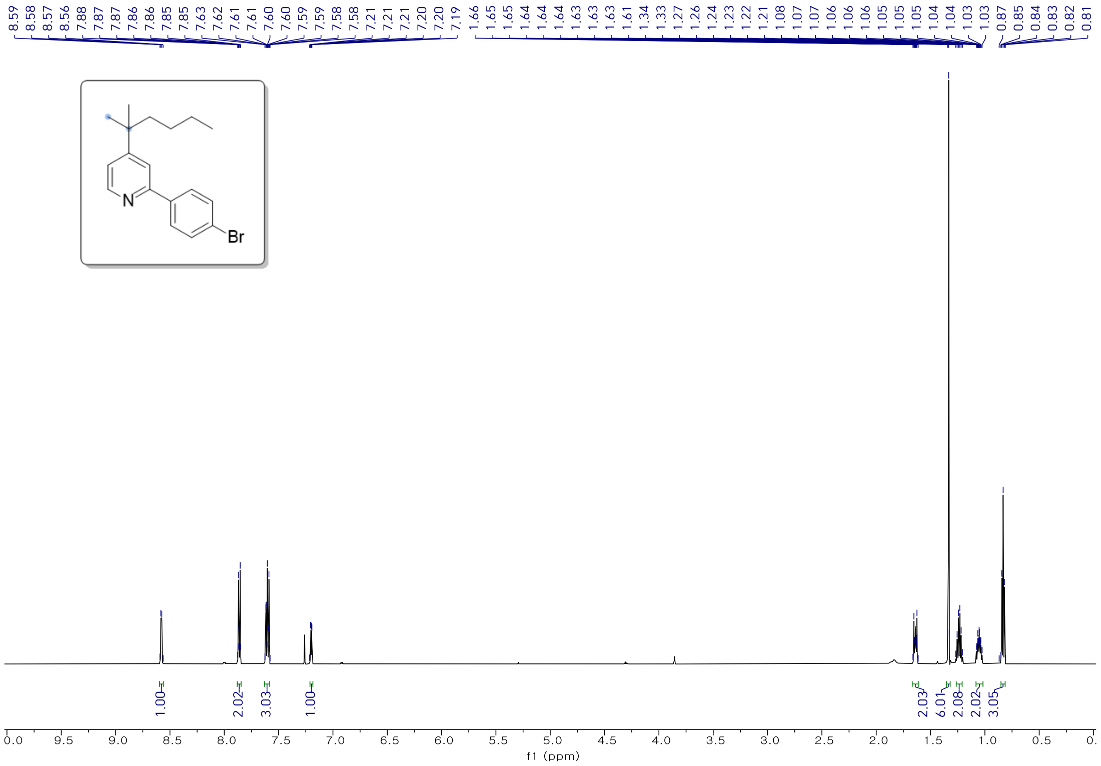

100 MHz, ^{13}C NMR in $CDCl_3$

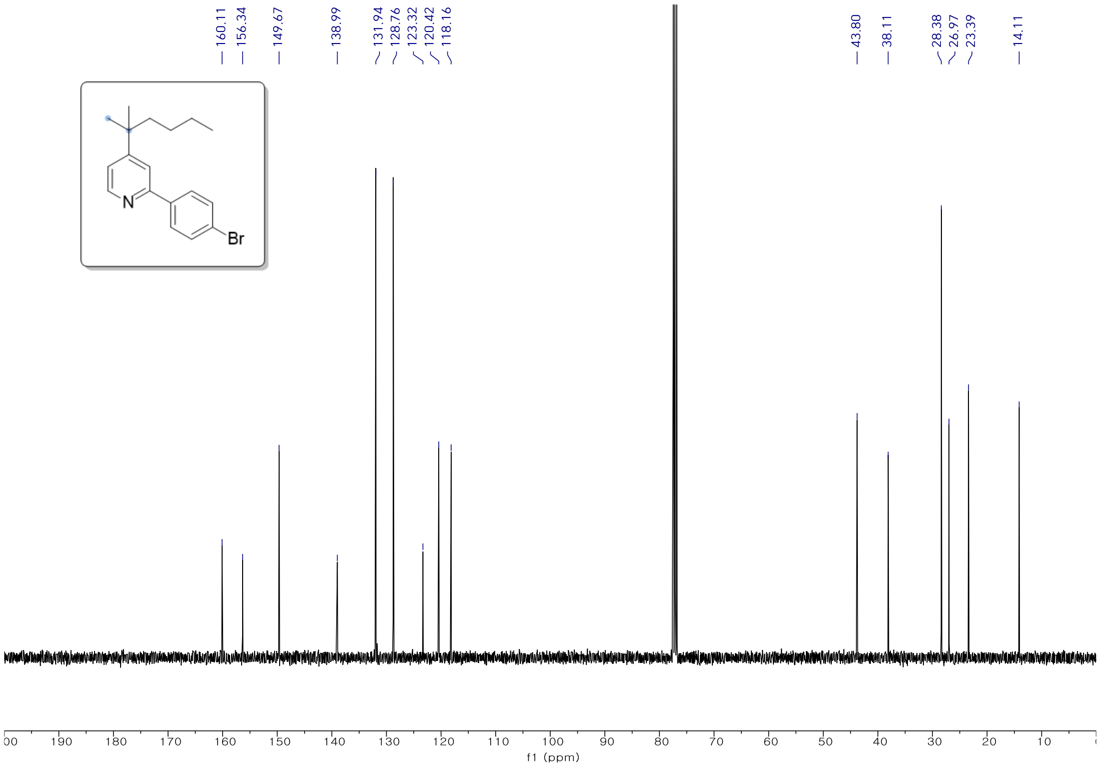

2-(4-methoxyphenyl)-4-(2-methylhexan-2-yl)pyridine (7b).

500 MHz, ^{1}H NMR in $CDCl_3$

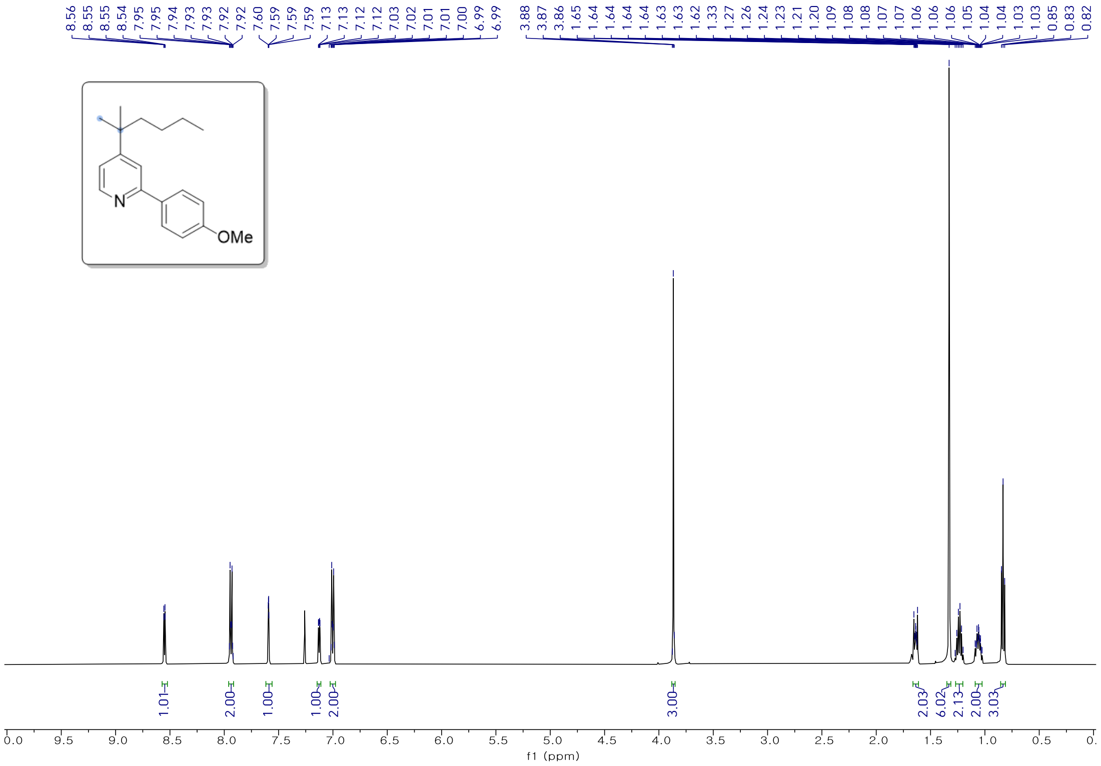

125 MHz, ^{13}C NMR in $CDCl_3$

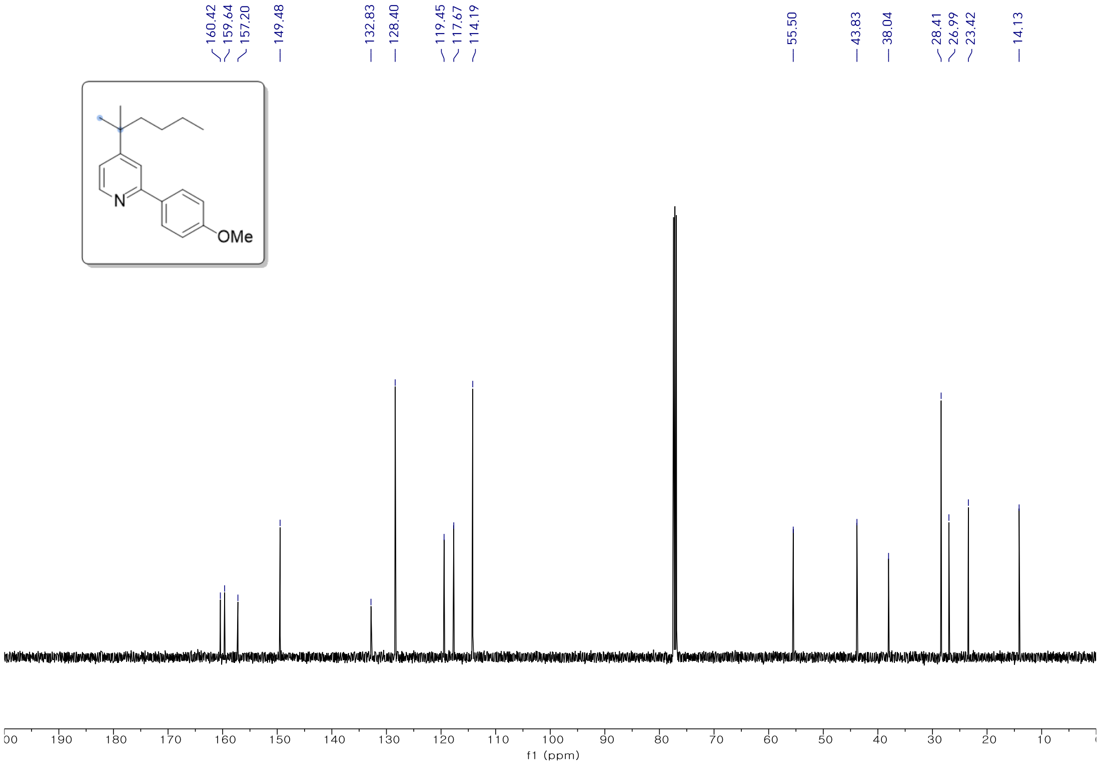

4-(2-methylhexan-2-yl)-2-(4-(trifluoromethyl)phenyl)pyridine (7c).

400 MHz, ^{1}H NMR in $CDCl_3$

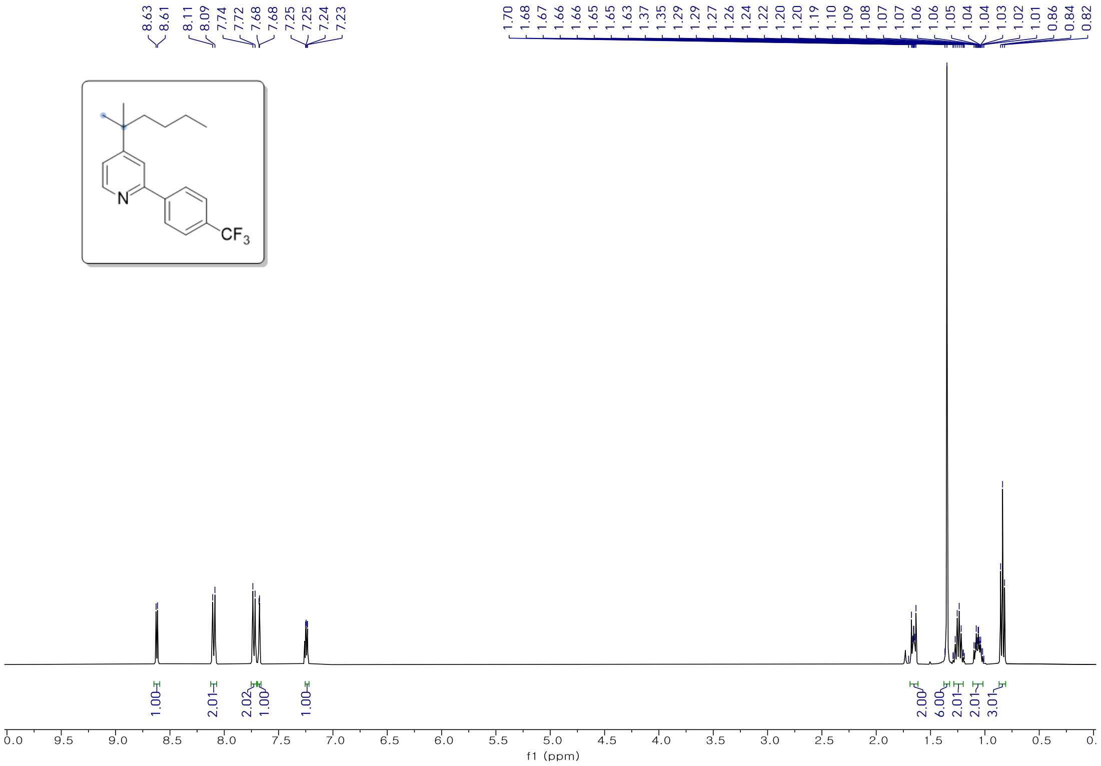

125 MHz, ^{13}C NMR in $CDCl_3$

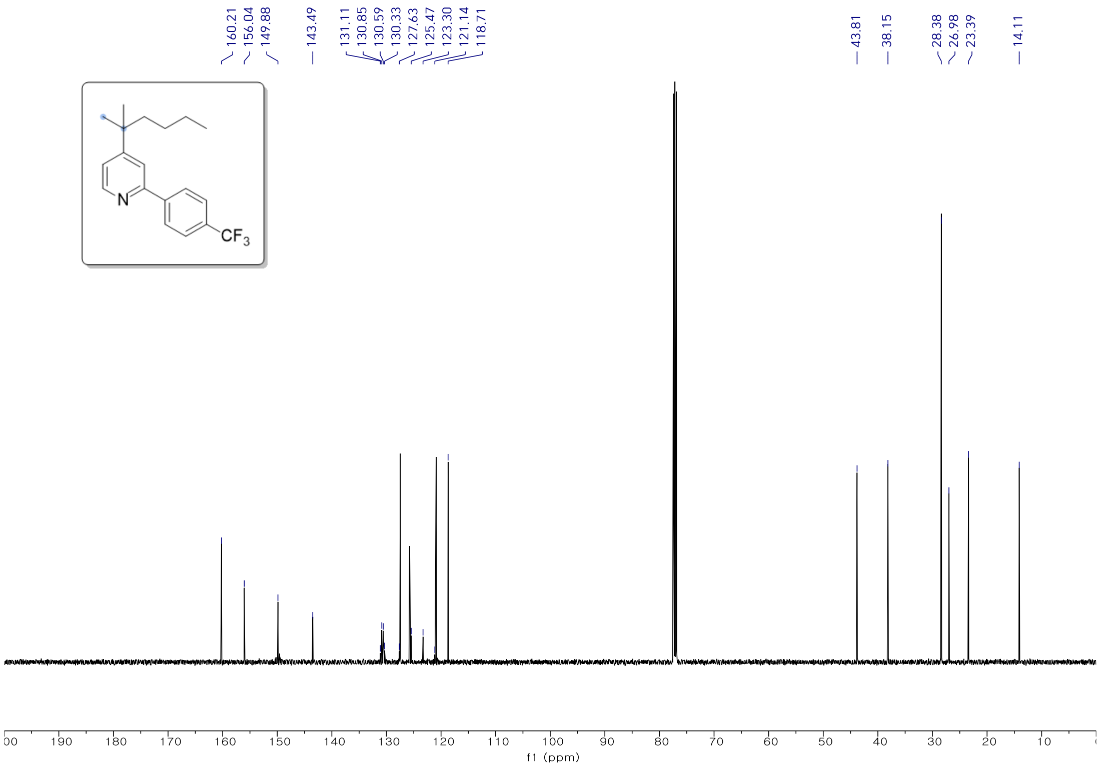

376 MHz, ^{19}F NMR in $CDCl_3$

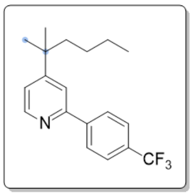

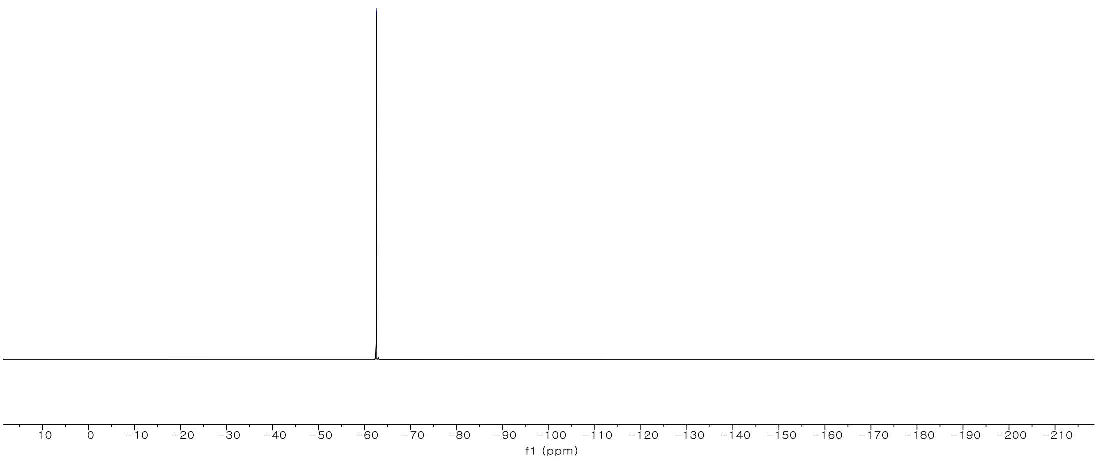

2-(3-bromophenyl)-4-(2-methylhexan-2-yl)pyridine (7d).

500 MHz, ^{1}H NMR in $CDCl_3$

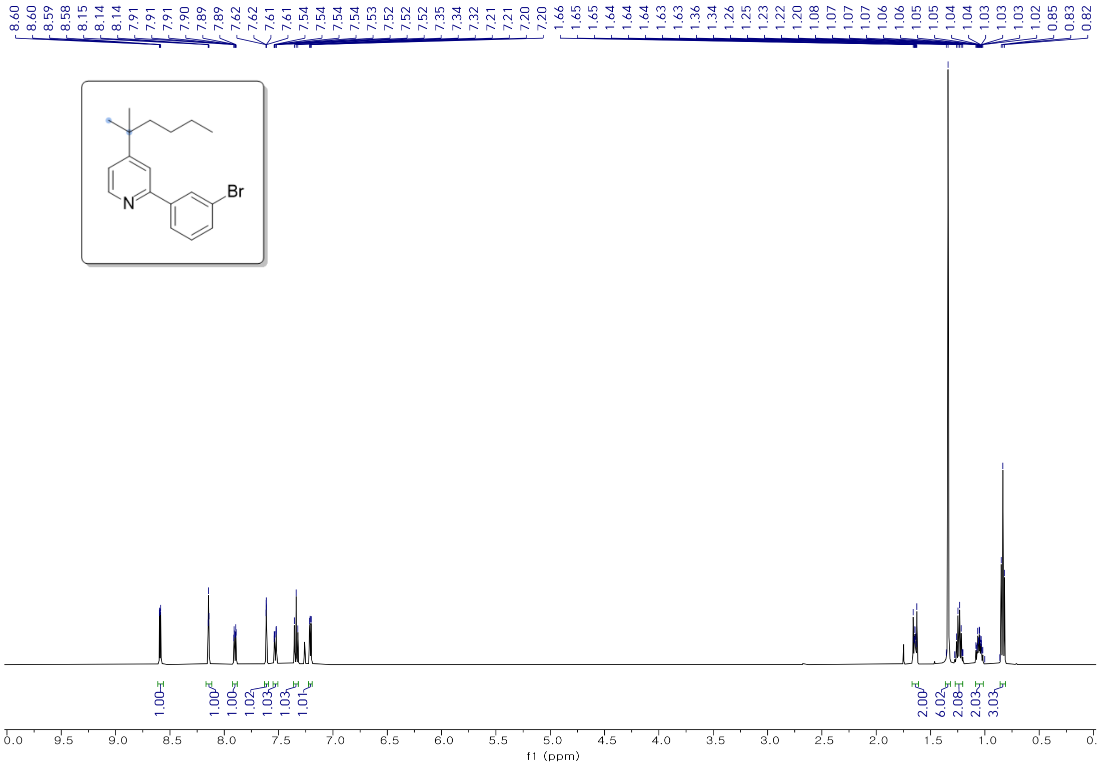

125 MHz, ^{13}C NMR in $CDCl_3$

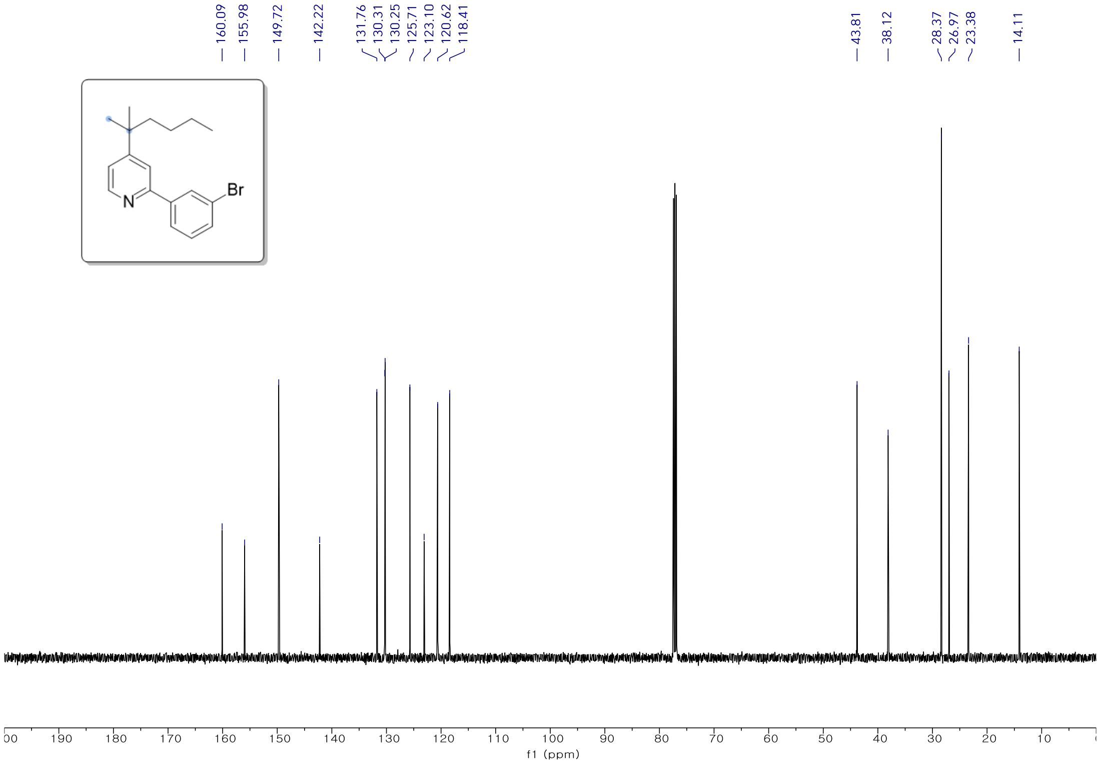

4-(2-methylhexan-2-yl)-2-(thiophen-2-yl)pyridine (7e).

600 MHz, ^{1}H NMR in $CDCl_3$

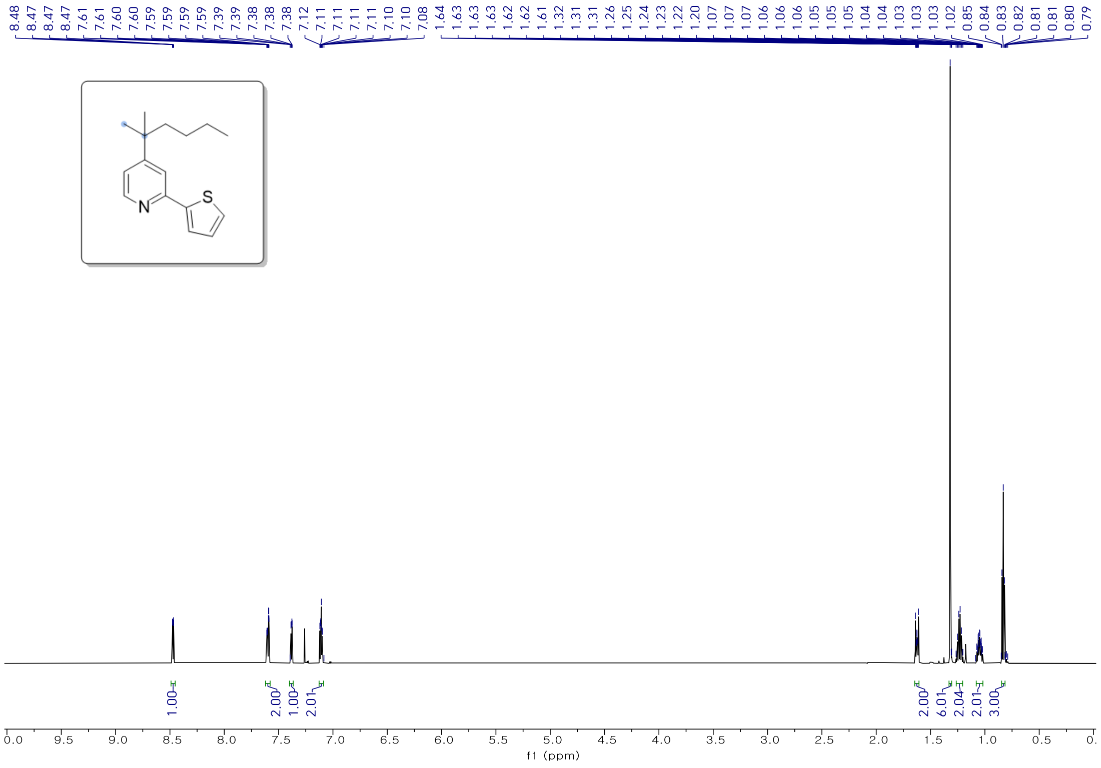

100 MHz, ^{13}C NMR in $CDCl_3$

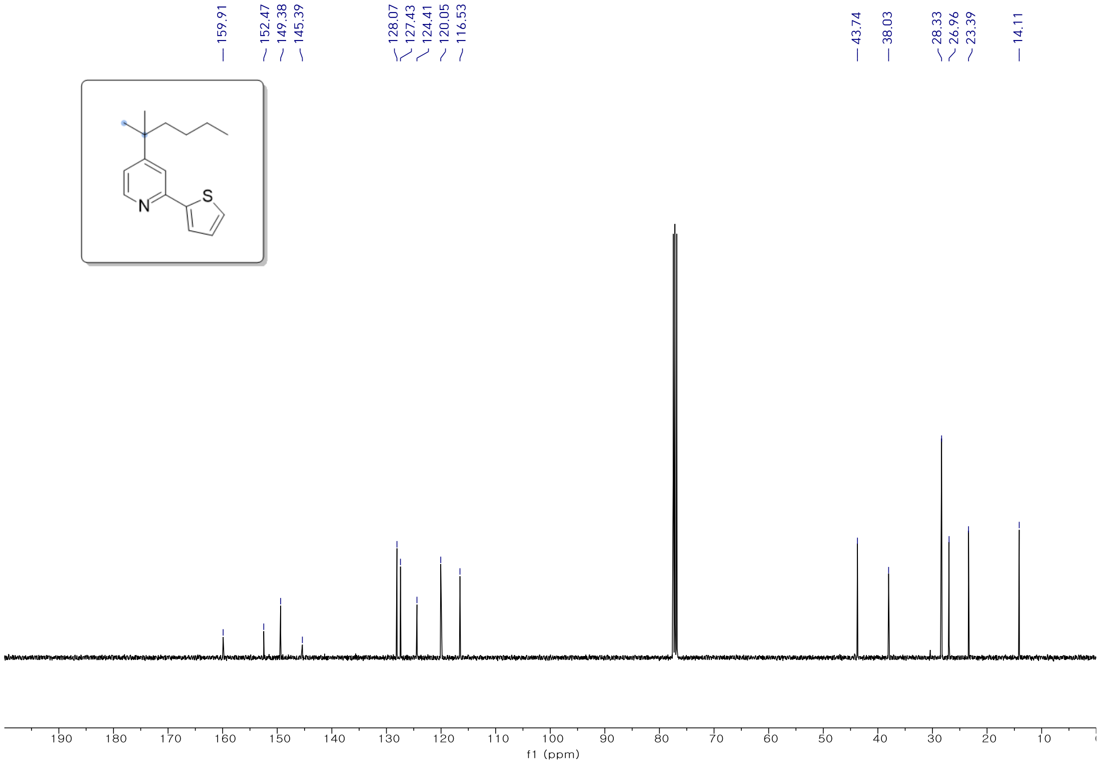

ethyl 4-methyl-4-(2-methylpyridin-4-yl)pentanoate (7f).

500 MHz, ^{1}H NMR in $CDCl_3$

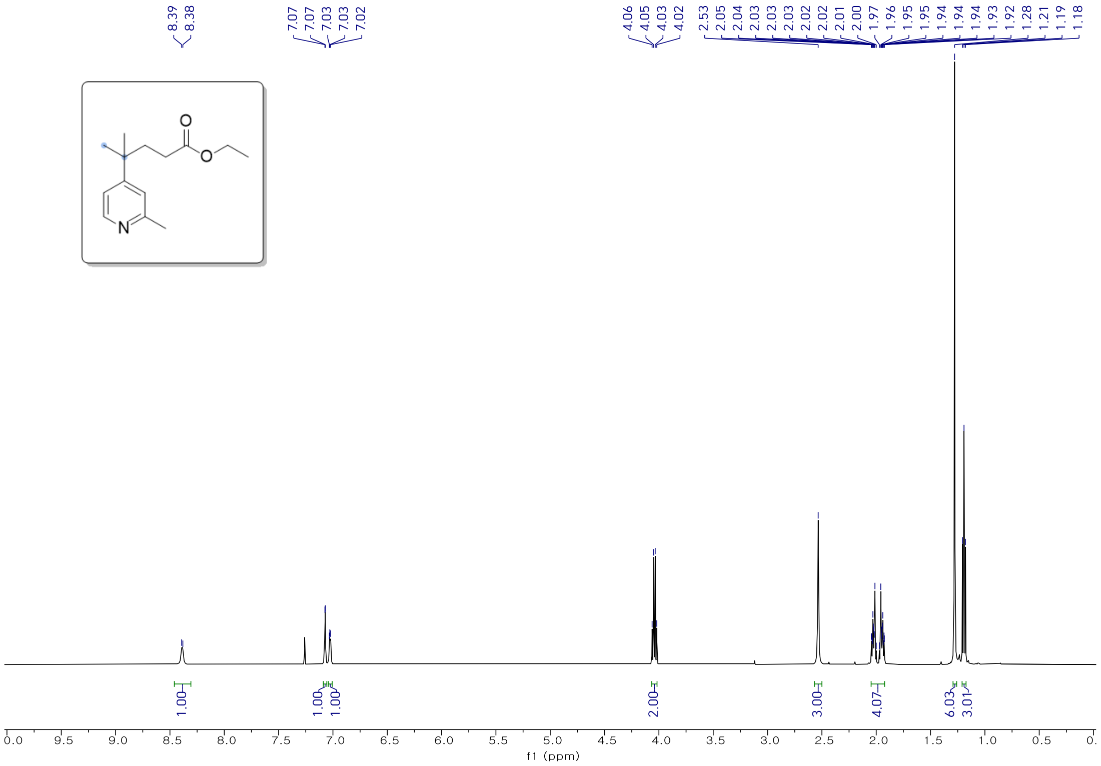

125 MHz, ^{13}C NMR in $CDCl_3$

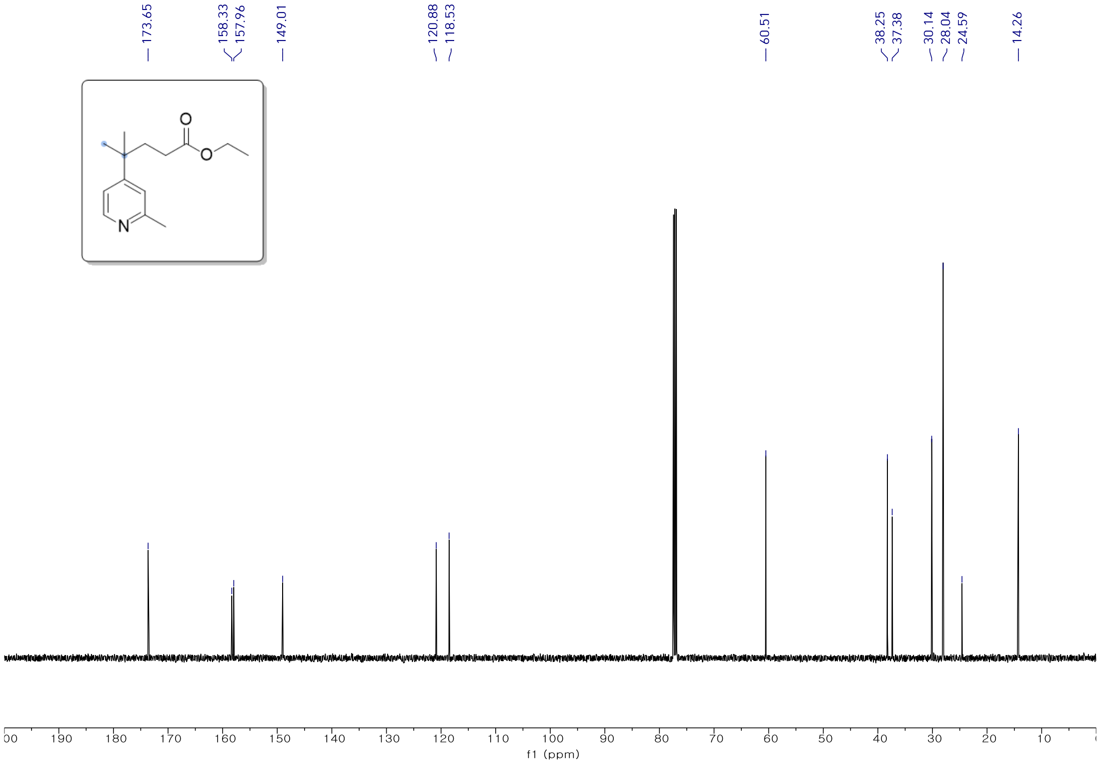

ethyl 4-methyl-4-(2-(trifluoromethyl)pyridin-4-yl)pentanoate (7g).

500 MHz, ^{1}H NMR in $CDCl_3$

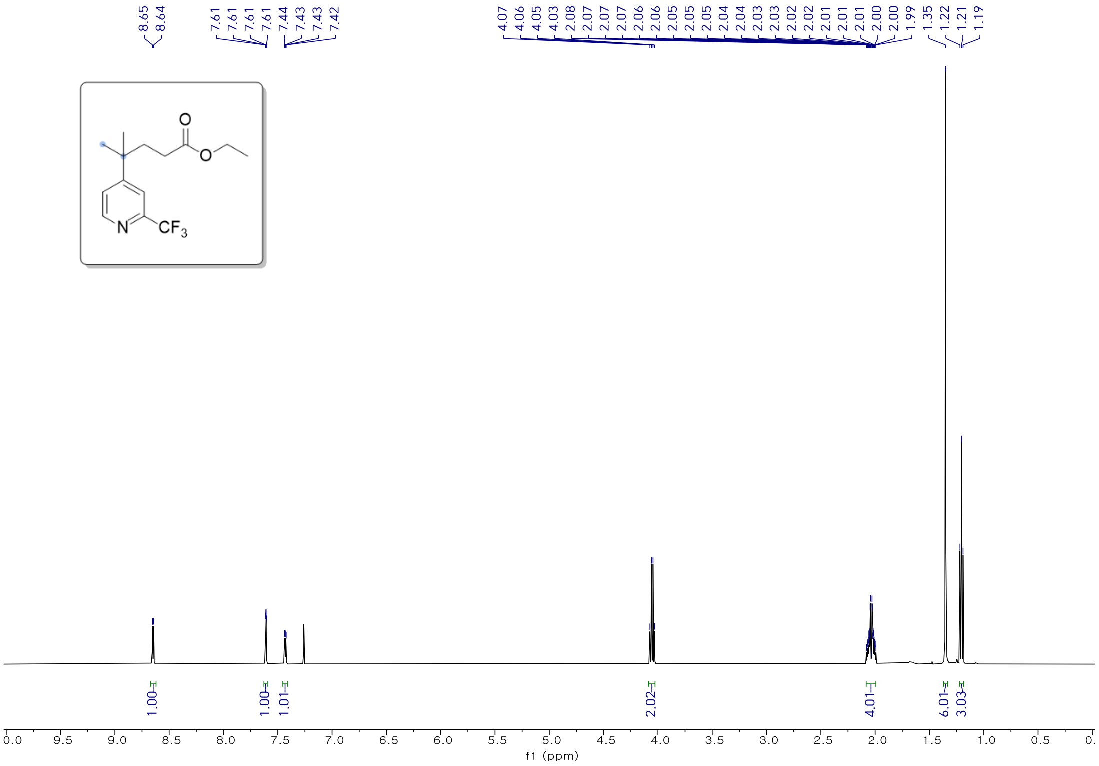

100 MHz, ^{13}C NMR in $CDCl_3$

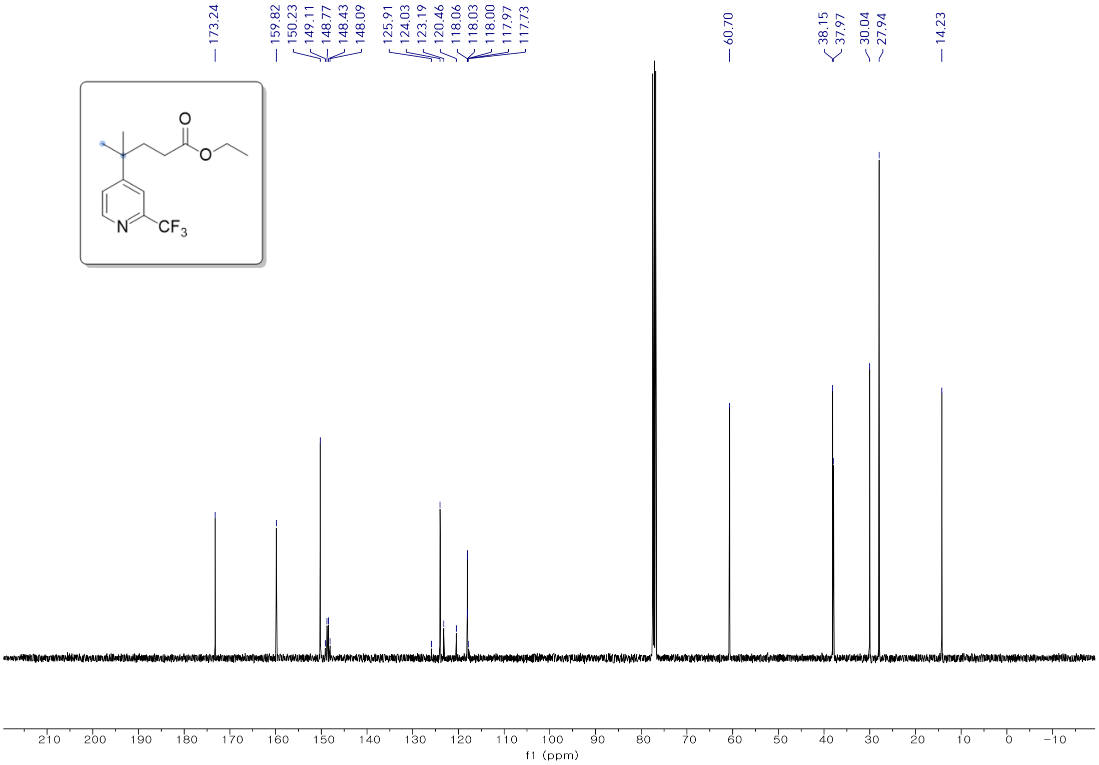

376 MHz, ^{19}F NMR in $CDCl_3$

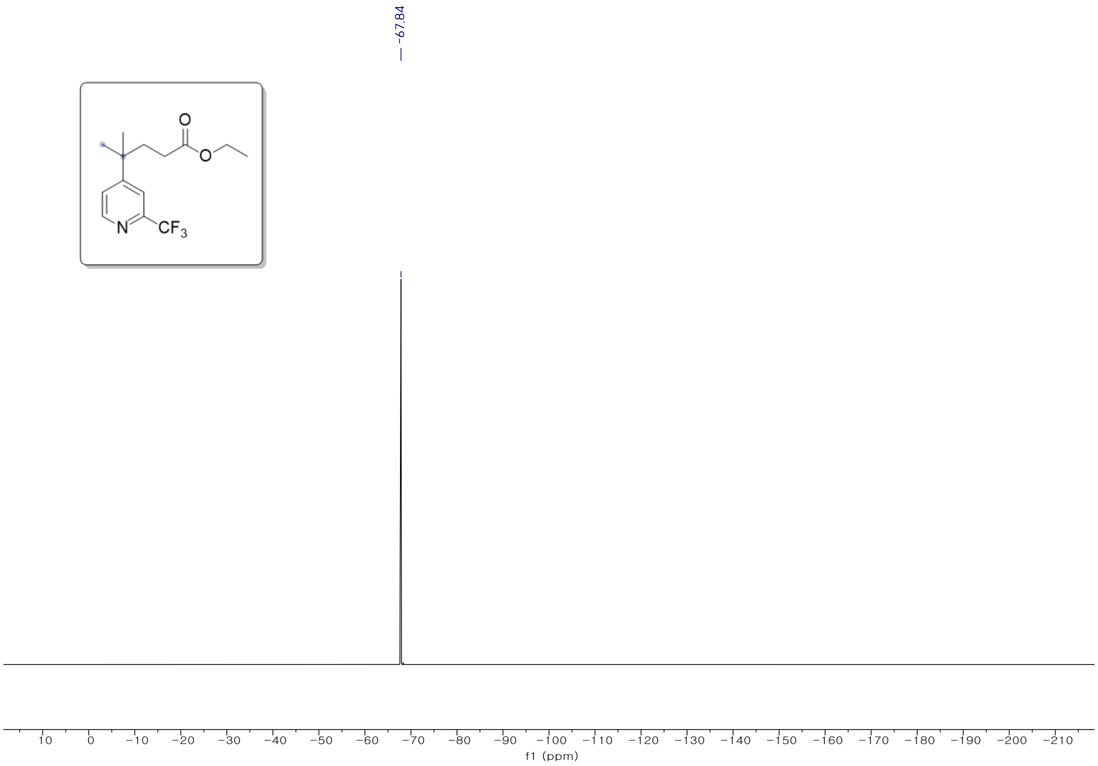

4-(2-methylhexan-2-yl)nicotinonitrile (7h).

400 MHz, ^{1}H NMR in $CDCl_3$

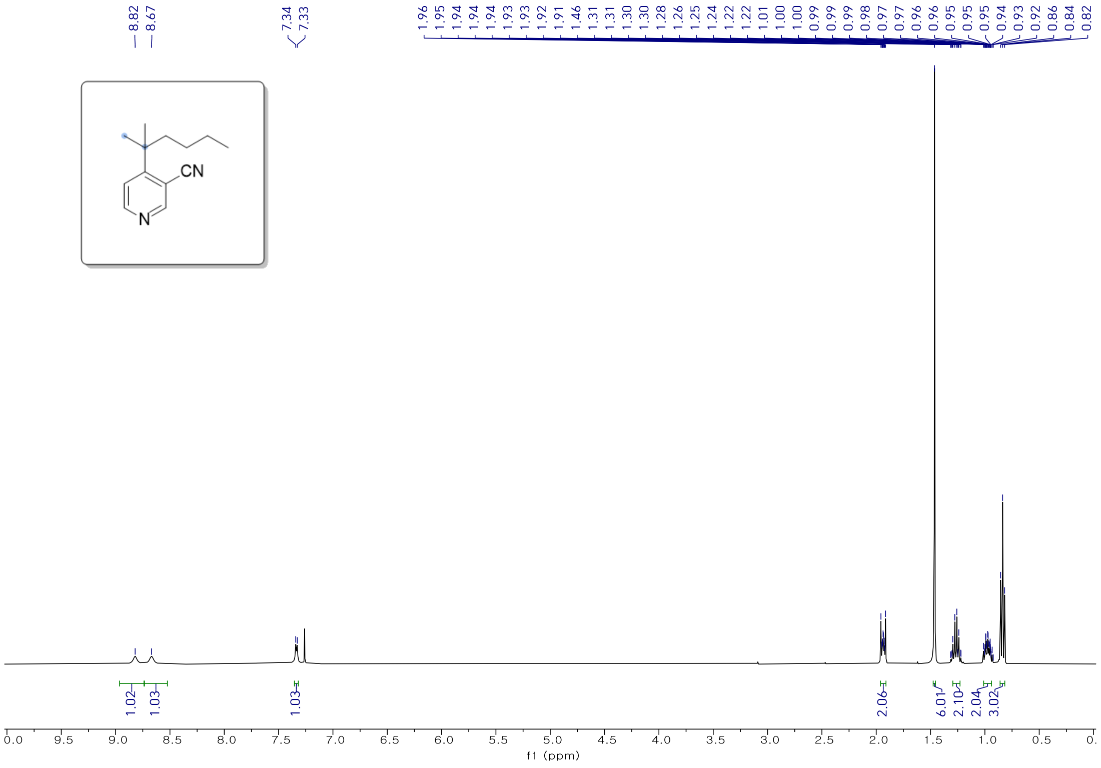

125 MHz, ^{13}C NMR in $CDCl_3$

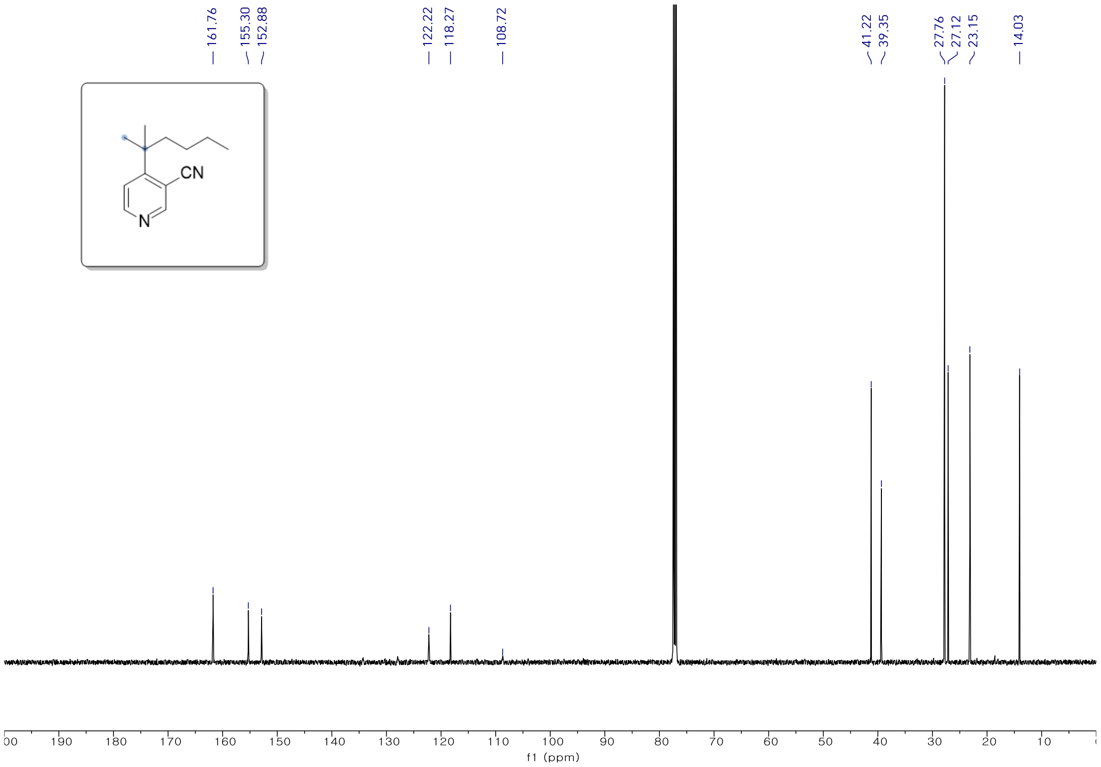

ethyl 4-(3-fluoropyridin-4-yl)-4-methylpentanoate (7i).

500 MHz, ^{1}H NMR in $CDCl_3$

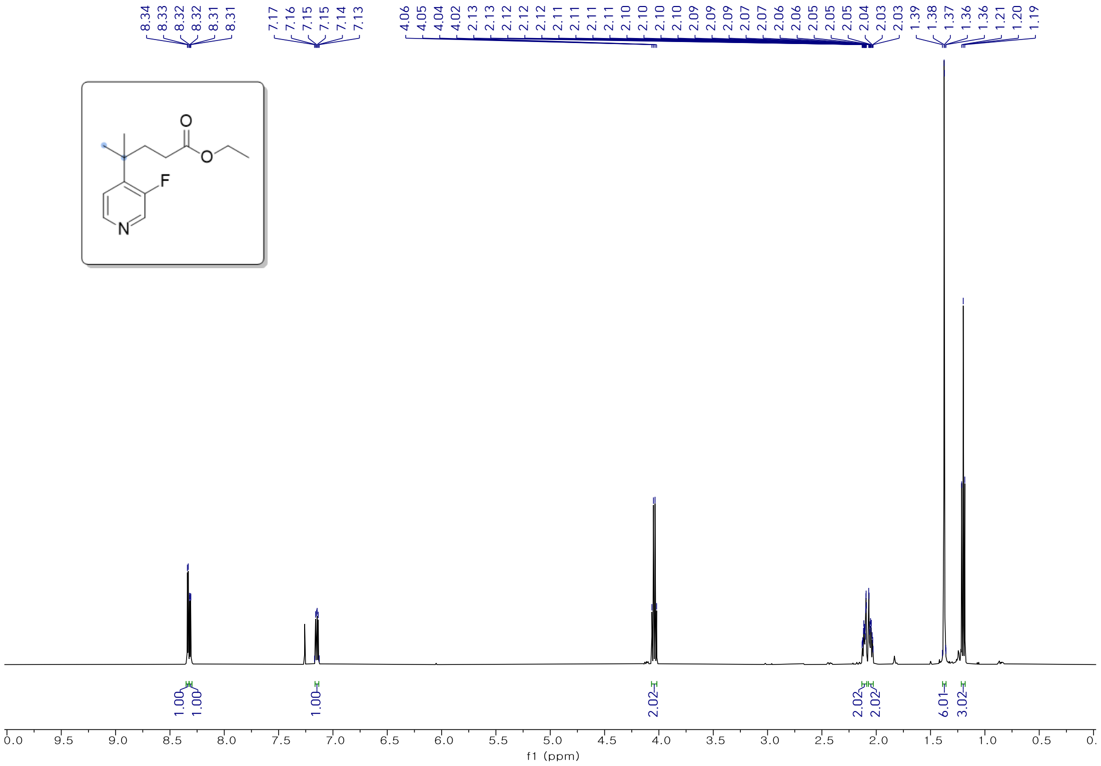

125 MHz, ^{13}C NMR in $CDCl_3$

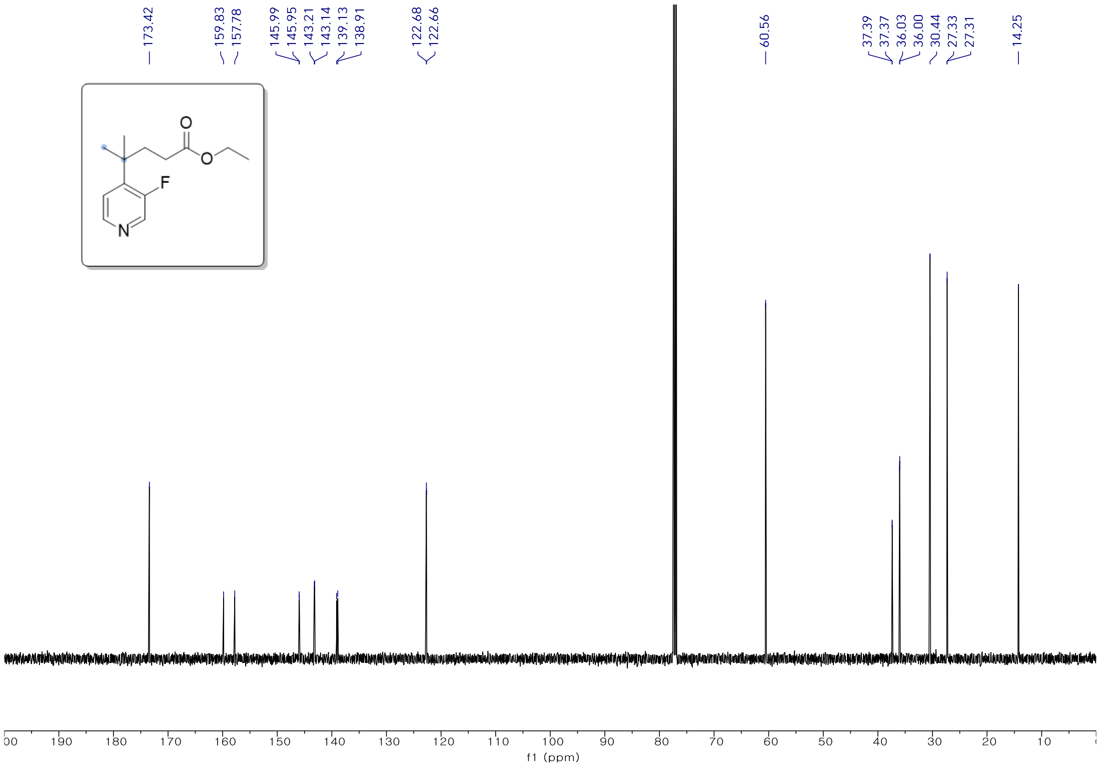

471 MHz, ^{19}F NMR in $CDCl_3$

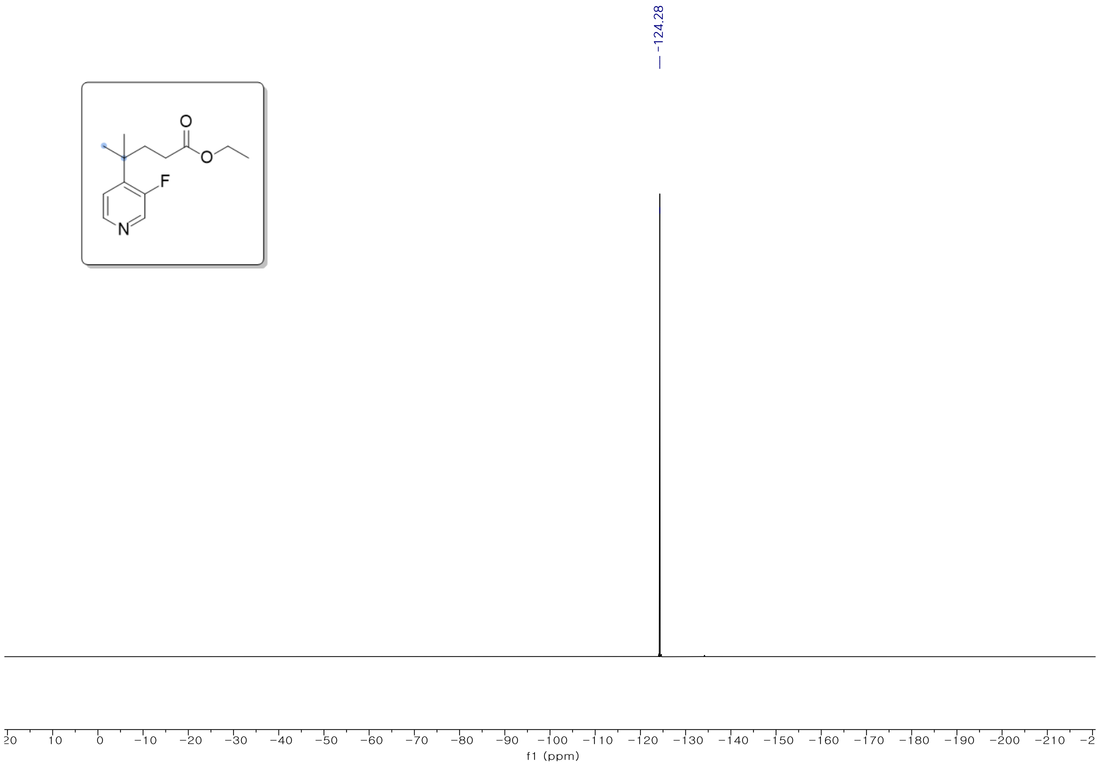

ethyl 4-(2,6-dimethylpyridin-4-yl)-4-methylpentanoate (7j).

500 MHz, ^{1}H NMR in $CDCl_3$

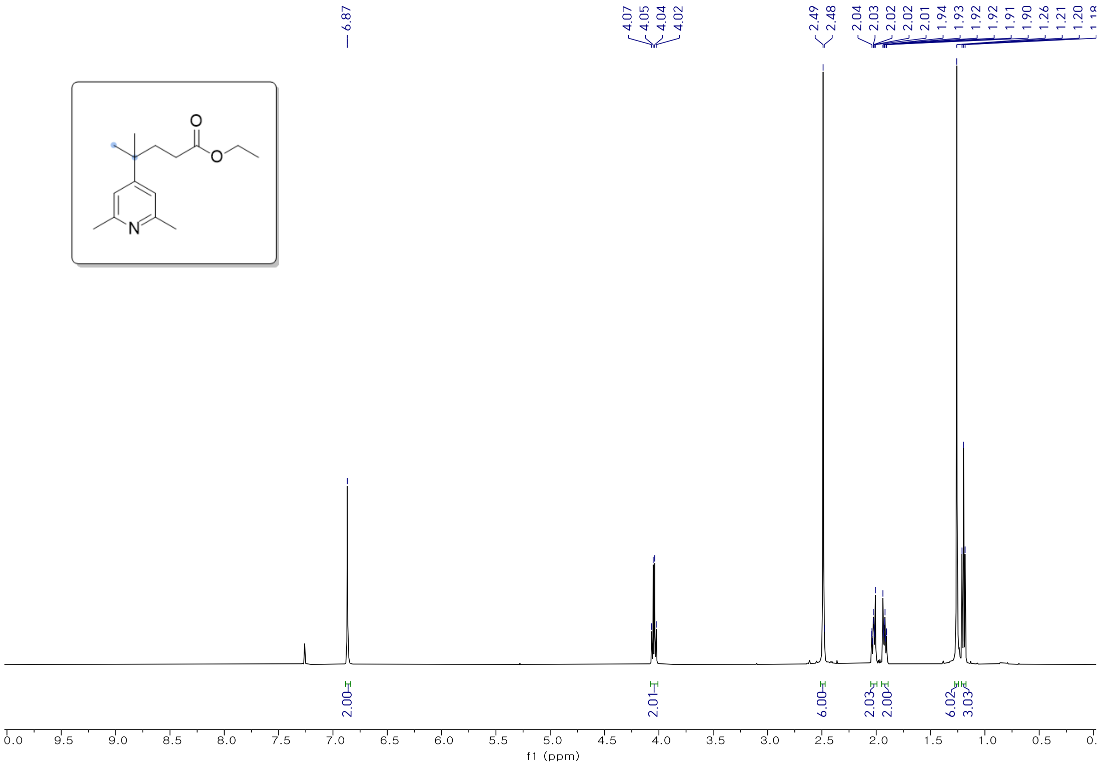

125 MHz, ^{13}C NMR in $CDCl_3$

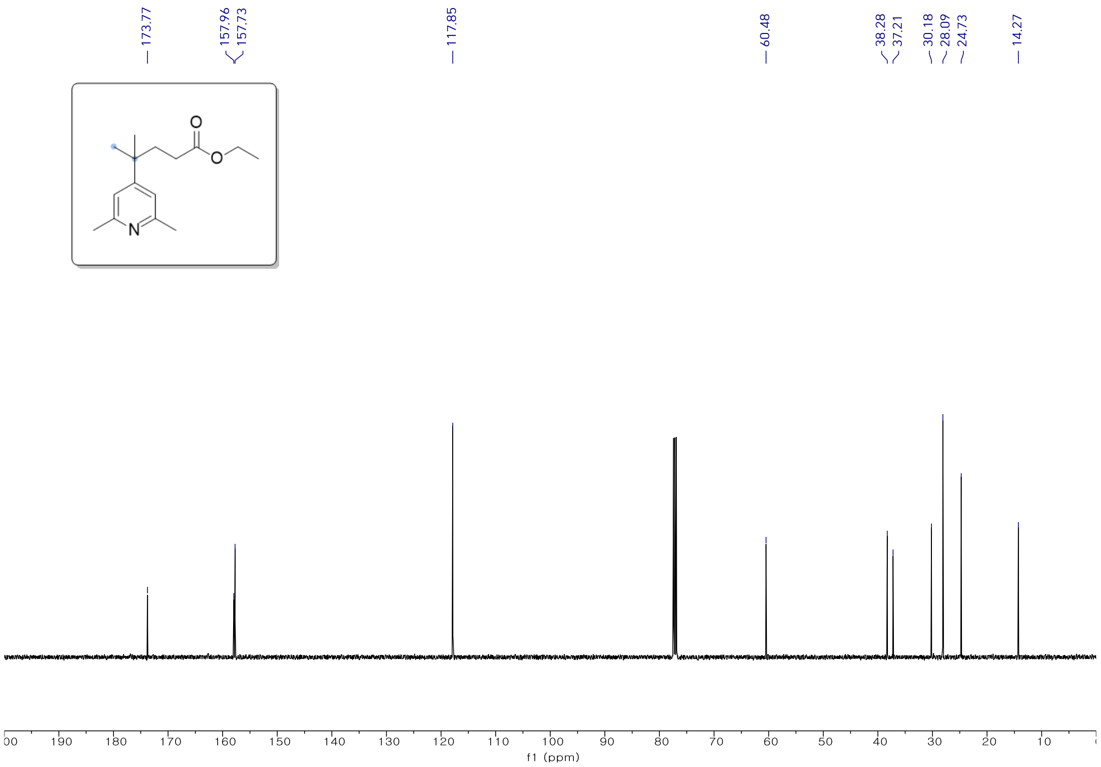

4-(2-methylhexan-2-yl)-6,7-dihydro-5H-cyclopenta[b]pyridine (7k).

500 MHz, ^{1}H NMR in $CDCl_3$

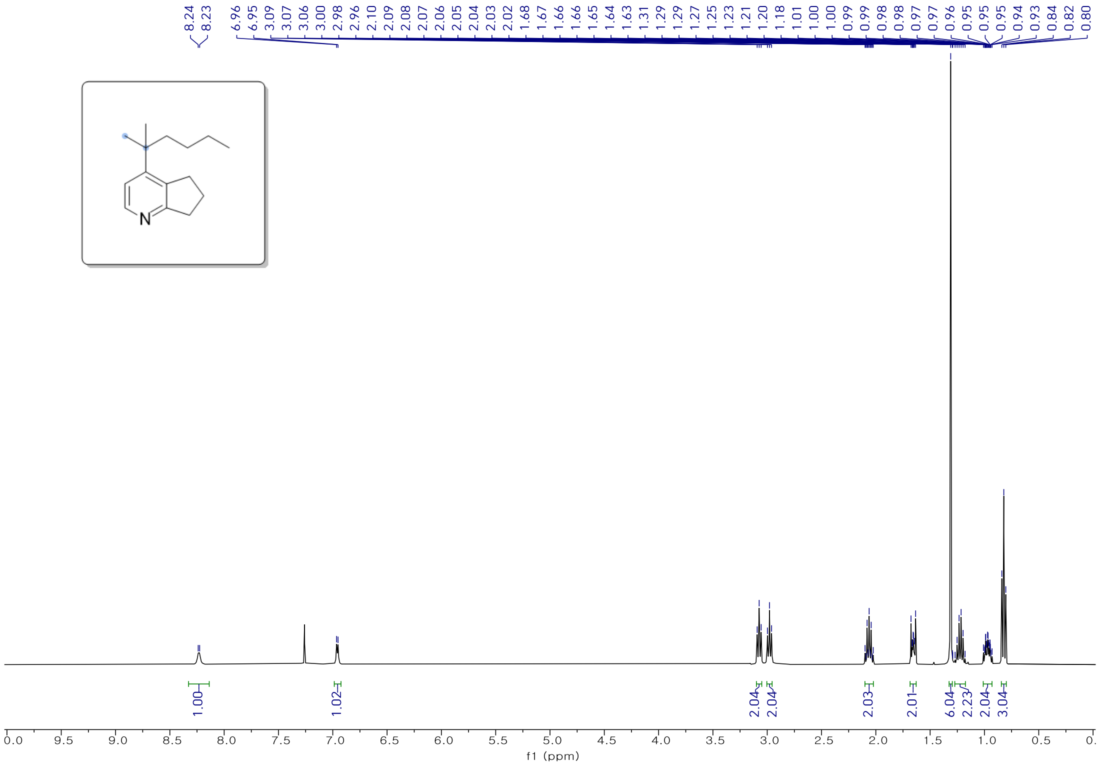

125 MHz, ^{13}C NMR in $CDCl_3$

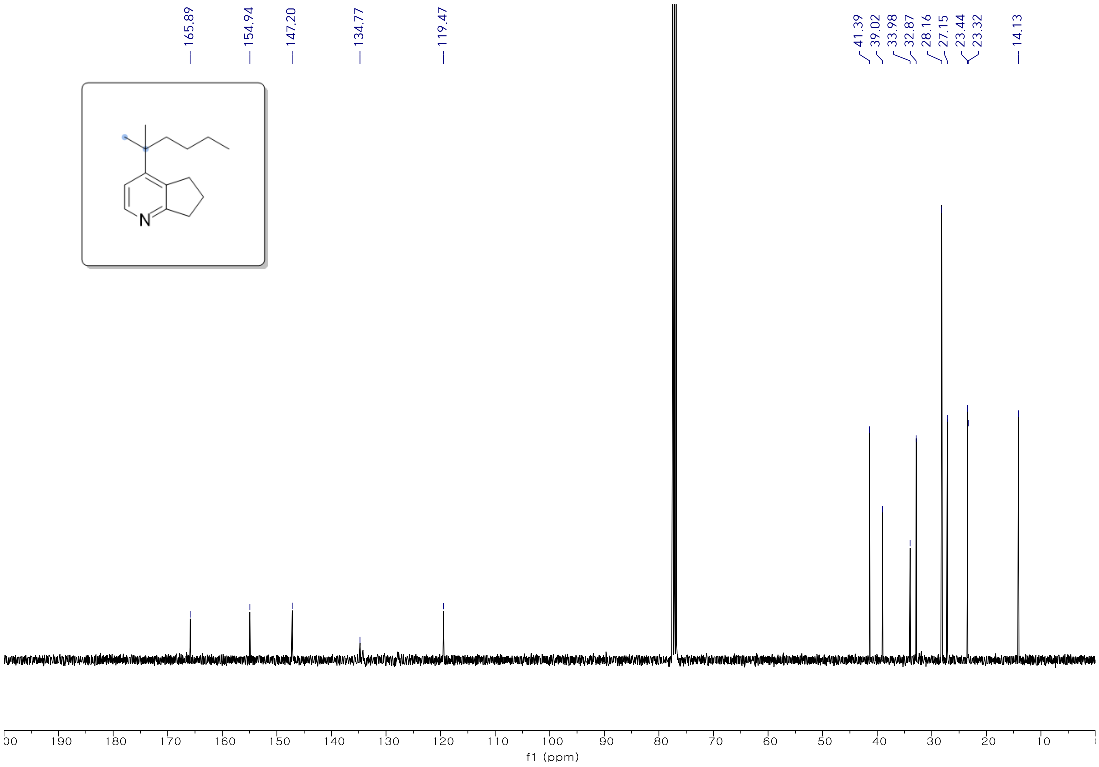

ethyl 4-methyl-4-(pyridin-4-yl)pentanoate (7l).

500 MHz, ^{1}H NMR in $CDCl_3$

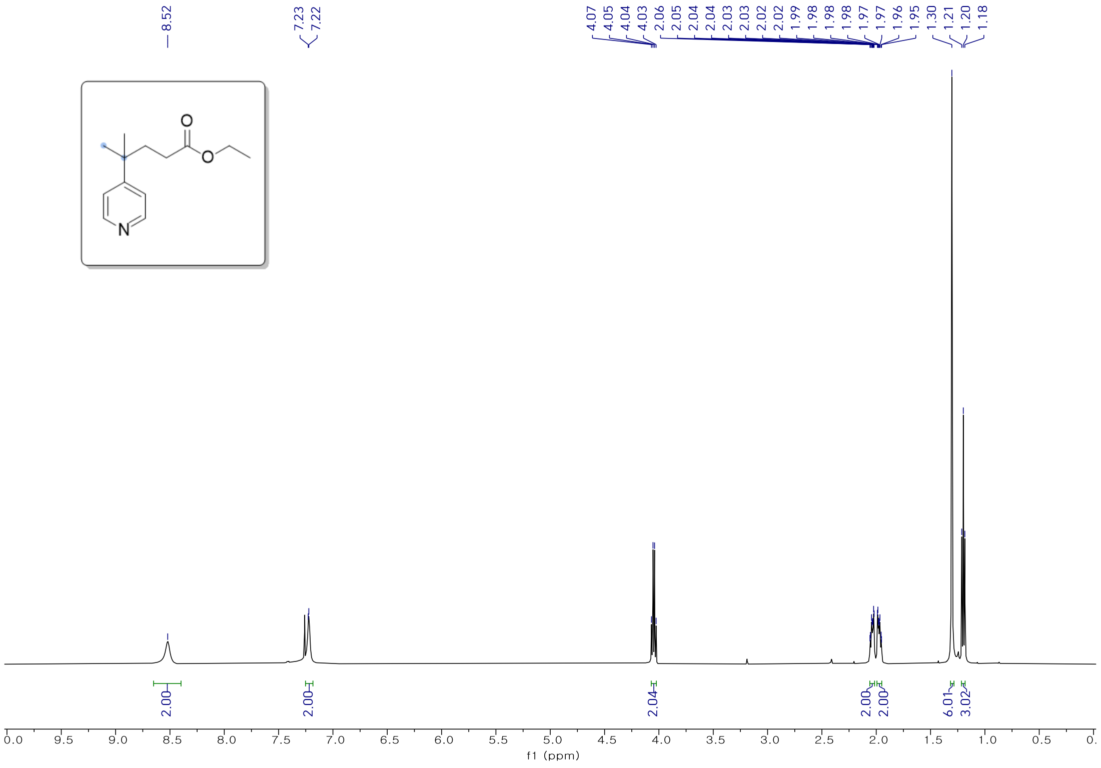

125 MHz, ^{13}C NMR in $CDCl_3$

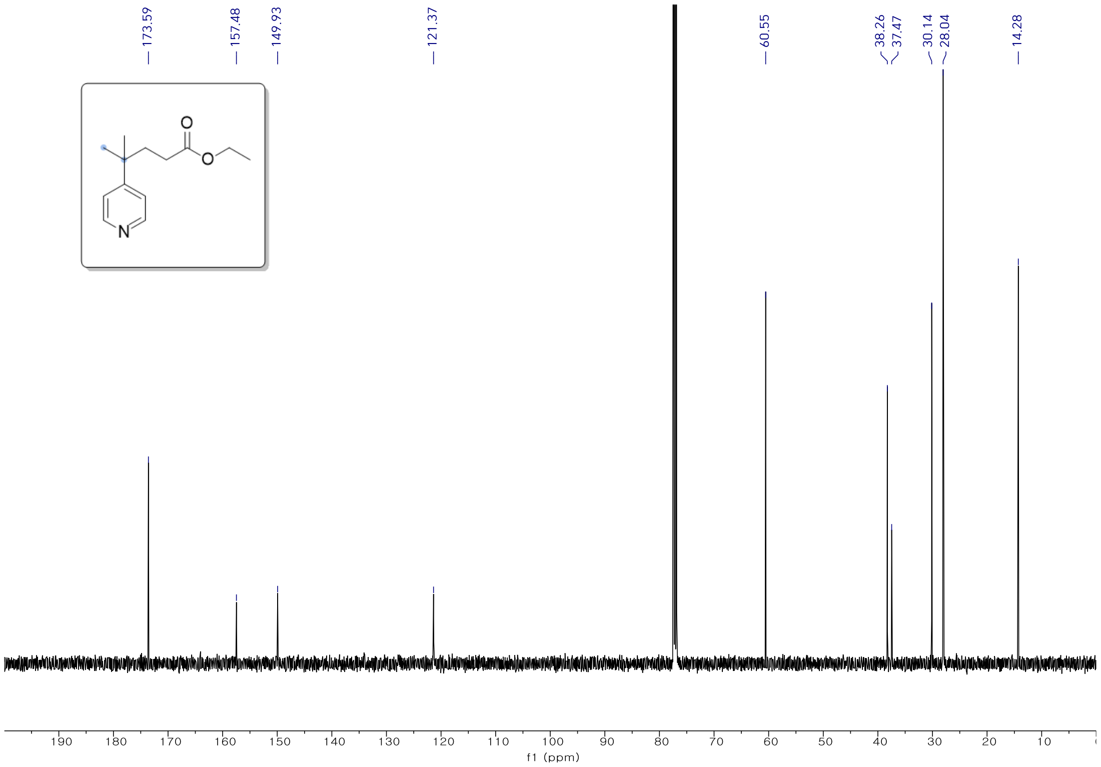

4-(2-phenylpyridin-4-yl)pentyl 2-(4-(4-chlorobenzoyl)phenoxy)-2-methylpropanoate (8a).

500 MHz, ^{1}H NMR in $CDCl_3$

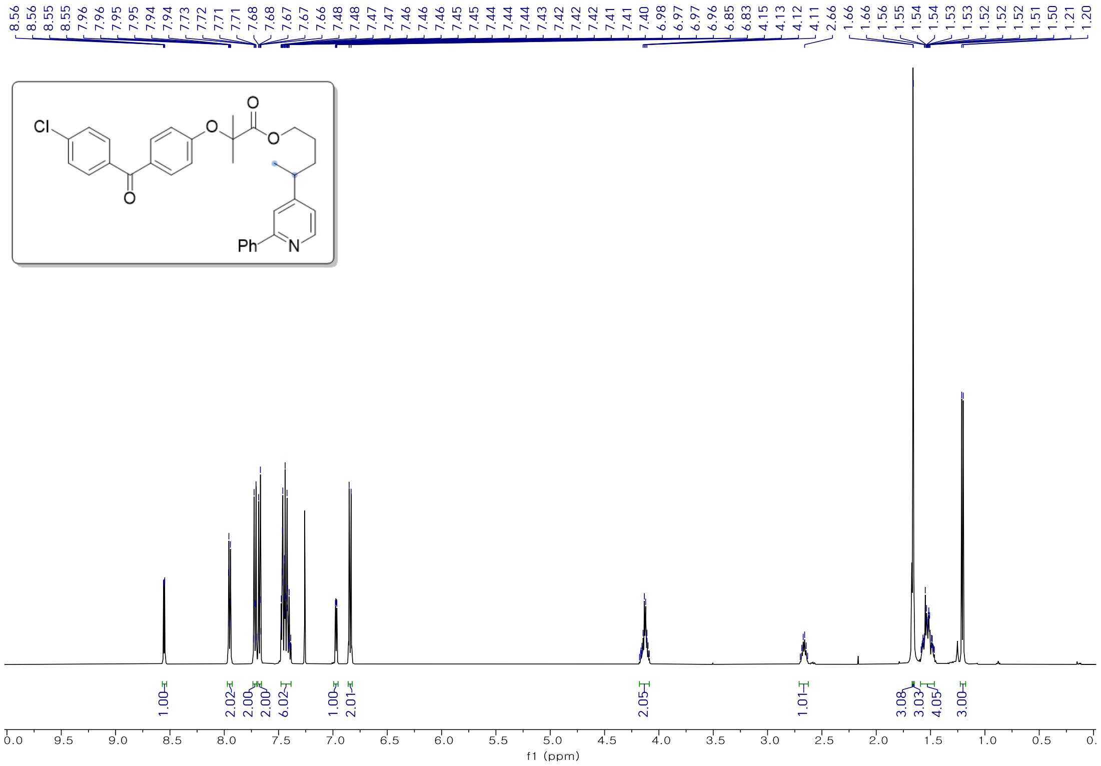

100 MHz, ^{13}C NMR in $CDCl_3$

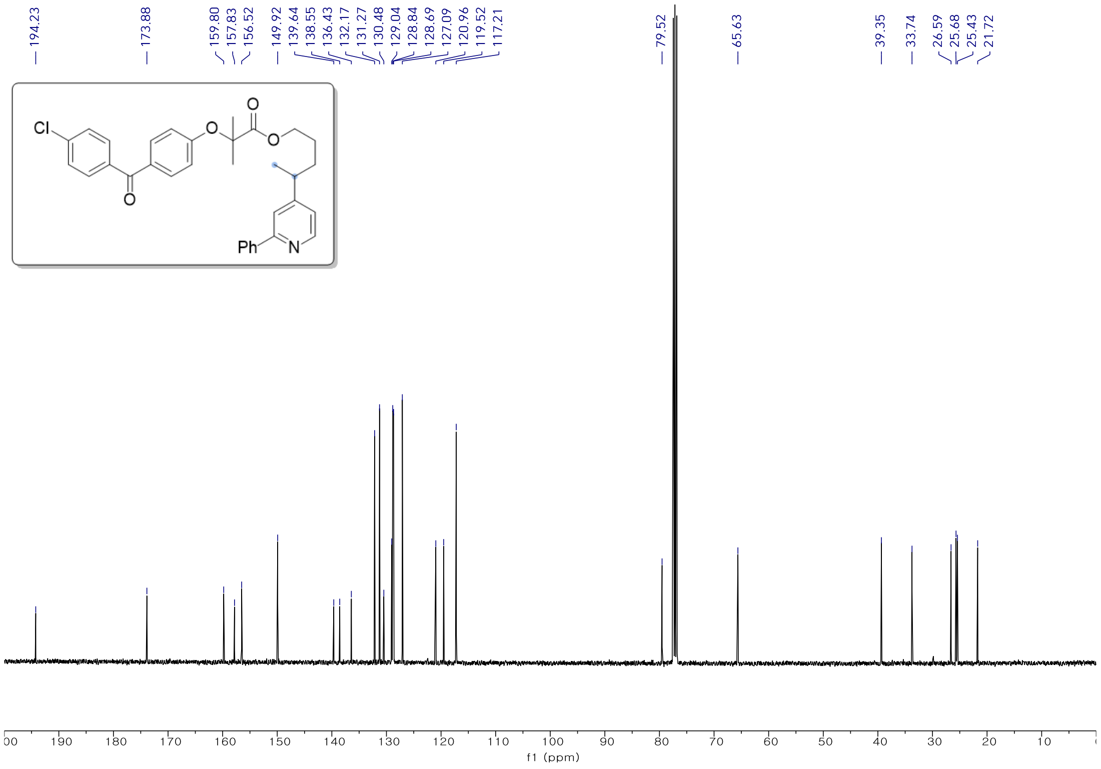

3-methyl-3-(2-phenylpyridin-4-yl)butyl 2-(10-oxo-10,11-dihydrodibenzo[b,f]thiepin-2-yl)propanoate (8b).

400 MHz, ^{1}H NMR in CDCl$_3$

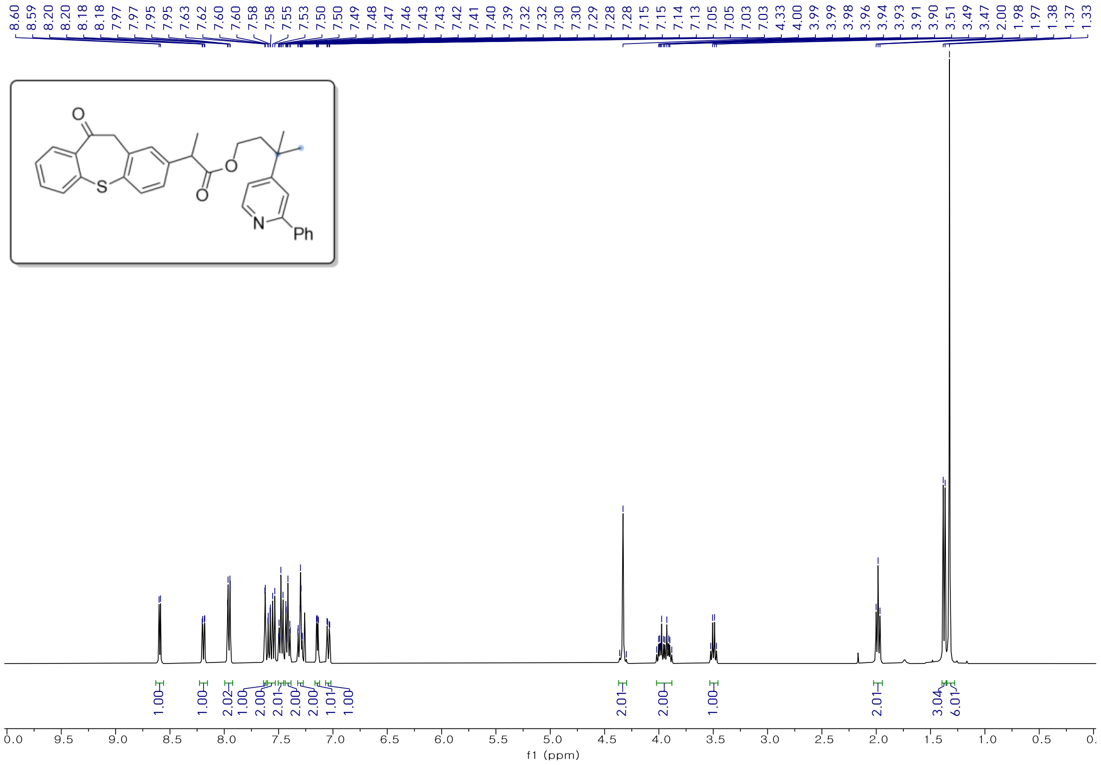

100 MHz, ^{13}C NMR in CDCl$_3$

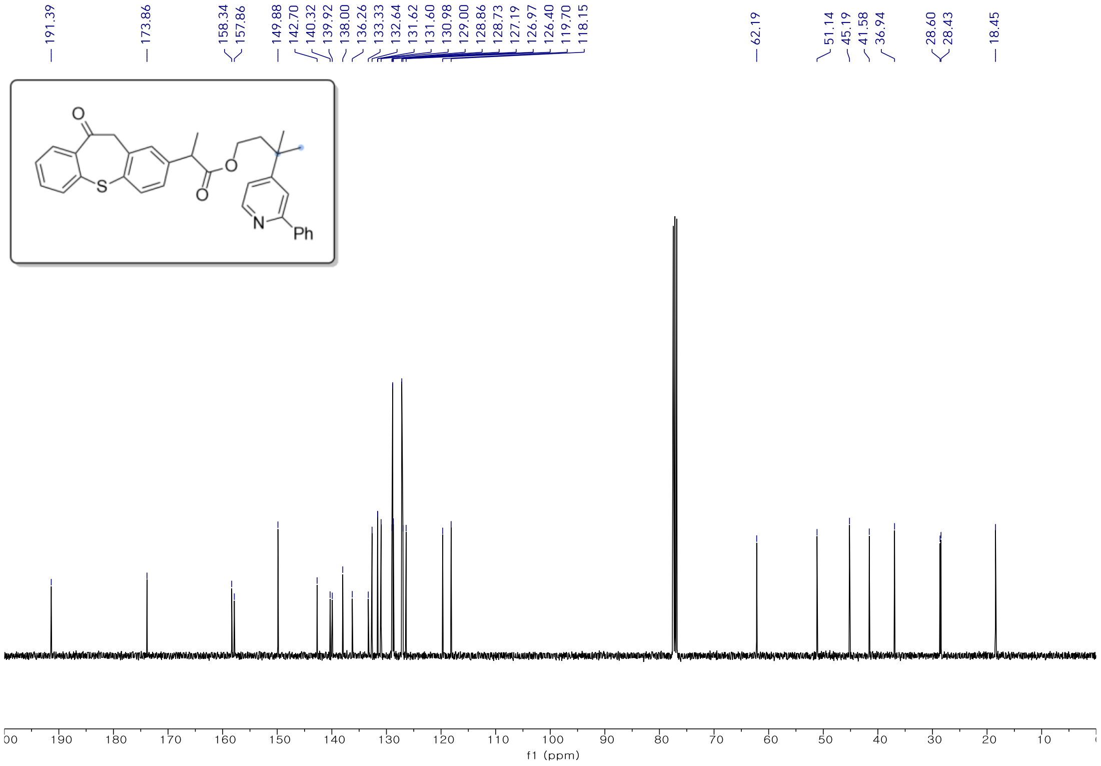

ethyl (S)-2-((tert-butoxycarbonyl)amino)-3-(4-(3-methyl-3-(2-phenylpyridin-4-yl)butoxy)phenyl)propanoate (8c).

500 MHz, ^{1}H NMR in $CDCl_3$

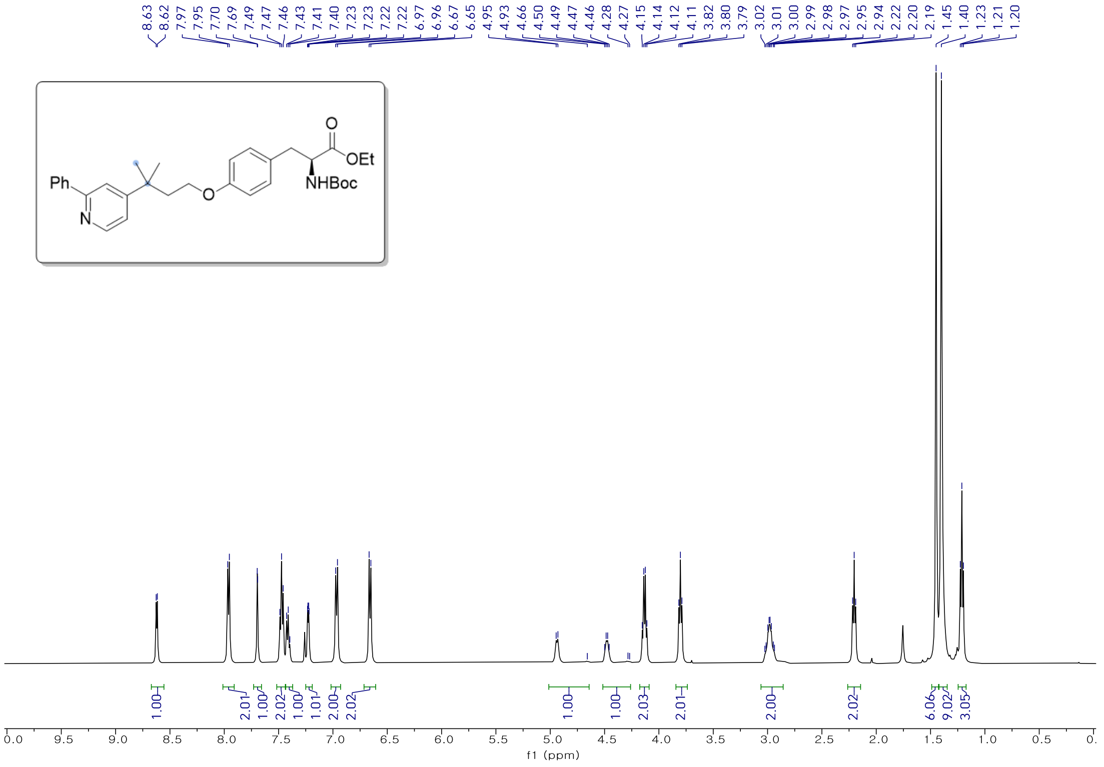

125 MHz, ^{13}C NMR in $CDCl_3$

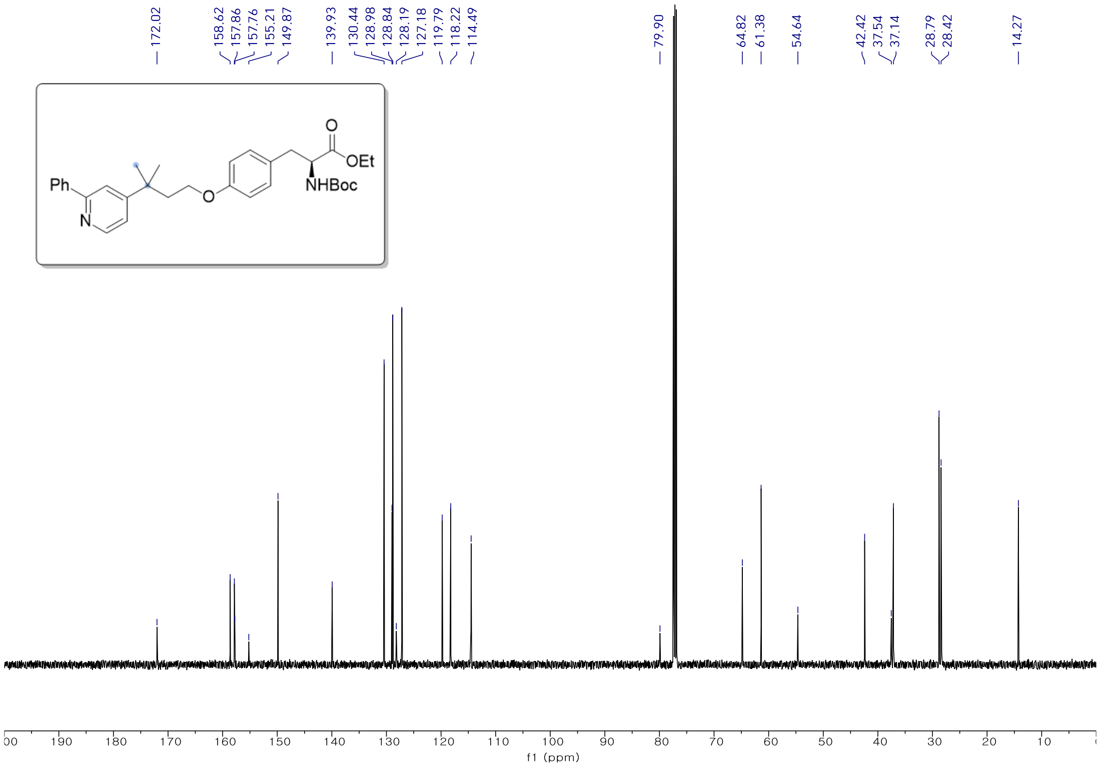

2-methoxy-4-(2-(2-phenylpyridin-4-yl)propyl)phenol (8d).

600 MHz, ^{1}H NMR in CDCl$_3$

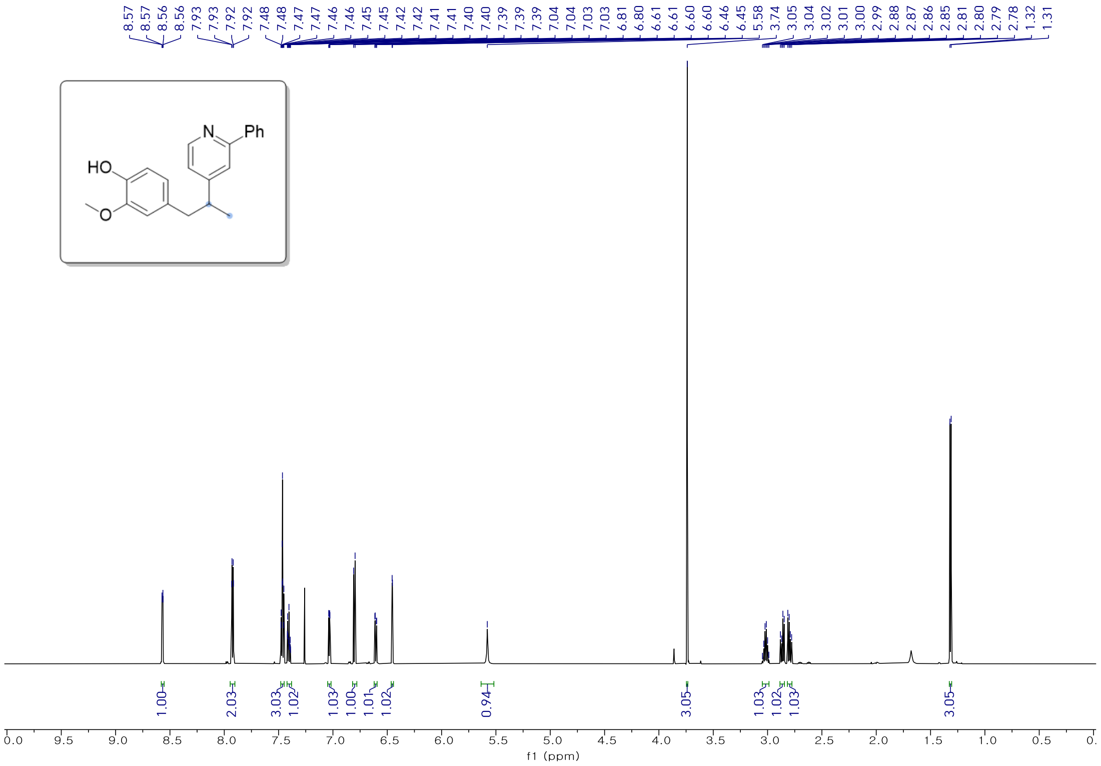

100 MHz, ^{13}C NMR in CDCl$_3$

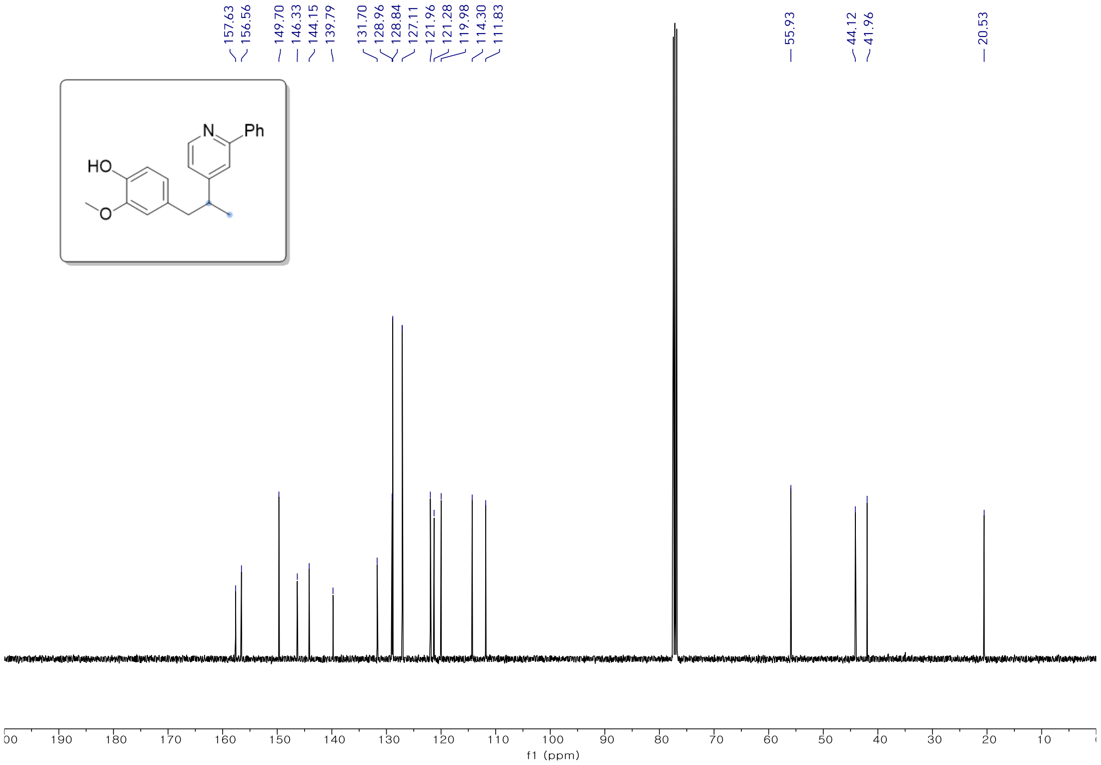

4-((2S,4R,5S,6R)-4,5-bis(benzyloxy)-6-((benzyloxy)methyl)tetrahydro-2H-pyran-2-yl)-2-phenylpyridine (8e).

400 MHz, ^{1}H NMR in $CDCl_3$

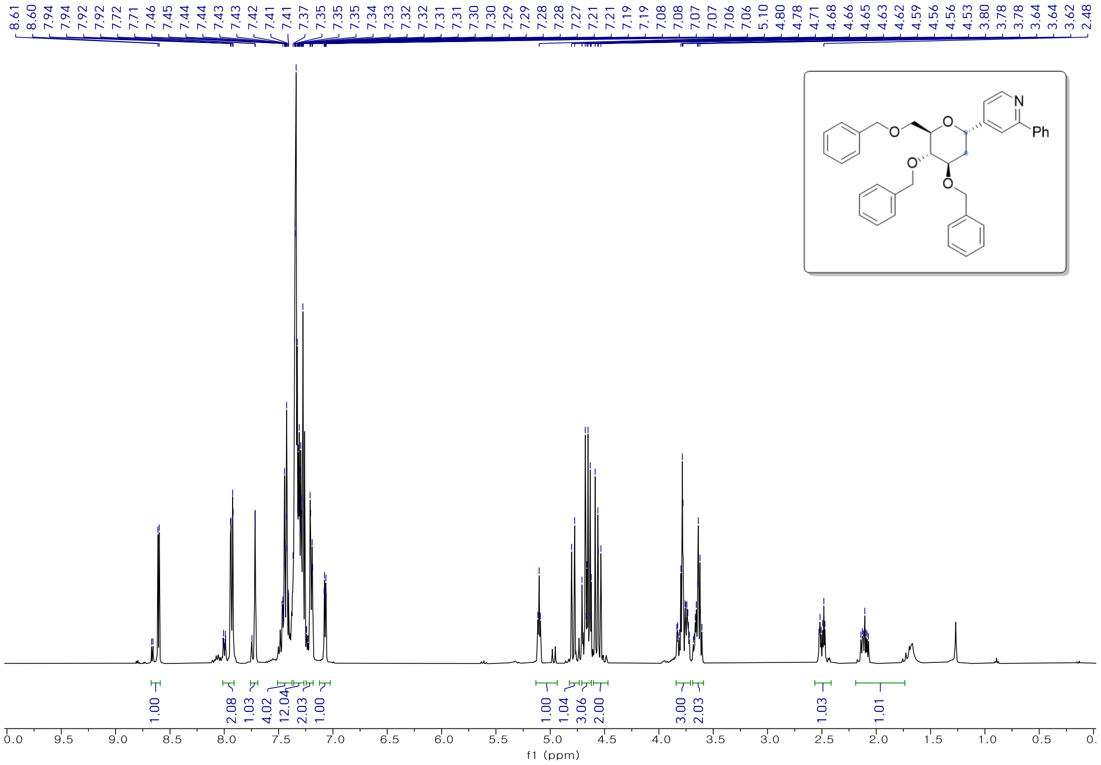

100 MHz, ^{13}C NMR in $CDCl_3$

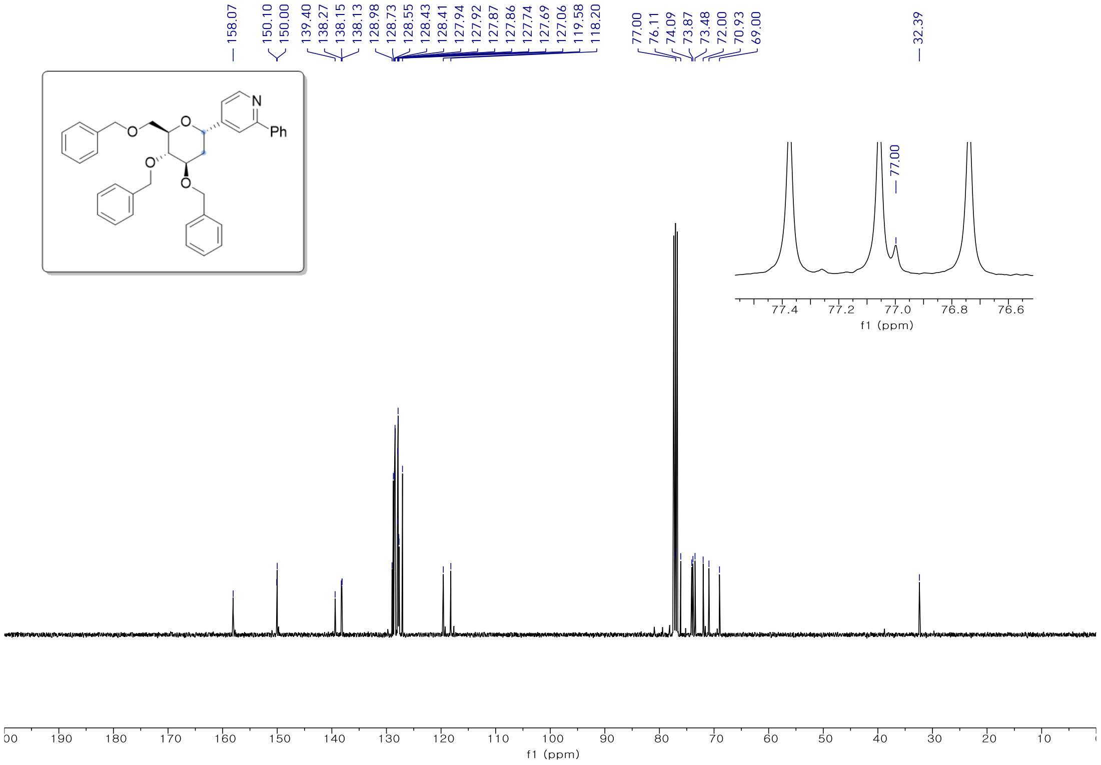

(8R,9S,13S,14S,17S)-13-methyl-17-((4-(2-phenylpyridin-4-yl)pentyl)oxy)-7,8,9,11,12,13,14,15,-16,17-decahydro-6H-cyclopenta[a]phenanthren-3-ol (8f).

500 MHz, ^{1}H NMR in $CDCl_3$

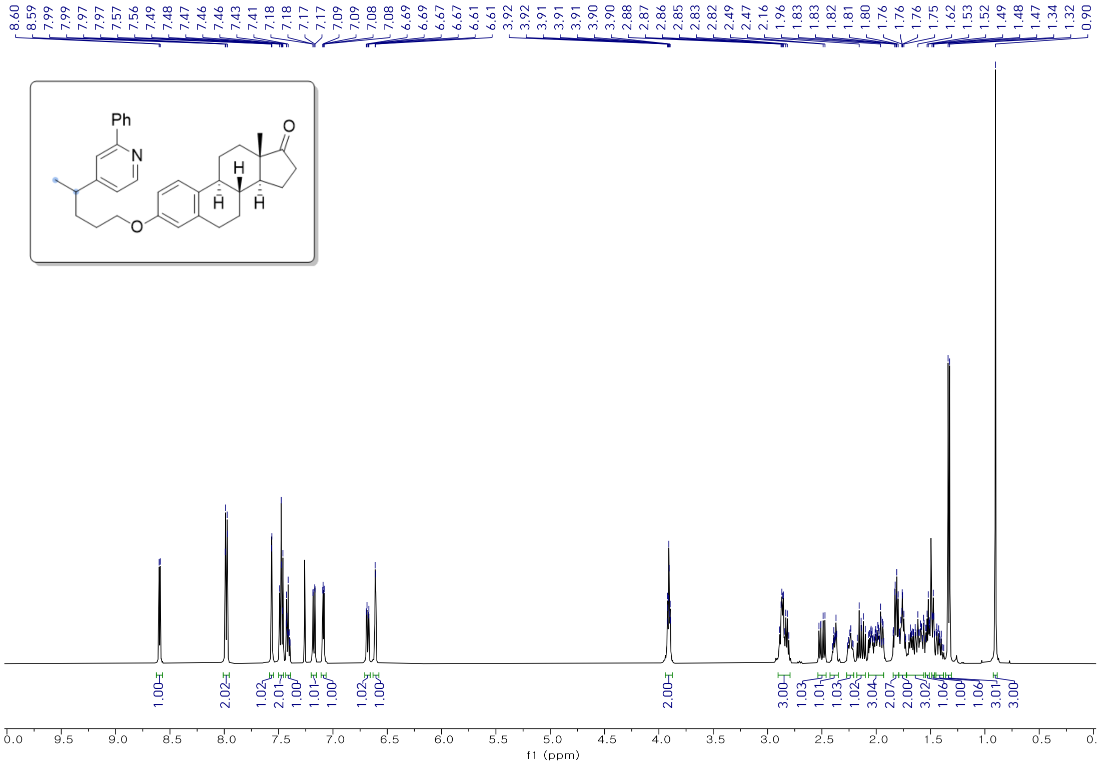

150 MHz, ^{13}C NMR in $CDCl_3$

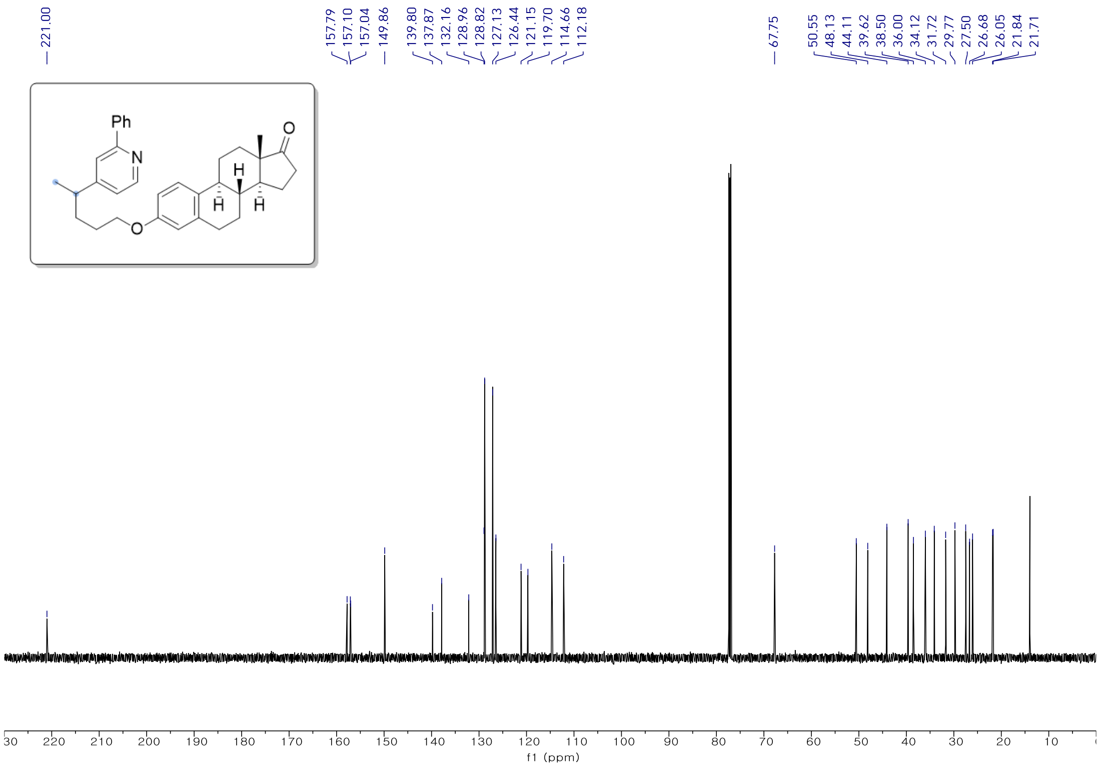

(8R,9S,10R,13S,14S,17S)-10,13-dimethyl-3-oxo-6,7,8,9,10,11,12,13,14,15,16,17-dodecahydro-3H-cyclopenta[a]phenanthren-17-yl 10-(2-phenylpyridin-4-yl)undecanoate (8g).

500 MHz, ^{1}H NMR in CDCl$_3$

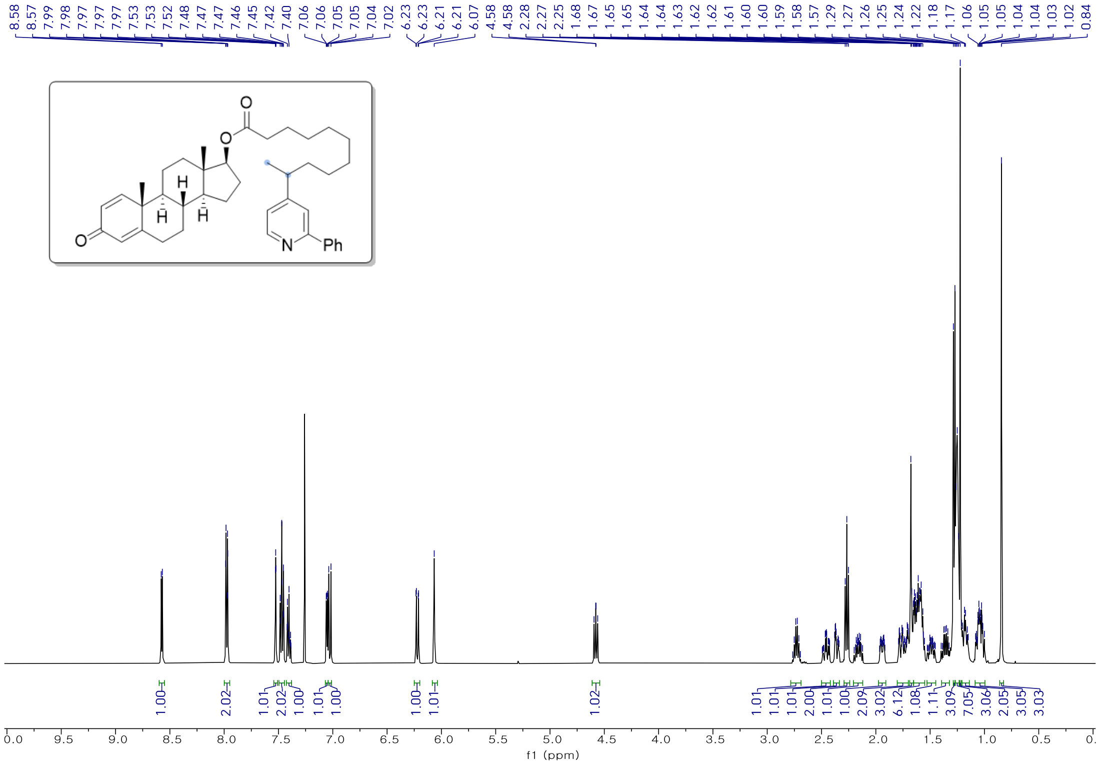

100 MHz, ^{13}C NMR in CDCl$_3$

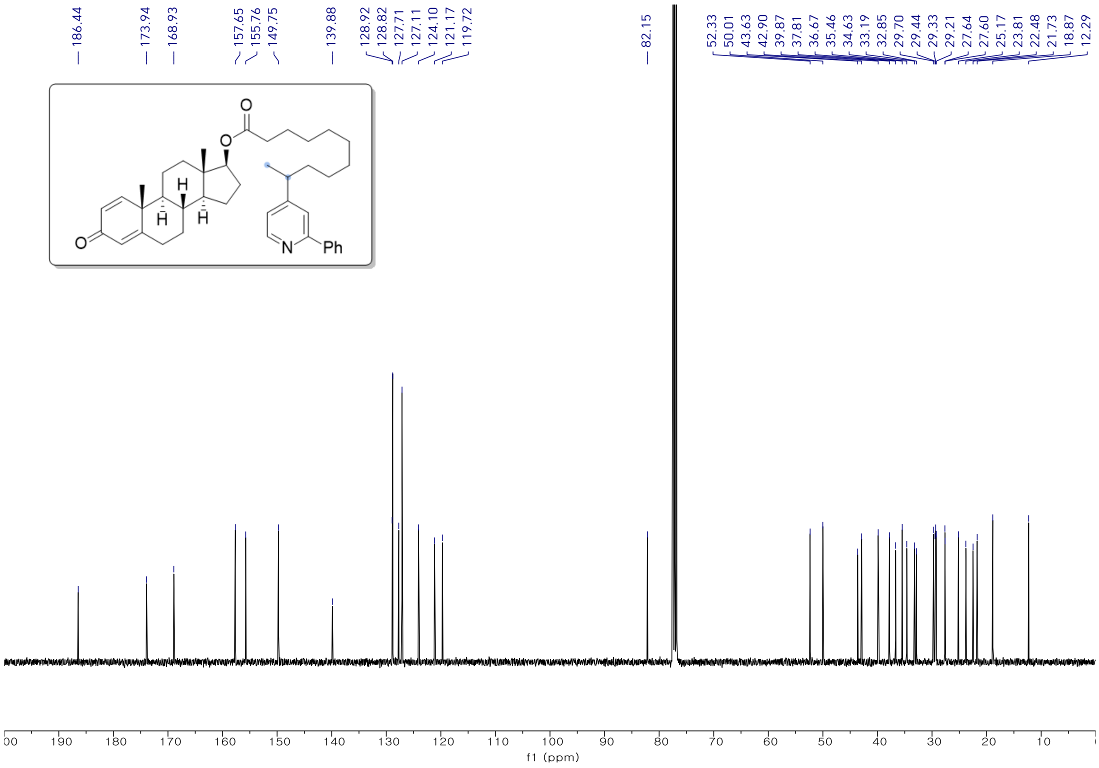

3,7-dimethyl-7-(2-phenylpyridin-4-yl)octan-1-ol (8h).

500 MHz, ^{1}H NMR in $CDCl_3$

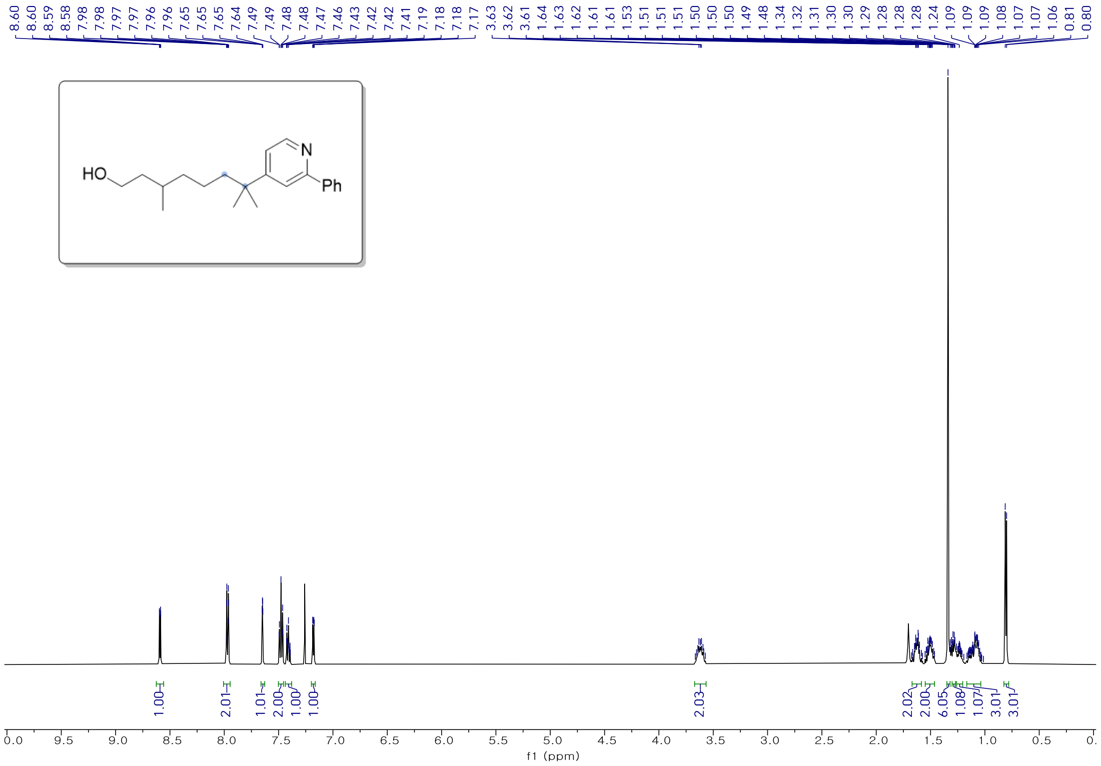

100 MHz, ^{13}C NMR in $CDCl_3$

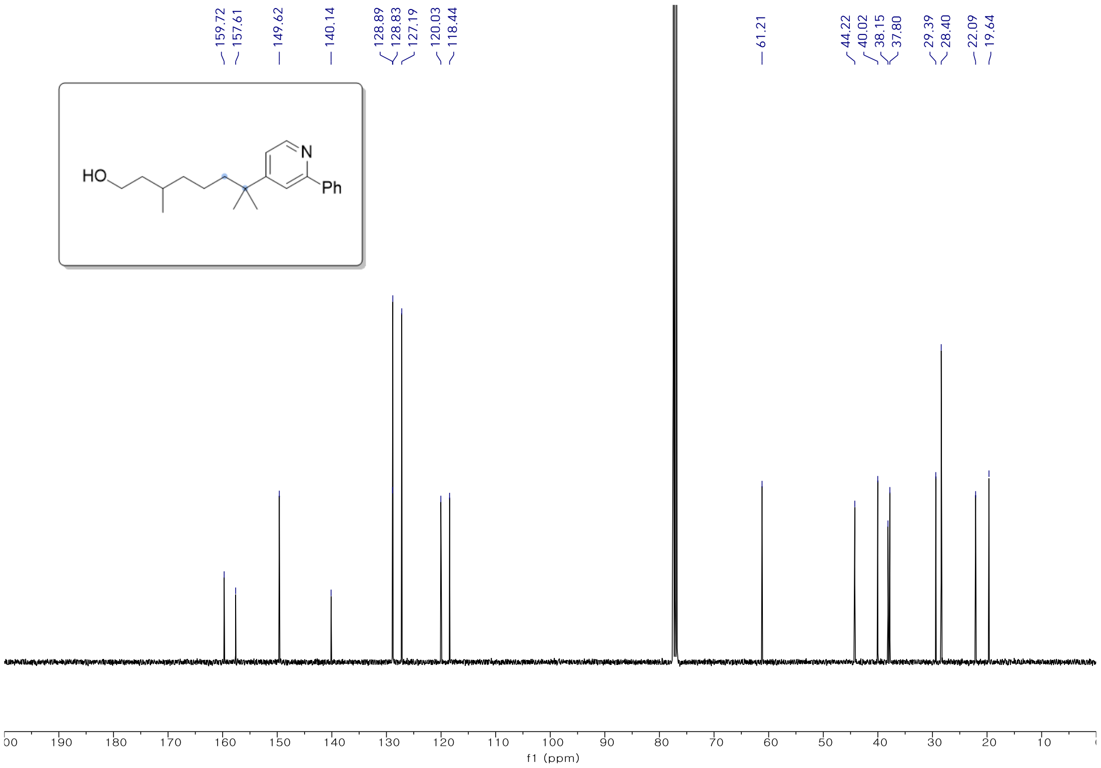

((4-(2-methylhexan-2-yl)pyridin-2-yl)methylene)bis(4,1-phenylene) diacetate (8i).

500 MHz, ^{1}H NMR in $CDCl_3$

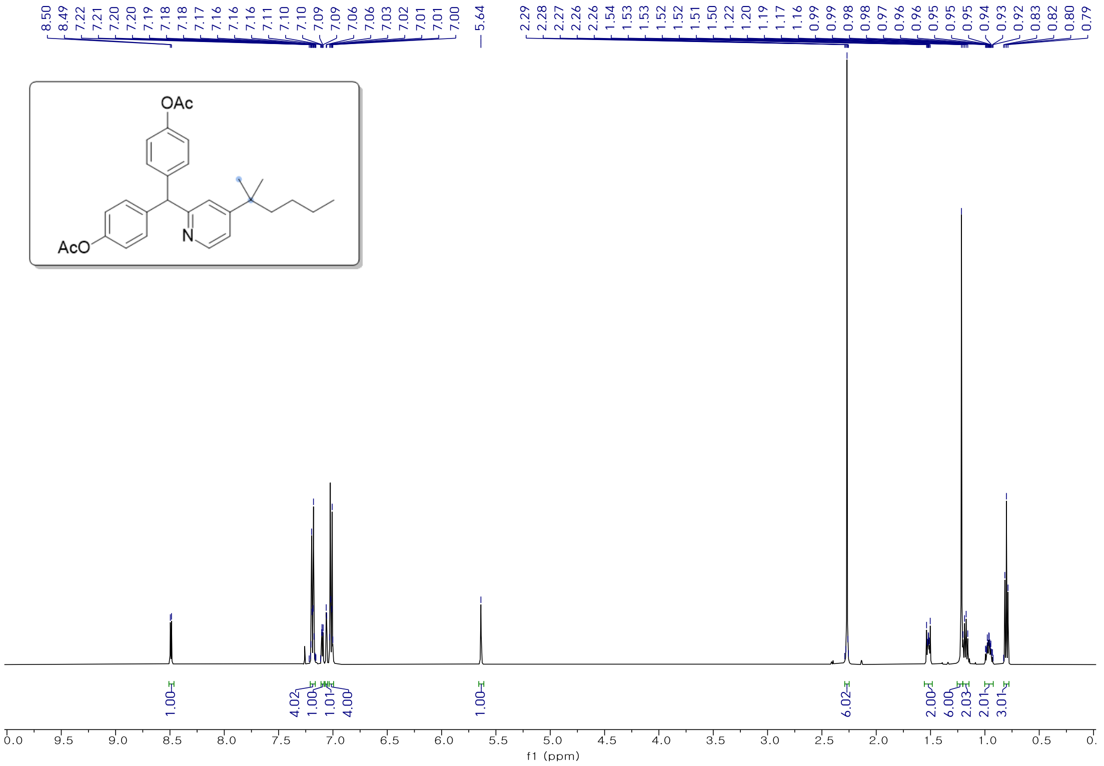

125 MHz, ^{13}C NMR in $CDCl_3$

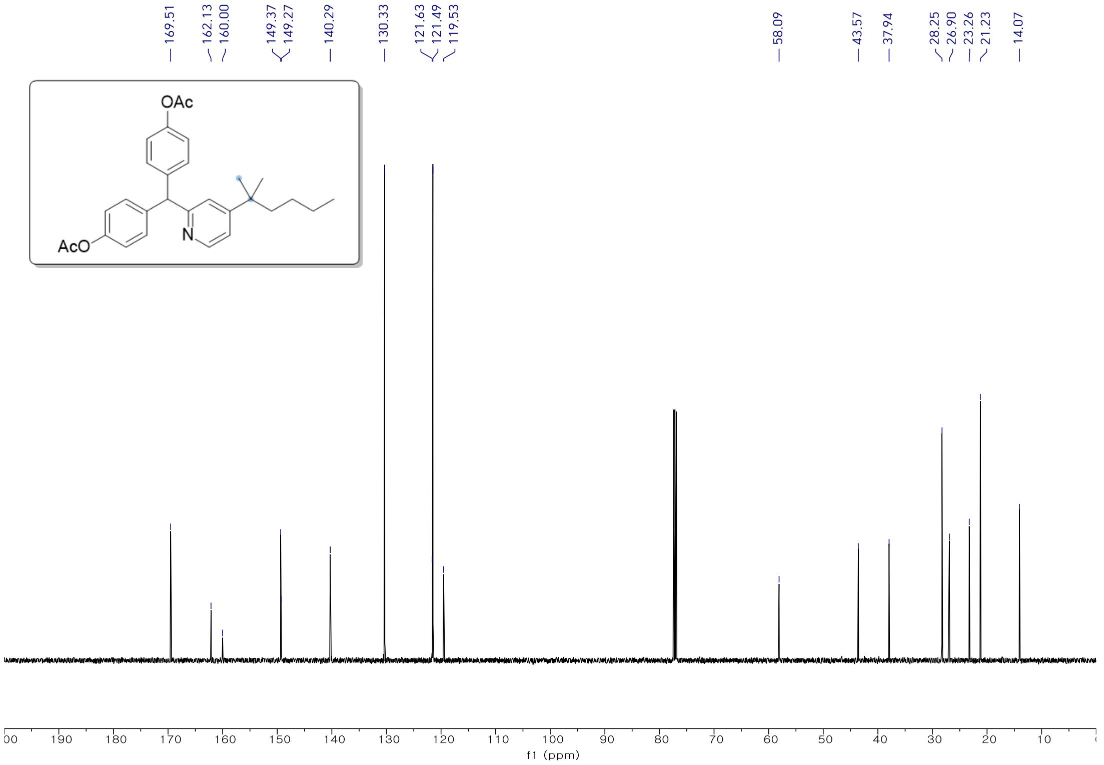

4-(2-methylhexan-2-yl)-2-((1-(4-phenoxyphenoxy)propan-2-yl)oxy)pyridine (8j).

500 MHz, ^{1}H NMR in $CDCl_3$

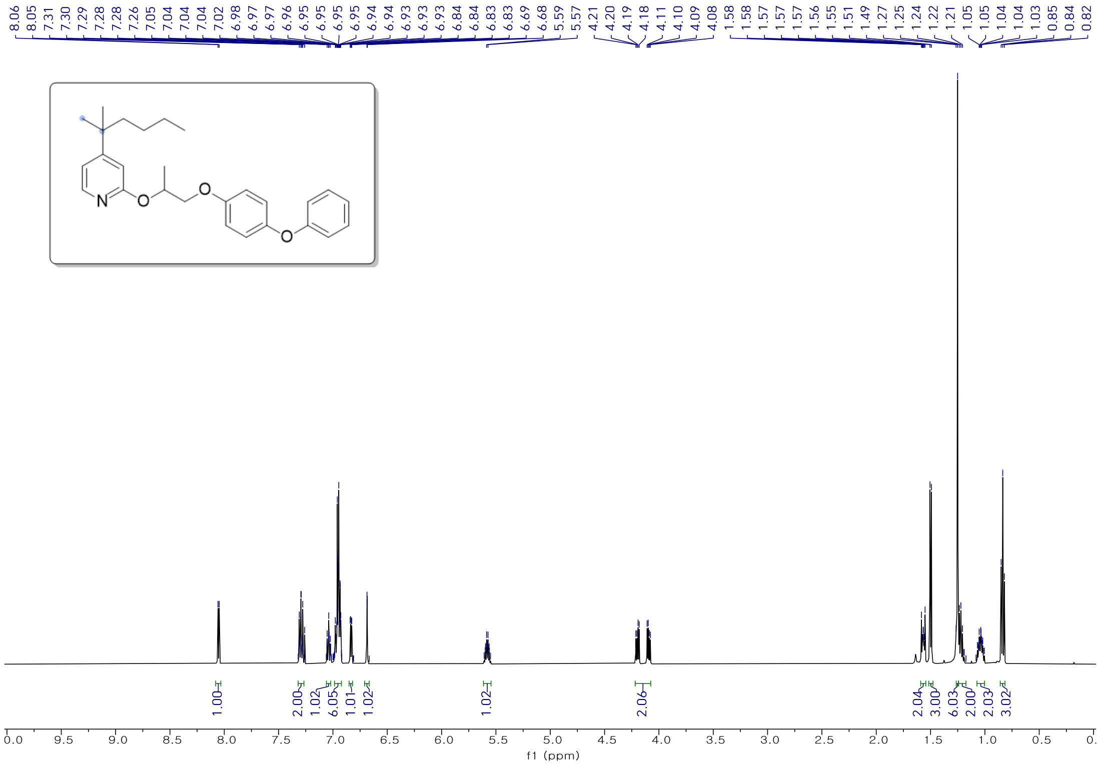

125 MHz, ^{13}C NMR in $CDCl_3$

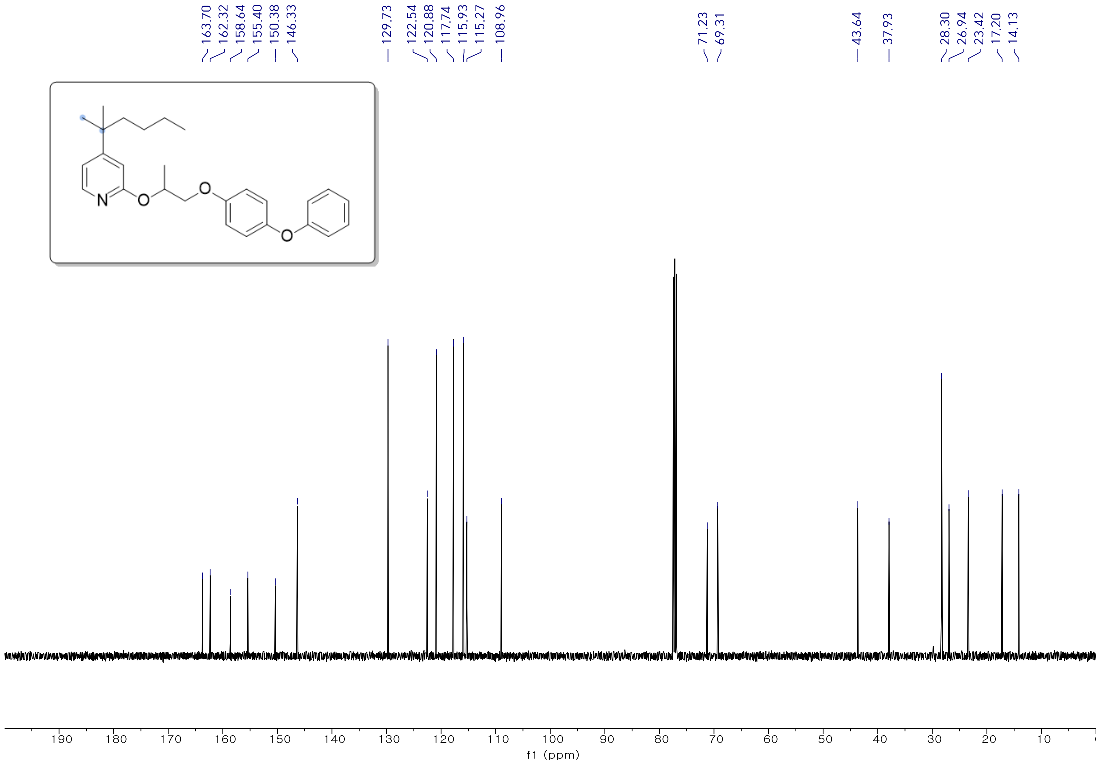

2-chloro-N-(4-chloro-3-(4-(2-methylhexan-2-yl)pyridin-2-yl)phenyl)-4-(methylsulfonyl)benzamide (8k).

400 MHz, ^{1}H NMR in $CDCl_3$

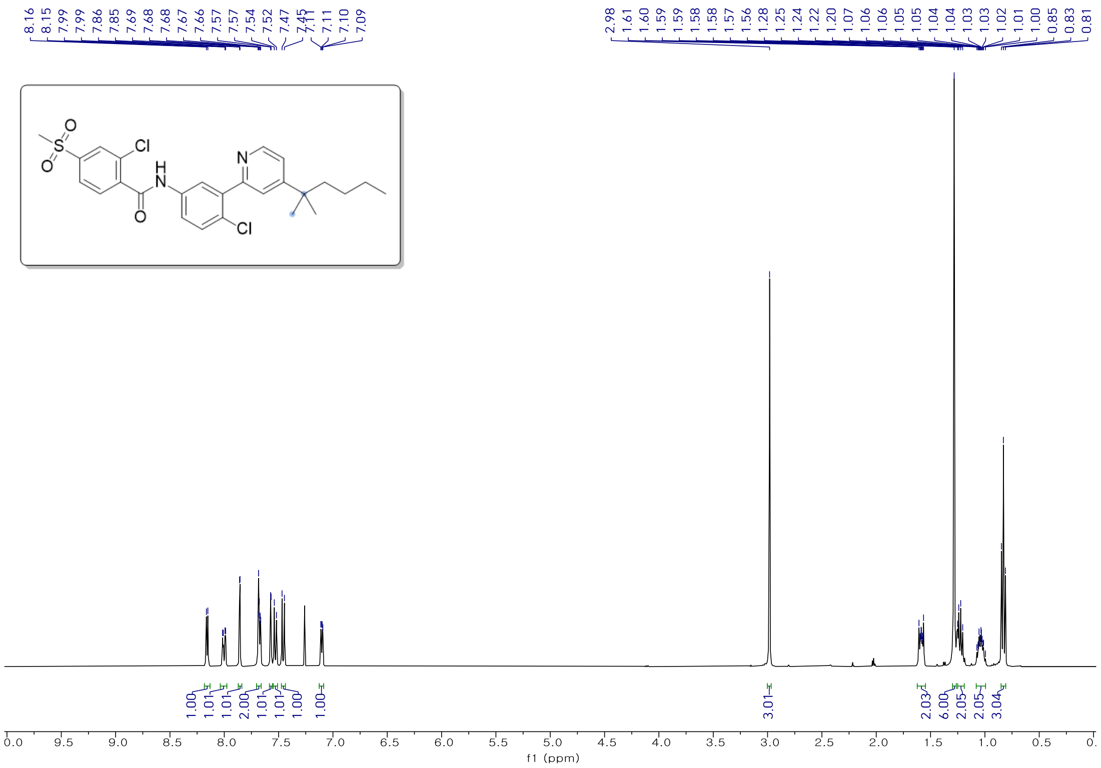

100 MHz, ^{13}C NMR in $CDCl_3$

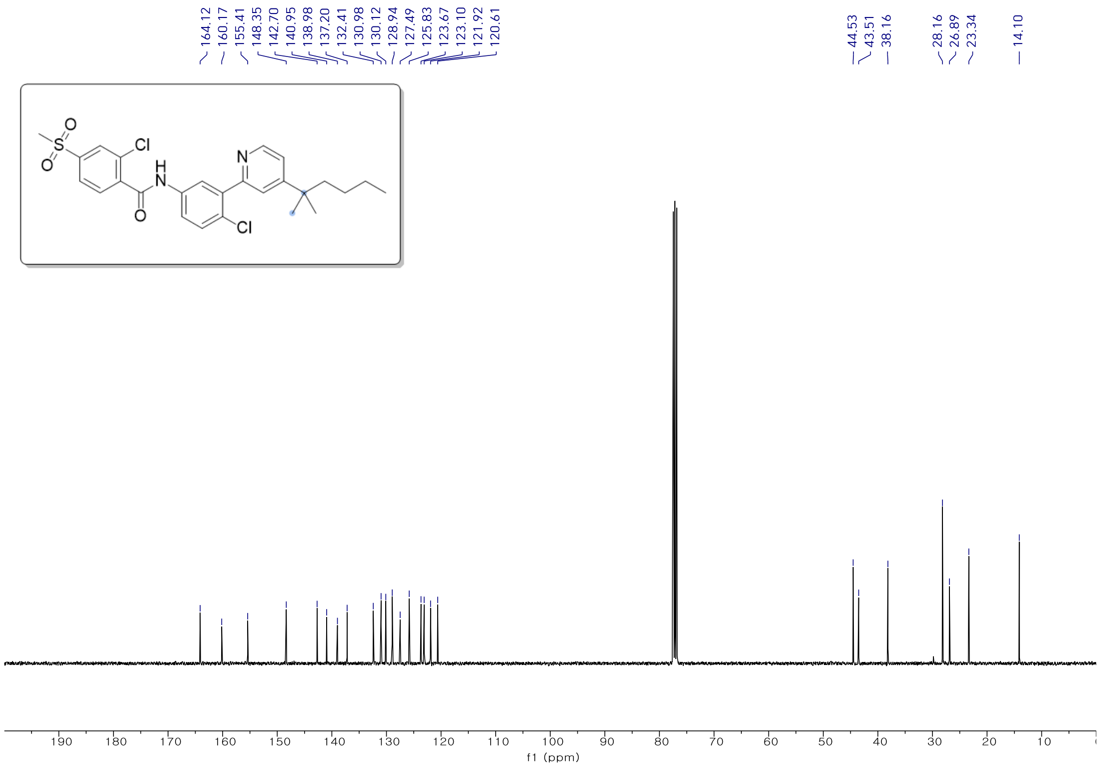

4-((1s,3s)-3-(methoxy-d3)-1-methylcyclobutyl-3-d)-2-phenylpyridine (3b-D_4).

500 MHz, ^{1}H NMR in $CDCl_3$

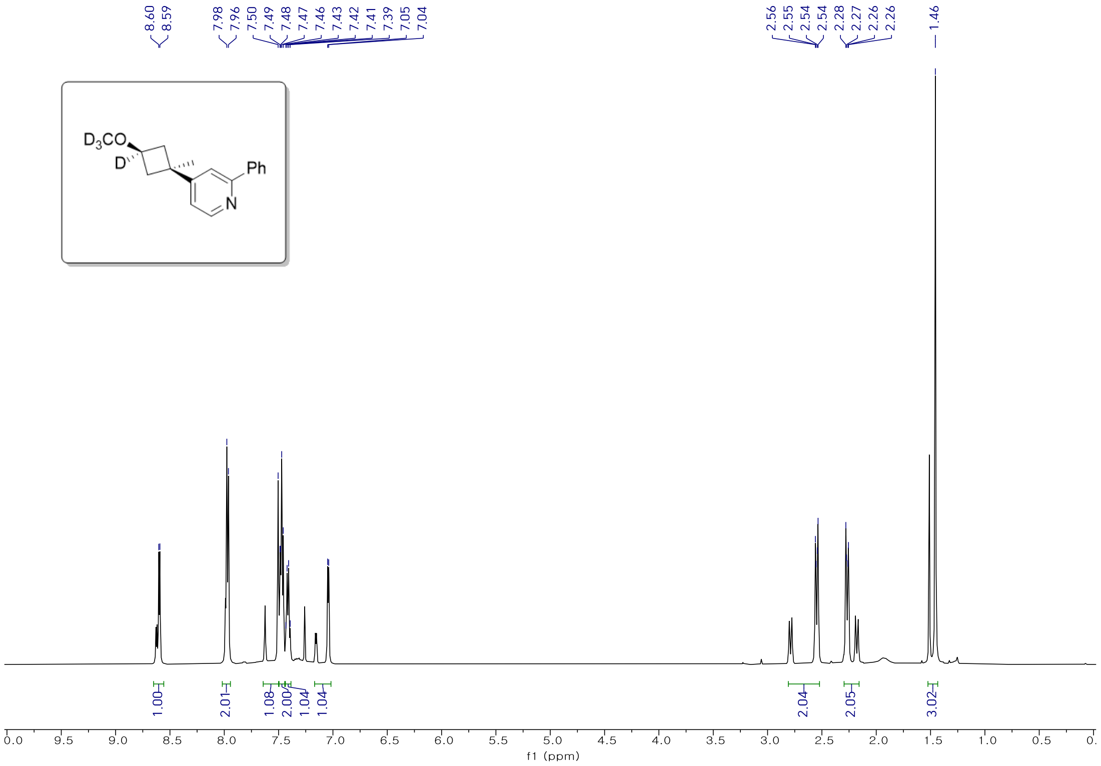

100 MHz, ^{13}C NMR in $CDCl_3$

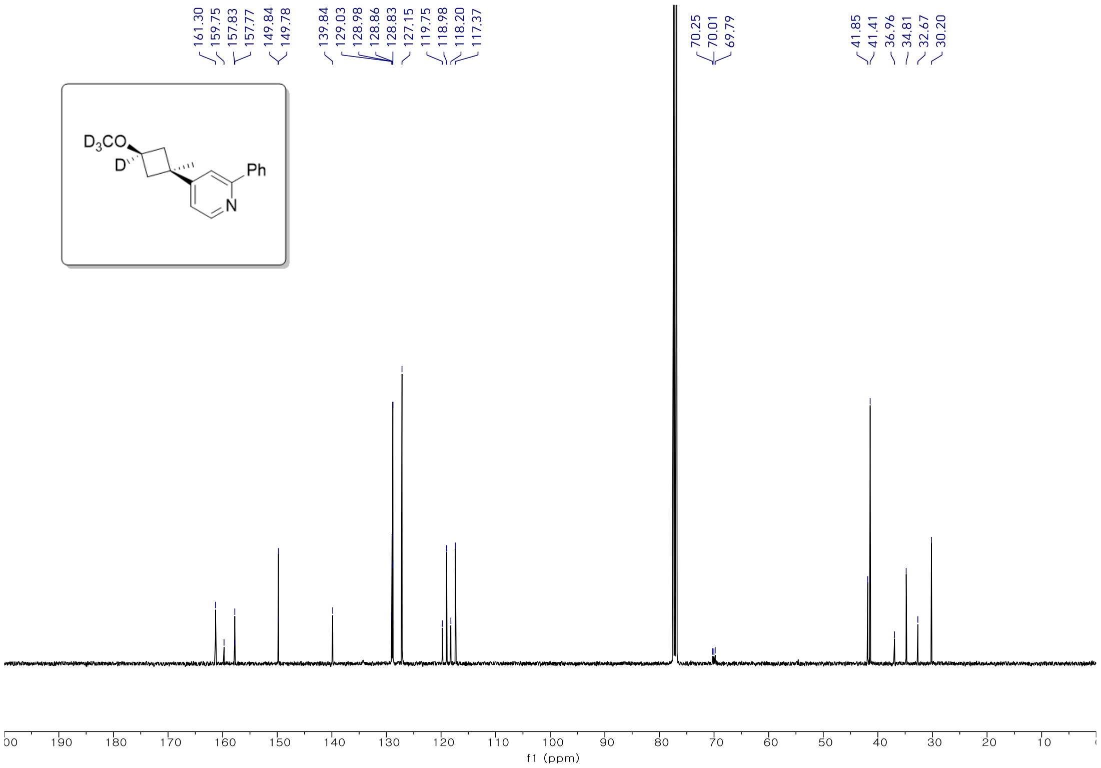

4-(pent-3-en-1-yl)-2-phenylpyridine (6ax).

500 MHz, ^{1}H NMR in $CDCl_3$

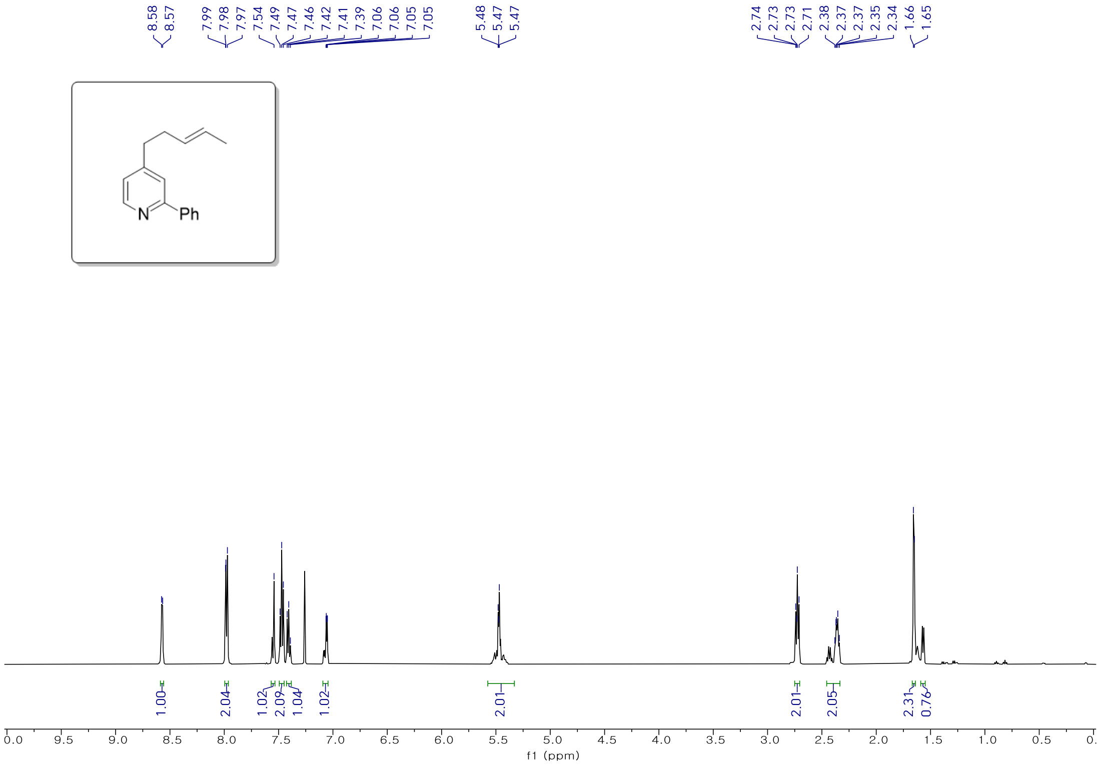

100 MHz, ^{13}C NMR in $CDCl_3$

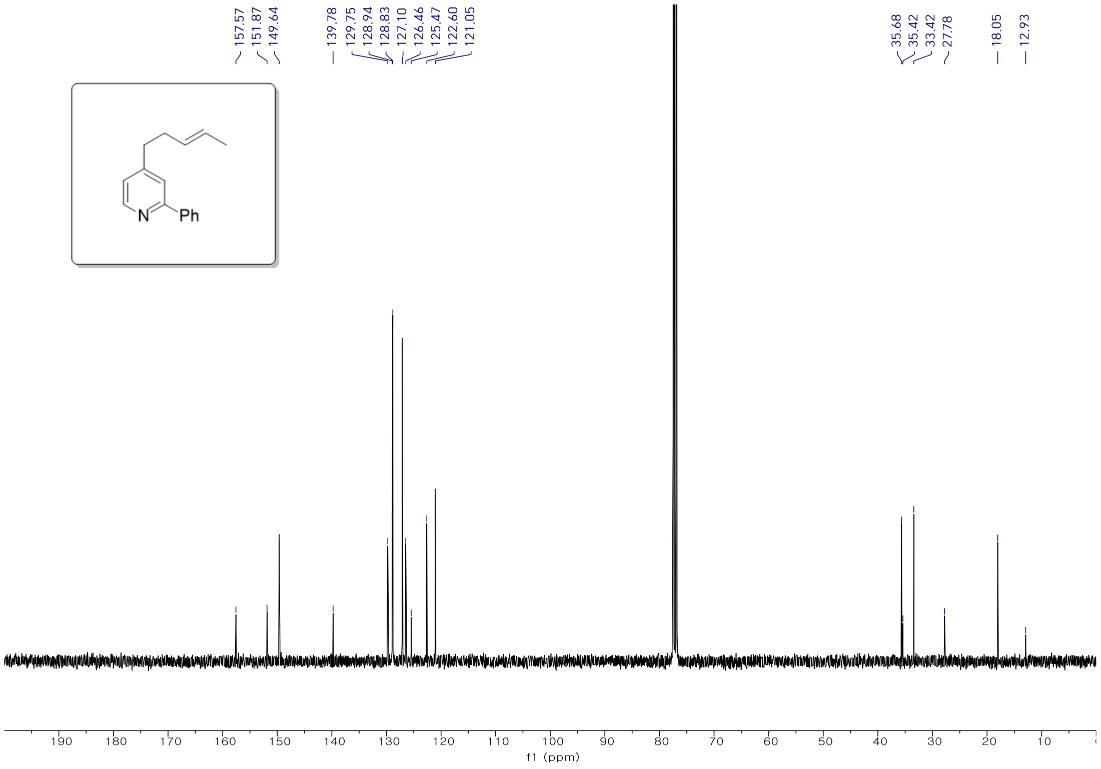

4-((2-methylcyclopentyl)methyl)-2-phenylpyridine (6ay).

500 MHz, ^{1}H NMR in $CDCl_3$

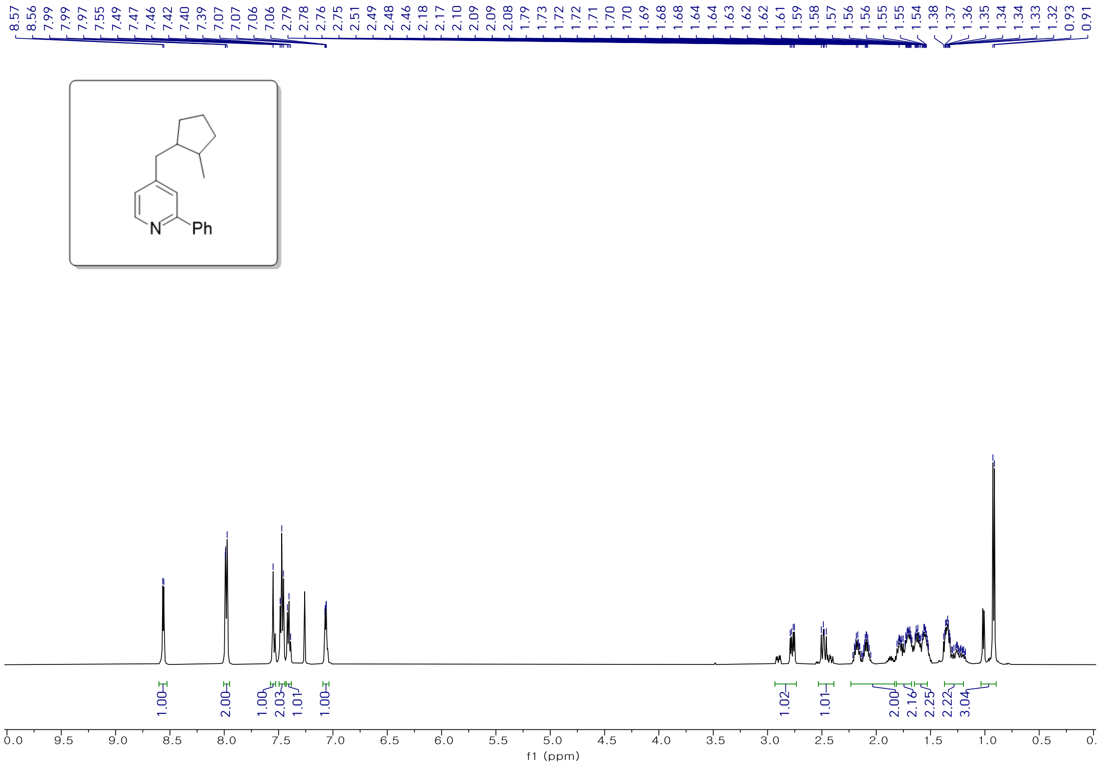

100 MHz, ^{13}C NMR in $CDCl_3$

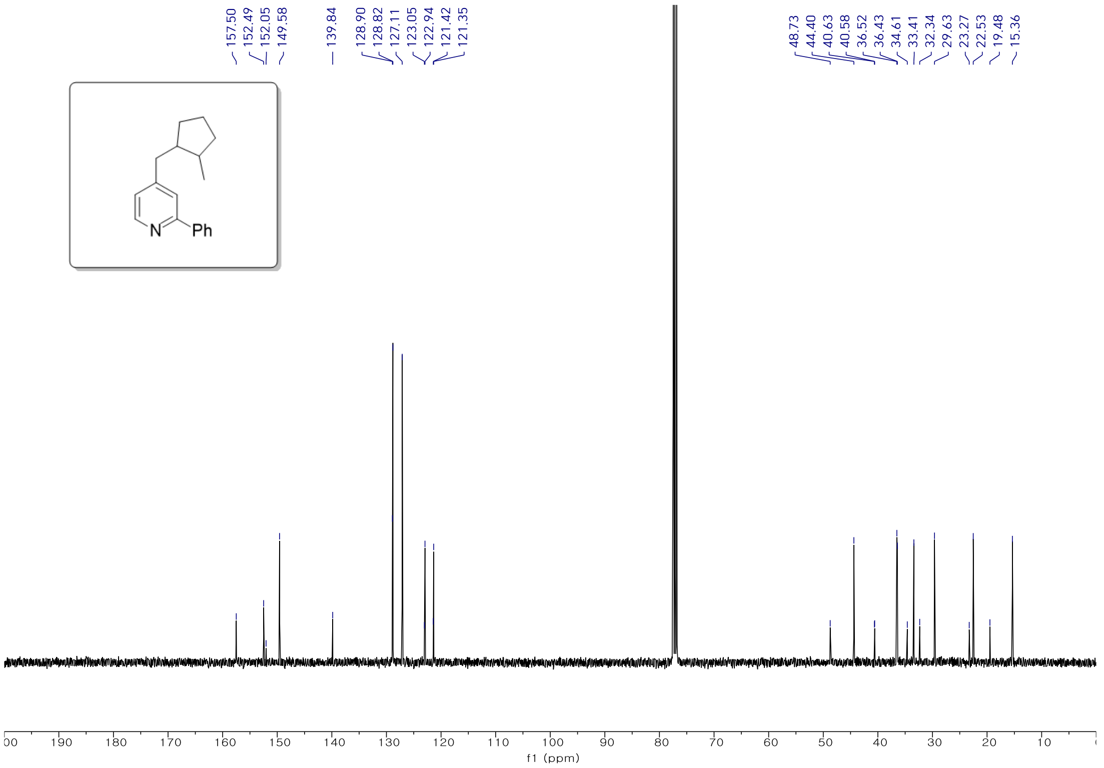

Supplementary References

(1) Moon, Y. *et al.* Visible light induced alkene aminopyridylation using N-aminopyridinium salts as bifunctional reagents. *Nat. Commun.* **10**, 4117 (2019)

(2) Klinger, D., Nilles, K. & Theato, P. Synthesis of polymeric 1-iminopyridinium ylides as photoreactive polymers. *J. Polym. Sci. A Polym. Chem.* **48**, 832–844 (2010)

(3) Johnston, K. A. *et al.* Concise routes to pyrazolo[1,5- a]pyridin-3-yl pyridazin-3-ones. *Org. Biomol. Chem.* **6**, 175–186 (2008)

(4) Kim, J. H., Ruffoni, A., Al-Faiyz, Y. S. S., Sheikh, N. S. & Leonori, D. Divergent Strain-Release Amino-Functionalization of [1.1.1]Propellane with Electrophilic Nitrogen-Radicals. *Angew. Chem. Int. Ed.* **59**, 8225–8231 (2020)

(5) Kirichok, A. A. *et al.* 1-Azaspiro[3.3]heptane as a Bioisostere of Piperidine. *Angew. Chem. Int. Ed.* **62**, e202311583 (2023)
